# Using behavioural theory to explore barriers and facilitators to physical activity in haemodialysis patients: an updated systematic review of qualitative evidence

**DOI:** 10.1080/21642850.2026.2707668

**Published:** 2026-07-27

**Authors:** Sarah Jasat, Roseanne E. Billany, Courtney J. Lightfoot, Amirah Essop-Adam, James O. Burton, Matthew P. M. Graham-Brown

**Affiliations:** a Leicester Partnership for Kidney Health Research, Division of Cardiovascular Sciences, School of Medical Sciences, and University of Leicester and NIHR Leicester Biomedical Research Centre, Leicester, UK; b Department of Renal Medicine, University Hospitals of Leicester NHS Trust, Leicester, UK

**Keywords:** Haemodialysis, behaviour theory, qualitative research, COM-B, physical activity

## Abstract

**Background:**

Physical activity (PA) improves health indicators for people receiving haemodialysis; however, participation remains low, and PA provision in routine care is inconsistent, despite recommendations from clinical practice guidelines. This review updates and integrates existing evidence on the attitudes towards PA and applies behaviour change theory to move beyond feasibility and identify components that support sustainable integration into routine clinical care.

**Methods:**

Five databases were systematically searched for studies reporting qualitative data on barriers and/or facilitators to PA (or its delivery) for adults undergoing maintenance haemodialysis and staff facilitating and observing. The findings were mapped to the Theoretical Domains Framework (TDF) and linked Capability, Opportunity, Motivation (COM-B) components, with candidate intervention functions identified through the Behaviour Change Wheel. A frequency analysis identified prominent domains from staff and patient data, and inductive thematic analysis within TDF domains identified barrier and facilitator themes.

**Results:**

Seventeen papers met the inclusion criteria. There were similarities in prominent TDF domains and themes between patient and staff data, and all areas of the COM-B were implicated as relevant. While all nine Behaviour Change Wheel intervention functions were identified, the APEASE-based assessment highlighted training, environmental restructuring, education, modelling, and enablement as the most feasible and relevant, with illustrative behaviour change techniques provided to support translation into practice.

**Conclusion:**

These findings identify areas of congruence in perceptions of barriers and facilitators across staff and patient groups, as well as key areas to consider when developing future interventions. Addressing these areas through the proposed intervention functions may enhance participation in PA and support sustained behaviour change.

## Key messages

- Environmental constraints and beliefs about the consequences of PA are the most prominent behavioural determinants, strongly shaping whether patients and staff view PA as feasible, safe, and worthwhile. These factors underpin many other barriers (e.g. safety concerns, disruption to dialysis, lack of resources) and therefore represent the most critical targets for intervention.
- Patients and staff share aligned beliefs about PA, recognising its potential benefits but also expressing similar fears about harm and disruption to dialysis, highlighting clear targets for education and reassurance in future interventions.
- Training and capability gaps remain major barriers, with patients often feeling too unwell or lacking confidence, and staff citing insufficient knowledge and practical skills to safely support PA.
- Reinforcement and social support act as powerful facilitators, with encouragement from staff, peers and family strongly influencing engagement and sustaining behaviour change.
- Using TDF-COM‑B mapping provides actionable guidance for implementation, identifying Training and Environmental Restructuring as key intervention functions to support the translation of PA guidelines into routine dialysis care.

## Introduction

While a lifesaving treatment for end-stage kidney disease (ESKD), maintenance haemodialysis is not without challenges and presents an indefinite, intense burden for patients (Jones et al., 2018). Treatment can be time-consuming and exhausting (Al-Naamani et al., 2024), with unpleasant side effects such as insomnia and muscle cramps (Flythe et al., 2019). Enforced sedentary time brought about by the dialysis schedule and symptoms such as fatigue increases the risk of cardiovascular disease (Cozzolino et al., 2018). A growing awareness of the inadequate quality of life experienced by dialysis patients has increased the urgency for options to lessen some of the burden (Mitema & Jaar, 2016).

Exercise and physical activity (PA) have been widely proposed to improve quality of life for patients receiving haemodialysis (Deligiannis et al., 2021; Gomes Neto et al., 2018). Despite this, the levels of PA within this population are low (Sutherland et al., 2021). The CYCLE-HD randomised controlled trial (RCT) demonstrated that a 6-month intradialytic cycling program significantly reduced left ventricular mass, and the PEDAL RCT, a similar program, demonstrated improvement in the physical component summary score of the Kidney Disease Quality of Life Short Form (Greenwood et al., 2021). Other PA studies aimed at this population, such as the EXCITE trial (Manfredini et al., 2017) and the DiaTT multicentre, cluster RCT (Anding-Rost et al., 2023) demonstrated improvements in tests such as the 6-minute walking test and the sit-to-stand. These studies demonstrated not only that exercise is safe for patients on haemodialysis, but also that it leads to meaningful improvements in important physiological outcomes relevant to these patients, such as cardiovascular disease and functional fitness. However, findings generally did not show improvements in overall quality of life or sustained PA behaviours. Clinical practice guidelines for exercise and lifestyle in chronic kidney disease recommend PA and exercise (Baker et al., 2022); however, these recommendations have not been widely translated into clinical practice (Wilund et al., 2020). Proposed barriers include staff shortages and uncertainty of evidence, as well as the difficulties of working with a deconditioned population (Jhamb et al., 2016).

Several reviews have focused on identifying barriers and facilitators to exercise, findings that are useful for designing effective interventions. However, to create sustained behaviour change through an intervention and beyond, understanding and targeting the underlying drivers of those behaviours is essential. To achieve this goal, the UK Medical Research Council guidelines for developing complex interventions advocate a theory-based perspective (Skivington et al., 2021). One behaviour change framework that is increasingly adopted is the Theoretical Domains Framework (TDF), which comprises 14 domains that influence behaviour (Cane et al., 2012). A large part of the value of the TDF is that its domains can be mapped to the Capability, Opportunity, Motivation-Behaviour (COM-B) model of behaviour change to provide a high-level view of behavioural determinants. Mapping to the COM-B also identifies targeted intervention functions and facilitates intervention development through the associated Behaviour Change Wheel (BCW) (Michie et al., 2011). Its use in this review enables the translation of qualitative insights into structured, theory-informed intervention strategies. The COM-B and BCW have been used to support intervention development in CKD-specific physical/activity programmes such as Kidney BEAM (Young et al., 2024), and their use in this review enables the translation of qualitative insights into structured, theory-informed intervention strategies.

Li et al. identified and reviewed qualitative studies exploring barriers and facilitators to PA in patients receiving haemodialysis and reported themes of disease distress, perceptions of exercise, environmental restrictions, spirit strength and hospital management (Li et al., 2021). The current work updates the identified literature and synthesises the results in the context of the TDF and COM-B. The aim of this updated review was to identify the components that future PA interventions and/or programs should focus on, to support the implementation of guidelines around PA and exercise for patients receiving dialysis.

## Methods

### Protocol and registration

**Figure 1.**
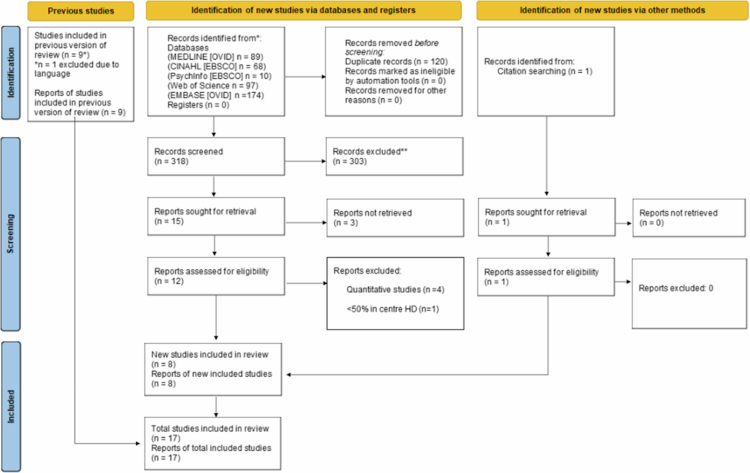
PRISMA flow diagram.

This review (PROSPERO: CRD420251047931) updates a previously published systematic review by Li et al. (2021) to include papers from the existing review and identify and integrate with newer publications. It is reported to be consistent with the Preferred Reporting Items for Systematic Reviews and Meta-Analyses (PRISMA) guidelines (Page et al., 2021) (Figure 1), and the protocol was prospectively registered (09/05/2025).

### Eligibility criteria

#### Types of studies

Qualitative or mixed-methods studies that explored barriers and facilitators to exercise and PA in patients receiving haemodialysis were included. Conference abstracts, theses, and review articles were excluded. Non-English language articles were excluded due to the difficulty in reliably interpreting barriers and facilitators in translated texts.

#### Types of participants

1. Patients (adults ≥18 years) receiving in-centre haemodialysis for >3 months.
2. Carers who live with patients receiving haemodialysis and/or provide emotional support and assistance in their daily lives.
3. Healthcare providers who work with patients receiving haemodialysis in any capacity, such as technicians, dialysis unit staff, nurses, physiotherapists or physicians.

Articles with <50% of participants receiving in-centre haemodialysis were excluded.

#### Types of outcomes

Barriers and facilitators affecting exercise and PA participation of patients receiving haemodialysis, as well as staff delivery, were reported by patients receiving haemodialysis, carers, or any staff members.

### Information sources

The following electronic databases were searched from July 2020 (end-date of the previous review search) to June 2025: MEDLINE (via OVID), EMBASE (via OVID), CINALH (via EBSCO), Web of Science, and PsycINFO (via OVID). The reference lists of the included studies and relevant reviews were also screened to identify additional eligible papers. Grey literature was not systematically searched. This decision was taken due to the focus on peer-reviewed qualitative studies and the challenges associated with conducting consistent quality appraisal and interpretive synthesis of non-peer-reviewed sources.

### Search strategy

The search strategy used comprehensive keyword combinations for the three concepts of interest: (1) Haemodialysis, (2) Exercise and Physical Activity, and (3) Qualitative study.

Full search strategies are available in Supplementary Material S1. Figure 1 shows the flow of information through each phase of the search.

### Data screening and extraction

Title and abstract screening were performed independently by two reviewers (SJ and REB). Disagreements were identified and resolved through discussion and consensus, with the involvement of a third reviewer where required.

Full-text screening was performed by a single reviewer (SJ), with all exclusions verified by a second reviewer (REB). Any uncertainties or disagreements were discussed and resolved through consensus.

Data extraction was performed by SJ and independently checked by REB.

Disagreements at both stages were infrequent and resolved through discussion.

#### Quality assessment

Risk of bias was assessed using the Critical Appraisal Skills Programme (CASP) checklist (Munabi‐Babigumira et al., 2015). Each study was required to meet the minimum objective criteria. Two authors (SJ and REB) independently reviewed each study using the checklist to reach consensus.

### Data analysis/synthesis

#### Theoretical domains framework coding

Barriers and facilitators were coded using the TDF/COM-B using the following method, adapted from a previously reported methodology (Booth et al., 2024):

1. Two reviewers (REB and SJ) independently coded data from the first seven papers, identified barriers and facilitators, and mapped these to TDF domains. Early mapping identified overlapping boundaries between TDF domains such as ‘Skills’ and ‘Beliefs about Capabilities’. To ensure consistency, this was discussed and a rationale to be followed was agreed, using the primary emphasis of participant accounts to guide the decision. Statements reflecting actual physical limitations (e.g. fatigue or comorbidity) were mapped to ‘Skills’, whereas evaluative beliefs about one’s ability to engage in physical activity were mapped to ‘Beliefs about Capabilities’. Examples of ambiguous quotes and mapping decisions are shown in Supplementary material S4. Discrepancies in coding and domain allocation were discussed and agreed upon through consensus.
2. Based on this initial double-coding phase, a codebook was developed to support consistent application of coding across studies.
3. One reviewer (SJ) coded the remaining papers, and coding/mapping was reviewed by a second reviewer (REB) against the established codebook. Any uncertainties or disagreements in coding or domain allocation were discussed and resolved through consensus, with reference to the established codebook and discussion within the wider author team where required. Newly formed barriers and facilitators were added iteratively to the codebook (Supplementary Material S2).
4. Where barriers or facilitators were broad or encompassed multiple concepts and therefore difficult to code accurately, they were split into multiple barriers/facilitators based on available data. To minimise inflation of frequency counts due to code splitting, broad barriers and facilitators were only divided where there was sufficient conceptual distinction within the source data, and consistent coding rules were applied across all studies. Once the split was agreed, the same process as above was applied to the new barriers and facilitators (all decisions on splitting can be found in Supplementary Material S3).
5. Codes were categorised according to data source (patients and staff). Staff data included perspectives from non-physician staff working in the HD setting, and physician data included perspectives from any medical doctor, whether working academically or clinically.
6. The number of times a barrier or facilitator was coded to a domain was recorded in a frequency analysis. Prominence was determined using a combination of (1) frequency of coding to a given domain and (2) prevalence across included studies. Domains represented in more than 50% of included papers were first identified, after which frequency counts were used to rank these domains, with the three highest-frequency domains categorised as ‘prominent’.

For mixed-methods studies, only findings derived from the qualitative component were extracted and analysed. Mixed-methods and purely qualitative studies were treated equivalently during coding and synthesis, with no differential weighting applied.

#### Thematic synthesis

Once coded and grouped within TDF domains, inductive thematic analysis was performed within each domain and for each participant group following the approach described by Braun and Clarke (2006). Similar barriers and facilitators were grouped into candidate themes within each domain. Themes were developed by interpreting patterns across studies, drawing on both the context of grouped barriers and facilitators and the original extracted data fragments. Themes were refined iteratively by two reviewers (SJ and REB), then further refined following discussion with the wider authorship team, including experienced qualitative thematic analysis researchers, until the themes were considered to capture both the core point and nuances of the data that evidenced it.

#### Sensitivity analysis

To ascertain confidence in the transferability of findings, a sensitivity analysis was done for IDE-only papers. The staff and patient frequency analysis were recounted, including only IDE papers, and prominent domains were assigned based on the new counts for both participant groups. The thematic analysis was also revisited to identify themes that became thin or disappeared with IDE-only data.

#### Confidence in findings

While a formal CERQual assessment was not undertaken, confidence in findings was considered in relation to cross-study prevalence, frequency of coding, and consistency of themes across data sources. Domains identified as prominent therefore reflect areas with greater breadth and depth of supporting evidence, although less frequently reported themes may still hold importance.

#### Researcher positionality

The researchers conducting this analysis had backgrounds in renal medicine, exercise physiology, and qualitative research with experience in developing and evaluating PA interventions in chronic kidney disease populations. As such, the research team held prior assumptions about the potential benefits of PA and the importance of its inclusion into routine care. Throughout the analytic process, these perspectives were reflected upon during coding and theme development to ensure that interpretations remained grounded in the data. Regular discussions between multiple researchers and involvement of the wider multidisciplinary author team supported reflexive consideration of alternative interpretations.

#### Ethics statement

This study did not involve the generation or collection of new data; therefore, ethical approval or exemption from an Institutional Review Board/Ethics Committee was not required.

## Results

### Characteristics of included studies

In total, 318 records were screened, and 8 new studies were identified (Figure 1). Although Li et al. included 10 studies (Heiwe & Tollin, 2012; Jhamb et al., 2016; Kontos et al., 2007; Liu et al., 2020; Painter et al., 2014; Sieverdes et al., 2015; Song et al., 2019; Sutherland et al., 2021; Thompson et al., 2016; Yongxin et al., 2015), as one study was written in Chinese (Yongxin et al., 2015), this was inaccessible and thus excluded from our updated review. The updated review included 17 studies as described in Table 1. Three studies were mixed-methods, and the remaining were qualitative. All studies included barriers and facilitators to exercise/PA in patients receiving haemodialysis. Eight studies referred to a specific intervention, and the remaining nine investigated exercise/PA in general. Of the studies linked to an intervention, six specifically referred to intradialytic exercise (IDE) and two to a non-IDE intervention. The non-intradialytic interventions were a virtual reality-based rehabilitation program and a pedometer-based walking intervention.

**Table 1.** Study characteristics.

#	Study	Country	*n*	Type of participants (*n*)	Methodology	Type	Intervention*	IDE**	Data collection	Analysis
1	Song et al. (2019)	China	44	Patients (44)	Not stated	Qualitative	No	No	Semi-structured interview	Content analysis
2	Kontos et al. (2007)	Canada	49	Patients (18)	Not stated	Qualitative	No	No	Focus groups	Inductive thematic approach
				Staff (24)						
				Family caregivers (7)						
3	Sieverdes et al. (2015)	U.S.A.	22	Patients (22)	Ground theory	Qualitative	No	No	Key informant interview	Content analysis
4	Thompson et al. (2016)	Canada	36	Patients (25)	Interpretive description	Qualitative	Yes	Yes	Interview	Thematic analysis
				Staff (11)						
5	Sutherland et al. (2021)	UK	20	Patients (20)	Not stated	Mixed Methods	No	No	Semi-structured interview	Framework method
6	Liu et al. (2020)	U.S.A.	10	Patients (10)	Not stated	Qualitative	No	No	Semi-structured interview	Framework method
7	Heiwe and Tollin (2012)	Sweden	10	Patients (10)	Not stated	Qualitative	Yes	Yes	Semi-structured interview	Phenomenography
8	Jhamb (2016)	U.S.A.	36	Patients (16)	Not stated	Qualitative	No	No	Semi-structured interview	Inductive manner
				Staff (14)						
				Nephrologists (6)						
9	Painter et al. (2014)	U.S.A.	21	Staff (15)	Focused ethnography	Qualitative	No	No	Semi-structured interview	Descriptive and interpretive
10	Hu et al. (2025)	China	10	Patients (10)	Phenomenology	Qualitative	Yes	No	Semi-structured interview	Colaizzi seven-step analysis method
11	Zelko et al. (2024)	Slovakia	21	Staff (16)	Not stated	Qualitative	Yes	Yes	Semi-structured interviews	Thematic analysis
				Nephrologists (5)						
12	Wodskou (2021)	Denmark	20	Patients (8)	Not stated	Qualitative	Yes	Yes	Semi-structured interview (patients)	Content Analysis
				Staff (12)					Focus groups (staff)	
13	Castillo et al. (2022)	Canada	43	Patients (17)	Not stated	Qualitative	Yes	Yes	Semi-structured interviews	Content Analysis
				Staff (19)						
				Nephrologists (7)						
14	Rothpletz-Puglia (2022)	U.S.A.	15	Patients (15)	Not stated	Mixed Methods	No	No	Semi-structured interviews	Thematic analysis
15	Huang, (2023)	China	19	Patients (19)	Not stated	Qualitative	No	No	Semi-structured interviews	Content Analysis
16	Sheshadri, (2020)	U.S.A.	30	Patients (30)	Not stated	Mixed Methods	Prior to and during	No	Semi-structured interviews	Thematic Analysis with Framework
17	Young (2015)	UK	52	Patient (35) Staff (17)	Not stated	Qualitative	Prior to and during	Yes	Semi-structured interviews and focus groups	Thematic Analysis with Framework

^*^ Denoting whether the paper explores attitudes to PA in connection with a specific intervention.

^**^ Denoting whether the paper explores attitudes to IDE.

Abbreviation: IDE; intradialytic exercise.

### Participant characteristics

This updated review included 282 patients, 128 dialysis staff members, 18 physicians and 7 caregivers. The included articles were published between 2007 and 2024. Three studies were based in China (Hu et al., 2025; Huang et al., 2023; Song et al., 2019), three in Canada (Castillo et al., 2022; Kontos et al., 2007; Thompson et al., 2016), six in the United States (Jhamb et al., 2016; Liu et al., 2020; Painter et al., 2014; Rothpletz-Puglia et al., 2022; Sheshadri et al., 2020; Sieverdes et al., 2015), two in the United Kingdom (Sutherland et al., 2021; Young et al., 2015) and one each in Sweden (Heiwe & Tollin, 2012), Slovakia (Zelko et al., 2024), and Denmark (Wodskou et al., 2021) (Table 1).

### Quality assessment

All included articles scored between 9 and 14 on the criteria for the CASP checklist (Munabi‐Babigumira et al., 2015) with an average score of 12.8, inferring satisfactory methodological quality (Supplementary Material S5). Typically, research questions were clearly stated and appropriate for qualitative methodology. The checklist scores were overall high, with sampling, data collection and analysis appropriately and clearly described. Common reasons for losing points were not describing the role of the researcher clearly, not describing the sampling method clearly or analysing whether appropriate, and not including in-text any consideration for ethical issues.

### Prominent TDF domains for barriers and facilitators

All studies reported barriers and facilitators. A total of 126 barriers and 128 facilitators were identified. Barriers and facilitators identified, along with frequency analyses, are shown in Table 2 for patients and Table 3 for staff (Supplementary Material S3 for study-level mapping to TDF domains). Across all TDF domains, there were 20 facilitator themes for patients and 16 for staff, with 23 barrier themes for patients and 21 for staff. Barrier and facilitator themes for each domain are presented in Table 4 with prominent domains highlighted. A full thematic analysis is presented in Supplementary Material S6, with illustrative quotes. While carer data was also coded for barriers and facilitators and mapped to TDF domains (Supplementary Material S3), only one paper included these perspectives and therefore further analysis was not possible.

**Table 2.** Frequency analysis of TDF domains coded by barriers and facilitators in the patient dataset.

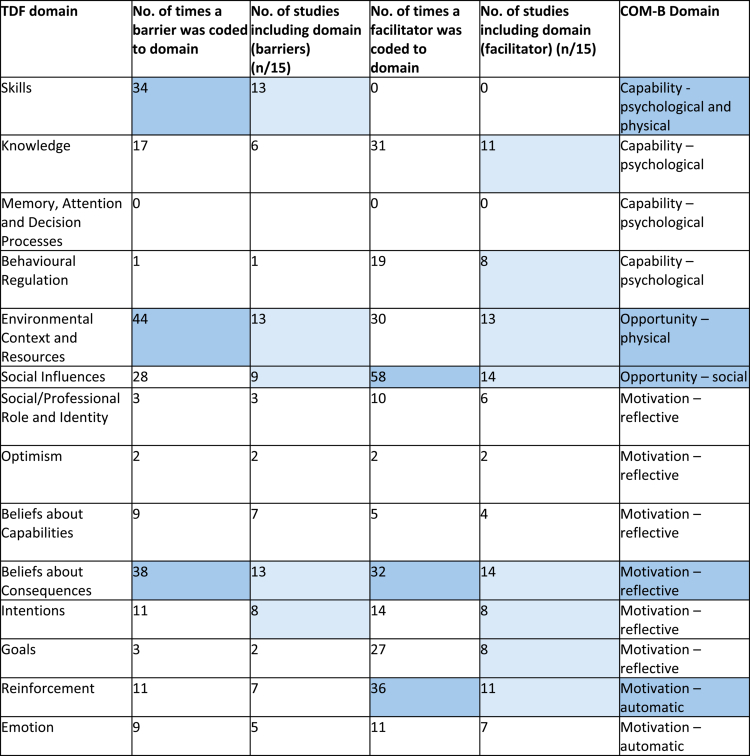

Note: Domains coded by ≥50% of papers in light blue, of these, the top 3 ranked by frequency are defined as prominent domains in dark blue.

**Table 3.** Frequency analysis of TDF domains coded by barriers and facilitators in the staff dataset.

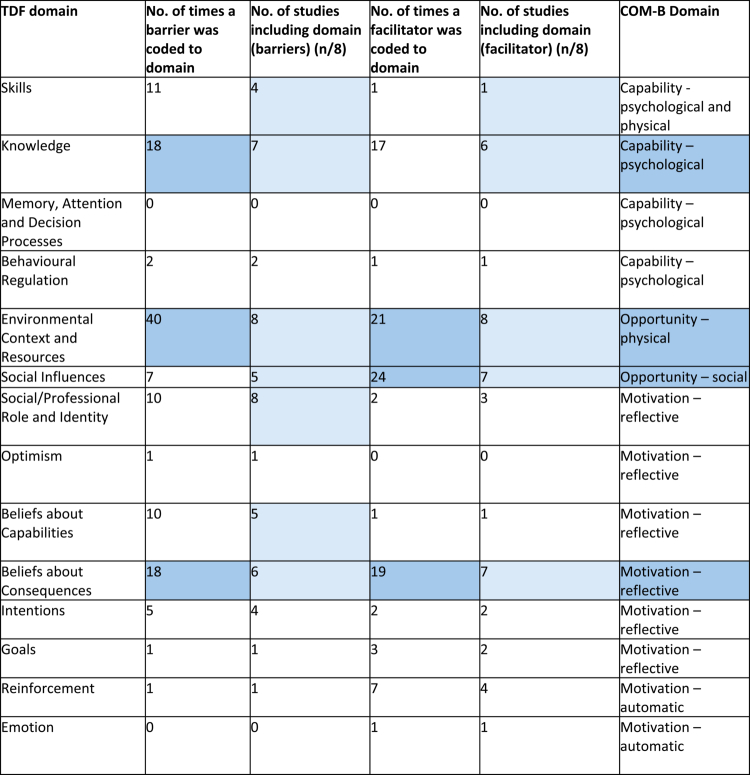

Note: Domains coded by ≥50% of papers in light blue. Of these, the top 3 ranked by frequency are defined as prominent domains in dark blue.

**Table 4.** Patient and staff themes within TDF Domains with associated COM-B construct.

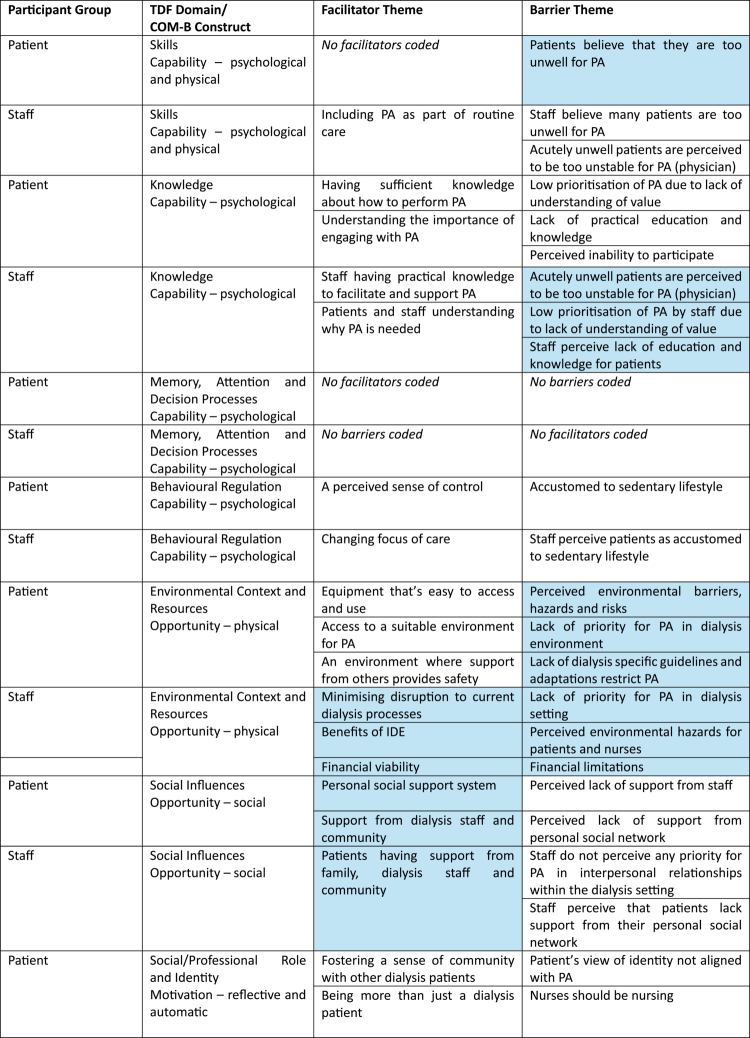
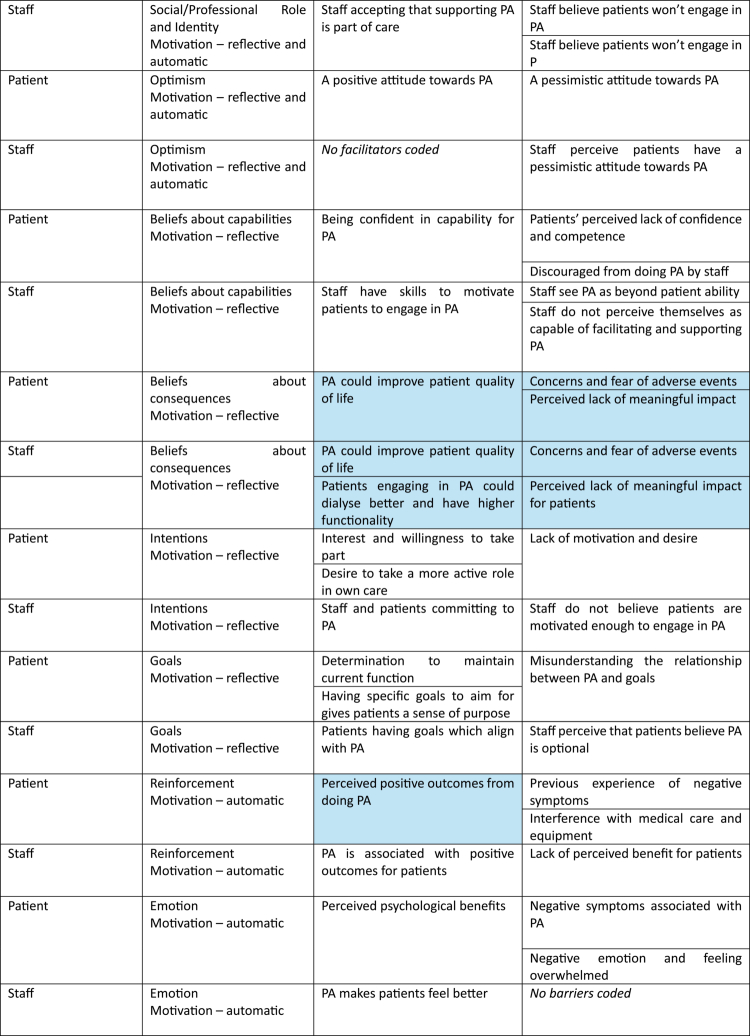

Note: The top 3 domains are shaded in blue and are discussed within the narrative results text.

Table 4 shows facilitator and barrier themes for patients and staff, grouped by TDF domains. Themes for the most prominent 3 domains for barriers and for facilitators, as determined by the frequency analysis (Tables 2 and 3), are detailed below. ‘Staff’ data encompasses perceptions of both non-physician dialysis staff and physicians, and distinctions are made where possible. The non-physician dialysis staff included nurses, technicians, dietitians, social workers, service workers, training assistants, healthcare assistants, managers and administrators. The physician group included patient-facing staff and academics.

### Prominent domains for patient data and accompanying themes

#### Patient barriers

##### TDF domain: environmental context and resources

‘Environmental context and resources’ was a prominent domain for barriers, coded 44 times, across 13/16 papers, which included patient perspectives. Four themes were defined.

Theme 1: Perceived environmental barriers, hazards and risks

The participants voiced concerns about the risk of adverse events related to perceived hazards in the environment, both within the dialysis centre (Heiwe & Tollin, 2012) and outside (Huang et al., 2023; Sheshadri et al., 2020; Song et al., 2019). Outside the dialysis centre, opportunities for PA were perceived to be limited (Song et al., 2019), ‘Risks of injury’ and ‘crowded environment’ (Song et al., 2019) were seen as deterrents to utilising public exercise facilities, while icy pavements, steps and obstacles in their local environment made it difficult for patients to access low entry barrier PA, such as walking around their neighbourhood and outside (Huang et al., 2023; Sheshadri et al., 2020; Song et al., 2019). Of the data reflecting in-centre PA, there was trepidation around the use of IDE bikes: ‘Well, it wasn’t quite firm, it moved around a bit…’ (Heiwe & Tollin, 2012).

Theme 2: Lack of priority for PA in the dialysis environment

Half the barriers within this domain (13/26) were related to the lack of priority for PA in the dialysis environment. This was particularly notable in papers analysing in-centre PA, with patients concerned about creating extra work for nurses (Young et al., 2015) and disrupting the routine of the centre (Heiwe & Tollin, 2012; Song et al., 2019; Thompson et al., 2016; Wodskou et al., 2021). A participant in one paper worried that movement from the PA could set off the alarm on their dialysis machine: ‘I just have to move this hand here, and the machine starts to roar’ (Wodskou et al., 2021). The lack of PA recommendations in existing resources (Song et al., 2019) and the requirement for patients to self-refer for PA (Castillo et al., 2022) highlighted the low priority of PA, even when IDE was not the focus. Other barriers highlighted that the dialysis environment was not set up to facilitate PA, with some centre layouts limiting interactions between patients and opportunities for peer support (Heiwe & Tollin, 2012) and no appropriate route to request support (Thompson et al., 2016).

Theme 3: Lack of dialysis-specific guidelines and adaptations restricts PA

Another commonly cited barrier to PA was the specific limitations dialysis patients experience compared to the rest of the population. Due to their deconditioned state, in some cases the patients reported finding PA equipment difficult to use (Sheshadri et al., 2020), or over-strenuous (Hu et al., 2025). Other dialysis-specific issues, such as fistulas or catheters, meant that activities such as swimming or weightlifting were not recommended (Jhamb et al., 2016). For IDE, there were limitations on what could be done without disturbing the access arm (Jhamb et al., 2016) or for patients who dialyse lying down (Kontos et al., 2007).

##### TDF domain: beliefs about consequences

‘Beliefs about consequences’ was a prominent domain for barriers, coded 38 times, across 13/16 papers, which included patient perspectives. Two themes were defined.

Theme 1: Concerns and fear of adverse events

Participants expressed concerns about possible negative outcomes from engaging in PA. These covered a range from worries about getting sick (Song et al., 2019), of sustaining an injury (Huang et al., 2023; Jhamb et al., 2016; Sheshadri et al., 2020; Song et al., 2019; Young et al., 2015), falling (Huang et al., 2023; Jhamb et al., 2016; Sheshadri et al., 2020; Sutherland et al., 2021), or experiencing pain (Heiwe & Tollin, 2012; Sutherland et al., 2021), as well as other issues that would negatively impact their treatment experience, such as increased fatigue (Heiwe & Tollin, 2012; Huang et al., 2023) or disrupting dialysis (Young et al., 2015). Worries about damaging the fistula were mentioned for both IDE (Heiwe & Tollin, 2012; Kontos et al., 2007; Young et al., 2015) and out-of-centre exercise (Huang et al., 2023; Jhamb et al., 2016; Sutherland et al., 2021). They also worried about the nurses being burdened (Heiwe & Tollin, 2012; Young et al., 2015) and that requesting help with IDE could negatively affect the medical support they received from staff (Thompson et al., 2016).

Theme 2: Perceived lack of meaningful impact

Many patients did not see the potential for PA to improve their lives. Some patients reported a lack of perceived benefit (Kontos et al., 2007; Sutherland et al., 2021), expressing a disillusioned attitude toward any improvement in their lives: ‘what good is it doing to me?’ Others expressed doubts about the benefits (Castillo et al., 2022) or held the view that PA was optional (Sieverdes et al., 2015).

##### TDF domain: skills

‘Skills’ was a prominent domain for barriers, coded 34 times, across 13/16 papers, which included patient perspectives. One theme was defined.

Theme 1: Patients believe that they are too unwell for PA

The lack of physical and mental capacity required to engage in PA was mentioned in several studies. These included dialysis-specific side-effects, such as fatigue, as well as other health conditions common in this population, such as heart disease (Huang et al., 2023; Kontos et al., 2007; Rothpletz-Puglia et al., 2022; Sheshadri et al., 2020; Song et al., 2019). The ongoing presence of pain and discomfort was reported as a barrier (Heiwe & Tollin, 2012; Huang et al., 2023; Kontos et al., 2007; Sheshadri et al., 2020) and patients also described feelings of depression and hopelessness, (Kontos et al., 2007): ‘You say to yourself, ‘it’s always some other thing’ and ‘what good is it doing to me?’ and you can get very depressed about it. And I think that takes away the will to do any exercise.’

#### Patient facilitators

##### TDF domain: social Influences

Social Influences was a prominent domain for facilitators, coded 58 times, across 14/16 papers, which included patient perspectives. Two themes were defined.

Theme 1: Personal social support system

Support from care partners (Sheshadri et al., 2020), family (Huang et al., 2023; Jhamb et al., 2016; Liu et al., 2020; Sheshadri et al., 2020; Sutherland et al., 2021), and their personal network (Huang et al., 2023; Jhamb et al., 2016; Liu et al., 2020; Sieverdes et al., 2015; Song et al., 2019) was valuable in encouraging PA. In some cases, family members or care partners facilitated the patient to exercise, often by creating opportunities for PA or providing company (Huang et al., 2023; Liu et al., 2020). A desire to be healthy to spend time with family was also noted as a motivation (Sheshadri et al., 2020): ‘I just want to be able to do the things I used to do. Stand up straight and tall. Go on hikes with my family’.

Theme 2: Support from dialysis staff and community

Many facilitators for PA were based on connections with dialysis staff or peers in the dialysis community. Interactions with other patients were said to inspire or encourage (Castillo et al., 2022; Hu et al., 2025; Huang et al., 2023; Jhamb et al., 2016; Thompson et al., 2016; Young et al., 2015), and many papers with an IDE focus referred to a sense of camaraderie and shared identity (Heiwe & Tollin, 2012; Jhamb et al., 2016; Thompson et al., 2016; Wodskou et al., 2021). Additionally, seeing inactive peers decline represented a cautionary tale (Sheshadri et al., 2020). Support from staff could be through tailored guidance and recommendations about PA or general encouragement (Castillo et al., 2022; Hu et al., 2025; Huang et al., 2023; Jhamb et al., 2016; Thompson et al., 2016; Wodskou et al., 2021) and covered many different types of staff.

##### TDF domain: reinforcement

Reinforcement was a prominent domain for facilitators, coded 36 times, across 11/16 papers, which included patient perspectives. One theme was defined.

Theme 1: Perceived positive outcomes from doing PA

Patients perceived many positive outcomes from engaging in PA, such as a sense of pride and satisfaction (Huang et al., 2023; Kontos et al., 2007) and enjoyment (Hu et al., 2025; Huang et al., 2023; Sutherland et al., 2021; Thompson et al., 2016). Some quotes highlighted that IDE passed the time more agreeably and made ‘the dialysis environment a lot more pleasant’ (Thompson et al., 2016). Many patient comments indicated that they had seen improvement in energy levels (Castillo et al., 2022; Huang et al., 2023; Song et al., 2019) and blood pressure (Jhamb et al., 2016; Kontos et al., 2007). Patients expressed hope that this improvement would continue and allow them to control decline or improve function: ‘Since I’ve been ill, I can’t do any work around the house, and I can’t look after my two children, so it’s good to know that I’m confident that I can get back to my previous physical condition now that I’m using this machine’ (Hu et al., 2025).

##### TDF domain: beliefs about consequences

‘Beliefs about consequences’ was a prominent domain for facilitators, coded 32 times across 14/16 papers, which included patient perspectives. One theme was defined.

Theme 1: PA could improve patients’ quality of life

Patients had many positive expectations associated with PA and beliefs about the benefits linked to PA. Most papers demonstrated a general belief that PA could help physical wellbeing, with additional benefits of weight control (Huang et al., 2023; Sheshadri et al., 2020; Sieverdes et al., 2015), stress management (Sieverdes et al., 2015), and help maintain independence (Huang et al., 2023; Rothpletz-Puglia et al., 2022; Sutherland et al., 2021; Wodskou et al., 2021). Improvements were sometimes articulated clearly with a specific outcome, such as taking less medication to control blood pressure (Kontos et al., 2007; Young et al., 2015), and sometimes described in more general terms: ‘I wouldn’t be doing quite as well as I am doing now if I wasn’t doing some form of exercise’ (Jhamb et al., 2016).

### Prominent domains for staff data and accompanying themes

#### Staff barriers

##### TDF domain: environmental context and resources

‘Environmental context and resources’ was a prominent domain for barriers, coded 39 times, across 7/8 papers, which included staff and physician perspectives. Three themes were defined.

Theme 1: Lack of priority for PA in the dialysis setting

The largest proportion of barrier codes (12/19) was summarised within this theme, which described a culture within the dialysis centre that did not include PA. Many other factors were prioritised over PA, such as the other tasks of nurses (Castillo et al., 2022; Kontos et al., 2007; Thompson et al., 2016; Wodskou et al., 2021; Young et al., 2015) or patients retaining their transport privileges (Kontos et al., 2007). Systematic neglect of PA was evident in the lack of resources (Painter et al., 2014), and dialysis centres and routines were not set up to support PA, with some staff worried that IDE could disrupt dialysis (Castillo et al., 2022; Wodskou et al., 2021; Zelko et al., 2024) and present an additional source of pressure (Castillo et al., 2022; Jhamb et al., 2016; Young et al., 2015). Even where attempts were made to introduce PA, the piecemeal, unplanned application led to issues, such as IDE equipment that wasn’t suitable for deconditioned patients (Jhamb et al., 2016), and the centres struggled to meet the logistical and practical needs (Castillo et al., 2022).

Theme 2: Financial limitations

Dialysis staff and physicians acknowledged that finances could be a barrier to PA (Painter et al., 2014; Zelko et al., 2024). Some concerns were in relation to patients being from low-income backgrounds and not being able to access PA opportunities that required payment (Painter et al., 2014). The cost of providing PA in the dialysis centre was also identified as a barrier, with physical resources such as exercise equipment specifically mentioned as something that needed to be evaluated: ‘I don’t know if we’re going to want to spend all this money on some sort of equipment and have half the patients not use it’ (Jhamb et al., 2016). Additionally, participants expressed uncertainty about financing additional staff to supervise and support PA (Zelko et al., 2024).

Theme 3: Perceived environmental hazards for patients and nurses

Both the dialysis and public environments were described as presenting many hazards for patients receiving haemodialysis. A barrier for IDE was the concern that exercise equipment may hinder access to patients, particularly in an emergency (Castillo et al., 2022; Kontos et al., 2007; Wodskou et al., 2021), as well as generally due to space limitations and the need for dialysis ‘machines, tubes, and power cords’ (Wodskou et al., 2021). Staff also described difficulties nurses faced when moving exercise equipment, specifically the bicycle used to IDE, noting that its size and weight were obstacles to use (Castillo et al., 2022; Kontos et al., 2007).

##### TDF domain: beliefs about consequences

‘Beliefs about consequences’ was a prominent domain for barriers, coded 20 times, across 6/8 papers, which included staff and physician perspectives. Two themes were defined.

Theme 1: Concerns and fear of adverse events

Concerns about patient or staff injury were raised in several papers. These ranged from specific fears of patients damaging the fistula (Kontos et al., 2007; Zelko et al., 2024) or falling off IDE equipment (Jhamb et al., 2016; Zelko et al., 2024) to general safety concerns (Kontos et al., 2007; Wodskou et al., 2021; Zelko et al., 2024). Staff also expressed concerns about injuring themselves moving the IDE equipment (Kontos et al., 2007). In addition to physical injury, some concerns were raised about patients losing their transport benefits (Kontos et al., 2007) or causing disruptions to the dialysis process (Kontos et al., 2007; Zelko et al., 2024).

Theme 2: Perceived lack of meaningful impact for patients

Staff and physicians expressed doubt about whether PA could offer tangible benefits (Castillo et al., 2022; Young et al., 2015). For some staff, this was related to uncertainty about the evidence (Castillo et al., 2022). In contrast, others had heard negative comments about introducing IDE: I have only ever heard negatives [about IDE]’ (Young et al., 2015), leading them to believe it would be unsuccessful.

##### TDF domain: knowledge

Knowledge was a prominent domain for barriers, coded 17 times, across 8/8 papers, which included staff and/or physician perspectives. Three themes were defined.

Theme 1: Low prioritisation of PA by staff due to a lack of understanding of value

There were several examples in the dataset of staff not assigning value to PA due to insufficient information. Some staff did not appear to have in-depth and accurate knowledge of the benefits of PA (Jhamb et al., 2016; Kontos et al., 2007), and were often unaware of resources for PA for patients (Kontos et al., 2007; Painter et al., 2014). One quote highlighted that staff may see the benefits as limited to mood: ‘exercise is just a qualitative thing whether you feel good or not and I don’t think that is a high priority’ (Kontos et al., 2007).

Theme 2: Staff lack of practical education and knowledge of PA (staff and physician theme)

A recurring concern among staff across different papers was practical matters related to PA. These included not knowing which patients were safe to exercise (Castillo et al., 2022) and what was correct to instruct them (Castillo et al., 2022; Painter et al., 2014; Thompson et al., 2016). Staff expressed uncertainty in advising or supporting patients with PA and IDE, particularly when the patients were not confident (Thompson et al., 2016). A junior staff focus group from one study pointed out that staff would need practical knowledge of how to use any equipment that was involved in a PA program: ‘We should all get shown how to use the machine. That would throw a spanner in the works if people say they didn’t know how to work it’ (Young et al., 2015).

Theme 3: Staff perceive a lack of education and knowledge for patients (staff and physician theme)

Staff noted a lack of practical information about PA for patients, addressing concerns such as blood pressure and fistula safety, with one participant expressing that PA could be incorporated into patient education to motivate them (Kontos et al., 2007). Staff and physician participants pointed out a lack of structure in the guidance that was provided: ‘The doctors just say, ‘Well, you need to lose weight, you need to exercise.’ But it’s not super helpful’ (Painter et al., 2014). The problem with lack of guidance and structure was echoed in pre-implementation discussions around IDE: ‘if you just bring pedals to a unit and you say here you go that’s where you’re likely gonna fail because we [nurses, technicians and doctors] lack that ability to really assess and tailor the programs to patients individually’ (Castillo et al., 2022).

#### Staff facilitators

##### TDF domain: social Influences

‘Social influences’ was a prominent domain for facilitators, coded 24 times, across 7/8 papers, including staff perspectives. One theme was defined.

Theme 1: Patients having support from family, dialysis staff and community

Patients were seen to be encouraged to engage in PA through the social influence of family outside the dialysis centre and of staff and peers within. Family’s attitude to PA was seen as important: ‘Most have a spouse or a sibling or child or parent that’s working with them in part of their lives. That person’s buy-in could be motivating’ (Jhamb et al., 2016). Peer influences within the dialysis unit were also suggested to be encouraging (Jhamb et al., 2016; Wodskou et al., 2021): ‘Maybe if they are seeing their friends across the aisle doing it, then that might motivate them to do it as well’ (Jhamb et al., 2016).

Healthcare professionals and dialysis staff were described as providing reassurance through recommendation (Jhamb et al., 2016; Thompson et al., 2016; Wodskou et al., 2021; Zelko et al., 2024) and tailored guidance (Castillo et al., 2022; Wodskou et al., 2021; Young et al., 2015; Zelko et al., 2024), and supportive in providing encouragement (Jhamb et al., 2016; Thompson et al., 2016; Wodskou et al., 2021; Young et al., 2015). The roles of senior staff, such as physicians (Young et al., 2015), along with physiotherapists and nurses (Wodskou et al., 2021), were specifically mentioned as influential. In particular, providing encouragement and tertiary support was suggested to be practical for staff, especially if a collaborative approach involving all grades was adopted: ‘Most of the staff I think would encourage the patients and have fun with it and help the patient have fun with it’ (Jhamb et al., 2016).

##### TDF domain: environmental context and resources

‘Environmental context and resources’ was a prominent domain for facilitators, coded 22 times, across 8/8 papers, which included staff and physician perspectives. Three themes were defined.

Theme 1: Minimising disruption to current dialysis processes

Staff data indicated a preference for PA options which would not affect current dialysis processes or would affect them minimally. Having additional staff to provide the PA program was suggested: ‘Well, there should be some staff available to deal with it and to do it. Basically, our nurses are quite busy’ (Painter et al., 2014). Where IDE was the subject, equipment that was easy to use, move and maintain (Kontos et al., 2007; Wodskou et al., 2021), thus requiring less support from staff (Jhamb et al., 2016; Thompson et al., 2016; Wodskou et al., 2021; Young et al., 2015) was preferable, and if this was in place then existing staff would be able to make minor adjustments and provide encouragement: ‘I think we have to do some minor adjustments on the bikes; seems to be a little bit more tension, just a little bit less tension, that’s something it’s quickly, we can do that and walk away’ (Thompson et al., 2016).

Theme 2: Benefits of IDE

Both staff and physicians acknowledged the benefits of PA during dialysis. The idea of exercising at the dialysis centre or while on dialysis was considered ‘convenient’ and a way to ‘turn that time into productivity’ and to avoid using precious free time (Jhamb et al., 2016). Physician data suggested that PA during dialysis could have safety benefits compared with PA at home (Jhamb et al., 2016).

Theme 3: Financial Viability

Staff and physician data highlighted the need for low-cost resources in any PA intervention. While data on this topic were limited, a statement from one nephrologist demonstrated an awareness of inexpensive options for PA: ‘To buy the necessary equipment, a few sets of bands and balls, that isn’t a problem’ (Zelko et al., 2024).

##### TDF domain: beliefs about consequences

‘Beliefs about consequences’ was a prominent domain for facilitators, coded 19 times by 7/8 papers with data about staff perspectives. Two themes were defined.

Theme 1: PA could improve patient's quality of life

Staff and physicians held the belief that PA offered benefits for patients’ quality of life, through mental (Jhamb et al., 2016; Wodskou et al., 2021; Zelko et al., 2024) and physical benefits (Castillo et al., 2022; Jhamb et al., 2016; Painter et al., 2014; Zelko et al., 2024). Some of these benefits were clinical, such as muscle mass (Zelko et al., 2024), but particularly in staff data, more general benefits were described, such as improving their attitude and keeping patients moving and functional (Painter et al., 2014; Zelko et al., 2024). Staff in one study expressed that PA could improve the experience of dialysis by altering ‘patients’ perceptions of dialysis time’ while physician data suggested feeling better from IDE could make the treatment less burdensome (Zelko et al., 2024).

Theme 2: Patients engaging in PA could dialyse better and have higher functionality

Staff had thoughts that PA could enhance dialysis by managing water weight (Jhamb et al., 2016) and vascular access (Zelko et al., 2024). A quote from one study also predicted that long-term benefits of PA leading to increased functionality for patients could lessen demands on nurses: ‘Taking care of a dialysis patient when they’re more able to do things on their own means they’re a much easier patient to take care of’ (Painter et al., 2014).

### Relevant COM-B domains and intervention functions

**Figure 2.**
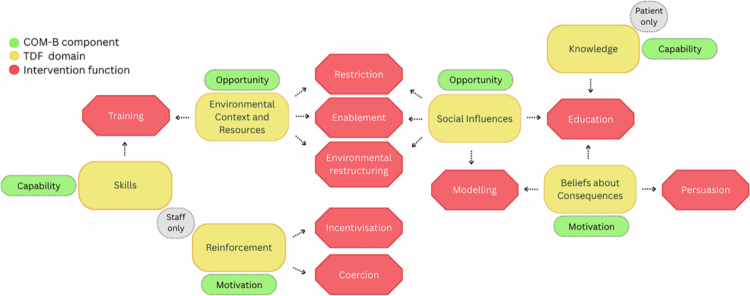
Prominent domains linked to COM-B components and intervention functions.

Due to the congruence between prominent domains for staff and patients, and similar prominent domains for barriers and facilitators, the implicated COM-B components also shared a focus (Figure 2). Both physical and social Opportunity components were implicated by staff and patient data, as well as reflective Motivation. All three components of the COM-B were implicated (Tables 2 and 3). The intervention functions associated with prominent domains cover all nine areas identified in the BCW: Education, Persuasion, Incentivisation, Coercion, Training, Restriction, Environmental restructuring, Modelling and Enablement.

### Sensitivity analysis

The frequency analysis for IDE only data was performed for patient and staff data (supplementary material S7). For patient data, there were minor changes in the ranking of prominent domains; however, the five highest-ranking domains remained unchanged. The prominent barrier domain ‘Skills’ swapped places with ‘Social influences’, which had originally ranked 4th. Similarly, among facilitator domains, 'Social influences' remained the most prominent. However, 'Beliefs about Consequences' and 'Reinforcement' were replaced by 'Knowledge' and 'Environmental Context and Resources', both of which had scored highly in the original analysis, and were ranked fourth and fifth, respectively, in the sensitivity analysis. Prominent domains for staff data remained consistent between the primary analysis and the sensitivity analysis.

Review of the thematic analysis showed that most themes identified in the primary analysis were retained. A small number of patient themes (one facilitator and three barrier themes) were no longer represented within the IDE-only subset, reflecting the reduced evidence base available for these themes. Importantly, no themes associated with the originally prominent patient domains were lost. For staff data, a greater number of themes were affected by restricting the analysis to IDE studies, particularly facilitator themes (7/16) compared with barrier themes (3/21). Of the themes associated with prominent domains, two facilitator themes (‘Benefits of IDE’ and ‘Patients engaging in PA could dialyse better and have higher functionality’) were no longer represented in the IDE-only subset. Overall, however, the pattern of findings remained broadly consistent with the primary analysis.

## Discussion

This updated qualitative evidence synthesis offers a theory-informed understanding of the behavioural determinants that shape engagement with physical activity (PA) among patients receiving haemodialysis, alongside the barriers and facilitators influencing healthcare professionals’ support for PA within dialysis settings.

By integrating the TDF and the COM-B model, and linking findings to candidate intervention functions from the BCW, this review moves beyond descriptive accounts of barriers and facilitators and provides a structured foundation for intervention development and implementation.

Patient findings were consistent with the original review (Li et al., 2021), previous quantitative studies (Clarke et al., 2019; Lightfoot et al., 2021; Mesa‐Gresa et al., 2024), and with the limited theory-guided work in this area (Clarke et al., 2019; Young et al., 2018). This review identified ‘Reinforcement’ as a prominent patient facilitator, which is a construct previously under-represented in haemodialysis despite being identified in other long-term conditions (Booth et al., 2024; Chee et al., 2025; Song et al., 2024), highlighting the role of positive experiences, perceived progress, and emotional reward in sustaining behaviour. Several other determinants identified here correspond to factors shown to influence actual PA behaviour in other chronic disease populations. The Social Cognitive Theory construct of self-efficacy has been shown to significantly associate with PA levels (McAuley et al., 2011; Patterson et al., 2014), supporting the relevance of prominent TDF domains identified in this review, like ‘Knowledge,’ ‘Skills’, and ‘Beliefs about Capabilities’. Similarly, analysis of survey data found that many benefits and barriers fall within the ‘Beliefs about Consequences’ domain, which significantly influence PA behaviour (Lightfoot et al., 2021). The presence of positive and negative beliefs about consequences coexisting highlights the non-linear and sometimes conflicting nature of behavioural determinants and the heterogeneity of this population. Such complexity suggests that intervention approaches should avoid assuming uniform responses to single strategies and instead adopt tailored, flexible designs that can accommodate differing and dynamic belief systems. In line with previous studies (Clarke et al., 2019; Li et al., 2021), patients reported a range of perceived benefits associated with PA. However, the inconsistent quality-of-life improvements observed in exercise trials (Anding-Rost et al., 2023; Graham-Brown et al., 2021; Greenwood et al., 2021) suggest that recognising the benefits of PA alone may not be sufficient to achieve meaningful improvement in patient outcomes. This highlights the importance of addressing the behavioural and contextual determinants that influence sustained engagement with PA.

Previous theory-based work (Clarke et al., 2019; Young et al., 2018) found similar barriers to staff facilitation of PA, such as a lack of knowledge and skills. This was identified in the current work and poses a significant barrier to introducing PA into routine practice. The skills requirement for staff also varies between programs: some models specify additional, exercise-specific staff (Bennett et al., 2026; Sheshadri et al., 2020; Young et al., 2018), whereas others are substantially nurse-led (Parker, 2016). The importance of staff knowledge is reinforced by findings in the wider CKD population, where inadequate understanding of the effects of PA and of prescribing exercise were the main reported barriers to exercise counselling for physicians who demonstrated low levels of exercise recommendation to their patients (Morishita et al., 2014). This updated review also identified barriers relating to low prioritisation of PA, consistent with evidence that perceptions of healthcare professionals, including the belief that PA is a lower priority than other medical issues, were significantly negatively correlated with patient PA levels (Regolisti et al., 2018).

Across both participant groups, there were clear areas of alignment. Patients and staff recognised potential benefits of PA, shared concerns about adverse events, and identified how social and environmental factors influence participation and delivery. This is distinct from some earlier work which demonstrated a disconnect between staff and patient perspectives (Wodskou et al., 2021; Young et al., 2015). The current findings imply that interventions may be more successful when designed to simultaneously target patient and staff determinants, given the reciprocal influence each exerts on the other’s beliefs, motivation, and opportunity. The knowledge, beliefs and behaviours of nurses appeared to greatly influence patients' own knowledge, beliefs about their capabilities and about likely outcomes of PA. Similarly, excerpts suggest that assumed reluctance from patients reduced staff motivation. Further research into how the perspectives of different groups influence each other would be fruitful and help identify key areas to target.

Despite substantial overlap between patient and staff perspectives, important differences were identified. Patients more frequently described symptom burden and physical limitations affecting PA engagement, whereas staff more often focused on organisational and safety considerations associated with supporting PA within dialysis services. While this may partly reflect the limited representation of exercise professionals within the staff samples, it suggests that effective interventions must address both individual and system-level determinants.

Findings from the IDE-only sensitivity analysis reinforce this interpretation. Patient themes remained largely unchanged, whereas several staff themes were no longer represented. Given the relatively small volume of staff data available from IDE studies, this likely reflects limitations of the evidence base rather than true differences in determinants. However, it does suggest that staff perspectives may be particularly sensitive to context, supporting the need for locally informed intervention development.

### A theory-informed intervention

The TDF identified behavioural determinants influencing PA participation and PA support within haemodialysis settings. By linking these determinants to COM-B and the Behaviour Change Wheel, we were able to identify candidate intervention functions and behaviour change techniques. The accompanying thematic analysis adds contextual insight into how these determinants are experienced in practice, allowing intervention developers to prioritise key barriers and facilitators and tailor intervention components accordingly. This provides a structured, theory-informed basis for intervention development.

While all nine intervention functions from the BCW were implicated by our data, the BCW recommends using strategic tools such as the APEASE criteria to assess suitability (Michie & Atkins, 2014). We provide a high-level overview of which functions meet the APEASE criteria in Table 5 and recommend that implementers focus on functions that meet the APEASE criteria. Below, we explore how themes in the prominent domains could be targeted through intervention functions and BCTs, prioritising functions judged appropriate by the APEASE criteria. Table 6 provides a detailed overview of the identified prominent domains, associated intervention functions, and example BCTs according to the Behaviour Change Technique Taxonomy v1 (Michie et al., 2011). Although outside the scope of intervention development, policy categories that could address these areas are also discussed.

**Table 5.** The nine candidate intervention functions, as identified through the BCW, were assessed for appropriateness using APEASE.

Candidate intervention function	Considerations for APEASE in the context of haemodialysis setting
Education	Appropriate; providing educational pamphlets or presentations on the benefits of PA for this population could increase willingness to engage in PA.
Persuasion	Existing staff not trained for this so not practical unless additional staff involved.
Incentivisation	Appropriate; providing social incentives such as certificates could encourage PA.
Coercion	Not acceptable in healthcare setting.
Training	Appropriate; staff training on recognising contraindications for PA in the haemodialysis centre could increase confidence in facilitating it.
Restriction	Not practical as restricting other activities to encourage PA would be unethical.
Environmental Restructuring	Potentially appropriate on a small scale; however, large-scale implementation may be limited by resources and lack of space within dialysis units
Modelling	Appropriate; observing peers or staff engaging in PA could positively influence behaviour.
Enablement	Appropriate; being provided with exercise equipment that is suitable for fitness level could encourage PA.

**Table 6.** Themes from prominent domains with potential candidate intervention functions and BCTs (with BCTTv1 codes) to complement the discussion section: ‘a theory-informed intervention’. Behaviour Change Techniques (BCTs) were coded according to the Behaviour Change Technique Taxonomy v1 (Michie & Atkins, 2014).

Prominent domain	Themes	Candidate intervention function	Examples from BCTTv1 (Michie & Atkins, 2014)
Skills (Capability)	Patients believe that they are too unwell for PA	Training	6.1 Demonstration of the behaviour, e.g. demonstrating how to do PA safely. 4.1 Instruction on how to perform the behaviour, e.g. providing instructions on how to engage in PA. 8.1 Behavioural practice/rehearsal, e.g. prompting patients to practice a specific exercise.
Knowledge (Capability)	Acutely unwell patients are perceived to be too unstable for PA Low prioritisation of PA by staff due to lack of understanding of value Staff perceive lack of education and knowledge for patients	Education	4.1 Instruction on how to perform the behaviour, e.g. providing instructions on how unstable patients can safely engage in PA. 5.1 Information about health consequences, e.g. benefits of exercising, safety for unstable patients. 5.6 Information about emotional consequences, e.g. explaining that PA is associated with improved mood. 7.1 Prompts/cues, e.g. clinical algorithms and exercise guides. 9.1 Credible source, e.g. leveraging trust by presenting information on the value of PA from clinicians to staff, or from nursing representatives to nurses.
Environmental Context and Resources (Opportunity)	Perceived environmental barriers, hazards and risks for patients and nurses Lack of priority for PA in dialysis environment Minimising disruption to current dialysis processes Benefits of IDE Financial viability Financial limitations	Environmental Restructuring	12.5 Adding objects to the environment, e.g. exercise equipment 7.1 Prompts/cues, e.g. adding visual cues to take part in a PA program. 12.1 Restructuring physical environment, e.g. simplifying processes involved in PA.
Social Influences (Opportunity)	Personal social support system Support from dialysis staff and community	Enablement	3.3 Social support (emotional), e.g. including patient family/carers in PA program but providing personal social support beyond of the dialysis community is outside the scope of an intervention. 3.1 Social support (unspecified), e.g. PA champions, peer competitions and challenges. 3.2 Social support (practical), e.g. practical counselling on how to engage with PA from staff.
		Environmental restructuring	7.8 Associative learning, e.g. adapting existing activities patients enjoy to include PA. 12.1 Restructuring the physical environment, e.g. creating an ‘exercise bay’ or ‘exercise area’ in the waiting room. 12.2 Restructuring the social environment, e.g. seating exercising patients together for their dialysis treatment. 12.5 Adding objects to the environment, e.g. posters which portray PA as a social activity
Beliefs about consequences (Motivation)	PA could improve patient quality of life Patients engaging in PA could dialyse better and have higher functionality Concerns and fear of adverse events Perceived lack of meaningful impact	Education	2.7 Feedback on outcome (s) of the behaviour, e.g. monitoring and providing feedback on improvements such as functionality tests and muscle mass. 5.1 Information about health consequences, e.g. educating about the benefits and safety of PA. 5.3 Information about social and environmental consequences, e.g. sharing case studies of PA building social connections. 5.6 Information about emotional consequences, e.g. providing information emphasising PA can improve mood.
		Modelling	6.1 Demonstration of the behaviour, e.g. by sharing best practice from units with successful PA programs.
Reinforcement (Motivation)	Perceived positive outcomes from doing PA	Environmental restructuring	7.1 Prompts/cues, e.g. establishing a specific time for engaging in PA within the dialysis. 12.5 Adding objects to the environment, e.g. adding visuals displaying PA goals or leaderboards.
		Incentivisation	2.2 Feedback on behaviour, e.g. providing feedback on number of completed PA sessions or progress towards engagement goal. 2.3 Self-monitoring of behaviour, e.g. providing progress charts for patients to fill in when they engage in PA. 2.6 Biofeedbacking, e.g. providing wearable technology to provide feedback on heart rate. 10.6 Non-specific incentive, e.g. certificate for completion of x IDE session.

#### Capability

The entry-level skill required to participate in a PA program should be within the capability of haemodialysis patients, and the importance of this has been reported previously (Wilkinson et al., 2025; Wilund et al., 2020). Although some IDE programs not designed for this setting have struggled to match patient skill levels (Hu et al., 2025), commonly used enablement options, such as intradialytic cycling or the use of resistance bands, easily allow for tailoring. Training through demonstrating exercise and giving feedback can further bridge the gap between the patients’ current physical capability and the capability needed to achieve guideline-recommended moderate to vigorous intensities (Baker et al., 2022) and has been successfully incorporated into many programs for this population (Graham-Brown et al., 2021; Greenwood et al., 2021; Ribeiro et al., 2023). Progressive training typically requires specialised staff (Gavanda et al., 2025); however, there are also resources and training available to support non-specialised staff in increasing patients’ physical function (Exercise in CKD Course | Grexercise, n.d.).

Education would tackle staff's lack of knowledge on the value of PA by providing information on health consequences, both the benefits of PA and that it is safe for most patients, and this could also increase patients' knowledge. However, increasing knowledge alone is unlikely to be sufficient to bring change, as this information is already included in guidelines (Baker et al., 2022), and a national survey showed very limited IDE and rehabilitation provision (Ancliffe et al., 2024). Analysis of a subsequent survey found that local policies were associated with the implementation of NICE guidance (Graham-Brown et al., 2025), and these are typically included when IDE is incorporated into routine practice (Forgeron & Valeriote, 2001; Martins et al., 2025). For non-specialised staff, prompts and cues such as integrating exercise guides and clinical algorithms to enable tailored exercise prescriptions and clinical safety reviews can increase staff knowledge of supporting PA with deconditioned patients (Bennett et al., 2022).

#### Opportunity

A well-designed IDE program increases opportunity, and typically patients do not report lack of opportunity as a barrier in settings where IDE is available (Heiwe & Tollin, 2012; Young et al., 2018). For staff, system-level integration and changes in workplace legislation that enable them to prioritise PA, as recommended in other clinical populations (Cunningham & O’Sullivan, 2021; Physical Activity, 2021), can increase the opportunities for them to support PA. In practice, this could include formalising PA support in job descriptions and quality processes. Concerns about staff hazards were primarily associated with earlier IDE bicycle models, which were notably large and heavy (Graham-Brown et al., 2016; Kontos et al., 2007). Newer designs, however, are considerably smaller and lighter (Martins et al., 2025), and as such, additionally address concerns about disruption, while the lower price can address financial barriers. Program using resistance bands (Segura-Ortí et al., 2009) can also tackle such issues, or an alternative intervention design outside the dialysis centre such as walking (Manfredini et al., 2022) subverts the need for equipment and the barriers that come with it. Similarly, by bringing in a PA program as part of the provision of care, the lack of dialysis-specific guidelines and adaptations is also addressed right away and does not need further examination or mapping. Providing visual cues and restructuring to simplify engagement have been shown to be helpful in engaging older adults to take part in physical outdoor activities (Badmos et al., 2025), and this would also be appropriate for the dialysis setting.

Tackling staff barriers was important to patients, who identified a lack of staff buy-in as a social barrier, and this has been a persistent challenge in previous work (Young et al., 2018). Staff support is strongly associated with greater patient participation in PA (Parker et al., 2021; Regolisti et al., 2018), highlighting the need for programs that staff find acceptable and for staff involvement in co-design (Suna et al., 2025). Leveraging social influences is a key mechanism for behaviour change, and this finding aligns with broader evidence (Far et al., 2015). Additional ways to enhance social opportunity for patients include using PA champions to model positive attitudes towards PA (NICE, 2019; Wen et al., 2024) or incorporating peer competitions and challenges with a social focus (Charles et al., 2020; Patel et al., 2021). Although environmental restructuring is often considered unfeasible due to limited space, in practice, it can involve minor changes to promote social participation, such as assigning patients participating in IDE to the same bay.

#### Motivation

PA programs for this population, particularly IDE programs, provide opportunities for patients to engage with and staff can facilitate. However, motivation ultimately determines their success, as evidenced by low uptake of IDE when it is available (Parker et al., 2021). Many staff determinants relate to beliefs about the consequences of PA, so education on the safety, benefits, and risks of non-participation is needed and should aim not just to inform but to motivate. Sharing success stories from units with established programs can model PA as routine care, and embedding PA into quality processes, such as audits, appraisals, and KPIs, could create meaningful consequences for failing to support PA.

Patients identified that positive reinforcement encouraged engagement in PA. Simple forms of environmental restructuring and incentivisation, such as visuals displaying PA goals at each dialysis station, leader boards for PA challenges, progress charts and certificates, could amplify this effect (Harris, 2019; Zhang et al., 2022). This work and others (Greenwood et al., 2021) highlighted the role of staff in motivating patients, indicating that staff training should include guidance on how to use reinforcement and encouragement to support consistent participation. Strengthening staff involvement in this way may also help address the high dropout rates reported in IDE programs (Martins et al., 2025; Parker et al., 2021).

The TDF, COM-B and BCW mapping identifies several candidate intervention functions (or components) most likely to be effective and a wide range of possible BCTs. Co-design with patients, staff, and decision makers and the use of existing evidence-based programs (Martins et al., 2025; Young et al., 2018) will be critical in tailoring intervention functions to local contexts, improving acceptability, and ensuring sustainability.

## Strengths and limitations

This review used a theory-informed synthesis approach, integrating TDF, COM-B and the BCW to understand behavioural determinants across diverse haemodialysis settings. Furthermore, by linking findings to candidate intervention functions through COM-B and BCW, guidance for future intervention development or program refinement is more immediately usable and relevant to the challenges of implementation. The current work combines perspectives on speculative and intervention-based PA but does not link reported barriers and facilitators to PA levels, nor does it factor the real-world impact of barriers or facilitators into the prominence ranking for domains. It is therefore crucial that the interpretation of these findings is complemented by quantitative and mixed-methods work, as some commonly reported barriers are not necessarily associated with lower levels of PA (Delgado & Johansen, 2012). The findings were intended to inform intervention development rather than produce graded recommendations; however, future work could apply frameworks such as CERQual to provide a more formal assessment of confidence in individual themes.

While using the TDF to understand barriers and facilitators to healthcare behaviours is becoming increasingly common, there remains little consensus on how the prominence of domains should be assessed. Some studies have relied primarily on coding frequency (Song et al., 2024), whereas others have considered additional factors such as level of elaboration and expressed importance (Bott et al., 2024).

To address limitations of frequency-only approaches, we incorporated both cross-study prevalence and coding frequency when identifying prominent domains. Domains identified as prominent were generally supported by both broader representation across studies and greater depth of supporting data, suggesting greater confidence in the consistency of these findings. However, prominence should not be interpreted as a formal measure of importance or confidence.

As with any frequency-informed approach, prominence rankings may be influenced by differences in reporting density across studies and by the subdivision of broad barriers and facilitators into multiple codes. Until greater methodological consensus is established, we recommend that prominence is interpreted as a heuristic for identifying commonly reported determinants rather than a mandate for prioritisation. Importantly, less frequently reported themes may still represent important barriers and facilitators within specific contexts and should not be overlooked during intervention development.

The boundaries between some TDF domains, particularly ‘Skills’ and ‘Beliefs about Capabilities’, are inherently overlapping, and different coding conventions may lead to alternative domain classifications. As mapping to COM-B components is dependent on TDF allocation, these decisions may influence the relative representation of capability and motivation within the synthesis.

In particular, classifying physical symptoms and limitations as ‘Skills’ emphasises physical capability as a key determinant, whereas alternative approaches may attribute these factors more strongly to beliefs about capability or motivation. As such, findings relating to the prominence of specific COM-B components should be interpreted with consideration of these conceptual choices.

The included studies covered an uneven split between participant groups. Carer data were coded; however, as only one paper included these perspectives, thematic analysis was not possible, limiting insight into their role in shaping physical activity behaviours. Although staff perspectives were included to support implementation, only eight papers included them, with a heterogeneous mix of roles disproportionately skewed towards nursing, and limited perspectives from gatekeeper roles, such as physicians and managers. Future research should prioritise the perspectives of gatekeepers, focusing on implementation outcomes such as acceptability, feasibility and sustainability, and ensure they are represented in the co-design process.

The IDE-specific sensitivity analysis demonstrated substantial consistency with the primary analysis. For patient data, the five highest-ranking domains remained unchanged, although there were minor changes in ordering. Although the smaller IDE staff dataset might be expected to produce greater variability, similar prominent domains were identified, suggesting broad consistency in staff-reported barriers and facilitators across contexts. Together, these findings support the transferability of the main analysis across IDE and non-IDE contexts, while suggesting that the relative prominence of some determinants may vary according to setting and mode of delivery.

Additionally, synthesising evidence from primary papers allows a breadth of information to be incorporated, but only a proportion of the original data is presented and analysed. Literature searches from inception may lead to an overemphasis on historical issues that have since been resolved, and barriers and facilitators will vary in different healthcare settings, geographies and cultures. As such, context-specific qualitative data to confirm the findings of this review before intervention development would be useful for exploring issues that may not have appeared in the literature and for examining how patients and staff currently view the targets identified in this work.

## Conclusion

Using the TDF and COM-B, this updated systematic analysis of qualitative studies identifies key behavioural areas to consider in the design and implementation of future interventions. Combined with the BCW, these findings provide a strong basis for future interventions to increase PA levels in this population.

## Supplementary Material

Supplementary_Materials_Overview_for_Systematic_Mapping_Review_V3.docx Supplemental Material

Supplemental Material Supplementary_Material_1.docx

Supplemental Material Supplementary_Material_2.docx

Supplemental Material Supplementary_Material_3.docx

Supplemental Material Supplementary_Material_4.docx

Supplemental Material Supplementary_Material_5.docx

Supplemental Material Supplementary_Material_6.docx

Supplemental Material Supplementary_Material_7.docx

## Data Availability

Data is available upon request.
