# Supplementary material for: Using behavioural theory to explore barriers and facilitators to physical activity in haemodialysis patients: an updated systematic review of qualitative evidence
Source: Health Psychol Behav Med. 2026 Jul 27;14(1):2707668. doi: 10.1080/21642850.2026.2707668 (PMC13410551; doi:10.1080/21642850.2026.2707668)
Supplement: Supplementary_Materials_Overview_for_Systematic_Mapping_Review_V3.docx — Supplemental Material [file RHPB_A_2707668_SM9123.docx]

1. Full search strategies
2. Codebook
3. Splitting and coding tables (patient, staff, physician and carer data)
4. Examples of ambiguous quotes and mapping decision
5. Risk of bias assessment (CASP checklist)
6. Full thematic analysis (patient and staff)
7. IDE only sensitivity analysis: codebook, frequency analysis and thematic analysis
8. Summary of prominent barriers and facilitator domains for patient data

| **Prominent Barrier Domains** | **Prominent Facilitator Domains** |
| --- | --- |
| Environmental context and resources | Social Influences |
| Beliefs about consequences | Reinforcement |
| Skills | Beliefs about consequences |

*Prominent barrier and facilitator domains for patient data*

1. Summary of prominent barrier and facilitator domains for staff data

| **Prominent Barrier Domains** | **Prominent Facilitator Domains** |
| --- | --- |
| Environmental context and resources | Social influences |
| Beliefs about consequences | Environmental context and resources |
| Knowledge | Beliefs about consequences |

*Prominent barrier and facilitator domains for staff data*
