# Supplementary material for: Using behavioural theory to explore barriers and facilitators to physical activity in haemodialysis patients: an updated systematic review of qualitative evidence
Source: Health Psychol Behav Med. 2026 Jul 27;14(1):2707668. doi: 10.1080/21642850.2026.2707668 (PMC13410551; doi:10.1080/21642850.2026.2707668)
Supplement: Supplemental Material — Supplementary_Material_1.docx [file RHPB_A_2707668_SM9122.docx]

1. Full search strategies

PubMed search strategy –adapted for other databases

Database Search terms

PubMed #1 (("Intradialytic"[All Fields] OR ("hemodialysis"[All Fields] OR "renal dialysis"[MeSH Terms] OR ("renal"[All Fields]

AND "dialysis"[All Fields]) OR "renal dialysis"[All Fields] OR "hemodialysis"[All Fields]) OR ("hemodialysis"[All

Fields] OR "renal dialysis"[MeSH Terms] OR ("renal"[All Fields] AND "dialysis"[All Fields]) OR "renal dialysis"[All

Fields] OR "hemodialysis"[All Fields]) OR ("renal dialysis"[MeSH Terms] OR ("renal"[All Fields] AND "dialysis"[All

Fields]) OR "renal dialysis"[All Fields]) OR ("renal dialysis"[MeSH Terms] OR ("renal"[All Fields] AND "dialysis"[All

Fields]) OR "renal dialysis"[All Fields] OR ("extracorporeal"[All Fields] AND "dialyses"[All Fields]) OR

"extracorporeal dialyses"[All Fields]) OR ("renal dialysis"[MeSH Terms] OR ("renal"[All Fields] AND "dialysis"[All

Fields]) OR "renal dialysis"[All Fields] OR ("extracorporeal"[All Fields] AND "dialysis"[All Fields]) OR "extracorporeal

dialysis"[All Fields]) OR ("renal dialysis"[MeSH Terms] OR ("renal"[All Fields] AND "dialysis"[All Fields]) OR "renal

dialysis"[All Fields] OR "dialysis"[All Fields] OR "dialysis"[MeSH Terms]) OR ("dialysis"[MeSH Terms] OR

"dialysis"[All Fields] OR "dialyses"[All Fields]))

#2 (("exercise"[MeSH Terms] OR "exercise"[All Fields]) OR (“intradialytic”[All Fields] AND ("exercise"[MeSH Terms]

OR "exercise"[All Fields])) OR ("physical therapy"[MeSH Terms] OR ("physical"[All Fields] AND "therapy"[All

Fields]) OR "physical training" All Fields]) OR ("physical training"[MeSH Terms] OR ("physical"[All Fields] AND

"training"[All Fields]) OR "physical activity"[All Fields] OR ("physical"[All Fields] AND "activity"[All Fields]) )

#3 (("qualitative study"[Publication Type] OR "qualitative study as topic"[MeSH Terms] OR "qualitative study"[All

Fields]) OR ("qualitative research"[MeSH Terms] OR ("qualitative research"[All Fields]) OR ("qualitative

description"[MeSH Terms] OR "qualitative description"[All Fields]) OR ("phenomenological study"[MeSH

Terms] OR " phenomenological study"[All Fields]) OR ("grounded theory"[MeSH Terms] OR "grounded

theory"[All Fields]) OR ("interview"[ MeSH Terms] OR "interview"[All Fields]) )

#4 #1 AND #2 AND #3
