# Supplementary material for: Using behavioural theory to explore barriers and facilitators to physical activity in haemodialysis patients: an updated systematic review of qualitative evidence
Source: Health Psychol Behav Med. 2026 Jul 27;14(1):2707668. doi: 10.1080/21642850.2026.2707668 (PMC13410551; doi:10.1080/21642850.2026.2707668)
Supplement: Supplemental Material — Supplementary_Material_2.docx [file RHPB_A_2707668_SM9119.docx]

# Codebook Papers Legend:

| **Number** | **Paper** |
| --- | --- |
| (1) | Song et al. (2019) |
| (2) | Kontos et al. (2007) |
| (3) | Sieverdes et al. (2015) |
| (4) | Thompson et al. (2016) |
| (5) | Sutherland et al. (2021) |
| (6) | Liu et al. (2020) |
| (7) | Heiwe and Tollin (2012) |
| (8) | Jhamb et al. (2016) |
| (9) | Painter et al. (2014) |
| (10) | Hu et al. (2024) |
| (11) | Zelko et al. (2024) |
| (12) | Wodskou et al. (2021) |
| (13) | Castillo et al. (2021) |
| (14) | Rothpletz-Pulgia, (2022) |
| (15) | Huang ,et al. (2023) |
| (16) | Sheshadri, et al. (2020) |
| (17a) | Young *et al*. (2015) (pre-implementation)* |
| (17b) | Young *et al*. (2015) (post-implementation)* |

Normal text denotes patients

*Italics denotes nurse/staff ***

**Bold denotes clinician****

***Bold and italics denotes carer***

*Study 17 includes pre and post implementation data and counted separately*.* The decision to count pre and post implementation separately was made because they describe two different contexts (before an intervention and after/during an intervention) and therefore capturing whether a barrier or facilitator occurred in both these contexts demonstrates prominence, which is the aim of the frequency analysis.

**Staff and clinician codes are counted separately for frequency analysis as they represent distinct viewpoints.

## Facilitators coded to domain

| **TDF Domain**  ***/COM-B construct*** | **Facilitators coded to domain** | **Justification/indications for coding** |
| --- | --- | --- |
| Knowledge  *Capability – psychological*  “An awareness of the existence of something” | Desire to acquire specific knowledge about exercise (1)(15) | Desire to gain knowledge |
|  | Knowledge about the benefits of physical activity (1)(5)(6)(7)(8)*(8)***(8)**(17a) | Having knowledge facilitates |
|  | Knowledge about the consequences of lack of physical activity (1)(2)(6)(7)(8)*(8)*(10)(12)(14) | Coded here when objective knowledge rather than belief about consequences for self |
|  | Education programme/package for patients *(2)* | To increase knowledge |
|  | Staff recognition of the benefits (general) of IDE *(4)* | Knowledge of benefits is facilitator, also coded to Beliefs about consequences |
|  | Needing staff to have practical knowledge of how to facilitate and support IDE (4)*(13)(17a)* | Anything around the specifics of IDE such as how to clean and maintain equipment, how to move safely |
|  | Knowing what to expect of IDE to address apprehension (10)(17a) | Knowledge of structure and what it entails empowers staff and patients |
|  | Patient education on benefits of exercise is key *(4)*(8)*(8)* | To increase knowledge |
|  | Offering a pilot trial of IDE (7)*(12)*(17a) | Trial provides knowledge of IDE |
|  | Staff knowing which patients are safe to take part in IDE and where IDE is contraindicated *(8)(12)(13)* | Knowledge of what is safe and not safe is facilitator |
|  | Demonstration of the exercises (5)(10)(12)(15) | Coded here because demonstration provides practical knowledge on how to perform exercise |
|  | Curiosity and interest (7)(10)(17a) | Coded here as desiring more knowledge |
|  | Shared understanding of how IDE is prioritised on dialysis unit *(4)* | When patients know that they need to wait for the equipment and are patient (because staff are busy) then staff are more likely to help |
|  | Awareness of different exercise options including those that suited for limited mobility patients (12) | Knowledge of different exercise options they can use in different situations |
|  | Needing staff to have sufficient knowledge to reassure and encourage patients *(17a)*(17a) | If staff are well informed, they can answer the concerns that patients have |
|  | Knowledge that a wide range of patients can/do take part in IDE *(4)(17b)* | Having this knowledge will support buy in from staff and patients |
| Skills  *Capability – psychological and physical*  “An ability or proficiency acquired through practice” | Exercise as part of routine care *(2)* | Coded here as suggests that staff do have skills to support if part of routine care. Also coded to Social/Professional and Intention |
| Social/Professional Role and Identity  *Motivation – reflective and automatic*  “A coherent set of behaviours and displayed personal qualities of an individual in a social of work setting” | Exercise as part of routine care *(2)* | Also coded to intentions; identity of staff is ‘to deliver routine care’ |
|  | Sense of community outside of dialysis (4)(5)(10) | Having something in common besides dialysis and sense of identity |
|  | Staff view exercise as team effort *(9)* | The team of staff see inclusion of exercise as all their roles |
|  | Guided by spiritual beliefs (6) | Religious identity motivates them to stay well |
|  | Connecting with previous identity as an active person (10) | Reminder of identity as someone who used to enjoy exercise |
|  | Desire to be productive member of society (10)(14)(15) | Coded here as being a productive member of society is part of identity |
|  | Ability to personalise activity to match personal preferences (10) | Coded here as the preferences linked to social/professional identity |
|  | Desire to not be seen as visibly sick (15) | Not wanting identity to be as sick person |
| Beliefs about capabilities  *Motivation – reflective*  “Acceptance of the truth, reality, or validity about an ability, talent or facility that a person can put to constrictive use” | Instructor providing increased confidence and capability (4) | Increases belief about capability, also coded to reinforcement |
|  | Confidence in ability to maintain activity (7) | Confidence that capable to maintain activity makes them more willing to start |
|  | Confidence in ability to perform activity (7)(8) | Having confidence the activity is within their capacity is a motivator |
|  | Seeing examples of dialysis patients doing exercise (15) | Seeing this increases belief in own capabilities |
|  | Staff feel able to motivate patients *(12)* | Beliefs about capabilities |
| Optimism  *Motivation – reflective and automatic*  “The confidence that things will happen for the best or that desired goals will be attained” | Positive attitude (2)(7) | Positive attitude = optimism |
| Beliefs about consequences  *Motivation – reflective*  “Acceptance of the truth, reality, or validity about outcomes of a behaviour in a given situation” | Perceived benefit to physical well-being (1)(2)(3)(5)(7)(8)*(8)(9)(11)***(11)**(12)*(12)*(13)(14)(15)(17a) | Where short term improvement to physical well-being is seen as a consequence of exercise |
|  | Perceived benefit to mental well-being (1)(2)(8)*(8)(11)***(11)***(12)*(15) | Where perceived benefit to mental well-being is seen as consequence of exercise. |
|  | Positive beliefs about physical activity (long-term benefits) (1)(6)*(8)*(10)*(11)* **(11)**(12)*(12)***(13))**(15) | Where long term improvement to physical well-being is seen as a consequence of exercise |
|  | Weight-loss (3)(15)(16b) | Weight-loss seen as consequence |
|  | Improved self-image (3) | Improved self-image seen as consequence |
|  | Coping with stress (3) | Improved ability to cope with stress seen as consequence |
|  | Staff recognition of the benefits (general) of IDE *(4)(11)* | Benefits are consequence of IDE, also coded to Knowledge |
|  | Maintain independence (5)*(9)(11)***(11)**(12)(14)(15) | Maintaining independence is consequence |
|  | Belief that IDE could enhance dialysis treatment *(8)* | Coded here as belief of enhancement of dialysis is a beneficial consequence |
|  | Functionally able patients are easier for staff to manage *(9)* | Coded here as perceived consequence of exercise is benefits to patients AND staff |
|  | IDE could improve experience of dialysis *(11)*(12)(17a)(17b)**(17b)** | Improved experience is believed to be consequence |
| Reinforcement  *Motivation – automatic*  “Increasing the probability of a response by arranging a dependent relationship, or contingency, between the response and a given stimulus” | Helping someone else is incentive to exercise (1)(14) | Where motivation is not to help themselves, but someone else and that increases exercise |
|  | Observing/feeling the benefits (1)(2)(4)(7)(8)*(9)*(10)*(12)(13)*(14)(15)(17b) *(17b)* | Where benefits are realised, coded to reinforcement as they reinforce future exercise. |
|  | Improvement in energy levels (1)*(12)*(13)(15) | Feeling benefit reinforces future exercise |
|  | Enjoyment (2)(4)(5)(8)(10) | Also coded to emotion |
|  | Encouragement from  staff (4)(8) | Some coded to Social, where reinforcement appeared to be the motivator, coded here too as receiving encouragement for exercise reinforces future exercise |
|  | Instructor providing increased confidence and capability  (4) | Feeling benefit reinforces future exercise, also coded to belief about capabilities |
|  | Previous good experience of rehab support (4)(5)*(9)* | both when patient has experience good past experience or staff witnessed improvement through exercise which reinforces for future exercise |
|  | IDE pleasant to pass the time on dialysis (7)(10)(12) | IDE -> pleasant effect, therefore coded here |
|  | Sense of pride and satisfaction from exercising (7)(15) | Pride and satisfaction reinforce future exercise, also coded to Emotion |
|  | Recognition of loss of physical fitness (8) | Coded here because drawbacks of not exercising are tangible and realised |
|  | Feeling of accomplishment (8)(10)(15) | Feeling of accomplishment reinforces future exercise |
|  | Feeling guilty if exercise is missed (8) | Coded here as not exercising is linked to feeling guilty |
|  | Staff observing patient enjoyment*(13)* | Seeing patient enjoyment reinforces staff to support. Also coded to social influences |
|  | Physical activity gives sense of purpose (14) | Sense of purpose reinforces interest in PA |
|  | Physical activity allows more water intake (15) | Exercising allows more water intake |
| Intentions  *Motivation – reflective*  “A conscious decision to perform a behaviour or a resolve to act in a certain way” | Willingness to engage (1)(2)(5)(7) | Demonstrates intention to take part |
|  | Exercise as part of routine care *(2)* | Also coded to social/professional and Skills |
|  | Already attempting exercise (6) | Demonstrates intention to do exercise |
|  | Patients taking responsibility for their own care *(4)* (7)(12)(15) | Coded here because taking responsibility is a sign of intention |
|  | IDE introduced as something active and pleasant while on dialysis (7) | Intention is to pass time with something active and pleasant |
|  | Feeling of taking an active role in their care (3)(7)(8)(14)(15) | Intention to take an active role and to try to improve, also coded to behavioural regulation |
| Goals  *Motivation – reflective*  “Mental representations of outcomes or end states that an individual wants to achieve” | Desire to live  (6)(12)(14)(15) | Goal is to continue living |
|  | Desire to gain/maintain independence (12)(14) | PA to reach goal |
|  | Desire not to decline (6)(12)(14)(15)(16b) | Goal to not decline |
|  | Desire to improve strength (7) | Goal to improve strength |
|  | Desire to regain previous ability (10)(14)(16b) | Goal is to regain previous ability |
|  | Patients having intrinsic goals (6)(8)*(8)*(10)(14)(15)(16b) (17a) | Where the goal comes from the patient |
|  | Patients having extrinsic goals *(8)***(8)**(10)(16b) | When the goal comes from an external source such as a incentivisation |
|  | Aiming to be healthy for transplant (12)**(13)**(15)(16b) | Goal to be healthy for transplant process and to get a transplant |
| Memory, Attention and Decision Processes  *Capability – psychological*  “The ability to retain information, focus selectively on aspects of the environment and choose between two or more alternatives” | *No facilitators coded* |  |
| Environmental Context and Resources  *Opportunity – physical*  “Any circumstance of a person’s situation or environment that discourages or encourages the development of skills and abilities, independence, social competence, and adaptive behaviour” | Group exercise ensures safety (1)(4)(10) | Exercising in a group environment assuages fears about safety which would otherwise be barriers |
|  | Access to gyms | Access to gym environment facilitates |
|  | Ongoing access to rehab equipment (10)*(12)* | Access to environments where rehab equipment is available. |
|  | Exercising alone (3) | Coded here as being alone is circumstance of environment that encourages exercise |
|  | User friendly IDE equipment for patients to use *(2)*(7)*(12)* | Coded here as if the equipment is user friendly it facilitates exercise and reduces staff requirement |
|  | IDE equipment that is easy to move and maintain *(2)(12)* | Coded here as equipment is resource |
|  | When patients can retrieve their own equipment and monitor themselves/low reliance on staff *(4)(8)*(12)*(12)(17b)* | Widened to include general management of exercise, also coded to knowledge. If equipment and environment are such that patients can retrieve them. |
|  | Supervision by healthcare professional (5)(7)(12)(13) | Staff supervision is a resource |
|  | IDE bikes available (5) | Available bikes are resource |
|  | Exercise at dialysis centre/unit (6)(7)*(8)* **(8)**(17a) | Exercising in the environment of dialysis centre is facilitator |
|  | Physical activity as part of job (6) | Coded here as circumstance that encourages physical activity |
|  | Integrating with daily tasks (6)(14)(15)(16b) | Also coded to behavioural regulation |
|  | Being able to regulate own workload (7) | Environment/resource allows self regulation (also coded to ‘behavioural regulation’) |
|  | Staff having time to support IDE *(9)(13)* | Coded here because the additional resource of time would allow staff to support |
|  | Additional, exercise specific staff *(9)(17a)*(17a) | Staff are resource |
|  | Active travel (3)(15) | Environment which permits/encourages transport as exercise facilitates it |
|  | Safety benefits of IDE **(8)** | Environment provides safety |
|  | Not taking up free time *(8)*(12)(17a) | Time is a resource |
|  | Being able to exercise at home (10) | Reduces barriers of needing to go to a different place such as gym or park which entail their own barriers. |
|  | Able to perform complementary activity as they wish in own time (10) | Where environment and resources allow them to perform similar or complementary activities in different settings, e.g. having weights or resistance bands to use at home |
|  | Low cost material resources **(11)***(11)* | Low cost requires less resource |
|  | IDE provided by non-nurse staff **(11)***(11)(12)(13)* | Allocation of financial resource |
|  | Exercise that can be done in bed *(12)* | Equipment and environment needs to be set up for this |
|  | Access to parks (15) | Park is environment |
| Social Influences  *Opportunity – social*  “Those interpersonal processes that can cause individuals to change their thoughts, feelings, or behaviours” | Social support from personal network (1)(3)(6)(8)(15) | Encouragement or support to exercise from interpersonal process supports activity. Predominantly from staff. |
|  | Support from care partner (15) | Social relationship |
|  | Group exercise ensures safety (1)(4)(10) | Coded to mean preference for exercising at dialysis centre either with staff or with peers |
|  | Recommendations of healthcare professionals (1)(4)*(4*)(8)(*8)(11)(12)*(13)(15) | Social influence of healthcare professionals |
|  | Importance of patient’s family (1)(3)(5)(6)(8)*(8)(9)* (10)**(11)**(14)(15)(16b) | Social influence of family |
|  | Patient encouragement from staff (4)(5)(7)(8*)(8)****(*8)***(9)*(12)*(12)*(13)(15)*(17a)* | encouragement/support, some also coded to reinforcement |
|  | The influence of personal values  about exercise (4) | When staff, family, friends are interested in exercise themselves they are more encouraging, and it is motivating |
|  | Camaraderie and normalcy in the Unit (4)(7)(8)*(8****)*(8)(12)** | Social influences of others in the unit |
|  | Fostering a positive common identity (4)(7)(8)*(8)* | Also coded to Social/Professional Role and Identity |
|  | Tailored physical activity (by healthcare professional) suitable for dialysis patients (4) (5)(10)**(11)***(11)(12)***(13)**(15)(17a)*(17a)* | Relationship with health professional is key |
|  | Positive sense of competition between patients (4)(10)(12)*(12)* | When competing or comparing with each other encourages activity due to social connection |
|  | Encouragement from patient peers (4)(8)(10) *(8****)(11)****(12)*(13)(17a) | Encouragement from peers is social influence |
|  | Inspiration from peers (13)*(13)*(15)(17a) | Social influence |
|  | Physician involvement (13)*(13)*(17a) | Physician involvement influences staff and patients through social influence |
|  | Exercise champions (13)*(13)(17a)* | To encourage patients to exercise though social interaction |
|  | Staff observing patient enjoyment *(13)* | Enjoyment from patients socially influences nurses and staff to support exercise. Also coded to reinforcement |
|  | Seeing inactive peers decline (15) | The social relationship is responsible |
|  | Collaboration between staff team to support IDE *(17b)* | The social impact of seeing other members of staff supporting IDE encourages staff |
|  | Active involvement and leadership of senior staff *(17b)* |  |
| Emotion  *Motivation – automatic*  “A complex reaction pattern, involving experiential, behavioural and physiological elements by which the individual attempts to deal with a personally significant matter or event” | Enjoyment (2)(4)(5)(8)(10)(15) | Feeling of enjoyment is facilitator, also coded to reinforcement |
|  | Sense of pride and satisfaction from exercising (7)(15) | Also coded to reinforcement, emotions of pride and satisfaction |
|  | Distraction from treatment (7)(8)*(8)*(15) | Coded here as desire to avoid feeling of boredom |
| Behavioural Regulation  *Capability – psychological*  “Anything aimed at managing or changing objectively observed or measured actions” | Change in medical culture *(2)* | Change to include monitoring exercise |
|  | Self-monitoring (specifically with technology) (10) | Self-monitoring allows regulation |
|  | Forming a routine (6)(15) | Regulating by forming a routine |
|  | Integrating with daily tasks (6)(14)(15)(16) | Also coded to environment |
|  | Feeling of taking an active role in their care (3)(7)(8)(14)(15) | Coded here as allows patient to regulate own exercise, also coded to Intentions |
|  | Choice within activity (4)(8)(10) | Coded here because reflects choice and ability to self-regulate, could be anything that allows choice such as different activities or different options within activity |
|  | Being able to regulate own workload (7)(10)(15) | Ability to regulate own workload (autonomy) was facilitator. Some also coded to Environmental |
|  | Choice of when to exercise during dialysis (7) | Choice allows self-regulation |

## Barriers coded to domain

| **TDF Domain**  ***/COM-B construct*** | **Barriers coded to domain** | **Justification/indications for coding** |
| --- | --- | --- |
| Knowledge  *Capability – psychological*  “An awareness of the existence of something” | Lack of information sources for patients and staff (1)*(2)(9)(11)* | Including knowledge that sources exist. Grouped as information for patients will also educate staff. |
|  | Misconceptions about the relationship between physical activity and condition(s) (1)(5)*(9)*(15) | False knowledge, lack of correct knowledge |
|  | Staff lack of knowledge of the benefits of physical activity *(2)(8)* | Where staff don’t have knowledge of benefits |
|  | Staff not knowing what is right to instruct *(4)(9)(13)* | Lacking knowledge of what is correct |
|  | Patients lack of knowledge of the benefits (5)(8) (15) | Where patients don’t have knowledge of benefits |
|  | Lack of enough information support for patients (1)*(2)*(10)(15) | When the omission of essential information is a barrier e.g. what is moderate exercise? |
|  | Staff lacking of knowledge of guidelines *(9)* | Need knowledge that guidelines exist |
|  | Lack of guidance/structure for patients *(9)*(10)**(11)***(11)***(13)***(13)* | Coded here because patients are not provided knowledge of how to exercise. Absorbed initial barrier ‘Not sure of what to do’ |
|  | Not knowing what to expect from exercise (7)(10)(17a) | Lack of knowledge leading to apprehension |
|  | Lack of information for caregivers and families  **(11)***(11)* | Information to increase knowledge |
|  | Not knowing which patients are eligible **(13)**(13)(17a) | Knowledge about eligibility |
|  | Lack of practical knowledge to support IDE (13) *(17a)* | e.g. where are the bikes Staff lack of knowledge of IDE discourages patients |
| Skills  *Capability – psychological and physical*  “An ability or proficiency acquired through practice” | Lack of physical ability (1)(2)(3)(5)(16b) | Coded here where the real rather than perceived lack appeared to stop them from participating |
|  | Poor physical condition (1)(6)(7)(8)*(8)***(8)***(9)*(10)**(11)***(11)*(12)*(12)*(13)(14)(15)(16b) | General poor physical health |
|  | Fatigue (2)***(2)(***3)(5)(8)*(8)***(8)***(9)*(12)*(12)*(14)(15)(16b) | Fatigue affects ability to perform exercise |
|  | Other health conditions (1)(2)(6)*(12)*(14)(15)(16b) | Specific other health conditions |
|  | Pain (general) (2)(6)(7)(16b) | Pain leads to diminished skills, also coded to Emotion |
|  | Poor mental health (2)*(8)(9)****(11)****(11)* | Coded here because not mentally capable of overcoming barriers to exercise, also coded to belief about capabilities |
|  | Exercise could be risky for unstable patients **(11)** | No physically capable of undergoing exercise as unstable |
| Social/Professional Role and Identity  *Motivation – reflective and automatic*  “A coherent set of behaviours and displayed personal qualities of an individual in a social of work setting” | Cultural beliefs about physical activity (1) | Coded here because culture is part of social role |
|  | Exercise monitoring/assessment/provision not included in care provision/ not the nurses’ role  *(2)(4)(8)(9)*(11)*(12)(13)*(17a)*(17a)* | Barrier for staff to support exercise as not seen as their role, includes seeing these tasks as others’ role e.g. physician |
|  | Identity as a sick person *(9)* | Being a ‘sick person’ becomes part of social identity |
|  | Desire to be a ‘good’ patient (4) | Coded here when desire to be ‘good’ is barrier to asking to exercise/asking for support |
|  | Staff don’t see patients as interested in exercise *(17a)* | Staff perception of patient identity is barrier |
| Beliefs about capabilities  *Motivation – reflective*  “Acceptance of the truth, reality, or validity about an ability, talent or facility that a person can put to constrictive use” | Patient belief that condition(s) preclude physical activity (1)*(4)*(16b) | When intrinsic factor related to condition stops the patient from exercising (belief rather than actual but may still be true) |
|  | Staff belief that patients are not capable of IDE (17a) | When intrinsic factor related to condition means staff belief patient cannot exercise (belief rather than actual but may still be true) |
|  | Belief that age limits capacity(2)(13)(15) | Belief that age limits capacity is barrier |
|  | Staff doubt in their capacity to assist with IDE *(4)(8)(17a)(17b)* | Staff beliefs about limited capacity is barrier |
|  | Lack of confidence in ability to perform activity (8) | Coded here where lack of belief in physical ability is the barrier rather than lack of actual skill, some also coded to skill |
|  | Poor mental health (2)*(8)(9)****(11)****(11)* | Coded here because not mentally capable of overcoming barriers to exercise, also coded and skills |
|  | New patients overwhelmed by physical therapy requirement **(11)***(11)* | New patients not capable of managing any additional requirements beyond HD |
|  | Additional source of pressure for patients (13) | Patients not capable of managing additional need |
|  | Detachment due to dialysis **(11)** | Feeling detached coded as emotion |
| Optimism  *Motivation – reflective and automatic*  “The confidence that things will happen for the best or that desired goals will be attained” | Patient belief that no improvement is possible *(9)*(15)(16b) | Lack of optimism |
| Beliefs about consequences  *Motivation – reflective*  “Acceptance of the truth, reality, or validity about outcomes of a behaviour in a given situation” | Concern injury and accident (1)(8)*(8)***(11)** *(11)*(13)*(13)***(13)**(15)(16(17a) | Accident and injury could be consequence |
|  | Concern exercise may lead to sickness (1)(15) | Sweating and/or then getting ill are beliefs of consequence |
|  | Lack of perceived benefit (2)(5) | Do not believe benefit would be consequence of activity |
|  | Fear of losing transport benefit makes exercise lower priority *(2)* | Consequence of missing transport deters from physical activity. Also coded to environment. |
|  | Reduced access to patients in emergency if IDE equipment in the way *(2)*(17a) | Belief that IDE equipment will slow emergency response |
|  | Nurses fear injury to self from IDE equipment  *(2)* | Coded here rather than emotion because it is worry of consequence rather than emotion of fear that is affecting behaviour |
|  | Belief exercise is not necessary for long-term health goals (3) | Belief that without exercise goals can still be achieved |
|  | Asking for help with IDE could result in less help for more important things (4) | Belief that asking for help could lead to consequence of reduced help overall |
|  | Fear of fistula damage *(2)*(2)(5)(7)(8)**(11)***(11)*(15)(17a) | Belief that exercise could damage fistula |
|  | Fear of pain (5)(7)*(8)*(10) | When fear of experiencing pain prevents exercise |
|  | Fear of falling (5)(8)**(8)(11)***(11)*(15)(16b) | Coded here because the fear of consequence that is barrier, rather than the emotion of fear stopping them |
|  | General fears about safety *(2)*(5)(8)**(8)(11)** *(11)(12)*(17a) | Belief that decreased safety could be consequence of activity |
|  | Fear of further fatigue (7)(10)(15) | Coded here rather than emotion as it is belief, not fear that influences |
|  | Patients fear being a burden to staff (7)(17a) | Belief that asking for help with exercise could burden staff |
|  | IDE could disrupt dialysis *(8)***(8)***(11)*(12)(17a) | Disruption of dialysis is consequence of concern |
|  | Movement from IDE can set off dialysis alarm (12) | Consequence of concern is the alarm, also coded to reinforcement where it has already happened. |
|  | Doubts about benefits (13)*(13)***(13)(17***a)* | Lacking belief of positive consequences |
|  | Belief that IDE would be unsuccessful *(17a)* | Belief that even if it was established, IDE would be unsuccessful |
| Reinforcement  *Motivation – automatic*  “Increasing the probability of a response by arranging a dependent relationship, or contingency, between the response and a given stimulus” | Pain during exercise (3)(7)(15) | Feeling pain in exercise previously reinforces dislike of exercise |
|  | Fatigue after exercise (10) | Where exercise causes fatigue that becomes a barrier to future exercise |
|  | Lack of perceived benefit (2) | Not feeling benefit previously after exercise decreases likelihood of exercising |
|  | Previous negative exercise experience influences perception of IDE (7)*(8)* | Absorbed ‘submaximal test experienced’ as defined by Heiwe & Tollin |
|  | Movement from IDE could set off dialysis alarm (12) | Also coded to belief about consequences, coded here because when it happens in the past it deters from future attempts |
|  | Unpleasant physical reactions to exercise (15)(16b) | Such as sweating or breathlessness, experiencing these reinforces dislike for exercise |
|  | Previous injury (15)(16b) | Previous injuries make them more cautious and reluctant to do PA |
| Intentions  *Motivation – reflective*  “A conscious decision to perform a behaviour or a resolve to act in a certain way” | Lack of motivation (1)(3)(5)(6) (8)*(8)***(8)***(9)***(11)***(11)*(12)(13)**(13)***(13)*(16) | Coded here as equivalent to lack of intention. In some places it is described as a consequence of dialysis. |
|  | Preference is not physical activity (1) | Preference for inactivity equivalent to lack of intention |
|  | Lack of interest (5)(12) | Lack of intention to take part |
|  | Preference to rest during dialysis (rather than IDE) *(12)* | Coded here as intention is to rest rather than exercise |
| Goals  *Motivation – reflective*  “Mental representations of outcomes or end states that an individual wants to achieve” | Patient belief that physical activity is optional (3)**(11)***(11)* | Belief that they can reach goals without physical activity |
|  | Not necessary for long-term health goals (3) | Belief that they can reach goal without physical activity |
|  | Goals unattainable so no point trying (16b) |  |
| Memory, Attention and Decision Processes  *Capability – psychological*  “The ability to retain information, focus selectively on aspects of the environment and choose between two or more alternatives” |  |  |
| Environmental Context and Resources  *Opportunity – physical*  “Any circumstance of a person’s situation or environment that discourages or encourages the development of skills and abilities, independence, social competence, and adaptive behaviour” | Existing resources do not include physical activity/exercise guidance (1)*(9)* | Coded here as limitation of resource |
|  | Bad weather (1)(6)(8)(15)(16b) | Coded here as weather affects environment |
|  | Cost of exercise facilities/equipment  (1)*(9)* **(11)***(11)* | The cost of resources is limitation |
|  | Some facilities pose high risk of injury (1) | Where environment is judged to risk safety this is a barrier. |
|  | Facilities too crowded (1)(8)*(8)* | When environment is crowded this is a barrier |
|  | Exercise not a priority for nurses *(2)(4)(12)(13)(17a)*(17a) | Coded here as the environment of a dialysis ward means that supporting exercise is not the nurses’ highest priority |
|  | Fear of losing transport benefit makes exercise lower priority *(2)* | Coded here as exercise environment is a location requiring transport to and from. Also coded to beliefs about consequences |
|  | Patient lack of time due to dialysis (2)(5)(7)(8)*(8)* | Time is limited resource |
|  | Nurses lack of time due to other tasks *(2)(4)(9)***(11)***(11)*(12)*(13)(17a)*(17a) | When nurses’ lack of time is a barrier to PA |
|  | IDE equipment not suitable for everyone (2)*(8)*(10) | Changed from IDE bikes to make broader |
|  | Reduced access to patients in emergency if IDE equipment in the way *(2)(12)(13)* | Also coded to consequence, constraints of space in dialysis environment is limitation |
|  | Manual handling challenges for staff *(2)***(13)** | Coded here because heavy IDE equipment is environmental factor |
|  | Exercising in the dialysis centre (3) | Desire to not exercise in the dialysis unit |
|  | IDE as an additional source of pressure *(4)(*8)*(8)***(8)***(17a)*(17a) | Coded here as barrier is a result of exercising in the dialysis unit environment |
|  | IDE could disrupt the routine on the dialysis unit (1)(4)(7)**(11)***(11)*(12)*(12)(13)* | When any aspect of IDE is disrupting the staff/unit routine. Focus is more on the environment so coded here not consequences. |
|  | Local environment risks (6)*(12)*(16b) | Environmental barriers |
|  | IDE bikes not stable (7) | Coded here because IDE equipment is environmental factor |
|  | Lack of resources (8)*(8)***(8)** | Such as equipment and storage facilities |
|  | Fistula/catheter prevents activity (8) | Where extrinsic circumstance of condition such as fistula/catheter prevents participation in desired activity |
|  | Physical limitation during dialysis prevents IDE(8) | Where dialysis medical set up e.g. chair/line limits movement |
|  | Lack of suitable exercise options (general) (8) | Where the reason for lack of sutiablity is not specified |
|  | Lack of privacy in IDE (5)(8)*(8)***(11)***(11)(12)* | Environment does not provide privacy |
|  | Expectation for patient to access benefits and not work *(9)* | Financial benefit encourages less activity |
|  | No appropriate route for patients to request assistance with IDE (4) | Environment does not provide alternative methods to request assistance |
|  | Dialysis environment restricts conversation/sense of community (7) | Issue is environment |
|  | Instructor lack of knowledge of condition (1) | No exercise provision in environment for these types of patients |
|  | Lack of in-centre options (5) | Coded here as there aren't options there so they think they can't do anything |
|  | Hard to use equipment (10)(16) | Environment/resource factor. Focus on equipment being difficult/fiddly/technical rather than patient not having skills. Not IDE |
|  | Cost of dedicated staff *(11)* | Cost is resource |
|  | Where IDE has specific logistical needs *(13)* | Resources to do IDE create barrier |
|  | Lack of time reducing effective delivery of IDE *(17a)*(17a) | Lack of time leading to lack of encouragement and supervision, time is a limited resource |
|  | Staff rely on patients to self-refer to take part in exercise *(13)*(13) | Self-referral is an environmental factor |
|  | Exercise options in local gyms are unsuitable for dialysis patients (1)(15) | Coded here as not physically skilled enough for gym exercises |
| Social Influences  *Opportunity – social*  “Those interpersonal processes that can cause individuals to change their thoughts, feelings, or behaviours” | Friends/acquaintances not as understanding as family/spouse (1) | Lack of social support from wider circle is barrier |
|  | Lack of support from social network (1)(3)*(11)* | Social network is social influence |
|  | Lack of support from health professionals (4)(7)(8)**(12)** | Health professional is social connection |
|  | Feeling excluded from social exercise opportunities with those who do not require dialysis (1)(15) | Feeling left out from exercise that social contacts do makes them less likely to exercise |
|  | Peers’ information/advice untrustworthy and confusing (1) | Coded here as informal information is social issue |
|  | Lack of guidance from healthcare professionals(1)*(2)*(15) | Coded here as it is the fact that the healthcare professionals with whom they have a relationship do not provide guidance |
|  | Family/friends lack of knowledge of condition(s) (1)(15) | Coded here not knowledge as it is lack of knowledge as a social norm that creates the barrier |
|  | Carers not included in care plan | Coded here because the social relationship is not being used to its fullest potential |
|  | Family concern of ability (1)*(9)*(14) | When family perceive patient to be incapable of exercise and at risk of injury, coded here as social issue |
|  | Direct guidance not to exercise from family (1)(15)(16b) | Social issue |
|  | Friends/Family lack of physical activity (3) | Coded here as not exercising is social norm |
|  | A lack of support from  management *(4)(11)* | Coded here as interpersonal issue |
|  | Inconsistent help from dialysis staff for IDE (4)(17a) | Coded here as inconsistent help displays attitude of another person and therefore social issue |
|  | Staff appear too busy to help with IDE (1)(4) | Not wanting to disturb busy staff is social/interpersonal issue |
|  | Lack of ‘buy in’ from staff (4)(12)*(13)* | Coded here as attitude of staff is social influence on patients |
|  | Excessive support from staff encourages dependency *(9)* | Where excessive support from staff leads to decreased independence |
|  | Dependence on others to exercise | Where not being able to exercise without facilitation from staff/family/friends/carer is a barrier |
|  | Negative comments about IDE from patients (17a) | Hearing a negative comment from another patient discourages patients from wanting to take part |
| Emotion  *Motivation – automatic*  “A complex reaction pattern, involving experiential, behavioural and physiological elements by which the individual attempts to deal with a personally significant matter or event” | Pain (general) (2)(6)(7) | Where the feeling of pain reduces interest in exercise, also coded to Skills |
|  | Apprehension towards exercise (10) | Feeling of apprehension is a barrier |
|  | Lack of enjoyment/monotonous nature of PA/disliking exercising/finding it boring  (7)(8)(10)(14) | Coded here as exercise elicits feeling of boredom so avoided |
|  | Frustration of failure (10) | Frustration is emotion |
| Behavioural Regulation  *Capability – psychological*  “Anything aimed at managing or changing objectively observed or measured actions” | Used to being sedentary (1)*(9)***(11)***(11)* | Coded here as sedentary is the formed routine |
