# Supplementary material for: Using behavioural theory to explore barriers and facilitators to physical activity in haemodialysis patients: an updated systematic review of qualitative evidence
Source: Health Psychol Behav Med. 2026 Jul 27;14(1):2707668. doi: 10.1080/21642850.2026.2707668 (PMC13410551; doi:10.1080/21642850.2026.2707668)
Supplement: Supplemental Material — Supplementary_Material_3.docx [file RHPB_A_2707668_SM9118.docx]

## Supplementary Material 4

## Splitting of existing themes - Patient data

**Data analysed from written text not just quotations**

## Facilitators

Where barriers are found in themes categorised as facilitators, they are highlighted in grey.

| Original facilitator  /Study | Data | | | New facilitators (after splitting) | | TDF domain mapped to |
| --- | --- | --- | --- | --- | --- | --- |
| 1. Song et al. (2019) | | | | | | |
| Group exercise ‘beneficial’  Song et al. (2019) | “My exercise habit was forced to develop by my wife, because she has  been accompanying me to easy walk since the beginning of my disease”, “Exercise is boring, but it will be fun if I can talk with someone to kill time”, | | | Social support from personal network | | Social influences |
|  |  |  |  | The importance of patient’s family | |  |
|  | “ Additionally, when they perceived their role as a company for others, they were motivated to exercise more frequently. For example, working out with elderly parents or helping their spouses to lose weight were described as motivations and incentives to become active | | | Helping someone else is incentive to exercise | | Reinforcement |
|  | Patients also described group exercise as an essential way of guaranteeing safety in case an emergency arose related to their poor physical conditions, | | | Group exercise ensures safety | | Social influences  Environmental context & resources |
|  | My neighbour didn’t ask me out to exercise sometimes due to my slow walking pace”, “my friends took me to do square dance, but I couldn’t get catch up with them, and  then they never ask me out any more”, | | | Lack of physical ability (*new barrier*) | | Skills |
|  |  |  |  | Friends/acquaintances not as understanding as family/spouse (*new barrier*) | | Social influences |
|  |  |  |  | Lack of support from social network (*new barrier*) | | Social influences |
|  |  |  |  | Feeling excluded from social exercise opportunities with those who do not require dialysis (*new barrier*) | | Social influences |
|  |  | | |  | |  |
| Perceived benefits of exercise – ‘physical well-being’  Song et al. (2019) | “Exercise can prevent muscular atrophy. In addition, it also can stimulate body function via exercise”, *“*Exercise can improve physical well-being and strengthen the immune system to prevent complications*”,* Their perceived benefits of exercise mainly landed on improving physical well-being and making them “feel leading a healthier lifestyle” and full of energy. Similarly, exercise was perceived as a critical  means for stimulating blood circulation, strengthening cardiac functioning, and controlling blood pressure as well. In addition, some patients regarded exercise as an effective way to control weight, improve the quality of sleep, and remove toxins through sweating. | | | Perceived benefit to physical well-being | | Beliefs about consequences |
|  |  |  |  | Positive beliefs about physical activity (long-term benefits) | |  |
|  | Some patients summarized the benefits of their exercise experience. For example, they said that long-term exercise  improved their immune system, thus keeping them from developing other diseases. It also decreased the risk of having stiff joints. | | | Observing/feeling the benefits | | Reinforcement |
| Perceived benefits of exercise – ‘mental well-being’  Song et al. (2019) | “Exercise improves my mood, which makes me feel young, just feel like I go back to my 20s . . .I feel energetic when exercising” | | | Perceived benefit to mental well-being | | Beliefs about consequences |
|  |  |  |  | Improvement in energy levels | | Reinforcement |
| Attitudes to exercise information – ‘Positive attitude’ | Patients said that they would like to gain a thorough understanding of exercise with specific focus on the types of suitable exercise, how exercise can improve health conditions, and the consequences of lack of exercise. Annual lectures on kidney disease  offered by medical staff for all the patients in the unit addressed knowledge related to information that mainly focus on considerations involving dialysis session. However, patients in the study reported that they were keen to obtain more information at these lectures about “how to exercise” (Male, age 50) and “how to eat better and properly”  (Female, age 62). “Which types of exercise is suitable for me? I would like to try it if it suits me’, “I really wanna know about what the benefits of doing exercise are” | | | Desire to acquire specific knowledge about exercise | | Knowledge |
|  |  |  |  | Positive attitude | |  |
|  | “The motivation of exercise for me comes from internet. It says that patients undergoing HD definitely require exercise except for strenuous”, “I would like to know about exercise by myself, maybe from a friend who has been doing yoga” | | | Knowledge about the benefits of physical activity | | Knowledge |
|  | Patients said that they would like to gain a thorough understanding of exercise with specific focus on the types of suitable exercise, how exercise can improve health conditions, and the consequences of lack of exercise | | | Knowledge about the consequences of lack of physical activity | | Knowledge |
| Attitudes to exercise information – ‘Potential information sources’  Song et al. (2019) | “The motivation of exercise for me comes from internet. It says that patients undergoing HD definitely require exercise except for strenuous”, “I would like to know about exercise by myself, maybe from a friend who has been doing yoga”  Patients preferred to receive their information about exercise from doctors or other health care professionals rather than from their peers with similar medical conditions. They viewed peers’ comments as untrustworthy and confusing. “I definitely want to know about exercise. Doctors and nurses should give us some suggestions in terms of exercise, for example, how to exercise” | | | Recommendations of healthcare professionals | | Social influences |
|  |  |  |  | Willingness to engage | | Intentions |
|  |  |  |  | Peers’ information/advice untrustworthy and confusing (*new barrier*) | | Social influences |
|  |  |  |  | Lack of guidance from healthcare professionals | | Social influences |
|  |  |  |  | Existing resources do not include physical activity/exercise guidance (*new barrier*) | | Environmental context and resources |
|  |  |  |  | Lack of information sources for patients and staff | | Knowledge |
|  |  |  |  | Not sure of what to do (*new barrier*) | | Knowledge |
| 2. Kontos et al. (2007) | | | | | | |
| Sources of motivation: desire and actual  improvement  Kontos et al. (2007) | ‘‘I enjoy exercising. I was always active. So the desire is still there and that’s what pulls me through the days that are a little tougher because I’m not so well.’’, For many patients,  actual improvement from exercise gives them the motivation to adhere to exercise activities. As one patient described: ‘‘Keeps the blood pressure down, that’s the big thing. That’s why I exercise. And that’s what motivates me, otherwise I have to take the blood pressure pill everyday, but now my blood pressure seems to be holding up alright.’’, Patients also spoke of psychological improvements they experienced from exercise. This is captured in the following statement of a patient: ‘‘It does help. It helps you mentally. You feel better because you have exercised, you feel tired physically but mentally and emotionally I think the exercise helps.’’ | | | Enjoyment | | Emotion, Reinforcement |
|  |  |  |  | Positive attitude | | Optimism |
|  |  |  |  | Perceived benefit to physical well-being | | Beliefs about consequences |
|  |  |  |  | Observing/feeling the benefits | | Reinforcement |
|  |  |  |  | Willingness to engage | | Intentions |
|  |  |  |  | Perceived benefit to mental well-being | | Beliefs about consequences |
| 4. Thompson *et al.* (2016) | | | | | | |
| Social Interaction | Many participants described enhanced social interactions with other IDE participants. Several of the men discussed instances when they were competing with other trial participants. These interactions were perceived as positive and promoted a sense of camaraderie and normalcy within the unit (Q40 and Q41). | | | Camaraderie and normalcy in the Unit | | Social Influences |
|  | “Yeah, it’s positive. And especially guys, guys enjoy that. If you’ve been around guys, sports guys and things like that, that’s the thing to do. And it makes the dialysis environment a lot more pleasant…There’s more excuse now to yell across the room.” | | | Enjoyment | |  |
|  |  |  |  |  |  | Reinforcement /Emotion |
|  | “I’ll raise the bar. Maybe somebody else will want to—when I was cycling the other day there, my neighbor, he said, ‘Maybe I should have a race with you.’ I says, ‘Well, bring it on, bring it on.’” Q41 | | | Positive sense of competition between patients | | Social Influences |
|  | Another participant explained that IDE fostered a more positive common identity (Q43). “Like, we’re really, really close, we’re kind of like a little family, and we’re all down—like, we all meet downstairs...they would say things as, ‘Oh’—they liked it [IDE], they really looked forward to it, they looked forward to it when they come here. One of them down there, he—I asked him if he was going to continue once the program was done, and—but I just found him to be a little—I thought he was maybe a little older, a little tired, but no, he was—he says he notices how even his spirits—and even when we go downstairs, like, he’s just all chirpy and happy about it.” | | | Fostering a positive new common identity with other dialysis patients | | Social Influences |
|  | “Like, you can ask us dialysis patients when we’re sitting waiting around for each other or when we’re dialyzing beside one another, it’s just something—another exciting thing that, yes, we have dialysis in common, but now this is a positive thing we have in common that we can talk to each other about and encourage each other with.” | | | Sense of community outside of dialysis | | Social/Professional Role and Identity |
|  |  |  |  | Encouragement from patient peers | | Social Influences |
|  | One participant said that IDE was a positive topic for patients outside of the unit and that she thought it had improved spirits (Q42). I asked him if he was going to continue once the program was done, and—but I just found him to be a little—I thought he was maybe a little older, a little tired, but no, he was—he says he notices how even his spirits—and even when we go downstairs, like, he’s just all chirpy and happy about it.” | | | Observing/feeling the benefits | | Reinforcement |
| 5. Sutherland et al. (2021) | | | | | | |
| PA designed specifically for patients on dialysis  Sutherland et al. (2021) | Seven participants (35%) identified the need for tailored, professional help in increasing PA specifically for dialysis which was currently lacking for most participants. “I think nobody’s sort of helping me with that sort of thing [PA]. No-one is helping you to do these things or suggesting doing these things……I would like more outside activity.” | | | Tailored physical activity (by healthcare professional) suitable for dialysis patients | | Social Influences |
| PA supervised by experienced trainer  Sutherland et al. (2021) | Ten participants (50%) said that they would  like to be offered more physiotherapy, stretching or rehabilitation exercises as these would be suitable to their physical needs. | | | Willingness to engage | | Intentions |
|  | Some had experienced rehabilitation support from previous hospital inpatient admission and felt they would have benefitted from more. | | | Previous good experience of rehab support | | Reinforcement |
|  | They also pointed to the need for supervision, for example by a physiotherapist in a healthcare setting, their own  home or another designated area that was not a public space, and suggested that demonstrating the exercises in a group or on  a one-to-one basis would also be helpful. Only two participants (10%) mentioned  that they would prefer to attend a gym | | | Supervision by healthcare professional | | Environmental Context and Resources |
|  |  |  |  | Lack of privacy in IDE (*new barrier*) | | Environmental Context and Resources |
|  |  |  |  | Demonstration of the exercises | | Knowledge |
| PA in the company of friends  Sutherland et al. (2021) | Eleven participants (55%) felt that having someone to participate in PA with them would be beneficial and motivational and would help maintain a normal lifestyle and  sense of community outside of dialysis. Support from family members and good relationships with healthcare professionals (HCPs) were also identified as potentially important cues to action as was the offer of an exercise bicycle on their dialysis days. | | | Sense of community outside of dialysis | | Social/Professional Role and Identity |
|  |  |  |  | Social support | | Social Influences |
|  |  |  |  | IDE bikes available | | Environmental Context and Resources |
|  |  |  |  | Importance of patient’s family | | Social Influences |
| 6. Liu et al. (2020) | | | | | | |
| Internal motivators | “I mean the only way I’m going to get out of this chair is to get up and start moving around. If I sit here it’s just going to get harder and harder for me to do, so I have to try, I have to try”  There was recognition that the lack of physical activity was detrimental to health. Eight individuals reported intrinsic motivation as a facilitator, with sources including the wish to walk  again, the desire to live, and religious faith. “[I want] to run around this block, my biggest thing is to be able to walk again”, | | | Knowledge about the consequences of lack of physical activity | | Knowledge |
|  | Seven participants said they tried to do regular physical activity, such as taking walks inside their building. | | | Forming a routine | | Behavioural regulation |
|  |  |  |  | Already attempting exercise | | Intention |
|  | Eight individuals reported intrinsic motivation as a facilitator, with sources including the wish to walk  again, the desire to live, and religious faith. “God makes it easy for me to move” | | | Guided by spiritual beliefs | | Social/Professional Role and Identity |
|  | “Life motivates me to get moving. I love living” | | | Desire to live | | Goals |
|  | “[I want] to run around this block, my biggest thing is to be able to walk again” | | | Patients having intrinsic goals | | Goals |
|  | “I mean the only way I’m going to get out of this chair is to get up and start moving around. If I sit here it’s just going to get harder and harder for me to do, so I have to try, I have to try”, | | | Positive beliefs about physical activity (long-term benefits) | | Beliefs about consequences |
|  | “Don’t like laying around, bed makes you weak” “If I sit here it’s just going to get harder and harder for me to do, so I have to try, I have to try” | | | Desire not to decline | | Goals |
|  | “I mean the only way I’m going to get out of this chair is to get up and start moving around. If I sit here it’s just going to get harder and harder for me to do, so I have to try, I have to try”, | | | Knowledge about the benefits of physical activity | | Knowledge |
| Family and friend support | The support of family and friends was found to be a key facilitator for 7 participants. For example, family members would often verbally encourage participants to walk and leave the house. Socialization with neighbors similarly promoted physical activity. For 1 participant, the importance of her spouse as an enabler was emphasized after he died: “We were doing things together. But now when it’s just me I don’t have anybody like him. He would always kind of like motivate me to do something and I would be trying to get up, get dressed.” Once in a while my daughter, we go out to a movie or we go out to dinner, and sometimes on the weekend I have three grandbabies that want to stay with me”  “We go out walking, walking around sometimes, me and her [goddaughter], walking around the parking lot and stuff.”  “I walk downstairs, go to the mailbox and say hi to people that is down there, and then come back upstairs” | | | Importance of patient’s family | | Social Influences |
|  |  |  |  | Social Support from personal network | |  |
| Feasibility of incorporating into  routine activities  Liu et al. (2020) | Seven participants described how they turned  necessary tasks, such as grocery shopping, into opportunities for physical activity. Household chores, such as dusting, was cited by 2 participants as a source of physical  activity. One participant was employed and walked to her job, which was several blocks away. To facilitate physical activity, participants suggested the physical activity sessions at the hemodialysis center or their apartment building. Others described how they used the equipment at adult day care, which they already attended, to facilitate physical activity. “I’ll go to the grocery store, walk around there for a while, and go shopping”, “[He] walks to hemodialysis and walks back so that gets him exercise. Moves for about 15-20 minutes, takes his time”, “It would be good if there was something to do after hemodialysis”, “I socialize at my day care, have been going there for years to see my friends”, “I go to day care twice a week and use the exercise machines there” | | | Integrating with daily tasks | | Behavioural regulation, Environmental Context and Resources |
|  |  |  |  | Physical activity as part of job | | Environmental Context and Resources |
|  |  |  |  | Exercise at dialysis centre/unit | | Environmental Context and Resources |
| 7. Heiwe & Tollin (2012) | | | | | | |
| Active participation and self-esteem  Heiwe & Tollin (2012) | The participants were not informed before the intervention that intra-dialytic cycling is much easier than is submaximal ergometer-cycle tests. Those who had undergone submaximal ergometer-cycle tests were, therefore, sceptical of the implementation. These patients described how they had undergone submaximal  ergometer-cycle tests and experienced them as extremely physically demanding and, hence, were against all forms of exercise. All patients, however, were obliged to try  intra-dialytic cycling at least once as a part of the implementation and to describe their experience. The sceptical patients now experienced relief, as the intra-dialytic cycling  was experienced as being much easier. It was evident that experiences from earlier submaximal ergometer cycle tests were a barrier to accepting the implementation of regular, evidence-based intra-dialytic cycling and that information about this should be included in the implementation of the intervention, together with pilot cycling for all patients. These measures will increase the  probability of successful implementation. | | | Previous negative exercise experience which influences perception of IDE (*new barrier*) | | Reinforcement |
|  | The participants felt a high internal locus of control for three reasons. Firstly, they felt that they were taking an active part in their care, and secondly, they had an opportunity  to decide when to exercise during haemodialysis. Finally, they could control the resistance of the cycle. | | | Feeling of taking an active role in their care | | Behavioural Regulation AND Intentions |
|  |  |  |  | Being able to regulate own workload | | Environmental Context and Resources and Behavioural Regulation |
|  | And secondly, they had an opportunity  to decide when to exercise during haemodialysis. | | | Choice of when to exercise during dialysis | | Behavioural Regulation |
|  | It was evident that experiences from earlier submaximal ergometer cycle tests were a barrier to accepting the implementation of regular, evidence-based intra-dialytic cycling and that information about this should be included in the implementation of the intervention, together with pilot cycling for all patients. | | | Offering a pilot trial of IDE | | Knowledge |
|  | “I thought it was good with the resistance as you could regulate it as you saw fit.” | | | Being able to regulate own workload | | Environmental Context and Resources and Behavioural Regulation |
| An opportunity for physical activity and exercise | Patients regarded the implementation of intra-dialytic cycling as positive. | | | Positive attitude | | Optimism |
|  | They were all aware of the importance of regular exercise for those with chronic kidney disease and became even more so after having been informed and educated by the physiotherapist. | | | Knowledge about the benefits of physical activity | | Knowledge |
|  | They were all aware of the importance of regular exercise for those with chronic kidney disease and became even more so after having been informed and educated by the physiotherapist | | | Knowledge about the consequences of lack of physical activity | | Knowledge |
|  | They were positive about the idea of exercise but described how it was impossible to add exercise into their daily life. They wanted to increase the amount of exercise they undertook, in order to increase muscular strength, but suffered from time constraints. | | | Patients lack of time due to dialysis (barrier) | | Environmental Context and Resources |
|  |  |  |  | Desire to improve strength | | Goals |
|  | The implementation of intra-dialytic cycling was, therefore, appreciated, as it gave them the opportunity to use their time in haemodialysis to exercise. They regarded intra-dialytic cycling as a way of saving valuable nondialysis time, which could be used for other pleasurable activities and rest. Intra-dialytic cycling gave them more free time. “I wouldn’t need to come into the hospital on my free days [to exercise]...I think it’s a good way to get regular exercise as it’s not so easy to do it alone at home.” | | | Exercising during dialysis gives more free time | | Environmental Context and Resources |
|  |  |  |  | Exercise at dialysis centre/unit | |  |
| A distraction that interrupts the routine and boredom  during haemodialysis | Most patients undergoing haemodialysis treatment find the time on dialysis to be boring and monotonous. It was suggested to patients in the implementation of the  intervention that the cycling could be seen as something active and pleasant while on haemodialysis. This motivated the patients to try intra-dialytic cycling. The patients reported the intra-dialytic cycling to be a welcome distraction, something that interrupted the routine and made time pass more rapidly…  “It’s easier to bear the time you’re chained to the machine if you’ve got something to look forward to, something to pass the time ...cycling for example.” | | | IDE pleasant to pass the time on dialysis | | Environmental Context and Resources |
|  |  |  |  | IDE introduced as something active and pleasant while on dialysis | | Intentions |
|  |  |  |  | Distraction from treatment | | Emotion |
|  | This experience increased patients’ acceptance of the implementation  and functioned as a facilitator, as the patients talked about this experience with other patients, including those who were sceptical to the implementation. | | | Camaraderie and normalcy in the Unit | | Social Influences |
|  |  |  |  | Fostering a positive new common identity with other dialysis patients | | Social Influences |
| Curiosity and expectation | Most participants described how they became curious about the implementation of intra-dialytic cycling at an early stage, when the physiotherapist informed them about it. Most participants looked forward to trying it. Providing information about the beneficial effects of evidence-based intra-dialytic cycling had a beneficial effect on the implementation process."Patients described  how they had heard about the project through contact with patient organisations, and this facilitated the implementation process. Patients were curious to see how well they would perform. Patients expected that cycling would increase muscular strength.  “Yes, I was a bit curious. Curious and expectant perhaps, about what it would lead to.” | | | Curiosity and interest | | Knowledge |
|  |  |  |  | Willingness to engage | | Intentions |
|  |  |  |  | Perceived benefit to physical well-being | | Beliefs about consequences |
| The need for confirmation | The participants expressed the need to have someone to talk to while cycling. The implementation of the intervention included the physiotherapist being present while  a patient cycled, and this increased patients’ acceptance of intra-dialytic cycling. The need to be seen as an individual and receive oral confirmation and the need to  have behaviour reinforced and encouraged by the staff were both important. Reinforcement by staff could be  indirect (by positive body language, for example) or  direct (“Yes, go for it, you can make it!”). | | |  | | Social Influences |
|  |  |  |  | Encouragement from  staff | | Reinforcement |
|  |  |  |  | Camaraderie and normalcy in the Unit | | Social Influences |
|  | The belief that the physiotherapist would not be able to continue to attend sessions in order to assist and encourage the cycling was a barrier that had a negative impact on the success of the implementation in the long run.  “The staff came in and were a bit curious.... You felt like the centre of attention for a while.” | | | Lack of support from health professionals (barrier) | | Social Influences |
|  | Despite the importance of being confirmed as an individual, few participants reported that they had received comments, questions, or reactions from any of the other  patients in the room.  Some acknowledged that they had not asked other patients about their experiences but pointed out that the placement of the beds and haemodialysis chairs in the room was not optimal for conversation. This had a negative impact on acceptance and support for the implementation in the long run, and this  needs to be considered in the implementation of the intervention. | | | Dialysis environment restricts conversation/sense of community (barrier) | | Environmental Context and Resources |
| Physical reactions and a sense of well-being  Heiwe & Tollin (2012) | Patients did not feel that the exercise period of 30 minutes was unduly long (despite their initial anxiety of not being able to manage as long as 30 minutes). This gave rise to feelings of satisfaction and pride in having cycled for so long. These positive experiences created a situation in which patient preferences facilitated and supported the maintenance of the implementation as routine clinical practice. Patients were surprised that they could continue cycling and that this  reduced the unpleasant discomfort (lactic acid), and these experiences facilitated the implementation process. “You didn’t have to sit and tense yourself. . . just sit calm and relaxed and use your legs. . .I didn’t feel anything when I sat like this in bed and cycled. . . that was the good thing about it, I felt no strain at all on my back.” | | | Sense of pride and satisfaction from exercising | | Emotion/Reinforcement |
|  |  |  |  | Confidence in ability to maintain activity | | Beliefs about capabilities |
|  | Most felt acute muscular discomfort (lactic acid) in the legs after 10 minutes of cycling. They were glad that the physiotherapist was standing next to them and encouraged them to continue cycling, as the acute muscular discomfort passed. | | | Supervision by healthcare professional | | Environmental Context and Resources |
|  | The long period of immobilization during haemodialysis treatment often causes stiffness. Patients reported that this problem was reduced after their first session of intra-dialytic cycling. They also expressed satisfaction that their physical well-being increased. The reduced stiffness, retained mobility, and increased well-being functioned as facilitating experiences. Indeed, some patients experienced fewer leg cramps and feelings of restless legs after having cycled. | | | Observing/feeling the benefits | | Reinforcement |
|  | Most felt acute muscular discomfort (lactic acid) in the legs after 10 minutes of cycling. They were glad that the physiotherapist was standing next to them and encouraged them to continue cycling, as the acute muscular discomfort passed. | | | Encouragement from  staff | |  |
|  | Some patients experienced various physical symptoms during and after cycling. Patients found it easier to lie down while cycling than to sit on a conventional ergometer cycle, which in some cases was associated with pain and problems with balance. Furthermore, problems with breathlessness while exercising were less problematic while cycling in bed or a haemodialysis treatment chair. | | | User friendly IDE equipment | | Environmental Context and Resources |
|  | Some patients experienced various physical symptoms during and after cycling. Patients found it easier to lie down while cycling than to sit on a conventional ergometer cycle, which in some cases was associated with pain and problems with balance. Furthermore, problems with breathlessness while exercising were less problematic while cycling in bed or a haemodialysis treatment chair. | | | Pain during exercise(barrier) | | Reinforcement |
|  |  |  |  | Poor physical condition (barrier) | | Skills |
| 8. Jhamb et al. (2016) | | | | | | |
| Theme 1: Knowledge and perceived benefits of exercise Jhamb et al. (2016) | “Making you feel better and just making you able to do more things I guess.”  “Exercising keeps you on a [reduced] stress level”  ...majority of the participants’ comments reflected a view that a major benefit of exercise was improved overall perception of health and well-being. Physical health benefits such as improving  cardiovascular health, energy level, muscular strength and balance emerged as a prominent sub-theme. Especially in the patient interviews. Male patients seemed to focus more on the muscular strength benefits and females on cardio-protective benefits. | | | Knowledge about the benefits of physical activity | | Knowledge |
|  | “I have COPD and exercise can help me breathe better”  “I wouldn’t be doing quite as well as I am doing now if I wasn’t doing some form of exercise.”  ...majority of the participants’ comments reflected a view that a major benefit of exercise was improved overall perception of health and well-being. Physical health benefits such as improving  cardiovascular health, energy level, muscular strength and balance emerged as a prominent sub-theme. Especially in the patient interviews. Male patients seemed to focus more on the muscular strength benefits and females on cardio-protective benefits. | | | Perceived benefit to physical well-being | | Beliefs about consequences |
|  | Interestingly, only a few of the patients commented on the mental health benefits, such as ... an increased sense of accomplishment. | | | Feeling of accomplishment | | Reinforcement |
|  |  |  |  | Perceived benefit to mental well-being | | Beliefs about consequences |
|  | “Was on three blood pressure pills, now I don’t take any [after exercising regularly] | | | Observing/feeling the benefits | | Reinforcement |
|  | “It gives me more energy. And the more energy I have, the more I feel like doing things.” | | |  |  |  |
|  | I can go longer than I would be able to go if I wasn’t doing exercise at all.” | | |  |  |  |
|  | “It gives me a better appetite.” | | |  |  |  |
|  | “ I don’t have the tightness in my knees” | | |  |  |  |
|  | “I wouldn’t be doing quite as well as I am doing now if I wasn’t doing some form of exercise.” | | |  |  |  |
|  | We found that all but one patient (72 yo White female) and surprisingly one staff member (dialysis technician, 28 yo) reported no knowledge of benefits of exercise. Most patients were unaware or underestimated the recommended frequency and/or duration of exercise:    “Probably couldn’t be no more than maybe twice a week” [Interview 10, F, 54yo, Black] | | | Patients lack of knowledge of the benefits (barrier) | | Knowledge |
| Theme 4: Motivation for exercise comes from within and from the  encouragement of others Jhamb et al. (2016) | “At one time I wasn’t sure that [exercise] is what I wanted to do, I realized later that’s what I needed to be doing. I’m 100% for exercise, I know first-hand that it is beneficial … anything is better than nothing…. it’s not so much of trying to be the best in the gym or the best in the place just the fact that you’re there” | | | Observing/feeling the benefits | | Reinforcement |
|  | “I hate to use the word but you just suck it up and that’s about it. So the worst thing to do is to just sit around and mope so you don’t do that. ….I feel really guilty when I come home on these cold days and I can’t go out. I feel guilty about that.” | | | Feeling guilty if exercise is missed | | Behaviour regulation/Emotion |
|  | “Every day, every chance and all day we should do something. Even though you’re tired you try to do what you can. You benefit by being able to do a little bit more.” | | | Perceived benefit to physical well-being | | Beliefs about consequences |
|  | “Well I should be doing a lot more, if I could. And I mean mentally, it bothers me that I can’t run up the steps anymore like I used to or it takes me longer to get from the car inside the house.” | | |  |  |  |
|  | “ It’s a battle and you know you just can’t sit there because that’s not doing you any good” | | | Knowledge about the consequences of lack of physical activity | | Knowledge |
|  | Participants also identified health incentives and achieving health goals as important motivators to exercise. | | | Having goals - intrinsic | | Goals |
|  | “[Doctors advised] that I had to exercise, to lose weight, whatever it was that I could do that would cause me to shed those pounds, that’s what I did” | | | Recommendations of healthcare professionals | | Social Influences |
|  | “My doctor, the one that I deal with at clinic, I’d do anything he tells me to do” | | |  |  |  |
|  | We also identified a strong trusting relationship of patients with their doctors (primary care and nephrologists) and dialysis staff, and this was a key motivating factor, | | |  |  |  |
|  | “And I have a crew down there at the dialysis clinic that don’t let you do that [sit and mope around]. They encourage it [exercise]. … it’s just a good environment” | | | Patient encouragement from staff | | Social Influences |
|  | “You gotta have somebody that when you work out, you gotta have somebody to get you motivated, Somebody to push you!” | | | Accountability partner | | Social Influences |
|  | “I have friends that I walk with” | | |  |  |  |
|  | “I have a son that’s 26 and he goes to a gym 3 times a week and he’s always telling me I should Exercise" | | | Importance of patient’s family | | Social Influences |
|  | ...along with encouragement from family.. | | |  |  |  |
|  | “Yeah that’s usually how our patients are motivated is by lobby discussion [in dialysis unit waiting room] with each other. Yeah usually they’re more receptive to hearing it from another patient” | | | Camaraderie and normalcy in the Unit | | Social Influences |
|  | ““It has to be interesting for you, for you to want to do it. …… If you can find something that you like to do that occupies your mind then you might want to keep it up” | | | Enjoyment | | Reinforcement /Emotion |
|  | “Finding what they enjoy you know would be number one” | | |  |  |  |
|  | A number of patients expressed self-motivation to exercise, arising from either experiencing positive benefits... | | | Observing/feeling the benefits | | Reinforcement |
|  | ...or from recognition of loss of physical fitness after starting dialysis (Table 5). | | | Recognition of loss of physical fitness | | Reinforcement |
|  | Patients who had accepted dialysis as a lifestyle change seemed to overcome the psychosocial challenges of being more physically active. | | | Feeling of taking an active role in their care | | Behavioural Regulation |
|  | along with encouragement from ... friends and other dialysis patients | | | Social support from personal network | | Social Influences |
|  |  |  |  | Encouragement from patient peers | |  |
| Theme 5: Recommendations for intra-dialytic exercise (Jhamb *et al*. 2016) | “If you could do it [exercise during dialysis] that would be great. It’s kind of torture sitting in a chair for 3 h. I’ve always questioned why there wasn’t something for us to do there besides sit there. It would be good for us” mentally to have something else to do there while we are sitting in those chairs” | | | Distraction from treatment | | Emotion |
|  | Moreover, by adding distraction to the mundane routine of dialysis, participants felt that it would make the time go faster and add value to the dialysis time. | | |  |  |  |
|  | A recurring theme was the convenience in terms of saving travel and precious non-dialysis time. | | | Exercising during dialysis gives more free time | | Environmental Context and Resources |
|  | “I’m the kind of person says that if you can do it, I can do it [if you saw someone else exercise, could that be motivating?]” | | | Camaraderie and normalcy in the Unit | | Social Influences |
|  | They reiterated that since the dialysis unit is a social environment and if a culture of exercise is introduced, it is likely to be well accepted by the patients. | | | Fostering a positive new common identity with other dialysis patients | | Social Influences |
|  | “I think before the exercise is implemented they should give an example to see how the exercise is, if they would be able to do them and how far they should go because we’re not in that position to do all that they might want us to do” | | | Confidence in ability to perform activity | | Belief about Capabilities |
|  |  |  |  | Lack of confidence in ability to perform activity (*new barrier*) | | Belief about Capabilities |
|  | However, an individualized engaging program with prior testing of individual’s capabilities was important to the patients. | | | Lack of confidence in ability to perform activity (*new barrier*) | |  |
|  | “Yeah more flexible type because I can choose the intensities because when I get tired or when I’ve had enough, I’m going to stop.” | | | Choice within activity | | Behavioural Regulation |
|  | “They should put something like that [information on exercise] on the wall [in dialysis unit]” | | | Patient education on benefits of exercise is key | | Knowledge |
|  | “You have to find some kind of a benefit from what you’re doing or you’re not going to do it and when I do it, I feel good. I feel good about myself. I feel good because I’ve done it. And that’s the only payment you can get from it.” | | | Observing/feeling the benefits | | Reinforcement |
|  | Interestingly many patients identified positive health benefits and the ability to do more as internal motivators for engaging in exercise. | | |  |  |  |
| 10. Hu et al. (2024) | | | | | | |
| Theme1: MHD patients’ overall perceptual experience of the VR rehabilitation system: An exciting and enjoyable experience | An exciting and enjoyable experience VR is able to simulate the movement style that MHD patients enjoyed before dialysis, provide training programs tasked with specific goals, and stimulate the visual and auditory senses through rich game content to make patients more willing to try VR’s model of rehabilitation training and find it an enjoyable experience. P2: “I was more of a badminton player before, but after dialysis I played very little, but this device just simulates that movement, it’s not that tiring and it’s very comfortable.”P5:“It always feels like time flies when I participate in VR rehab.” P7:“I can always adjust the scene and background music when I do the tasks, and I can see my own score, and I get happy when I surpass others.”P10: “I set the highest score in a boxing game and it gave me a sense of achievement, it was a pleasure.” | | | IDE pleasant to pass the time on dialysis | | Reinforcement |
|  |  |  |  | Patients having extrinsic goals | | Goals |
|  |  |  |  | Feeling of accomplishment | | Reinforcement |
|  |  |  |  | Reminder of exercise previously enjoyed | | Social/Professional Role and Identity |
|  |  |  |  | Positive sense of competition between patients | | Social Influences |
|  |  |  |  | Choice within activity | | Behavioural Regulation |
| Theme1: MHD patients’ overall perceptual experience of the VR rehabilitation system: Motivations that drive potential rehabilitation | Traditional rehabilitation training is often boring andtime-consuming, and MHD patients do not complete it well dueto the impact of physical burden resulting in poor rehabilitationtraining [8]. VR provides a “novel” and “interesting” way of reha-bilitation training for the MHD group, which provides a varietyof personalized training programs, real-time feedback so thatpatients can see their own progress, and at the same time, theuse of VR rehabilitation system can provide the function of mul-tiple people online at the same time. At the same time, using VRrehabilitation system can provide the function of multiple peopleonline at the same time, and they can help each other in thegame system, which gradually becomes a new topic of commu-nication between patients, and they look forward to the next VRrehabilitation training, which strengthens the motivation ofpatients’ rehabilitation training. P1: “I found it interesting, it wassomething more different and he provided me with unexpectedsurprises compared to regular rehabilitation.” P2: “I did feel mymuscles being exercised while playing the game and overall hewas good for me and I would like to proceed to participate.” P8:“I like the networking and playing with others, and I can alsohelp each other in it without worrying about any levels that Ican’t get through.” P10: “I can always see my score and rankingin this, and I can also correct my wrong movements, for example,when I am boxing if I hit the wrong target, the system will remindme in time, it is very interesting.” | | | Emotion | | Lack of enjoyment/monotonous nature of PA/disliking exercising/finding it boring |
|  |  |  |  | Curiosity and interest | | Knowledge |
|  |  |  |  | Self-monitoring (specifically with technology) | | Behavioural Regulation |
|  |  |  |  | Sense of community outside of dialysis | | Social/Professional Role and Identity |
|  |  |  |  | Encouragement from patient peers | | Social Influences |
|  |  |  |  |  | |  |
| Theme 2: physical and psychological improvement of MHD patients by VR rehabilitation system: Gaining a sense of control | MHD patient group, with the increase of dialysis age various complications appear, greatly aggravate the patient’s physical burden, seriously affect the patient’s daily life [13]. Patients aspire to return to the level of physical activity they had before they became ill, and to return to society as soon as possible with appropriate social responsibilities. P5: “The VR system was able to simulate my previous level of physical activity and I was able to complete tasks in VR without much effort, giving me greater confidence in my recovery.” P6: “After being sick I spent most of the time sitting or lying down, sometimes I couldn’t walk very far, now with this device I can control my limbs to do a little movement again. P9: “He doesn’t just work on my own physical functioning, it has helped me to improve something about my memory and my ability to think things through, such as when playing a game of touch cubes, where I need to put a greater amount of effort into thinking about the routes of the cubes ahead of time in order to make sure that I get the score for the level.” | | | Patients having intrinsic goals | | Goals |
|  |  |  |  | Desire to be productive member of society | | Social/Professional Role and Identity |
|  |  |  |  | Desire to regain previous ability | | Goals |
|  |  |  |  | Observing/feeling the benefits | | Reinforcement |
| Theme 2: physical and psychological improvement of MHD patients by VR rehabilitation system: Psychosocial and emotional relief in MHD patients | Due to the incurable nature of the disease, MHD patients have to frequently go back and forth to the hospital for renal replacement therapy, which produces a relatively heavy economic burden, and at the same time, the contradiction between the decline in physical function and the inability to take on the responsibilities of the real society leads to self-negative emotions, of which anxiety and depression are common negative emotions in MHD patients [14]. P3: “It’s not quite the same as the exercise training that the doctors and nurses told me to do before, I’m not very resistant to the way it works anymore, and I’m in a better mood.”P4: “Since I’ve been ill, I can’t do any work around the house, and I can’t look after my two children, so it’s good to know that I’m confident that I can get back to my previous physical condition now that I’m using this machine.” P6: “I was on abdominal dialysis before, but now I’ve changed to hemodialysis, and just recently I’ve been more worried about the progression of my condition, and I’ve been more irritable, but I feel like I can relax a little bit more and be more comfortable after using VR.” P9: “I’ve been on dialysis for three years, three times a week, going back and forth to the hospital to dialyze, it’s really a bit tiring, but this hospital-zation through these few times of using this device, the scene changes and the music in it kinda relaxes me.” | | | Observing/feeling the benefits | | Reinforcement |
|  |  |  |  | Enjoyment | | Reinforcement/Emotion |
|  |  |  |  | Observing/feeling the benefits | |  |
|  |  |  |  | Desire to regain previous ability | | Goals |
|  |  |  |  | Positive beliefs about physical activity | | Beliefs about consequences |
|  |  |  |  | Knowledge about the consequences of lack of physical activity | | Knowledge |
| Theme 3: individualized needs of MHD patients during VR rehabilitation training: The need for specialized support for healthcare professionals | Since the MHD patients in this study were all new to the VR rehabilitation system, they showed nervousness about the operating procedures of the equipment and about the new things that appeared during the training process. Meanwhile, during the interviews, it was understood that some MHD patients lacked knowledge about their own rehabilitation training, and there were gaps in the intensity and frequency of the rehabilitation tasks they needed to accomplish, so the professional guidance of healthcare personnel during the rehabilitation training had a greater impact on the rehabilitation effect of the patients. P2: “It’s my first time to use this VR equipment, how to operate it in the beginning, I need the doctors and nurses to teach me a little bit in advance, but slowly get familiar with it.” P4: “In the process of using VR to play the rowing game, I feel that the whole person is involved, sometimes my body will follow the hull of the boat to the left and right, I am afraid of having a fall, and if there is a healthcare worker next to me, I will be very relieved.” P7: “I used to think that if I played all these games to a certain score, I could reach the goal of training, but then I realized from listening to the medical staff that the rehabilitation of my disease is along-lasting process.” | | | Knowing what to expect of IDE to address apprehension | | Knowledge |
|  |  |  |  | Demonstration of the exercises | |  |
|  |  |  |  | Group exercise ensures safety | | Environmental Context and Resources |
|  |  |  |  | Apprehension towards exercise | | Emotion |
|  |  |  |  | Lack of guidance/structure for patients | | Knowledge |
|  |  |  |  | Social support | | Social Influences |
|  |  |  |  | Tailored physical activity (by healthcare professional) suitable for dialysis patients | | Social Influences |
| Theme 3: individualized needs of MHD patients during VR rehabilitation training: The need for community and family continuity in the use of VRrehabilitation systems | Rehabilitation training for MHD patients is a dynamic adjustment and long-lasting process, and some MHD patients in this study expressed the hope that after discharge from the hospital there could be such a device in their own families and communities that they could continue to use VR for rehabilitation training during the interviews. P3: “The last time I used VR for rehab in the hospital my grandson and granddaughter saw it and they said it was really good, I’m thinking about getting a set of this equipment at home when I get out of the hospital.” P8: “Usually I need to go downstairs or go to the park to exercise, but now as long as there is a place for a computer at home, I hope I can exercise at home.” P10: “In the future, with advanced technology, I hope that the community center near my home will also have such equipment for our convenience, so that we don’t have to go back and forth to the hospital.” | | | Access to rehab equipment | | Environmental Context and Resources |
|  |  |  |  | Importance of patient’s family | | Social influence of family |
|  |  |  |  | Being able to exercise at home | | Environmental Context and Resources |
|  |  |  |  | Able to perform complementary activity as they wish in own time | | Environmental Context and Resources |
| 12. Wodskou *et al*. (2021) | | | | | | |
| Motivation for Intradialytic Exercise and Perceived Barriers: Expected Benefits of Physical Activity | Many patients described being physically active in their daily lives in terms of housework and gardening, and some participated in physical and/or social activities, such as  swimming, cycling, and fitness. Patients with musculoskeletal pain experienced physical  activity as having had a positive effect on their pain and expected that IE could reduce or  prevent pain. One patient talked about the body getting older and experiencing pain:  “The age . . . it starts to affect the legs, especially the knees when I lie still. When I  have been gardening, been out digging and such, it goes better when I have been  active. So therefore, I think, that some activity while lying here, either cycling  or doing an exercise program, that it will be be . . . it will be better. Because the  inactivity is not good”.  Other expected benefits included increased muscle mass, physical mobility, and  higher energy levels. Several patients felt it was important to maintain or improve physical  function to participate in meaningful activities and enhance quality of life.  “Well, my quality of life lies on my ability to be active, and if that starts to be  difficult then my quality of life goes too, so therefore I would very much like to  build something that can promote activity”.  Patients also mentioned wanting to live a long and healthy life and to be in shape for  a possible kidney transplant. Short-term expected benefits included preventing cramps  and resting better during hemodialysis, as well as having something to do during dialysis  while reserving time outside dialysis for other things. | | | Perceived benefit to physical well-being | | Beliefs about consequences |
|  |  |  |  | Positive beliefs about physical activity (long-term benefits) | |  |
|  |  |  |  | Maintain independence | |  |
|  |  |  |  | Knowledge about the consequences of lack of physical activity | | Knowledge |
|  |  |  |  | Desire to live | | Goals |
|  |  |  |  | Desire for independence | |  |
|  |  |  |  | Aiming to be healthy for transplant | |  |
|  |  |  |  | Desire not to decline | |  |
|  |  |  |  | Not taking up free time | | Environmental Context and Resources |
|  |  |  |  | IDE pleasant to pass the time on dialysis | | Reinforcement |
|  |  |  |  | IDE could improve experience of dialysis | | Beliefs about consequences |
| Motivation for Intradialytic Exercise and Perceived Barriers: The Intradialytic Exercise Program | All patients welcomed the suggestion that a physiotherapist would instruct them the first time they did IE, after which they would be required to do the exercises themselves. Patients shared opinions as to whether tablets were a good idea to illustrate the exercises or even necessary because the proposed program was simple enough to remember. One patient commented on the timing of exercise. She did not think that it should last more than 15–30 min at the beginning of hemodialysis to avoid exercise at the end of dialysis, when fluid removal can cause hypotension. Several patients thought leg exercises were relevant but also asked for exercises for the upper body, particularly the neck and shoulders, where they experienced pain. A patient knowledgeable about athletics said: “If it’s blunt needles, then I think you can do shoulder rolls and neck bends and all that”. Some patients thought exercise equipment could boost motivation and requested dumbbells, hand grip strengtheners, massage balls to roll underfoot, elastic exercise bands, bed bikes, and ball blankets to relieve muscle tension. Several types of equipment available would also allow a variety of exercises that took the limitations of hemodialysis into account. Patients suggested equipment could be stored in a box or locker like the ones in which they kept their hemodialysis equipment, allowing them to find it themselves without help from the busy nurses. Based on prior experience with physical activity, some patients felt that the social aspect would be motivating. They suggested that patients exercise simultaneously to, as one patient put it, “jazz each other up a little”. One patient thought that an element of competition could heighten his motivation, and another asked for variation, “so you don’t do the same thing every time”. | | | Demonstration of the exercises | | Knowledge |
|  |  |  |  | Supervision by healthcare professional | | Environmental Context and Resources |
|  |  |  |  | When patients can retrieve their own equipment and monitor themselves/low reliance on staff | |  |
|  |  |  |  | Patients taking responsibility for their own care | | Intentions |
|  |  |  |  | Camaraderie and normalcy in the Unit | | Social Influences |
|  |  |  |  | Positive sense of competition between patients | |  |
| 14. Rothpletz-Puglia *et al*. (2022)* *most of the data was general around the experience of dialysis. Only coded where specifically linked to physical activity. | | | | | | |
| Typical daily activities | “I do less physical running and less heavy lifting, but I still try to stay active, stay positive, talk to people, continue to pray, stay active in the church, and believe and do everything I can to live a normal life.” | | | Maintain independence | | Beliefs about consequences |
|  |  |  |  | Desire not to decline | | Goals |
|  |  |  |  | Patients having intrinsic goals | | Goals |
| Hardship | “And I have lupus going on with me too, so that can be rheumatoid arthritis. So I walk a lot, because I don’t want to stiffen up with the rain and all that stuff that’s going on.” | | | Perceived benefit to physical well-being | | Beliefs about consequences |
|  |  |  |  | Knowledge about the consequences of lack of physical activity | | Knowledge |
| The Movement to Achieve Typical Daily Activities | As opposed to structured exercise, all participants characterized their movement as daily physical activity, including lifestyle activities such as walking a dog, completing errands like grocery shopping, attending church, playing board games in a group living setting, participating in social events, going to the hairdresser, and arranging health care appointments. | | | Integrating with daily tasks | | Environmental Context and Resources AND Behavioural Regulation |
| Friends, Family, and Faith | In addition to family, 2 participants also mentioned the importance of their dog for their daily walking activity and companionship. | | | Importance of patient’s family | | Social Influences |
|  |  |  |  | Integrating with daily tasks | | Environmental Context and Resources AND Behavioural Regulation |
| Positive reappraisal | “So, my daughter said, well, Ma, you gotta be careful. I said I am careful. I take my time; I take my steps as far as I can go. When I’m tired, I stop. Okay. But I just wanted to get my legs back in order again. I can’t run as fast as I used to, but I can run a little bit like that, right. But I’m so glad, with dialysis—if it wasn’t for dialysis, I don’t think I would have been here.” | | | Desire to regain previous ability | | Goals |
|  |  |  |  | (*new barrier*) Family concern of ability | | Social Influences |
|  | “I want to live. I don’t wanna survice, either. I wanna enjoy life. I realize that my attitude is mine, my attitude. Nobody’s responsible to make me happy but me. I don’t sit there and dwell on negativity.” | | | Desire to live | | Goals |
| Purposeful | “So when I feel weak, I’m like no, I can’t just be down like this. I’ve got to push myself. I can’t stiffen up, and I can’t just be tired like this. I’ve got to go take care of my business. I’ve got to go. I’ve got to go take care of my business.” | | | Desire not to decline | | Goals |
|  |  |  |  | Desire for independence | |  |
|  |  |  |  | Maintain independence | | Beliefs about consequences |
|  | “When I go to do a project, or go to do some painting work, or go to just install a door, or do some plumbing job, or install a water heater, the next day. And that help me due to my condition. It help me feel good. It helps me feel real strong. Helps me feel positive.” “I fixed a door for her. How much do I owe.  you? Uh-uh, no. No, nothing. No, no, no. Mm-mm. It was me. I did that from the bottom of my heart. And make me feel so good, so useful.” | | | Integrating with daily tasks | | Environmental Context and Resources AND Behavioural Regulation |
|  |  |  |  | Desire to be productive member of society | | Social/Professional Role and Identity |
|  |  |  |  | Observing/feeling the benefits | | Reinforcement |
|  |  |  |  | Helping someone else is incentive to exercise | |  |
|  | Similarly, other engaged participants viewed activity to feel a sense of purpose. | | | Physical activity gives sense of purpose | | Reinforcement |
| Refocus on planning | “I said, you gotta come better than this, you gotta do something better. So, what I did was this. I would get up out of my bed and I would hold onto the wheelchair and I would step. And I learned how to walk myself in my daughter’s house, okay. They had to carry me down the stairs. From there, they brought me into the ambulance and brought me to dialysis. And from there I said, you know what? I gotta practice on my own. So, I used to go up and down the stairs. Take my time up and down the stairs, to where I didn’t have to use the wheelchair anymore.” “But my life is different now being on dialysis because I’m taking more control. It’s not like I have to go to work. I have to come to the hospital because it’s my health, but I have a choice, too. When I was in the workforce, it was just set. So, I wasn’t really planning.” | | | Desire for independence | | Goals |
|  |  |  |  | Feeling of taking an active role in their care | | Intentions |
|  |  |  |  | Desire to gain/maintain independence | | Goals |
|  | One participant worked each day to walk on her own to live independently again. | | | Desire for independence | | Goals |
|  |  |  |  | Feeling of taking an active role in their care | | Intentions AND behavioural regulation |
| Habitual Activity Patterns | Many participants talked about remaining active each day to maintain independence, and most talked about striving to do more even when it is difficult or when they feel weak. | | | Desire to gain/maintain independence | | Goals |
| 15. Huang et al., (2023) | | | | | | |
| Preparation for kidney transplantation: Meet the weight requirement of kidney transplantation | After all, there are some weight requirements for transplantation. Being physically active is still good for my weight control. (Q13, H)  Overweight patients usually lose weight through physical activity to successfully obtain additional rewards, that is, to reach the standard of kidney transplantation. | | | Aiming to be healthy for transplant | | Goals |
|  |  |  |  | Positive beliefs about physical activity (long-term benefits) | | Beliefs about consequences |
|  |  |  |  | Weight-loss | |  |
| Supervision of family members and nephrology staff (Relatedness): Guilt for family members | Because before I didn't raise dogs, I didn't sleep at night, could not come to bed in the morning and could not sleep normally. You see, when my son asked me to have this dog, he was telling me to go out and do more activities, rather than stay at home all the time. (Q14, B)  Family members of patients are important factors in the external supervision of patients' physical activities | | | Importance of patient’s family | | Social Influences |
| Supervision of family members and nephrology staff (Relatedness): Suggestions of nephrology staff | The doctors and nurses only asked me to control my weight during hemodialysis and try to keep it as long as possible (weight), but they didn't provide specific information about physical activity. (Q15, C)  I can walk just, and doctors don't let me run ah. Previously the director said to try to avoid strenuous exercise, do not take a big deal of movement, is to walk a walk, trot down, not too long. Do not race like others because, after all, I have the disease. (Q16, P)  I think it's good to walk, not to sleep at home. (Q17, A)  Undetailed health education also created external supervision for some patients. Some patients said that the staff let them do light physical activity; some reported that doctors do not allow patients to exercise vigorously. As a result, the patient concluded that walking every day is the most suitable activity for them. In addition, when staff asked patients to control their weight, the patients also indirectly knew that they needed to do physical activities. | | | Recommendations of healthcare professionals | | Social Influences |
|  |  |  |  | Tailored physical activity (by healthcare professional) suitable for dialysis patients | |  |
|  |  |  |  | Perceived benefit to physical well-being | | Beliefs about consequences |
| Keeping healthy is an important personal goal: Fear of rapid decline of physical condition | I was afraid that I would not be able to walk. These patients who have come here for more than ten years will be in wheelchairs. What should I do? I am afraid of this….There is a dialysis aunt here, who is over 60 and in good spirits. She always looks after her daughter. She walks two or three kilometers every day, and she deliberately goes to this distant vegetable market to buy vegetables. She has been through it for decades. I think she is in good spirits. (Q18, O)  There is a patient who doesn't like to walk and always lies down. She just suffers a lot. (Q19, G)  It is not surprising that many patients hope to obtain the expected health benefits from physical activity, including feeling healthier, and improving their sense of well‐being and quality of life. Although this form of motivation was initially an external motivation, accompanied by some fears about the consequences of being sedentary, many participants have internalized health threats as the result of changes of personal importance. Often, these motivations are based on participants' desire to avoid painful symptoms experienced by other people undergoing haemodialyses. For example, patients hope to make their legs strong enough through physical activity, slow down the decline of physical functions, be able to walk stably and delay the use of wheelchairs. Patients with poor health are considered as typical examples of a sedentary state, such as yellow or black skin, which is described as ‘visible on the face’. Some patients become active because they are afraid of being the same as the above patients. In addition, prolonging life is the ultimate goal of patients and one of the sources of motivation for patients to start physical activity. | | | Desire not to decline | | Goals |
|  |  |  |  | Maintain independence | | Beliefs about consequences |
|  |  |  |  | Inspiration from peers | | Social Influences |
|  |  |  |  | Seeing inactive peers decline | |  |
|  |  |  |  | Desire to live | | Goals |
|  |  |  |  | Desire to not be seen as visibly sick | | Social/Professional Role and Identity |
| Fulfillment of family and social responsibility (Relatedness): Assuming family responsibilities | I have a child over three years old. He basically stays in one place and plays all the time. Well, I'm going to stand there all day. It would be better if there was a seat to sit on. I would feel uncomfortable if I couldn't. Because I'm not very active in general, I can't support taking care of my children. (Q20, N)  In addition to improving basic Competence, some patients believed to keep healthy to get along with their families and fulfill their family responsibilities, as well as maintain their normal social roles and functions. Most patients reported that the frequent haemodialyses have brought financial burdens to the family and dragged down the family. A young patient reported that he wanted to maintain a normal family income and raise children, while the older patient believed that improving physical fitness would reduce the extra burden of troublesome children to transport themselves for haemodialyses treatment. Some patients also explained that showing their illness to the patient at the social level would arouse sympathy from others. Therefore, for patients, maintaining a person's normal social function is an important requirement for starting physical activities. | | | Importance of patient’s family | | Social Influences |
|  |  |  |  | Desire to be productive member of society | | Social/Professional Role and Identity |
| Fulfillment of family and social responsibility (Relatedness): Worry about losing job | I am a teacher. I go out to teach others. If people see me in a very poor state and guess that I am a patient, they will doubt whether my teaching content is correct, right? (Q21, E) | | | Declining condition could jeopardise profession | | Social/Professional Role and Identity |
|  |  |  |  | Do not want to be seen as visibly sick | |  |
| Support from family and patients (Relatedness): Encouragement and companionship of family | When it rains, my wife always accompanies me for a walk at Wetland Park. (Q22, E)  Over time, the family's companionship is an automatous regulation by which the patient can proactively integrate physical activity into life. Meanwhile, patients with similar physical functions spontaneously formed patient circles to encourage each other and discuss their physical activity experiences. | | | Social support from personal network | | Social Influences |
|  |  |  |  | Support from care partner | |  |
|  |  |  |  | Forming a routine | | Behavioural Regulation |
| Support from family and patients (Relatedness): Discussing with other patients | I have discussed with my fellow patients, just run three kilometers a day, walk two kilometers, anyway, this day. (Q23, O) | | | Inspiration from peers | | Social Influences |
|  |  |  |  | Seeing examples of dialysis patients doing exercise | | Beliefs about capabilities |
| Making the protocol your own: Stepwise change in personal exploration | My own understanding of staying active may mean moving every day. But you can't exercise vigorously, just do what you can. (Q24, J)  I started by walking five kilometers and then I figured it out myself. I added the amount, jogged for three kilometers, started running one kilometer, and could run two kilometers or three kilometers. Now I basically run three kilometers and then walk back, that's it. (Q25, O)  Participants mentioned how, over time, their lifestyle behaviours were motivated and progressively changed by the formation of a new pattern and routine. Most patients did physical activity according to their physical condition. The common stopping criteria for patients were subjectively ‘tired’ and ‘slight sweating’. The number of steps patients explored for this was typically 5000 to 10,000 steps. | | | Patients having intrinsic goals | | Goals |
|  |  |  |  | Patients taking responsibility for their own care | | Intentions |
|  |  |  |  | Being able to regulate own workload | | Behavioural Regulation |
| Making the protocol your own: Integrating into daily life | I may walk about 10,000 steps a day, if I don't come to the hospital. Every time (without hemodialysis), I just walk straight from our company to the subway line 3, and then it is estimated that it will be four or five stops, about 10,000 steps. (Q26, A)  Because the time and place for physical activity are flexible, patients can smoothly integrate physical activity into daily life, and can easily achieve the specified goals. | | | Integrating with daily tasks | | Behavioural Regulation  AND Environmental Context and Resources |
| Making the protocol your own: Using convenient resources effectively | I live on Fangwei Road, very close to the hospital. When I came for hemodialysis and when I left, I didn't take a car, I just walked, and I walked fast. (Q27, G)  I am jobless. Anyway, I send my wife to work in the morning, so I go running, which can also pass the time. (Q28, O)  I have a WeChat public account, where I can read and download related knowledge about people undergoing hemodialysis, and there is content on how to do activities. Anyway, just look at these (public accounts) information. (Q29, C)  For patients who go to work, commuting is the best way to increase physical activity. Walking on haemodialyses and housework were also appropriate methods for patient selection. Convenient activity resources, such as flexible time and accessibility to facilities, helped patients develop the habit of physical activity. For example, compared with gyms, parks were the most accessible and low‐cost resources for people undergoing haemodialyses. Internet information has become easy access for patients to learn about physical activity. | | | Active travel | | Environmental Context and Resources |
|  |  |  |  | Distraction from treatment | | Emotion |
|  |  |  |  | Desire to acquire specific knowledge about exercise | | Knowledge |
|  |  |  |  | Demonstration of the exercises | |  |
|  |  |  |  | Access to parks | | Environmental Context and Resources |
| Physiological and psychological effects (Competence): Physical function improvement | I think doing regular physical activity is also very powerful. In the past ten years, I have never had this flu, and I have not taken cold medicine once in more than ten years. I think this is an encouragement to me. (Q30, I)  Participants said that their new lifestyle behaviours were pleasant or personally satisfying.  Some of them described in detail how they gradually improved their physical condition after insisting on physical activity. | | | Observing/feeling the benefits | | Reinforcement |
|  |  |  |  | Perceived benefit to physical well-being | | Beliefs about consequences |
| Physiological and psychological effects (Competence): Full of energy and vitality | I don't have the energy if I don't walk. If I walk more often now, I will feel better. (Q31, L)  Patients reported how physical activity transforms negative emotions into positive ones, making them happier and more energetic. | | | Perceived benefit to physical well-being | | Beliefs about consequences |
|  |  |  |  | Perceived benefit to mental well-being | |  |
|  |  |  |  | Observing/feeling the benefits | | Reinforcement |
|  |  |  |  | Improvement in energy levels | |  |
| Physiological and psychological effects (Competence): Reduced restrictions on basic needs | I can't drink water if I'm not active. Because as soon as I drink water, the weight will increase, and then the amount of hemodialysis will increase. If the amount of hemodialysis is increased, I will suffer. I walk more every day; I can sweat and drink more water. (Q32, R)  Drinking water is the most basic physiological demand of patients, and it is also the basic life function limitation of haemodialyses. Physical activity helps patients sweat and drink more water, which has become an important way for patients to control their weight besides haemodialyses. | | | Observing/feeling the benefits | | Reinforcement |
| Enjoyment (Autonomy): Sense of achievement | I think it's still useful to go out and walk around every day. The doctors all said, ‘You see this old lady is still doing well’. I think this sentence gave me a lot of encouragement. (Q33, I)  The patient's adherence to physical activity promoted the formation of a new lifestyle. The stronger the physical and mental benefits they perceived, the greater their sense of achievement, which encouraged the patient to keep going, creating a virtuous circle. The sense of achievement also came from the improvement of the patient's own comprehensive state, especially when compared with other sedentary patients. The affirmation of professionals made the patient's sense of achievement reach its peak. | | | Feeling of accomplishment | | Reinforcement |
|  |  |  |  | Encouragement from staff | | Social Influences |
|  |  |  |  | Feeling of taking an active role in their care | | Intentions AND Behavioural Regulations |
| Enjoyment (Autonomy): Enjoying being active | Last year on National Day, even though it was cold and rainy, I ran. I was running hot, sweating, and feeling very comfortable. Now I enjoy running more and more. (Q34, O)  I was physically active every day. Even though I was a little uncomfortable that day and did not want to run, I would still walk, taking a 20 minute, nearly 30 minutes path. (Q35, P)  Enjoyment of physical activity was an important determinant of maintaining physical activity. The patient spoke with pride when it came to running when it rained and going out for a walk with his or her neighbours when it snowed. | | | Enjoyment | | Emotion |
|  |  |  |  | Sense of pride and satisfaction from exercising | | Emotion AND Reinforcement |
| 16. Sheshradi et al. (2020) | | | | | | |
| Maintain or improve functional ability | ‘I just want to be able to do the things I used to do. Stand up straight and tall. Go on hikes with my family. We used to hike all around Marin’.  ‘I’ve got this walker, so I guess I should be using it to walk. If I do that enough, maybe I won’t have to use it anymore’. | | | Desire to regain previous ability | | Goals |
|  |  |  |  | Patients having intrinsic goals | |  |
|  |  |  |  | Importance of patient’s family | | Social Influences |
| Maintain or improve activity level | ‘I used to be a lot more active before dialysis. I want to get back to where I was before all this’. | | | Desire to regain previous ability | | Goals |
|  |  |  |  | Patients having intrinsic goals | |  |
|  |  |  |  | Desire not to decline | |  |
| Lose weight for kidney transplantation | ‘I have this goal of losing one and a half kilos each month so I can get back on the list’. | | | Patients having extrinsic goals | | Goals |
|  |  |  |  | Aiming to be healthy for transplant | |  |
|  |  |  |  | Weight-loss | | Beliefs about consequences |
| Other personal motivation | ‘I’m going to get a new service dog, and this’ll be a good time to train him’. | | | Integrating with daily tasks | | Behavioural Regulation  AND Environmental Context and Resources |
|  |  |  |  | Patients having intrinsic goals | | Goals |
| 17a. Young *et al*. (2015) (pre implementation of IDE) | | | | | | |
| Enhanced  knowledge and  skills Young *et al*. (2015) | | “I suffer a lot from cramp, would the cycling  make any difference to that?”  All patients anticipated a wide variety of potential improvements, primarily reduction in symptoms, better cardiovascular health and confidence. Patients wanted more information about the benefits of IDE and what participation would involve. Patients proposed that an opportunity to ‘try’ IDE without commitment would enhance their confidence, enable informed decision-making and reduce fears. | Curiosity and interest | | Knowledge | |
|  |  |  | Knowledge about the benefits of physical activity | |  |  |
|  |  |  | Offering a pilot trial of IDE | |  |  |
|  | |  | IDE could improve experience of dialysis | | Beliefs about consequences | |
| Assessment Young *et al*. (2015) | | “One of the problems when you come of dialysis is that... your legs have stiffened up...if it will help with that, brilliant” “If the BP could improve it might motivate people to take part” | IDE could improve experience of dialysis | | Beliefs about consequences | |
|  |  |  | Perceived benefit to physical well-being | |  |  |
|  |  | “I quite enjoy [exercise]...and I tend to push things a bit...but I don’t know now whether that’s a good or a bad thing. I’d like to ask someone”  “How long would you exercise for if you did do it?” | Knowledge to reassure and encourage patients | | Knowledge | |
|  |  |  | Tailored physical activity (by healthcare professional) suitable for dialysis patients | | Social Influences | |
|  |  |  | Knowing what to expect of IDE to address apprehension | | Knowledge | |
|  |  | “I think until you’ve tried it you don’t know, you have to try it” |  |  |  |  |
| The influences of  peers and  colleagues Young *et al*. (2015) | | “People talk and everybody on the units tend to know each other and then say well I had a go on [the bicycle], it’s great, you know, and then more people will come forward I think” “I think if patients see the next person cycling they’ll say I cycling they’ll say I could do it”  Patients described being strongly influenced by their peers and felt that seeing others they viewed as similar exercising on haemodialysis would alleviate their fears and positively influence how capable they felt to participate in IDE. The desire for peer support was particularly relevant for female patients from minority ethnic backgrounds | Encouragement from patient peers | | Social Influences | |
|  |  |  | Inspiration from peers | |  |  |
|  | |  |  | |  | |
|  | |  |  | |  | |
|  | | “If people are negative about it then you’ll get other people oh I don’t want to do it either.”  Patients from two focus groups, however, suggested that any negative experiences may dissuade others. Social influences | Negative comments about IDE from patients | | Social Influences | |
| Assessment Young *et al*. (2015) | | “I think I’d want an ok from my consultant to say that you are fit enough to do it for a start. Because obviously underlying problems again, you know, could make a difference to what you do and how long you do it.” “You want to tailor the thing to your specific needs. You can’t have one size fits all.” “If you know you’re going to get another assessment, you want to be better. It gives you more encouragement to do it and you don’t want to fail. You know it’s going to be monitored so you tend to be a little bit more committed.”  All patients in the pre-implementation  phase felt an exercise assessment, including Nephrologist approval, was imperative prior to  embarking on an IDE programme. Patients deemed this assessment necessary to determine  safety and for exercise to be tailored in light of their individual co-morbidities, ages and frailties, which were considered to be important determinants of exercise ability. Such assessment  was anticipated to further increase self-belief in relation to IDE participation. Ongoing assessment of progress was also anticipated to help maintain long-term motivation by reinforcing  the benefits of exercising | Physician involvement | | Social Influences | |
|  | |  |  | |  | |
|  | |  | Tailored physical activity (by healthcare professional) suitable for dialysis patients | |  | |
|  |  |  | Patients having intrinsic goals | | Goals | |
| Exercise  professional  support Young *et al*. (2015) | | “I think Physiotherapist would be trained. I  do not feel nurse would know much.”  “I would have thought [an exercise  professional] ought to set [the bike] up for  us really. Set it up properly and tell us what  we‘re capable of.”   “Who’s going to be there to give in-depth  advice and answer questions, [an exercise  professional] but also fairly clued up as to  our problems as renal patients... who can  give an honest kind of an answer how far I  can go with the exercise bike.”  Patients felt that assessment should be conducted by a professional with both renal and exercise expertise. | Additional, exercise specific staff | | Environmental Context and Resources | |
|  |  |  | Exercise monitoring/assessment/provision not included in care provision/ not the nurses’ role | | Social/Professional Role and Identity | |
|  |  |  | Tailored physical activity (by healthcare professional) | | Social Influences | |
|  |  |  | Knowledge to reassure and encourage patients | | Knowledge | |
| 17b. Young *et al*. (2015) (post implementation of IDE) | | | | | | |
| Positive outcomes of participating in IDE Young et al. (2015) | | “I used to struggle with my blood pressure, towards the end it always used to drop, I wondered if the exercise would help to stabilise it and it did, so that was a plus” (56 year old male Asian patient) “I can now walk up to the village which is about half a mile and I feel it’s the cycling that’s helped” (75 year old female White British patient) “Because of the exercise I can sleep better. I can sleep 5–6 hours at a time” (67 year old male Asian patient)  Post-implementation all patients  described experiencing personal improvements as their main motivation to continue. These  were primarily enhanced functional abilities (e.g. increased walking capacity), better physiological and psychological health (e.g. blood pressure control, improved mood) and a reduction in  symptoms. Such improvements were evident through reassessment, enhanced exercise performance and patients observations of improvements within their daily lives. | Observing/feeling the benefits | | Reinforcement | |
|  |  |  | IDE could improve experience of dialysis | | Beliefs about consequences | |

## Barriers

Any facilitators coded in themes that were categorised as barriers are highlighted in green

| Original barrier  /Study | Data | New barriers (after splitting) | TDF domain mapped to |
| --- | --- | --- | --- |
| 1. Song et al. (2019) | | | |
| Self-Perceived Barriers to Exercise  Song et al. (2019) | “The barriers depend on patients’ physical condition. 98% of people like us have hypertension, which is contradicted with overloaded exercise”, , “I may get cold even if just loosen my clothes, so I dare not work out to sweat. . . I will have a cold”, “Doing exercise in the gym is too vigorous. You know, strenuous exercise is not suitable for patients with HD, which is not harmful to Shen Qi” | Misconceptions about the relationship between physical activity and condition(s) | Knowledge |
|  |  | Exercise options in local gyms are unsuitable for dialysis patients | Environmental Context and Resources |
|  |  | Cultural beliefs about physical activity | Social/Professional Role and Identity |
|  | “I found I’ve got osteoporosis this year. I assume people with long-term HD have the same condition with me, suggesting our bones are frail. So bone fracture may occur if exercise is performed” | Patient belief that condition(s) preclude physical activity | Beliefs about capabilities |
|  |  | Concern injury and accident | Belief about consequences |
|  |  | Poor physical condition | Skills |
|  |  | Other health conditions |  |
|  | “I may get cold even if just loosen my clothes, so I dare not work out to sweat. . . I will have a cold” | Concern exercise may lead to sickness | Beliefs about consequences |
|  | “I rarely do exercise. I have never thought about it at all. I am lazy . . . I have no  motivation to exercise . . . no reason, no motivation, and I am used to it” | Preference is not physical activity | Intentions |
|  |  | Lack of motivation |  |
|  |  | Used to being sedentary | Behavioural Regulation |
|  | “The predominant barrier is the cold weather now. I feel I would be more active if spring was coming . . . I am afraid of cold, so I just wanna stay in a room with air conditioner in the winter and chatting with family” | Bad weather | Environmental Context and Resources |
| Negative Influences from Others  Song et al. (2019) | Patients expressed that their local health care providers advised them to take more rest and restrict the load of exercise or physical activities to avoid deterioration in kidney function and/or various unpleasant symptoms or comorbidities. One participant reported this: “The local doctor told me that I needed more sleep and patients like me were not allowed to do physical work in fear of getting too tired before my dialysis session started” | Lack of guidance from healthcare professionals | Social influences |
|  | “My family don’t understand what exercise is about due to poor educational  background. Actually, they have never mentioned it to me . . . They take care of  me and have concern about my physical condition”, “Both my daughter and daughter in-law work in this hospital. They asked me not do any exercises, because they don’t want me too tired”, “My husband always says that you don’t have to have concern about anything and don’t do any physical activity in order to avoid to further impair my Shen Qi”, “In terms of doing household work, my family have never expressed their attitudes . . . but I have to look after my little granddaughter and do housework because my children are too busy working”, “I don’t have to do anything, because my mum and wife can do everything. There is no need for me to do housework”, Few verbal support is like “You can do exercise as you need, but you need to be based on your physical condition”, Nearly the other half of patients told us that their family members did not allow them to exercise because they considered exercise inappropriate for patients undergoing HD due to potential fatigue and injury caused from exercise—for example, “My family thinks exercise makes me feel tired, so all they tell me is to have more rest” | Lack of support | Social influences |
|  |  | Family/friends lack of knowledge of condition(s) | Social influences |
|  |  | Direct guidance not to exercise from family |  |
|  |  |  |  |
| Limited Exercise Information Resources  Song et al. (2019) | Nearly all patients reported that they had never been advised by health care professionals to carry out any types of exercise. Using a plastic ball to enhance arteriovenous fistula’s function was the only type of exercise recommended by health care professionals. Although some patients attempted to do certain types of exercise, they were unable to obtain enough reliable information from health care professionals about which exercise is suitable for them and how to avoid impairing fistula or causing injury. One participant reported how eager she was to learn yoga, but said,  I really have concern about my fistula because keeping balance or supporting my body with hands is the fundamental motion of yoga . . . No one can provide me with any advice or suggestion . . . I don’t trust the yoga coach, because she lacks professional knowledge of my disease.” | Instructor lack of knowledge of condition | Environmental Context and Resources |
|  |  | Lack of guidance from healthcare professionals | Social Influences |
|  | Meanwhile, patients reported that they had no aspiration to ask doctors for information about exercise unless doctors spontaneously recommended or guided them to perform exercise. For example, “Doctors and nurses are too busy to talk with us, so I don’t think it would be proper to ask them about how to do exercise” | Staff appear too busy to help with IDE |  |
|  |  | IDE could disrupt the routine on the dialysis unit | Environmental Context and Resources |
| Lack of enough  information  support (used as a code on its own)  Song et al. (2019) | “Doctors told me to do exercise moderately, and it is beneficial. But they didn’t say anything about specific exercise intensity, so I understand exercise as walking” | Lack of enough information support for patients | Knowledge |
| Perceptions about Exercise - Utilisation of exercise facilities (not grouped as barrier or facilitator by paper but does include data)  Song et al. (2019) | Patients also mentioned that using an exercise facility tended to challenge them with “high membership cost”, “risks of injury”, and “crowded environment”  All but one young man (Male, age 25) described a local exercise facility as unsuitable and were unwilling to utilize it. The most prominent reason behind this point of view was that they perceived the modalities of exercise performed at exercise facilities as too strenuous. Patients also mentioned that using an exercise facility tended to challenge them with “high membership cost” (Male, age 48), “risks of injury” (Male, age 77), and “crowded environment” (Male, age 35). | Cost of exercise facilities/equipment | Environmental Context and Resources |
|  |  | Some facilities pose high risk of injury |  |
|  |  | Facilities too crowded |  |
| 2. Kontos et al. (2007) | | | |
| ESRD, its sequelae, and other comorbidities  Kontos et al. (2007) | “You need to have some time to recuperate from dialysis. As you know it’s exhausting, and it takes a while until you get over it, a few hours. I mean, overnight sleep is fine. But you find yourself in the morning exhausted, and still you want to sleep again. I don’t know about other people but that is how I feel.”, In reference to his wife who receives dialysis treatment, a family care provider similarly commented: “Dialysis saps your energy. The fatigue, oh the fatigue.” Comorbidities of various kinds were experienced by all patient participants and were identified as barriers to exercise as exemplified in the following statement: “I feel somewhat exhausted after a bit of exertion, you know. Of course I have a bit of a breathing problem, bronchitis, that goes with the lung disease, taking puffers and so on. I also had a double bypass. I had a lot of things happening, that may have something to do with why I don’t exercise. Having kidney disease, heart disease, lung disease and the rest of it and  being the age I am, I’m 76 years old now, that doesn’t really put you in the frame of mind to start any marathon.”, “And we all have osteoporosis at some level, and arthritis. And the pain and the discomfort...  ruins your will to continue. You say to yourself, ‘it’s always some other thing’’ and ‘‘what good is it doing to me?’’ and you can get very depressed about it. And I think that takes away the will to do any exercise. | Other health conditions | Skills |
|  |  | Lack of physical ability |  |
|  |  | Fatigue |  |
|  |  | Belief that age limits capacity | Beliefs about capabilities |
|  |  | Pain (general) | Emotion AND Skills |
|  |  | Poor mental health | Skills, Beliefs about capabilities |
|  |  | Lack of perceived benefit | Reinforcement |
|  |  | Lack of benefit | Beliefs about consequences |
| Equipment  Kontos et al. (2007 | In both hospitals, only upright exercise bicycles  were available for patients who wished to exercise  during dialysis. Patients who were restricted to a  reclining position during treatment were consequently unable to use the bicycles. As one nurse  stated, ‘‘The ones with low blood pressure, they  cannot do the bicycle because they have to be sitting  up to do the bicycle, and with low blood pressure  you have to lie them down with the legs higher.’ | IDE equipment not suitable for everyone | Environmental Context and Resources |
|  | In addition, family care providers and patients expressed fears that using the bicycle would damage the fistula or chest line | Fear of fistula damage | Beliefs about consequences |
| Time  Kontos et al. (2007) | “You don’t have time at home. I come to dialysis 3 days a week. I get up at 5 am and I get home about 1:30–2:00 pm and then I have to have a nap, I have household chores, I have bills, I have garbage, I have all kinds of stuff. I don’t have time to exercise at home.”, | Patient lack of time due to dialysis | Environmental context and Resources |
|  |  |  |  |
| 3. Sieverdes *et al*. (2015) | | | |
| Living with Chronic Illness | Participants described how their lives had been affected by renal failure, comorbid conditions, and dialysis. Factors such as lower energy levels on dialysis days and changes in daily routines, habits, and social interactions inducing stress and bringing on an awareness of decreased quality of life were described. | No barriers/facilitators coded from data |  |
| 4. Thompson *et al*. (2016) | | | |
| Norms within the Dialysis Unit (patients) | Patients described aspects of the unit’s social structure that were barriers to receiving assistance with IDE. The existing processes for obtaining help from staff (ringing the bell) were viewed as inappropriate for IDE (Q33). One patient expressed concern that using the bell for help with exercise could have negative consequences when help was urgently needed. | No appropriate route for patients to request assistance with IDE | Environmental Context and Resources |
|  |  | Asking for help with IDE could result in less help for more important things | Beliefs about consequences |
|  | For one patient, not being a “bother” by asking for things was important to the role of the “good patient” (Q34). “I don’t like asking them for anything. I’m just not that kind of person. I’ve never asked for help in my whole life. I’m just a person that goes and do stuff. But I suppose I could. I mean, like, when I want my cup of tea, I usually wait until one of them will come, and then I’ll ask—although this morning, I didn’t; I had to call them. But I don’t like to bother them, because they’re busy, and so I try and bother them as little as possible, and I think they appreciate that.” Q34 | Identity as a ‘good’ patient | Social/Professional Role and Identity |
|  | Some participants were concerned that IDE would disrupt the “routine” of the unit (Q32) “…if we want something to do with the equipment, we would have to push the red button, which somebody up front’s got to answer the red button, and it disturbs—then it would disturb everybody’s routine.” | IDE could disrupt the routine on the dialysis unit | Environmental Context and Resources |
|  | "They’re never just convenient to wave down. You know, you’ve got to ring your bell, and then if you start ringing your bell for frivolous things, then they start ignoring you later when you really need them to come when you ring the bell.” | Staff appear too busy to help with IDE | Social Influences |
|  |  | Lack of support from health professionals |  |
| 5. Sutherland et al. (2021) | | | |
| The demands of PA are incompatible with dialysis. | Most participants found that dialysis reduced motivation to undertake PA.. believed dialysis reduced their capacity to continue with regular physical activities or muscle wasting.…you can’t do much especially when you are in a dialysis centre….dialysis comes in and dominates your life a bit… | Patient lack of time due to dialysis | Environmental Context and Resources |
|  |  | Lack of in-centre options |  |
|  |  | Lack of physical ability | Skills |
|  | Tiredness was also commonly perceived as a barrier: 17 participants (85%) reported they felt too tired to participate in PA especially on dialysis days | Fatigue | Skills |
|  | Concern that something may happen to their fistula (dialysis access) if they exercised during dialysis was common. | Fear of fistula damage | Beliefs about consequences |
|  | Most participants found that dialysis reduced motivation to undertake PA, (five believed dialysis reduced their capacity to continue with regular physical activities or muscle wasting.…you can’t do much especially when you are in a dialysis centre….dialysis comes in and dominates your life a bit… | Lack of motivation (because of dialysis) | Intentions |
|  |  | Lack of perceived benefit | Beliefs about consequences |
|  | including some who felt that if the opportunity arose, they would not take it: 12 participants (60%) | Lack of interest | Intentions |
| PA presents a risk for patients on dialysis  Sutherland et al. (2021) | Fourteen (70%) participants on dialysis feared that PA would cause further pain or other adverse consequences. | Fear of pain | Beliefs about consequences |
|  | Six (30%) participants found that their fear of falling limited daily activities including walking, although others felt less at risk if they used a stick or other mobility aid. | Fear of falling | Beliefs about consequences |
| 6. Liu *et al*. 2020 | | | |
| Lack of motivation | For 6 participants, a subtheme was a lack of motivation, as shown in Table 4. The lack of a motivator, either external or internal, was perceived as an often insurmountable hurdle. “I don’t have anything to motivate me to say, well let’s get up and do this. Let’s go take a walk”  •“I feel like I’ll get up later, I’ll get up in a few minutes. I keep laying there” | Lack of motivation | Intentions |
| Effect of medical conditions, especially pain | Medical conditions were a barrier for 5 participants. Although the interview guide did not have questions about pain, 5 participants independently cited pain as a limitation. Dizziness was another barrier; 1 participant stated he/she often rested to relieve dizziness. One participant was blind and felt uncomfortable without his wife nearby. | Poor physical condition | Skills |
|  |  | Other health conditions |  |
|  |  | Pain (general) | Emotion AND Skills |
| Environmental restrictions | Physical accessibility and local weather played a major role for 4 participants. One participant cited the difficulty of stairs; 2 cited uneven sidewalks as barriers. Weather was also a common concern because the participants lived in an area with frequent snowfall, causing slippery walkways. “I don’t go to people’s houses unless I know what kind of step they have”  “I don’t do too much outside walking when there is ice on the ground” | Local environment risks | Environmental Context and Resources |
|  |  | Bad weather |  |
| 7. Heiwe & Tollin (2012) | | | |
| The design and function of the intra-dialytic cycle  Heiwe & Tollin (2012) | The needles in the arteriovenous fistula/graft must not be moved while exercising. It was hypothesised that this would be a factor that would scare the patients and discourage them from performing intra-dialytic cycling, even though the implementation of the intervention included information that there were no risks for their arteriovenous fistula/graft. However, none of the participants related that they had experienced any problems with this. They described how the design and function of the cycle were more important to them | Fear of fistula damage | Beliefs about consequences |
|  | Most patients experienced the cycle as easy to handle without assistance and, thus, regarded it as a useful tool for exercising while undergoing haemodialysis. “I was surprised how easy it was to cycle right there in bed.” The phase of the implementation during which the cycle was designed and modified based on pilot patients’ opinions about the cycle facilitated successful  implementation. | User friendly IDE equipment for patients to use | Environmental Context and Resources |
|  | Some patients experienced shortcomings in the design of the intra-dialytic cycle. Those who cycled energetically  complained that the cycle was not completely still while cycling. Some found it difficult to keep their feet on the pedals, while others found the pedals to be inflexible. These experienced shortcomings functioned as barriers to intra-dialytic cycling and had a negative impact on the implementation process. “Well, it wasn’t quite firm, it moved around a bit. . ..” | IDE bikes not stable | Environmental Context and Resources |
| Doubtful thoughts and emotions  Heiwe & Tollin (2012) | Patients had been informed by the physiotherapist, as part of the implementation of the intervention, that the workload would be sufficiently small for all patients to be able to cycle for 30 minutes. Patients, however, reported feelings of worry, fear, and doubt that they would not have the physical capacity required to cycle for 30 minutes and would have to interrupt the session. | Lack of physical ability | Skills |
|  | Some patients had problems with lower or upper back pain and were concerned that cycling in bed or the haemodialysis chair would increase their pain. The participants also worried that they would experience cycling as boring in the long term, and this would cause them to drop out. | Fear of Pain | Beliefs about consequences |
|  |  | Pain (general) | Emotion AND Skills |
|  |  | Poor physical condition | Skills |
|  | The participants also worried that they would experience cycling as boring in the long term, and this would cause them to drop out. | Lack of enjoyment/monotonous nature of PA/disliking exercising/finding it boring | Emotion |
|  | Some thought of cycling as a strenuous activity and feared muscle tiredness | Fear of further fatigue | Beliefs about consequences |
|  |  | Not knowing what to expect from exercise | Knowledge |
| Concern for the work situation of the healthcare  providers | The implementation of the intervention did not include providing any information to the patients about how the implementation would affect the work situation of the healthcare providers. The participants, however, expressed concern that the work situation of the healthcare providers would be negatively affected by the implementation of intra-dialytic cycling. They did not want to burden the staff and expressed worry that evidence-based  intra-dialytic cycling would cause stress for the staff, as it would probably be time consuming and energy consuming to place the cycle at the end of the patient’s bed. Patients did not want to burden the staff,  and this attitude was thus a barrier to the implementation process. The patients considered the cycle to be heavy, and they perceived this as an obstacle for staff.  The idea that cycling was a burden for nurses became another barrier for successful implementation, even though the participants never heard the staff mention  this, and the cycle had been designed to be easy for the staff to handle (with the aid of wheels and a lifting system). “. . .you [the staff] get a sort of extra job when you have to set up the bike and adjust it to each patient. . .” | Patients fear being a burden to staff | Beliefs about consequences |
|  |  | IDE could disrupt the routine on the dialysis unit | Environmental Context and Resources |
| 8. Jhamb *et al*. (2016) | | | |
| Reported barriers to exercise: dialysis makes exercise challenging | “I was very active at one time and now I’m very tired. You can’t make your body do what you want it to do either all the time because you’re tired” | Fatigue | Skills |
|  | “I think the dialysis itself takes enough out of them. I know it does for me; when I go home I have such a headache, I lay down and sleep for a couple of hour” |  |  |
|  | Fatigue or lack of energy, especially post-dialysis fatigue was universally cited as the biggest barrier to exercise and was described as “drained”, “just don’t feel like doing anything after HD” |  |  |
|  | “You can’t do weights anymore when you’re on dialysis… [because of fistula]” | Fear of fistula damage | Belief about Consequences |
|  | “I was always worried about my fistula if I would fall or something." |  |  |
|  | Limitations on lifting weights due to fear of injuring the fistula |  |  |
|  | inability to do water exercises due to dialysis catheter | Fistula/catheter prevents activity | Environmental Context and Resources |
|  | “Based on the fact that I now have a catheter and the fistula, it’s prohibited of me from going back to that [water pool exercise class]” |  |  |
|  | “I have Band-Aids on the sites that were where the needles had been and I don’t want to do anything that would cause them to bust or start bleeding again. So those days I don’t do any exercise” | Concern injury and accident | Beliefs about consequences |
|  | Several participants commented on the poor overall health and comorbidities such as arthritis, amputations, leg weakness and blindness as common barriers. | Poor physical condition | Skills |
|  | “ have severe arthritis ….. in my knees and in my shoulders” |  |  |
|  | “Not too many of us are walking without wheelchairs and canes” |  |  |
|  | “[Other patients] have a lot of other disabilities. One of them has diabetes, he’s blind. Another guy has a double amputation.” |  |  |
|  | “I’m not sure we have the stamina to continue with exercising for any length of time……, I know what I should be doing but once again what I should be doing and what I can do are 2 different things. There are people of course that are and can only do certain things” |  |  |
|  | “I usually can only exercise on the days that I don’t go to dialysis, so that really only allows me 2 days a week” | Patient lack of time due to dialysis | Environmental Context and Resources |
|  | “As a result of the dialysis, I have to cram six days of living into three, and so I live alone, so I have to do everything myself” |  |  |
|  | “Because with my schedule right now I’m trying to get a kidney and I have so many appointments to go to and I have so much going on” |  |  |
|  | ...and time constraints due to dialysis were other major dialysis-related barriers |  |  |
|  | “There are days you know where you just don’t feel like doing a whole lot so you just don’t do it. Between being tired and having other physical problems too, it’s tough to get somebody motivated enough to want to try to do it. | Lack of motivation | Intentions |
|  | “I’d stop exercising and then you know when you stop something you get lazy about it and no motivation” |  |  |
|  | As with adopting any lifestyle modification, lack of motivation is one of the biggest challenges – and patients, staff and nephrologists readily recognized this. Patients reported that dealing with physical and mental challenges of having a chronic illness and being on dialysis made exercise a low priority for them. |  |  |
|  | “I have fallen several times because I wasn’t paying attention to what I was doing and I have been lucky so far” | Fear of falling | Beliefs about consequences |
|  | Fear of falling... were some of the other reported barriers |  |  |
|  | “When it’s cold outside, I don’t have as much [exercise/walking]” | Bad weather | Environmental Context and Resources |
|  | lack of suitable exercise options were some of the other reported barriers | Lack of suitable exercise options (general) | Beliefs about consequences |
|  | lack of counselling by staff | Lack of support from health professionals | Social Influences |
| Reported barriers to intra-dialytic exercise: intra-dialytic exercise should be safe without disrupting usual care | limitations due to inability to use the access arm | Physical limitation during dialysis prevents IDE | Environmental Context and |
|  | “Getting it [stationary bike] up to the chair would be a challenge. It would probably be too big and clunky of a device to put in there” |  |  |
|  | “I found that [stationary bike that he used in a past research study] to be very boring, I didn’t like it……….. And I don’t think anybody did because I don’t see anybody doing it now” | Exercise not a priority for nurses | Environmental Context and Resources |
|  | Some of them had prior experience using a stationary pedaling bike during HD and offered reasons for poor adherence - patients felt that the bike was boring |  |  |
|  | Some of them had prior experience using a stationary pedaling bike during HD and offered reasons for poor adherence - patients felt that the bike was boring | Lack of enjoyment/monotonous nature of PA/disliking exercising/finding it boring | Emotion |
|  | Only a few patients stated that exercising in front of others was a significant barrier  “I don’t like to be what I think is doing performing in front of people because I don’t think I would do it well” | Lack of privacy in IDE | Environmental Context and Resources |
|  | All participants felt that extensive staff involvement in any intra-dialytic exercise program would be impractical | IDE as an additional source of pressure | Environmental Context and Resources |
|  | “Yes it would [impact workload] and guarantee they wouldn’t have time to add that to their load. They’re extremely busy the whole time. …so adding one more responsibility to them I don’t think is realistic” |  |  |
|  | “I go [to dialysis] early in the morning so I ain’t gonna be doing no exercise early in the morning I like to sleep during the dialysis mostly” | Exercising in the dialysis centre |  |
|  | “If you did it [exercise in dialysis unit prior to starting HD] none of us want to stay there any longer than we have to….you just don’t want to be there. It’s a mental thing, you just want to get out. |  |  |
|  | An interesting barrier that emerged was patients’ resistance to changing the routine of dialysis. | Lack of motivation | Intentions |
| 11. Wodskou *et al*. (2021) | | | |
| Motivation for Intradialytic Exercise and Perceived Barriers: Perceived Barriers to Intradialytic Exercise | The main barrier to IE was concern about triggering the hemodialysis machine alarm.  Most patients had experienced triggering the machine alarm if they moved their cannulated  arm at all. The concern with the machine alarm seemed to be the alarm itself and not  potential reasons for the alarm, such as changes in blood pressure or dialysis flow. No  patients were concerned about hypotension or cramping resulting from IE. One patient  said,  “No, I get that too when I lie down. Whether you stand or lie down, you can  easily lie down again. So that’s not a problem, is it?” Patients felt that alarms inconvenienced both them and, particularly, the nurses. Several patients also had experienced varying levels of alarm sensitivity over time. They believed that the machine would sometimes allow them to do the exercises but would be set off by the slightest movement at other times, preventing physical activity. A few patients suggested having special exercises that they could do in bed on days when the machine alarmed a lot. As one patient put it: “But then I do it (exercise) on the bed because I just have to move this hand here, and the machine starts to roar. I just tried that, so this is the day when I can’t move that arm. I also must be careful not to bend the tube because then it (the machine) will also scold”. Patients worried about needles and tubes as potential barriers to IE. They noted that physical activity must not accidentally pull out the needles; one cannot be physically active with sharp needles inserted, and one must be careful not to clamp or tangle the tubes. Several patients mentioned the importance of keeping the cannulated arm at rest to protect needles and tubes and avoid triggering the alarm. One patient who was being dialyzed via central venous catheter felt there was no risk of damaging her intravenous access. In addition to alarms, needles, and tubes, the most frequently reported barrier to IE was unwillingness or, as one patient phrased it, “laziness”. Three patients mentioned this, reporting that they were otherwise active or were not interested in “gymnastics”. Two participants who were still working mentioned that they lacked the energy to be physically active during hemodialysis and needed primarily to rest. Finally, patients mentioned fatigue during hemodialysis, feeling “poisoned” due to their renal failure, musculoskeletal pain, the busy workloads of nurses, and concern for fellow patients as potential barriers to intradialytic exercise. | IDE could disrupt dialysis | Beliefs about consequences |
|  |  | IDE could disrupt the routine on the dialysis unit | Environmental Context and Resources |
|  |  | Movement from IDE can set off dialysis alarm | Beliefs about consequences AND Reinforcement |
|  |  | Nurses lack of time due to other tasks | Environmental Context and Resources |
|  |  |  |  |
|  |  | Awareness of different exercise options including those that suited for limited mobility patients | Knowledge |
|  |  | Lack of motivation | Intentions |
|  |  | Lack of interest |  |
|  |  | Fatigue | Skills |
|  |  | Poor physical condition |  |
|  |  | Nurses lack time for IDE due to other tasks | Environmental Context and Resources |
| Motivation for Intradialytic Exercise and Perceived Barriers: Nurses’ Opinions and Approval Are Important | Nurses’ opinions were important to patients’ motivation for IE. A few patients feared  that nurses would be irritated and tell them to get back into bed and lie still if the machine  alarmed during exercise. On the other hand, patients expressed confidence about exercising  if nurses approved, e.g.,  “Interviewer: Is there anything you worry about in relation to standing up and  doing exercises? Patient: Nothing . . . Just that they say it’s best not to get up.  That’s what the nurses say. Interviewer: So, you would feel like standing up if  the nurse approved? Patient: Yes, of course. No problems. Nothing at all”.  Patients disagreed as to whether the nurses should encourage patients to exercise  during dialysis. Most patients thought it could motivate them, while others did not want  nurses to interfere in their decision to exercise or felt that the nurses lacked time to support  IE. | Lack of ‘buy in’ to IDE from staff | Social Influences |
|  |  | Lack of support from health professionals |  |
|  |  | Nurses lack time for IDE due to other tasks | Environmental Context and Resources |
|  |  | Encouragement from staff | Social Influences |
| 15. Huang et al., (2023) | | | |
| Poor physical condition: Symptom burden | Comorbid conditions and symptom burden were described as common features of physical inactivity in people undergoing haemodialyses. Symptoms were considered functional limitations in people undergoing haemodialyses and most commonly included fatigue, pain, and shortness of breath. The muscles may be significantly less. Because I feel that my arms and legs are getting more and more tired, that is, I will have no energy all over my body. (Q1, F)  I was very tired after hemodialyses, and I just wanted to go home and lie down to rest or sleep. (Q2, R) | Poor physical condition | Skills |
|  |  | Fatigue |  |
|  |  | Fear of further fatigue | Beliefs about consequences |
| Poor physical condition: Increasing age and haemodialyses vintage | I feel this way myself, because I have been through it for a long time, um, it will definitely get worse as I go on. (Q3, N)  Besides, patients acknowledged that aging and haemodialyses duration process were unalterable facts and limited the patient's physical function year by year. Middle‐aged and elderly patients feel more strongly. | Belief that age limits capacity | Beliefs about capabilities |
|  |  | Patient belief that no improvement is possible | Optimism |
| Poor physical condition: Physical activity related concerns | Symptoms were considered functional limitations in people undergoing haemodialyses and most commonly included fatigue, pain, and shortness of breath.   Participants also avoided activities involving the arms due to problems such as arteriovenous fistula stenosis.  While I may feel that increasing physical activity is no different from everyday life, my body responds. For example, panting, especially sweating a lot, chest tightness and shortness of breath, I feel that my own breath is not enough for myself. (Q4, F)  Before I started hemodialyses, I swam and played badminton. I never swam after hemodialysis because I was afraid that fistula would be infected by unclean swimming pool. (Q5, D) | Pain during exercise | Reinforcement |
|  |  | Unpleasant physical reactions to exercise |  |
|  |  | Fear of fistula damage | Beliefs about consequences |
| Poor physical condition: Negative comparison | I used to go to the gym to run on the treadmill, but now I don't dare to run on the treadmill, because sometimes the speed is accidentally increased, and I can't keep up. Then I don't want to do any activities. (Q6, P)   In particular, patients who enjoyed moderate‐to‐high‐intensity activity before haemodialyses had the strongest negative contrast, causing their interest in physical activity to plummet. | Exercise options in local gyms are unsuitable for dialysis patients | Environmental Context and Resources |
| Lack of support from family, friends and nephrology staff: Lack of support from family | Patients described accidental injuries were caused by activities overestimating their physical ability.   Failed physical activity experiences created fear of activity in the patient and, more importantly, were the strong reason for the family to discourage the patient from being active.  My family told me to stay at home and not to go out and worry about my accident. And I have diabetes, I'm afraid I'm outside low blood sugar, what if something happens? (Q7, M)  I've now put a burden on my home. Who helps to watch a grandchild while I'm doing hemodialysis treatment? is it? I had previously fallen down the stairs, tripped the hand, fractured the bone, and played a plaster. My wife said to have spared you from getting this arm of the arteriovenous fistula. (Q8, D) | Concern injury and accident | Beliefs about consequences |
|  |  | Fear of falling |  |
|  |  | Other health conditions | Skills |
|  |  | Previous injury | Reinforcement |
|  |  | Direct guidance not to exercise from family | Social Influences |
|  | Going out with friends or colleagues was a good opportunity for patient activity. | Social support from personal network | Social Influences |
| Lack of support from family, friends and nephrology staff: Friends estranged | We have a team playing badminton and swimming, and now they don't dare to call me. They are also afraid because I am not a normal person. There's nothing wrong with being inactive anyway, right? (Q9, D)   However, the excessive sense of responsibility of friends was worried that going out will cause secondary harm to the patients, which gradually reduced the opportunities for the patients to participate in activities. At the same time, it also damaged the social interaction of patients, and the concept of ‘patients are sick and different from normal people’ was deeply rooted in the patients' mind. | Feeling excluded from social exercise opportunities with those who do not require dialysis | Social Influences |
|  |  | Family/friends lack of knowledge of condition(s) |  |
|  |  | Misconceptions about the relationship between physical activity and condition(s) | Knowledge |
| Lack of support from family, friends and nephrology staff: Inconsistent health education help from staff | No one said to me to be active. I don't know it (physical activity) and don't value that. (Q10, N)  The nurse has said that I had better move about, but she simply said a few words. (Q11, A)   Although nephrologists regularly see patients, they have poor health education on physical activities for patients. Some patients said that they never learned this from the staff of the nephrology department, and thought that the staff was too busy to explain the physical activity to patients. At the same time, the patient felt that the staff did not mention physical activity, indicating that it was not important. | Patients lack of knowledge of the benefits | Knowledge |
|  |  | Lack of enough information support for patients |  |
|  |  | Lack of guidance from healthcare professionals | Social Influences |
| Lack of conditions: Physical environment | I won't go out for activities when it rains. When it is too cold, I am afraid of catching a cold. (Q12, B)  Patients described in detail how severe weather hindered their plans to travel outside. | Bad weather | Environmental Context and Resources |
|  |  | Concern exercise may lead to sick | Beliefs about consequences |
| 16. Sheshradi et al. 2020 | | | |
| Barriers | The most common barrier reported prior to the intervention was lack of motivation (30%), followed by initial difficulty using the pedometer (17%) and concerns about safety while walking (7%). | Lack of motivation | Intentions |
|  |  | Hard to use equipment | Environmental Context and Resources |
|  |  | Concern injury and accident | Beliefs about consequences |

| Lack of motivation | ‘I just spent Monday and Tuesday pretty much in bed. Not sure why’. ‘I saw that goal, and I didn’t think I could reach it, so I didn’t think it was worth trying’.  The most common barrier reported prior to the intervention was lack of motivation (30%) | | | Lack of motivation | | Intentions | |  |
| --- | --- | --- | --- | --- | --- | --- | --- | --- |
|  |  |  |  | Patient belief that no improvement is possible | | Optimism | |  |
|  |  |  |  | Goals unattainable so no point trying | | Goals | |  |
| Problems with utilizing pedometer initially | ‘I wear sweatpants to dialysis, and I’ve got no belt loop. When you clip it on, it sags, and then it doesn’t give you the right number’.  ‘It didn’t seem like it was working so I didn’t put it on’.  The most common barrier reported prior to the intervention was lack of motivation (30%), followed by initial difficulty using the pedometer (17%) | | | Hard to use equipment | | Environmental Context and Resources | |  |
| Concern about safety while walking | ‘I’ve been told I need to be more careful when I’m moving around’.  ‘My wife tells me she’s afraid I might fall, so she doesn’t like me to go outside. I’ve had falls before’. | | | Direct guidance not to exercise from family | | Social Influences | |  |
|  |  |  |  | Fear of falling | | Beliefs about consequences | |  |
|  |  |  |  | Previous injury | |  |  |  |
| Health-related barrier | ‘I had to go to the ER, with chest pain again. So that week was a bust’.  ‘It’s time for my colonoscopy and I couldn’t really go anywhere for a while’.  ‘I was getting cataract surgery, so I figured I’d wait’.  The most common barrier reported to study personnel during the intervention was a health-related barrier such as an illness or hospitalization | | | Poor physical condition | | Skills | |  |
|  |  |  |  | Other health conditions | |  |  |  |
|  |  |  |  | Lack of physical ability | |  |  |  |
| Reported symptoms that prevented walking to best of ability | ‘I have chest pain when I walk sometimes, and I have to stop. I don’t want to do more than my body can do – I know my body’.  ‘I just kept having coughing fits. It was hard to breathe, and I didn’t like that’.  ‘I’m wearing a leg brace, but I’ve still got this knee pain’.  Twenty-seven percent of participants also reported extra-dialytic symptoms that affected their ability to walk at their best level, with the most common being dyspnea with exertion, general body soreness and fatigue. | | | Pain (general) | | Skills | |  |
|  |  |  |  | Poor physical condition | |  |  |  |
|  |  |  |  | Unpleasant physical reactions to exercise | | Reinforcement | |  |
|  |  |  |  | Concern injury and accident | | Beliefs about consequences | |  |
|  |  |  |  | Patient belief that condition(s) preclude physical activity | | Beliefs about capabilities | |  |
| Reported barrier specific to dialysis | ‘I’m just too tired after dialysis. It takes me about a day to recover’.  ‘How I feel, it really depends on my blood pressure. If it gets too low – say, lower than 120 – I get lightheaded. Things start to move, and I have to get steady’.  ‘I’ve just got too much fluid on me’.  Dialysis-related barriers to walking were expressed by 27% of participants, including 13% reporting post-dialysis fatigue, 13% inability to remove adequate amounts of fluid during dialysis or bothersome swelling despite dialyzing per prescription, and 3% intradialytic hypotension. | | | Fatigue | | Skills | |  |
|  |  |  |  | Poor physical condition | |  |  |  |
| Weather or other environmental issues | ‘There’s not a lot of places to walk around here, and some of the people, I don’t always feel comfortable walking’.  ‘It’s just been raining all week, and the pavement here is uneven. My wife is worried I might slip and fall. I got this cane, but you know how it is’.  Finally, 13% of participants reported that their ability to walk was adversely affected by the weather. | | | Bad weather | | Environmental Context and Resources | |  |
|  |  |  |  | Local environment risks | |  |  |  |
|  |  |  |  | Concern injury and accident | | Beliefs about consequences | |  |
| 17. Young *et al*. (2015) (pre implementation of IDE) | | | | | | |  |  |
| Staff workload Young *et al*. (2015) | | Staff and patients both expressed concern about a lack of staff resources and busy workloads within the HD environment. All patients were wary of creating extra work for staff and believed that a lack of time would reduce supervision and encouragement during exercise. Staff agreed, and junior staff in particular strongly believed that IDE would increase in their workload. “If [IDE] requires less intervention on [the] part [of the staff] then it’s a good thing but if they’re being called up more frequently then it’s probably a bad thing.” “My concern is the nurses need to be nursing not lifting bikes on and off beds” “The whole thing works better if you have [the nurses] co-operation and rather than increasing their work its in the quieter periods so it works for everybody” | Nurses lack time for IDE due to other tasks | | Environmental Context and Resources | |  |  |
|  |  |  | IDE as an additional source of pressure | |  |  |  |  |
|  |  |  | Exercise not a priority for nurses | |  |  |  |  |
|  |  |  | Lack of time reducing effective delivery of IDE | |  |  |  |  |
|  |  |  | Exercise monitoring/assessment/provision not included in care provision/ not the nurses’ role | | Social/Professional Role and Identity | |  |  |
| Patients fears and  anxieties Young *et al*. (2015) | | You’re a little bit more aware of what damage it could do. You’re a little bit scared of the consequences should anything happen.” “I wonder whether it might affect, any movement, and pull the needles out.” “I am more concerned if I have low blood pressure, and pass out I would be stuck on a chair with a cumbersome bike at the end” “If you are on this bike and you fall ill, there is no doctor [at the satellite unit]. You have got the same chance as Jo Public ringing an ambulance.” | Concern injury and accident | | Beliefs about consequences | |  |  |
|  |  |  | Reduced access to patients in emergency if IDE equipment in the way | |  |  |  |  |
|  |  |  | IDE could disrupt dialysis | |  |  |  |  |
| Staff beliefs about  IDE and patients Young *et al*. (2015) | | “We’re all aware of the need to exercise but its time and inclination, if it’s provided for you when you’re trapped [having dialysis] you can’t avoid it.” | Exercise at dialysis centre/unit | | Environmental Context and Resources | |  |  |
|  |  |  | Not taking up free time | |  |  |  |  |
|  |  | “When it was first mentioned one of the nurses came to me and said they won’t let you do that. I said yes they will, why wouldn’t they? They won’t. And he ... didn’t seem too keen”   This [the views of the staff] directly contrasted with patient views, who despite their initial anxieties, viewed IDE as an opportunity to overcome exercise barriers and a positive use of treatment time. Patients were aware of staff members’ negative perceptions and felt this could dissuade them from participating | Not knowing which patients are eligible | | Knowledge | |  |  |
|  |  |  | Staff belief that patients are not capable of IDE | | Beliefs about capabilities | |  |  |
| Patients’ fears and anxieties Young *et al*. (2015) | | Patients were initially unsure of the consequences of participating in IDE which was described as “going into the unknown” (Pre-implementation focus  group). Patients described a range of emotions about participation including being afraid of  disrupting their treatment, particularly through dislodging their needles and injuring themselves. They were fearful that a cumbersome bike may impact on safety should an emergency  occur. These concerns were particularly evident in patients dialysing at satellite units and those  who had experienced hypotension during treatment. | Not knowing what to expect from exercise | | Knowledge | |  |  |
|  |  |  | Concern injury and accident | | Beliefs about consequences | |  |  |
|  |  |  | Fear of fistula damage | |  |  |  |  |
|  |  |  | Reduced access to patients in emergency if IDE equipment in the way | |  |  |  |  |
|  |  |  | General fears about safety | |  |  |  |  |
|  |  |  | IDE could disrupt dialysis | |  |  |  |  |
| 17b. Young *et al*. (2015) (post implementation of IDE) | | | | | | | | |
| Staff workload Young *et al*. (2015) | | “Well in this situation here and now, we are short of  nurses...if you put an extra load on them about  exercising, I don’t think that’s possible.” (67 year old  male, Asian patient)  “Its getting the nurses to [help] and making sure  they don’t forget. Either they have forgotten or they  are probably shorted staffed.” (57 year old male,  Asian patient)  Seven patients concluded that staff did not prioritize IDE because of their workload, a view confirmed by five staff members.  y. Seven  patients concluded that staff did not prioritize IDE because of their workload, a view confirmed  by five staff members.  Large workloads, lack of time and unpredictable shift patterns also led staff to report difficulty attending IDE training sessions or using the knowledge and skills gained from them. This  view was confirmed by seven patients who expressed frustration that staff were not as adept at  running the programme as exercise professionals because of lack of familiarity. | Patients fear being a burden to staff | | Beliefs about consequences | |  |  |
|  |  |  | Nurses lack time for IDE due to other tasks | | Environmental Context and Resources | |  |  |
|  |  |  | Inconsistent help from dialysis staff for IDE | | Social Influences | |  |  |
| Lack of staff  responsibility Young *et al*. (2015) | | “For a start you will have to have [an exercise  professional] there but once the staff are trained  then its fine. That will reassure [patients] and then  you can get some dedicated staff to do it.” (65 year  old male Asian patient)  Staff and patients believed that there was too much variation  in IDE provision due to a lack of responsibility for the programme amongst staff. Seven patients identified the importance of an exercise professional to the initial implementation of the  programme but also felt that a dedicated staff member (not necessarily an exercise professional) was important to ongoing success. This individual could act as a ‘coach’, providing feedback, encouragement and support. | Additional, exercise specific staff | | Environmental Context and Resources | |  |  |
|  |  |  | Needing staff to have sufficient knowledge to reassure and encourage patients | | Knowledge | |  |  |

# Originally mixed themes

Barriers coded grey; facilitators coded green

| Original barrier  /Study | | Data | New codes (after splitting) | | | TDF domain mapped to |
| --- | --- | --- | --- | --- | --- | --- |
| 3. Sieverdes et al. (2015) | | | | | | |
| Physical Activity Is Optional  Sieverdes et al. (2015) | | “I can do a lot of things. Sometimes it is hard to think straight. I have a mental barrier. I would lose. Reported barriers to physical activity included dialysis, motivation, fatigue, problems with joints, muscle weakness, or pain in lower extremities during physical activity. | Lack of motivation (barrier) | | | Intentions |
|  |  |  | Fatigue (barrier) | | | Skills |
|  |  |  | Lack of physical ability (barrier) | | |  |
|  |  |  | Pain during exercise (barrier) | | | Reinforcement |
|  |  | Physical activity was perceived as optional and good to do, but not necessary for long-term health goals. | Physical activity is optional (barrier) | | | Goals |
|  |  | No participants reported associations or connections of physical activity with the long-term anticipated health outcomes of receiving a kidney transplant. Physical activity was perceived as optional and good to do, but not necessary for long-term health goals. | Belief exercise is not necessary for long-term health goals (barrier) | | | Goals, Beliefs about consequences |
|  |  | Most stated that they would not be interested in exercising at their dialysis center and preferred a plan around exercising on nondialysis days. | Exercising in the dialysis centre (barrier) | | | Environmental context and Resources |
|  |  | The overwhelming majority indicated that they would like to exercise alone or with a close relative or significant other who could help motivate them to do more. | Exercising alone (facilitator) | | | Environmental context and Resources |
|  |  | Perceived benefits of physical activity included weight loss, an effective way to cope with stress, and improved self-image. In general it’s more about the look for me and feeling good about how I look. I’d like a flat stomach and feel better about myself.”, More about self-identify: “I guess my central concern lately is the amount of weight that I have put on…” | Perceived benefit to physical well-being | | | Beliefs about consequences |
|  |  |  | Coping with stress (facilitator) | | |  |
|  |  |  | Improved self-image (facilitator) | | |  |
|  |  |  | Weight-loss (facilitator) | | |  |
|  |  | The overwhelming majority indicated that they would like to exercise alone or with a close relative or significant other who could help motivate them to do more. | Social support from personal network (facilitator) | | | Social Influences |
|  |  |  | The importance of patient’s family | | |  |
|  |  | Seventy-three percent responded that they used walking or bicycling at a moderate intensity between 15 and 60 minutes per session several times per week | Active travel (facilitator) | | | Environmental Context and Resources |
|  | | | | | | |
| Social support goes both ways  Sieverdes et al. (2015) | | Identified behaviors supportive of physical activity by social networks included modeling physical activity and encouraging them to join in, scheduling activities that involved physical activity with the participants, or verbally encouraging the participants to become more physically active. Social system as support: “My mom and I used to go to the gym together but now we don’t because her work schedule changed. We used to ride bikes together in her subdivision but once again we don’t anymore because of the schedule change. They are always encouraging me to get up and be active. My mom always says that working out helps with your self-esteem. . . . | Social support from personal network (facilitator) | | | Social Influences |
|  |  |  | Importance of patient’s family (facilitator) | | |  |
|  |  | Identified barriers to physical activity included friends and family members not modeling exercise behaviors and not encouraging the participants to exercise. I have friends but they don’t want to work out either.”, Social system as barrier: “My friends are in the same boat. Instead of encouraging each other we just say, ‘You know how it is.’ Then we will come up with our excuses for not losing weight or joining a program.” | Friends/Family lack of physical activity (barrier) | | | Social Influences |
|  |  |  | Lack of support from social network (barrier) | | |  |
|  | |  |  | | |  |
| Chronic stress of living and finding the means to persevere  Sieverdes et al. (2015) | | Sources of stress for these participants included unemployment, disability, financial constraints, not having a support system, dialysis days, caregiving role (eg, taking care of parents, children), not having time to do previously enjoyed activities because of scheduled dialysis, waiting on a donor, and negative effects of illness including death  The participants discussed using a variety of coping strategies...The subthemes of hopefulness, and self-efficacy (ie, sense of being able to engage in a program for self-management of end-stage renal disease including physical activity) began to emerge ...as they described their stress and ways that they were managing the stressors of their daily lives. “Usually I am pretty relaxed. I need to incorporate lifestyle changes that produce some beneficial results like concentrating on exercise programs and lower calorie food preparations . . . just because I am on dialysis it doesn’t mean I can’t do other things, because I can certainly do plenty more than I do now.”, “I try to think positively. I was hard headed at one time but now I am more positive and take it 1 day at a time.”, |  | | |  |
|  |  |  | Feeling of taking an active role in their care | | | Behavioural Regulation AND Intentions |
|  | | | | | | |
| Spirituality as a strength and connectedness  Sieverdes et al. (2015) | | The majority of participants (n=18, 82%) discussed the positive benefits mentally, spiritually, and physically from their belief in God and from the social support of their religious communities. Benefits include feelings of hopefulness, strength, motivation to continue living, connectedness (sense of belonging), and the role of faith in being able to cope with their chronic illness and while waiting with uncertainty of whether they will receive a kidney transplant. | *Not coded as not specific to physical activity* | | |  |
|  | | | | | | |
| Technology  Sieverdes et al. (2015) | | Participants perceived that technology may be useful in increasing their awareness of eating patterns and how much physical activity they were doing (ie, self-monitoring). “I would try it out. It would give me more knowledge about what how I am doing and would give me the ability to track what I am doing. I’m not exactly health conscious and it might change my attitude once I had more information to go on.”, Participants rated their comfort level with technology in general and the potential benefits of incorporating technology in their lifestyle to measure and improve their health. Participants perceived that technology may be useful in increasing their awareness of eating patterns and how much physical activity they were doing (ie, self-monitoring). Many implied that self-monitoring may lead to increased physical activity and spoke about how it could affect their confidence in managing their condition. | *Not coded as not specific to physical activity* | | |  |
| 4. Thompson et al. (2016) | | | | | | |
| Theme 1 Support (patients) subtheme: Encouragement from dialysis staff | | Participants identified the staff and the kinesiologist as the main sources of support during the study. Several patients expressed that the staff encouraged their participation in IDE typically through simple words of encouragement (Q5 and Q6). One participant could not define how support had been conveyed to her, but the staff’s reaction to IDE had given her a sense of esteem (Q7) well, the nurses actually encouraged then [when the study staff were not there]; they were the ones that said, ‘Go faster __________ !’…So just the encouragement, probably…it was really good; it was helpful.” Q5 “…they were cheering us along—well, really, I can speak for me—they would be cheering me along and giving me compliments and just encouraging me, telling me how well I’m doing, and telling me they see a change in me.” Q6 “I think—I felt that the nurses were impressed; that’s one feeling that I got. I don’t know for sure, but that’s one feeling that I got, that they were impressed that we were doing this.” | Encouragement from  staff (facilitator) | | | Reinforcement |
| Theme 1 Support Subtheme: Inconsistent help from dialysis staff | | It was more common for patients to comment on the inconsistency of the staff’s involvement. Many participants described lack of support in the form of inconsistent help with the exercise equipment (Q8); several participants attributed this variability to the nurse (rather than situational factors; Q9). For some patients, the staff were perceived as inaccessible for help (Q10). Another participant expressed frustration with the staff’s lack of accountability, explaining that asking for equipment from particular staff members was such a “struggle” that he did not participate in IDE when those staff members were working (Q11).  “I know the nurses don’t like doing it. They don’t ask you and they don’t remind you, ‘Are you going to do your exercises?’ Some do, some don’t.” Q8  “It depends on the nurse you have. Some days it will be problematic [getting help with the equipment], other days, it’ll be just fine. Depends on who your nurse is that day.” Q9  “Oh, I was totally motivated, but again, it was the struggle of Oh, well, you know, I guess maybe I won’t be doing it because I just don’t feel like asking this particular nurse. Then I don’t want to ask, let’s say, [person D], who’s not my nurse, ‘Can you get it for me?’ You know what I mean? ‘Cause the first reaction is, ‘Well, who’s your nurse? How come you didn’t ask her?’” Q11 | Inconsistent help from dialysis staff for IDE (barrier) | | | Social Influences |
|  |  |  | Lack of support from healthcare professionals | | |  |
|  |  | “Well, unless you’re willing to ring the emergency bell and get them to come over just to ask for your exercise equipment, you’re practically waiting for one of them to walk around.” Q10 | IDE could disrupt the routine on the dialysis unit | | | Environmental Context and Resources |
| Theme 1 Support: subtheme Increased body confidence and sense of capability through technical instruction | | Patients commonly viewed the kinesiologist as the primary source of support for IDE. Some participants perceived support from the kinesiologist in the form of technical instruction and trusted her expertise and knowledge (Q12). For most patients, the kinesiologist’s technical instruction was interpreted as having emotional meaning. Patients expressed that they gained confidence in their physical capabilities from training with the kinesiologist. The caring and esteem conveyed in the actions of the exercise specialist enhanced patients’ body confidence, sense of capability, and feeling like an individual  “She helped me with what level I should go to and what I could handle, and that way, I felt very good about that.” Q15 | Instructor providing increased confidence and capability  (facilitator) | | | Beliefs about capabilities |
|  |  |  | Tailored physical activity (by healthcare professional) (facilitator) | | | Social Influences |
|  |  |  | Recommendations of healthcare professionals (facilitator) | | |  |
|  |  | “She, you know, puts everything on and makes sure that I’m doing it properly. And that’s good, too, because you can hurt yourself if you don’t do it properly.” Q12 | Group exercise ensures safety (facilitator) | | | Social Influences |
|  |  | “She was so encouraging that it makes you want to do it. I found I could do more than I thought I could.” Q14 | Encouragement from staff (facilitator) | | |  |
|  |  | “I’m going to continue on my own, because you [the exercise program] already gave me the tools to work with and I already could see what it does to my life and to my personal life, my personal self, my health life—I see what it does for me.” Q13 | Previous good experience of rehab support (facilitator) | | | Reinforcement |
|  |  |  | Observing/feeling the benefits (facilitator) | | |  |
|  |  |  | Choice withinactivity | | | Behavioural regulation |
|  |  | Some participants perceived support from the kinesiologist in the form of technical instruction and trusted her expertise and knowledge | Needing staff to have practical knowledge of how to facilitate and support IDE (facilitator) | | | Knowledge |
| Theme 2: The role of the dialysis nurse – IDE is the nurse’s role AND the influence of personal values about exercise | | “It depends on the nurse you have. Some days, it’ll be problematic, other days, it’ll be just fine. Depends on who is your nurse that day.” | Inconsistent help from  dialysis staff for IDE (barrier) | | | Social Influences |
|  |  | Because patients commonly viewed IDE as beneficial, many expressed that staff involvement in IDE was consistent with their role as caregiver and advocate (Q22). Patients described the staff’s role as providing encouragement and assistance with the equipment (Q22 and Q23). Most patients were aware that the staff saw IDE as “extra work”; however, many patients believed that staff participation in IDE was feasible (Q23 and Q24). One patient expressed resignation about the situation, because he viewed systemic factors as a limitation to their involvement (Q25); other patients viewed staff involvement as nurse dependent (Q26). Several patients viewed the more physically active staff as more interested in participating in IDE (Q27).  “Yeah, pretty much it’s the staff who will be doing it hands on, like, because I don’t know if they’re [the study staff] going to be here for, let’s say, the whole time for that study or not, it just falls to the nurses who’s also doing the things that they have to do. Know what I mean? “But I was also kind of disappointed that they weren’t more enthusiastic about having the patients maybe do a task, enjoy their task, occupy their time more, and to have a benefit to the patient…That’s what—that kind of wasn’t—didn’t sit well with me necessarily, that that they should be willing to do everything for the patient…” Q22  “I mean, even if I’m done my leg exercises and I’m sitting there with 5 pounds of weight on each ankle, I still need someone to undo that, get the bike, get it set up, and ready to go for the next thing. And you’re busy or [person A]’s busy—whoever’s there—so the nurses could handle that job quite easily.” Q23  “I think that they should realize that exercise is important for us people, and that they should maybe show a little more enthusiasm towards us doing some exercise. But I know that they’re overworked and understaffed, so what can you say?” “It’s one more job for them. I’ve heard from other nurses that, ‘Oh, this is—why do we have to do this?’” | Lack of ‘buy in’ to IDE from staff (barrier) | | |  |
|  |  |  | Staff appear too busy to help with IDE (barrier) | | |  |
|  |  | …She [the nurse] would stop and chat about the stuff and she’d get a rubber band and do some exercises, too…You know, because she exercises a lot herself, right? | The influence of personal values  about exercise (facilitator) | | | Social Influences |
|  |  |  | Encouragement from staff (facilitator) | | |  |
| 5. Sutherland et al. (2021) | | | | | | |
| Perceived benefits of increased PA  Mixed views on the benefits of PA for dialysis patients.  Sutherland et al. (2021) | | Many participants were aware of the benefits of PA in general, commenting that they had enjoyed PA prior to their illness and that it was important to keep active in order to stay well and maintain their independence. However, nine (45%) participants (five females, age range 35–73, and four males, age between 36 and 84) found difficulty in identifying benefits that might arise from increasing PA and some expressed the view that PA offered little or no benefit for patients on dialysis. I don’t think it [PA] would make any difference… …You’re limited in what you can do. You know you are coming here for treatment  basically. | Knowledge about the benefits of physical activity (facilitator) | | | Knowledge |
|  |  |  | Enjoyment (facilitator) | | | Reinforcement /Emotion |
|  |  |  | Perceived benefit to physical well-being (facilitator) | | | Beliefs about consequences |
|  |  |  | Maintain independence (facilitator) | | | Beliefs about consequences |
|  |  |  | Patients lack of knowledge of the benefits (barrier) | | | Knowledge |
|  |  |  | Misconceptions about the relationship between physical activity and condition(s) (barrier) | | | Knowledge |
|  |  |  | Lack of physical ability (barrier) | | | Skills |
| 10. Hu et al. (2024) | | | | | | |
| Theme 4: future improvements and recommendations for the VR rehabilitation system: Obstacles and challenges | | For some MHD patients, the timely feedback setting of VR allows them to see their scores and rankings in real time, but sometimes patients also get frustrated during the training process because of the failure of the game level caused by their mistakes, but they still express their willingness to try again. At the same time, due to the strong sensory stimulation, patients invest a lot of energy to focus on the content of the VR training, which can lead to fatigue at the end of the training. P1: “The last time I was in one of the levels in the touch cubes, I kept blundering and choosing the wrong color of the cubes and ended up at the bottom of the rankings, and it would feel like frustration arose at that time, but I didn’t end up not giving up, and ended up clearing the level as well.” P2: “Of all the games I like the badminton one the best, but it gets to the later levels when it the ball gets faster and faster and I have to get very involved so it creates a certain amount of fatigue at the end of it.” P6: “I don’t really like the boxing game in it, it doesn’t feel very suitable for women to play and the font of the game in it is not very recognizable in traditional Chinese.” | Frustration of failure | | | Emotion |
|  |  |  | Fatigue after exercise | | | Reinforcement |
|  |  |  | Exercise options don’t fit cultural/social role | | | Social/Professional Role and Identity |
|  |  |  | IDE equipment not suitable for everyone | | | Environmental Context and Resources |
|  |  |  | Hard to use equipment | | |  |
| Theme 4: future improvements and recommendations for the VR rehabilitation system: Expect a better personalized training program | | At present, most of the VR equipment is for commercial use, the main target group is the healthy population, and there are very few VR rehabilitation and training equipment for the MHD group[15]. Therefore he simulates the physical activity level of mostly healthy people, and its application in the MHD community would make many people uncomfortable with its intensity and frequency of training. P4: “At the beginning when I adjusted the difficulty are medium, and then in the process of training found that it is more strenuous for me, the back of the adjustment to low, and slowly only adapt.” P5: “I don’t really like the fact that the back-ground music in this is all in English, if it was changed to my liking I might be able to accept him more.” P10: “At first it was still difficult to play all these games, so I did it more slowly, if I could adjust it to suit me, it would make my training more effective.” | Poor physical condition | | | Skills |
|  |  |  | Being able to regulate own workload | | | Behavioural Regulation  AND  Environmental Context and Resources |
|  |  |  | Ability to personalise activity to match personal preferences | | | Social/Professional Role and Identity |
| 13. Castillo *et al*. (2021) | | | | | | |
| Knowledge, skills and expectations:  Risks and benefits for staff and patients  Most believed IDE was associated with a variety of health benefits  A few expressed doubts over benefits  Many raised concerns over risk of injury | | ‘So, I say you get more energy, more flexibility. Your legs move better, you know. You can go up the stair without feeling like you climb a mountain, you know’. – Marlena, patient  ‘Because when you’re on the machine you get very sick and you get lightheaded and your blood pressure drops and, you know, it’s not easy, trust me, it’s not an easy thing. Yeah it’s very rough. Yeah so I wouldn’t want to take a chance’. – Sandra, patient  ‘Yeah the only issue that I see with that is in the off chance of an emergency, you know, your blood pressure bottoms out or because you’re pedalling and everything your heart rate will be a little bit higher, right’. – Ian, patient | Improvement in energy levels | | | Reinforcement |
|  |  |  | Perceived benefit to physical well-being | | | Beliefs about Consequences |
|  |  |  | Concern injury and accident | | | Beliefs about Consequences |
|  |  |  | Poor physical condition | | | Skills |
|  |  |  | Doubts about benefits | | | Beliefs about Consequences |
| Knowledge, skills and expectations:  Assumptions about patient eligibility | | ‘It depends on their health state and depends on their age too. Age matters if you are very old, some people are very old there so I don’t believe those people can do the exercise’. – John, patient  ‘Sounds great but I think the crowd that I’m with, a lot of them come in walkers. Some come in the little carts so, in my personal view, if you got 20% of us you did very well’. – Robert Redford, patient | Not knowing which patients are eligible | | | Knowledge |
|  |  |  | Poor physical condition | | | Skills |
|  |  |  | Belief that age limits capacity | | | Beliefs about capabilities |
| Knowledge, skills and expectations:  Assumptions about patient interest  Staff and patients believed few eligible patients would be interested in IDE | | ‘So you’re coming in there with an exercise machine into an environment that is not used to it you’re gonna have a very big uphill battle. For me personally it’s wonderful, but for my surrounding people, my peers, I think you’re gonna have a terrible time’. – Callie, patient  ‘I don’t think there’s anybody that would like to bother them about exercise…’. – Jennifer, patient | Lack of motivation | | | Intentions |
| Knowledge, skills and expectations:  Identifying potential candidates  Some patients preferred to be approached by staff | ‘If they have someone that would ask you if you want to do exercise rather than waiting for you to ask them, it might motivate more people’. – Lola, patient | | | Staff rely on patients to self-refer to take part in exercise | Environmental Context and Resources | |
| Category 2: human, material and logistical resources  Concerns about workload  Participants believed an IDE intervention would significantly increase staff workload and would be difficult to prioritize  Perceptions of workload may be influenced by past experiences  Many believed nursing staff would be resistant to more work  A few believed workload may lessen | ‘…so they’re like all over the place trying to cover each other and stuff like that. So I’m just wondering how that’s gonna affect somebody coming in to just stay with me while I do those exercises…’. – Guinea Pig, patient  ‘The other thought though is the impact on the staff. Because someone on the staff has to bring the bike put it in place, move it away, and depending on what the mechanics and logistics are that’s an added workload for the staff and that’s got to be factored in for sure’. – Sheldon, patient | | | Nurses lack time for IDE due to other tasks | Environmental Context and Resources | |
| Category 2: human, material and logistical resources  Need for exercise professionals  Many participants believed exercise professionals should take responsibility for IDE to address workload concerns | ‘Well the advantage of the physiotherapist was guidance’. – John Doe, patient | | | Supervision by healthcare professional | Environmental Context and Resources | |
| Category 2: human, material and logistical resources:  Space and equipment  Units differ in space requirements for bike storage and movement  Biking equipment must fit dialysis chair/beds and be easy to move, use, maintain and clean | ‘I’ve been back on dialysis for over two years and there’s nothing… nobody seems to know where there’s a bike’. – Marie, patient | | | Lack of practical knowledge to support IDE | Knowledge | |
| Category 3: social dynamics of the unit  Champions    Champions are important enablers of IDE    Nephrologists were believed to be instrumental in supporting practice changes    Nurse champions were seen as necessary for supporting practice changes at the bedside and encouraging patients to cycle, though many suggested it would be difficult to recruit nurse champions    Patient champions were believed to encourage other patients, though not all patients would welcome a peer champion | ‘Just maybe the staff pushing a little bit more, you know, just trying to talk you into it without being too pushy. You know, just reminding you that the equipment’s there and how good you felt when you were on it’. – Bandit, patient    ‘Well it can only be a positive thing, because you have a champion helping you, encouraging you that’s got to be a positive thing’. – Susy, patient    ‘I think the staff going to patients and saying that we think…the cycling program is for you and it will help you. Patients would react to that a certain degree more so than if oh these three people are involved…’. – Timbo Slambo, patient    ‘Speaking only for myself, I think I’d find it an unnecessary pressure’. – Sheldon, patient | | | Encouragement from staff | Social Influences | |
|  |  |  |  | Social support |  |  |
|  |  |  |  | Encouragement from patient peers |  |  |
|  |  |  |  | Inspiration from peers |  |  |
|  |  |  |  | Recommendations of healthcare professionals |  |  |
|  |  |  |  | Physician involvement |  |  |
|  |  |  |  | Exercise champions |  |  |
|  |  |  |  | Additional source of pressure for patients | Belief about capabilities | |
| Category 3: social dynamics of the unit  Patient stories to ignite motivation    Patient stories and experiences are highly influential and can motivate staff and patients to engage in IDE | ‘This lady in the article, she was the one who was biking back then. I don’t know what year that was when her picture was taken…. And she was in there and I thought if she can do it I can do it’. – Canadian Kidney Girl, patient | | | Inspiration from peers | Social Influences | |
| 14. Rothpletz-Puglia *et al*. (2022)* *most of the data was general around the experience of dialysis. Only coded where specifically linked to physical activity. | | | | | | |
| Sporadic Activity Patterns | Barriers to exercise included sleep disruptions, arthritis, and a dislike of exercise. Sleep disruption was the most common reason for lack of activity. Most participants described arising very early in the morning, sometimes at 3 or 4 AM, to get ready and wait for transportation to dialysis. They do not adjust this pattern on a nondialysis treatment day. Many people also talked about sleep difficulty, and several mentioned only getting a few hours of sleep at night and then napping throughout the day. | | | Fatigue | Skills | |
|  |  |  |  | Lack of enjoyment/monotonous nature of PA/disliking exercising/finding it boring | Emotion | |
|  |  |  |  | Poor physical condition | Skills | |
|  |  |  |  | Other health conditions | Skills | |
|  | Other participants relayed that exercise made them feel better when they did it, and one participant described trying to exercise so that he would be eligible for a kidney transplant. | | | Observing/feeling the benefits | Reinforcement | |
|  |  |  |  | Aiming to be healthy for transplant | Goal | |

## Splitting of existing themes - Staff Data

**Data analysed from written text not just quotations*
Papers including staff perspectives:*

## Facilitators Staff Where barriers are found in theme categorised as facilitators, they are highlighted in grey.

| Original barrier/Study | Data | | | | | New barriers (after splitting) | | TDF domain mapped to | | |  |  |
| --- | --- | --- | --- | --- | --- | --- | --- | --- | --- | --- | --- | --- |
| 2. Kontos et al. (2007) | | | | | | | | | | |  |  |
| Formal incorporation of exercise into the treatment  plan  Kontos et al. (2007) | When asked what would be required to facilitate patient engagement in exercise, staff overwhelmingly pointed to the need for change in the medical culture where no priority is accorded to exercise for wellness  in the treatment plans for older hemodialysis patients. Rehabilitation is prescribed for acute conditions but the absence of physicians’ recommendation that patients participate in regular exercise as part of their health promotion poses a barrier to patients’ participation in, and nurses’ encouragement of, exercise. “I think you need to have at the outset, when you’re first told that you have kidney disease, I think there needs to be a whole education program including nutrition, exercise, what’s going to happen to you, and all the options that are open to you. Exercise is never even discussed but if it were discussed that would certainly motivate me.” “Exercise can be introduced. They already comply with the diet and fluid restrictions because they have to. So exercise should also be something they have to do. This has to be part of their education and once they get involved in their own treatment, in the same way that they are already with diet and fluid, I believe they will get the motivation and they will continue. And if it were part of their treatment, it would be part of what we do too.”, “You can measure their blood pressure, you can see that their medications are working or not working, you can check their hemoglobin to see if they are anemic, but exercise is just a qualitative thing whether you feel good or not and I don’t think that is a high priority” | | | | | Change in medical culture | | Behavioural regulation | | |  |  |
|  |  |  |  |  |  | Lack of guidance for patients from healthcare professionals | | Social influences | | |  |  |
|  |  |  |  |  |  | Exercise monitoring/assessment/provision not included in care provision/ not the nurses’ role | | Social/Professional Role and Identity, | | |  |  |
|  |  |  |  |  |  | Lack of enough information support for patients | | Social influences | | |  |  |
|  |  |  |  |  |  | Lack of information sources for patients and staff | | Knowledge | | |  |  |
|  |  |  |  |  |  | Education programme/package for patients | | Knowledge | | |  |  |
|  |  |  |  |  |  | Exercise as part of routine care | | Social/Professional Role and Identity, AND intention AND Skills | | |  |  |
|  |  |  |  |  |  | Staff lack of knowledge of the benefits of physical activity | | Knowledge | | |  |  |
| 4. Thompson *et al*. (2016) | | | | | | | | | | |  |  |
| Patients getting their own equipment  Thompson et al. (2016) | “Well, my thing is not that I would not want patients to not do the exercise program,  but again, if they were going to be taking away the kinesiologist and they would  want to just implement the program in general, I would really cater it more to the  independent patient that could grab their supplies for themselves and record  their own blood pressures and things like that for further study, versus that being  the nurse’s job, because sometimes if there’s an acute situation, again, the patients  are stuck in the chair and there’s nothing they can do, but where as if they come in  and got their own supplies, there are still things that they can do, regardless of  whether the nurse is there.” “to at least make an effort in getting their own supplies, like the weights or the bikes, and if they need help, to ask us.” There was agreement among dialysis staff that IDE would be more sustainable if patients set up their own exercise equipment (located in the unit) before treatment. Although several staff expressed that they could help frailer patients with their equipment, other staff commented that this was not feasible (Q38). Getting one’s own equipment was valued for “saving [staff] time.” More commonly, this task was valued as a sign of the patients taking responsibility for their care | | | | | When patients can retrieve their own equipment for IDE and monitor themselves/low reliance on staff | | Environmental Context and Resources | | |  |  |
|  |  |  |  |  |  | Patients taking responsibility for their own care | | Intentions | | |  |  |
|  | “Good patient education. I think that that would be number one [for the sustainability of the program], is really strong patient education, that they are doing this for their benefit, that this is what benefits them, and that they are responsible ...But I think number one is it’s just so key that it’s patient education, that they understand it’s their responsibility.” | | | | | Patient education on benefits of exercise is key | | Knowledge | | |  |  |
|  | Getting one’s own equipment was valued for “saving [staff] time.” More commonly, this task was valued as a sign of the patients taking responsibility for their care | | | | | Feeling of taking an active role in their care | | Intentions | | |  |  |
| 8. Jhamb et al. (2016) | | | | | | | | | | |  |  |
| Knowledge and perceived benefits of exercise Jhamb et al. (2016) | “ I think it would help as far as depression goes, you know, I think exercise has definitely been shown to lift the mood, elevate your mood.” | | | | | Knowledge about the benefits of physical activity | | Knowledge | | |  |  |
|  | “They will have a lot of energy, more energy. That would be a major benefit”   “ Improving the metabolism …… I think that an improved metabolism could actually help with their nutritional status.” | | | | | Perceived benefit to physical well-being | | Beliefs about consequences | | |  |  |
|  | “ I just think it might change their whole attitude, like to a more healthier, more compliant attitude, if they start exercising.” | | | | | Perceived benefit to mental well-being | | Beliefs about consequences | | |  |  |
|  | “Maybe a mental health benefit that they’re doing something productive rather than just whiling away time in the dialysis chair” | | | | |  |  |  |  |  |  |  |
|  | “[Exercise during HD would] bring their blood pressure up while they were on the machines, and actually it would hopefully help them lose a little bit more water-weight, if they broke out a little sweat while they were on the machines.” | | | | | Belief that IDE could enhance dialysis treatment | | Beliefs about consequences | | |  |  |
|  | “Improved cardiac health, improved cardiac output. For some of our diabetic patients and patients with vascular disease I would think that even some simple exercises would help to improve their vascular health” | | | | | Knowledge about the benefits of physical activity | | Knowledge | | |  |  |
|  | “I like to keep people moving, the more you move, the more the muscle grows back, the less likely it is for you to have atrophy problems and then its gonna eventually impact your ADLs [activities of daily living]” | | | | | Knowledge about the consequences of lack of physical activity | | Knowledge | | |  |  |
|  | We found that ... surprisingly one staff member (dialysis technician, 28 yo) reported no knowledge of benefits of exercise. | | | | | Staff lack of knowledge of the benefits of physical activity | | Knowledge | | |  |  |
| Theme 4: Motivation for exercise comes from within and from the  encouragement of others | “I’m a huge proponent [of exercise], if you’re able, I wanna encourage patients to do whatever that I believe and they state is their ability level.” | | | | | Perceived benefit to physical well-being | | Beliefs about consequences | | |  |  |
|  | “I believe in the use it or lose it mentality. You know whether it’s tough for them to workout sometimes, it’s almost like you have to kinda push through it to reap the benefits… I think there may be …. some form of exercise that almost everyone can do. I definitely think that exercise is important in any way they can fit it in” | | | | |  |  |  |  |  |  |  |
|  | “Maybe the end goal of improving their energy, improving how they feel after dialysis, any beneficial factors….like I said energy or stress” | | | | | Patients having intrinsic goals | | Goals | | |  |  |
|  | “Some of our patients are motivated by health goals, it doesn’t just have to be transplant, if they wrestle with something else health wise and they’re on a path for improvement their own health for some can be motivating as well.” | | | | |  |  |  |  |  |  |  |
|  | “The support from the medical practitioners would be critical, I mean these are the same people interacting with them about all aspects of their life and their goals” | | | | | Recommendations of healthcare professionals | | Social Influences | | |  |  |
|  | “Most have a spouse or a sibling or child or parent that’s working with them in part of their lives. That person’s buy in could be motivating” | | | | | Importance of patient’s family | | Social Influences | | |  |  |
| Theme 5: Recommendations for intra-dialytic exercise (Jhamb *et al*. 2016) | A dialysis unit administrator described his experience with a pilot group physical therapy program in his unit involving an in-center physical therapist: “it was well tolerated, it was well accepted … was extremely positive from patients. The anecdotal patients’ feedback was overwhelming, not just one or two, essentially all patients seemed to very much appreciate the attention, the diversion…. the patient response one after another after another totally was “This is fun, I like it, I love the person, I look forward to it”. From our measurements of patient perception, participation, and their feedback, it was a wonderful success.” | | | | | Distraction from treatment | | Emotion | | |  |  |
|  | “trying something new in a very routine and monotonous process that they live with every week. It may end up that something like that is motivating because it’s different” | | | | |  |  |  |  |  |  |  |
|  | “some people get pretty bored…..but if they were able to do some exercise, that would be a way to spend the time” | | | | |  |  |  |  |  |  |  |
|  | “They would see how much fun other people were having doing it, and if the staff gets into it, just walking around and doing their work, they’ll be playing around with it, and it just makes for a very light and fun afternoon, time will go fast.” | | | | | Encouragement from peers | | Social Influences | | |  |  |
|  |  |  |  |  |  | Camaraderie and normalcy in the Unit | |  | | | | |
|  | “The convenience too, if they were here for 3–4 h, you know” | | | | | Exercise at dialysis centre/unit | | Environmental Context and Resources | | |  |  |
|  | “You’re not doing it outside of dialysis which is precious time to our patients” | | | | | Not taking up free time | |  |  |  |  |  |
|  | “It’s that mentality if you see someone else trying to improve themselves and then you kind of like feel bad about yourself …….. And then maybe if they are seeing their friends across the aisle doing it, then that might motivate them to do it as well.” | | | | | Fostering a positive common identity as patients who exercise | | Social Influences | | |  |  |
|  | “Group activities where you see other people enjoying it and you feel like you want to participate” | | | | |  |  |  |  |  |  |  |
|  | “Being involved in group activities not necessarily them being pin-pointed you know all by themselves?” | | | | |  |  |  |  |  |  |  |
|  | “Most of the staff I think would encourage the patients and have fun with it and help the patient have fun with it.” | | | | | Patient encouragement from staff | |  |  |  |  |  |
|  | “Have a spirited person to try to engage them, and just kind of make it fun.” | | | | |  |  |  |  |  |  |  |
|  | “I think what would be best initially is just to do some pretty heavy education on the benefits. You know especially kind of hitting home the role of exercise with giving them energy… you need to get stronger so you can live on your own again leave the nursing home, get back to home and this is something that can help you reach those goals.” | | | | | Patient education on benefits of exercise is key | | Knowledge | | |  |  |
|  | “Maybe testimonials [from patients about benefits of exercise] ….if they could see that” | | | | | Positive beliefs about physical activity (long-term benefits) | | Belief about Consequences | | |  |  |
|  | “It would be every few months, do some sort of contest or raffle or something they could earn if they do their exercise,[like we did for fluid gains]” | | | | | Patients having extrinsic goals | | Goals | | |  |  |
|  | “I’m sure that [gift cards] would be incentive but that’s costly. Everybody likes money!” | | | | |  |  |  |  |  |  |  |
| Theme 6: Dialysis staff attitude towards promoting exercise Jhamb *et al. (2016)* | “There’s clearly a significant population of patients who could participate in some kind of planned exercise during a dialysis treatment, I know that to be possible. I know it can be done and that it can be tolerated” | | | | | Knowledge about the benefits of physical activity | | Knowledge | | |  |  |
|  |  |  |  |  |  | Staff knowing which patients are safe to take part in IDE and where IDE is contraindicated | |  |  |  |  |  |
|  | they [staff] strongly felt that their direct responsibility should be limited to patient encouragement, motivation and monitoring. Staff felt that a program that required minimal staff assistance could be easily accommodated and would not add to their workload “I don’t see it [encouraging exercising] being a huge issue …. monthly education with them on that [exercise] like I do with the diet and you know adding a little bit of work but I don’t think it would cumbersome.” [Interview 19, 35 yo, dietitian] “I don’t know if that would, you know, interfere with us making sure that they’re okay ‘cause we do every half hour check-ups basically we’re making our rounds to every patient, making sure that they’re okay, checking all of their vitals.…….. I wouldn’t see it [exercise during dialysis] being that much of a hassle.” | | | | | When patients can retrieve their own equipment for IDE and monitor themselves/low reliance on staff | | Environmental Context and Resources | | |  |  |
| 9. Painter *et al.* 2014 | | | | | | | | | | | | |
| Theme 2: Benefits of physical activity | | Staff were unanimous in extolling the benefits of physical activity for everyone, including their patients. Specific examples of patients benefitting from regular exercise were given. “Physical activity improves a lot of lives, you know, so I don’t see why this would be any different”. “I mean you may not have working kidneys but you should be able to be active. Otherwise you’re just kind of stagnant and don’t do anything. I think some patients just kind of you know, ‘I’m on dialysis, I don’t ever really feel good.’ But I think if they got up and moving and actually had a little bit more physical activity they might feel a lot better than if they just kind of sit there and let the sickness take over their life.” | | | Perceived benefit to physical well-being | | | | Beliefs about consequences | | | |
|  |  | We didn’t think one of our younger kids was going to pull through. He ended up coming out of it. I think his turning point was he got a dog, and then after he got a dog, then he was like ‘I’ve got to take him for a walk.’ I’d see him out walking around in the neighborhood.” | | | Previous good experience of rehab support | | | | Reinforcement | | | |
|  |  | “Taking care of a dialysis patient when they’re more able to do things on their own means they’re a much easier patient to take care of. Here and at home. They feel better, they’re happier, they’re not needy. You know, [imitates whining tone] ‘wipe my chin’.” | | | Functionally able patients are easier for staff to manage | | | | Beliefs about consequences | | | |
|  |  | However, there was a nuanced interpretation of physical activity among dialysis staff in that “doing more” activity meant patients could do more for themselves in general. Self-care was highly valued, and physical activity outside the clinic setting was viewed as a pathway to increased ability and independence inside the clinic, such as patients getting their own blankets or ice. Thus, for many, being “more active” meant being more proactive in self-care and less demanding of staff time. | | |  |  |  |  |  |  |  |  |
|  |  | All interviewees observed deteriorating physical function over time for most patients and seemed frustrated and often saddened by this. One nurse’s reaction to this spiral of incapacity was: “It is sad to watch them. I feel bad for them.” Another nurse said it was “disappointing,” implying an element of choice on the patient’s part. She explained how this cycle results in complete immobility and dependence: “At first, patients do not feel well because of their renal failure. When they start dialysis they feel better, but by that time they have already settled into a pattern of inactivity.” “When a woman formerly ambulatory returned to the clinic in wheelchair after a hospitalization, I asked her ‘are you feeling weak?’ ‘No.’ ‘Well, then how come you’re in a wheelchair?’ ‘Well, when they wheeled me out of the hospital they said I had to go out in a wheelchair and I’m supposed to stay in the wheelchair.’” “We see them go downhill. They will go from being very independent and then just slowly [they start to say], ‘Will you get this?’ ‘Will you do that?’ ‘Will you move my leg?’ Just the simplest things that they use[d] to be able to do, they cannot anymore.” “People start out being able to walk, then they’re in a wheelchair, then they’re in their motorized wheelchair, then they’re coming in on a gurney. They get to the point where they can’t feed themselves.” “...it is a cycle. It makes for a much happier place when people are able to do things for themselves. You see them bebop in the door and then a year later they’re in a wheelchair and they can hardly raise an arm. It snowballs and then the next thing you know they can’t do anything and they’re miserable and they’re upset and they’re putting on a lot of fluid and then they don’t feel good. It is sad to see them go from having kidney failure and being a pretty normal person to someone who’s just completely dilapidated, just has no life after they leave here because they just don’t feel good.” | | | Maintain independence | | | | Beliefs about consequences | | | |
| 11. Zelko et al. (2023) | | | | | | | | | | | | |
| The benefits of intradialytic exercise: Improvements in patients’ physical functioning and musculoskeletal structure | | All participants expressed one or more benefits related to improvements in muscle strength or muscle mass. Three-quarters believed that IDE increased patients’ muscle strength, and one-quarter expressed benefits related to gains and maintenance of muscle mass volume. A minority reported improvements in muscle endurance, flexibility and fine motor skills.  “In that manner, exercise increased their muscle volume and aroused them physically. Simply, they had more endurance and strength” [nurse, f, 34 y, 10 ype]  “The patient couldn’t make it 200 metres uphill in his walk home from dialysis. He travelled everywhere in his car. At the very end of our exercises, he was able to walk that way comfortably. He could walk to his home on the second floor.” [training assistant, m, 21 y, 1 ype] | | | | Perceived benefit to physical well-being | | | | Beliefs about consequences | | |
| The benefits of intradialytic exercise: Improvements in patients’ psychosocial functioning | | Nearly all participants believed that IDE positively affected patients’ psychosocial functioning. In their view, patients in standard dialysis regimens are highly vulnerable, resigned, self-regretful, depressive, grieving, wrathful, beaten inside and socially isolated. Participants expressed that IDE could change patients’ self-perception, make patients happier, less stressed, depressed and anxious, and contribute to positive attitudes and co-activation.  “It (exercise) is for them like summer sunshine, such lighting, revival and so on.” [nurse, f, 60 y, 31ype]  “They are very old, some very stone-hearted, and also without zest for life. Maybe some somatic-psychological effects of the warm-up and a little bit of stretching help them.” [nurse, f, 52 y, 28 ype]  “Maybe they were also different in a psychological setting. Because when you’re there, patients are happier. Some couldn’t wait for your arrival.” [nurse, f, 43y, 20 ype]  “We (patients and training assistants) were conversing during the exercise; therefore, they (patients) didn’t feel absolutely alone.” [training assistant, f, 22 y, 1 ype]  “There’s a possibility of endorphins secretion and the patient should feel better, that he’s not just lying there and feels beaten by lying for four up to five hours.” [training assistant, m, 21 y, 1 ype] | | | | Staff recognition of the benefits (general) of IDE | | | | Beliefs about consequences | | |
|  |  |  |  |  |  | Perceived benefit to mental well-being | | | |  |  |  |
| The benefits of intradialytic exercise: Improvements in patients’ clinical profile and quality of therapy | | The majority of participants believed that IDE could improve (to a certain and individual extent) the clinical profile of haemodialysis patients. The most frequently reported benefits were improvements in glucose metabolism, better cardiac and vessel health, a decrease in overhydration and lower morbidity and mortality of patients due to improvements in their nutritional status.  “Well, I think it (exercise) helps them to better quality of treatment.” [manager, m, 57 y, 23 ype]  “In the majority of diabetic (patients), if they exercise, there’s a decrease in blood saccharides. They burned more saccharides because they produced more insulin.” [nurse, f, 49 y, 20 ype] “Well, I think that exercise improved their blood flow, (in) the circulatory system and the blood circulation in brain and also filling of veins and thus for us also better blood flow in vascular access.” [nurse, f, 43 y, 20 ype]  “I think that general health status (is affected). Because when we exercise, we strengthen our immunity.” [nurse, f, 38 y, 16 ype] | | | | Positive beliefs about physical activity | | | | Beliefs about consequences | | |
|  |  |  |  |  |  | Belief that IDE could enhance dialysis treatment | | | |  |  |  |
| The benefits of intradialytic exercise: Improvements in patients’ independence and self-efficacy | | Most participants believed that IDE could improve patients’ self-efficacy in activities of daily life, decrease their dependence on help with basic needs from caregivers and family members and improve the level and safety of patients’ mobility.  “They stop feeling like diseased persons who need to be treated and who need care from all others around, but actually can live a full life. They can join (others); they can work, and they can go on trips.” [nurse, f, 54 y, 36 ype] | | | | Maintain independence | | | | Beliefs about consequences | | |
| The benefits of intradialytic exercise: Alterations in patients’ perception of time spent in dialysis treatment | | According to most participants, haemodialysis patients qualify time spent on dialysis as “boring”, “useless” and “tiresome and endless”. Half of the participants stated that IDE could alter patients’ perceptions of dialysis time and thus relieve their stereotypic feelings about the dialysis routine.  “Dialysis time goes faster for them, especially for older patients, who normally sleep or watch TV during dialysis, but most frequently check clocks and ask: how much is left? How much to the end?” [nurse, f, 45 y, 6 ype]  “Like I mentioned, it’s a stereotype, it’s boring to spend four hours sitting. Not everyone likes watching TV or reading. Maybe their movements on the bed are important regarding the shortening of that time.” [nurse, f, 34 y, 10 ype] | | | | IDE could improve experience of dialysis | | | | Beliefs about consequences | | |
| The benefits of intradialytic exercise: Distractions of patients’ minds from feelings of physical and psychological discomfort | | One-third of participants believed that IDE led to the distraction of patients’ negative thoughts about nephrology diseases, causes and causality of their health problems and further consequences during disease progression.  “They (patients) didn’t have time for deep thinking; they also detached themselves a little from their diseases.” [nurse, f, 43 y, 23 ype] | | | | Perceived benefit to mental well-being | | | | Beliefs about consequences | | |
| 17a. Young *et al*. (2015) (pre implementation of IDE) | | | | | | | | | | | |  |
| Enhanced  knowledge and  skills Young *et al*. (2015) | | | “A lot of [patients] will come up with...  barriers, so if you can have the  knowledge about it to be able to  overcome those just in a general chat”  (Senior staff focus group)  staff expressed a need for greater knowledge and also requested comprehensive training to enhance their skills around running an IDE programme, particularly setting up the bikes and encouraging patients to participate. | Knowledge to reassure and encourage patients | | | Knowledge | | | | |  |
|  |  |  |  | Patient encouragement from staff | | | Social Influences | | | | |  |
| Assessment Young *et al*. (2015) | | | “Will we get any training on how to work [the bike]?” (Junior staff focus group) “[Training] gives the staff chance to learn about [IDE] and understand the information and be able to learn it. You can’t expect them to do it straight away.” (Senior staff focus group) | Staff having practical knowledge of how to facilitate and support IDE | | | Knowledge | | | | |  |
| The influences of  peers and  colleagues Young *et al*. (2015) | | | “We should all get shown how to use the machine. That would throw a spanner in the works if people say they didn’t know how to work it.” (Junior staff focus group) | Staff having practical knowledge of how to facilitate and support IDE | | | Knowledge | | | | |  |
|  |  |  |  | Lack of practical knowledge to support | | | Knowledge | | | | |  |
|  |  |  | “I think it’s there persona around the person giving the advice. We all have the knowledge but I think [the patients] might prefer initially to have [an exercise professional].” (Senior staff focus group)  Junior staff strongly  Table 5. Facilitators to IDE post-implementation, as identified by patients and staff.  Post implementation  facilitators  Theory domains Patient Staff  Positive outcomes of  participating in IDE  Beliefs about consequences,  Reinforcement, Emotion  “I used to struggle with my blood pressure,  towards the end it always used to drop, I  wondered if the exercise would help to  stabilise it and it did, so that was a plus” (56  year old male Asian patient)  Last year [before the cycling] the dialysis  patients tended to be unmotivated, depressed.  I’ve seen them cycling and they are more  cheerful, happy, its helping them” (Nurse)  “I can now walk up to the village which is  about half a mile and I feel it’s the cycling  that’s helped” (75 year old female White  British patient)  “As a doctor working on a dialysis unit it can  sometimes be fairly bleak in that dialysis is a  very good treatment for keeping people alive  but doesn’t always enable people to live. I think  if there is a treatment that makes [patients] feel  better that makes you feel a whole lot better  about what you do to people.” (Consultant  Nephrologist).  “Because of the exercise I can sleep better.  I can sleep 5–6 hours at a time” (67 year old  male Asian patient)  “I am surprised at a ...lady I thought wouldn’t  do it but she did. I needed to be proven wrong  because we are not always right” (Senior  nurse)  Collaboration and  teamwork  Social influences, Behavioural  regulation, Social/professional  role and identity  “Even [nurse in charge] will get the bikes out  ...you have to have her on board, and the two  deputies.” (Dialysis Assistant)  “Because the [exercise professional] has taught  [the patients] we just set the bike up for them.  Patients will tell us how to fill the paperwork  in...they will say come back in however many  minutes or if I have a problem I will call you...”  (Nurse)  Junior staff strongly stressed the need for training to be available to all grades of staff and professional groups, whilst some senior staff believed they already possessed the knowledge and skills required. | Tailored physical activity (by healthcare professional) suitable for dialysis patients | | | Social Influences | | | | |  |
| Exercise  professional  support Young *et al*. (2015) | | | “[An exercise professional] would be  specifically coming to do [IDE] and we  have got a lot of other things and we can  be taken away at any point.” (Junior staff  focus group) | Nurses lack time for IDE due to other tasks | | | Environmental Context and Resources | | | | |  |
|  |  |  |  | Additional, exercise specific staff | | | Environmental Context and Resources | | | | |  |
|  |  |  | “I think long term it doesn’t need to be [an  exercise professional] that runs it, you  might find a champion comes from the  most unlikely source really...” (Senior  staff focus group)  Junior staff felt strongly that this professional should also be responsible for the day to day provision of IDE, whilst senior staff believed that support from an exercise professional was not required beyond initial implementation and that responsibility for the programme might come from another source such as a nurse, non-qualified member of staff or patient. | Exercise champions | | | Social Influences | | | | |  |
| 17b. Young *et al*. (2015) (post implementation of IDE) | | | | | | | | | | | |  |
| Positive outcomes of participating in IDE Young et al. (2015) | | | Last year [before the cycling] the dialysis  patients tended to be unmotivated, depressed.  I’ve seen them cycling and they are more  cheerful, happy, its helping them” (Nurse) “I am surprised at a ...lady I thought wouldn’t do it but she did. I needed to be proven wrong because we are not always right” (Senior nurse)  Staff were also strongly motivated by these benefits, which they had either observed for  themselves or heard about from patients. Positive changes in patients’ moods, improved concordance with dietary and fluid recommendations and reduced symptoms were particularly influential to staff. Observing these benefits dispelled misconceptions about patients’ abilities and  boosted staff engagement. All staff positively described how their involvement had made them feel good and how the ethos of the unit had changed to one of health promotion  following implementation. | Observing/feeling the benefits | | | Reinforcement | | | | |  |
|  |  |  |  | Knowledge that a wide range of patients can/do take part in IDE | | | Knowledge | | | | |  |
| Collaboration and teamwork Young et al. (2015) | | | “Even [nurse in charge] will get the bikes out ...you have to have her on board, and the two deputies.” (Dialysis Assistant) “Because the [exercise professional] has taught [the patients] we just set the bike up for them. Patients will tell us how to fill the paperwork in...they will say come back in however many minutes or if I have a problem I will call you...” (Nurse)  A collaborative approach between staff members of all  grades and disciplines, as well as patients was seen to facilitate the initial implementation and  maintenance of the programme. For junior staff, the active involvement and leadership of senior staff was particularly influential. Four staff members also described how patients competent in using the bikes and familiar with the running of the programme had a positive  influence. | Collaboration between staff team to support IDE | | | Social Influences | | | | |  |
|  |  |  |  | Active involvement and leadership of senior staff | | |  |  |  |  |  |  |
|  |  |  |  | When patients can retrieve their own equipment for IDE and monitor themselves/low reliance on staff | | | Environmental Context and Resources | | | | |  |

## Barriers Staff Where facilitators are found in theme categorised as barriers, they are highlighted in green.

| Original barrier  /Study | Data | New barriers (after splitting) | TDF domain mapped to |
| --- | --- | --- | --- |
| 2. Kontos *et al*. (2007) | | | |
| \| Transport  Kontos et al. (2007) \| patients’ fear of the penalties resulting from missed rides was identified by nurses as a factor impeding exercise practice. As one nurse commented, ‘‘One of the barriers with...exercise...[is] the Wheel-Trans because people are fearful of losing their Wheel-Trans. If they lose it, it’s a big deal.’’ So great were patient worries over penalties that nurses commented it was commonplace for patients to request shortened dialysis treatment if it appeared they might miss their Wheel-Trans. A nurse who expressed belief in the importance of exercise for patients commented that exercise is a low priority when ‘‘you have patients screaming, ‘...I want to get off [my dialysis], my Wheel-Trans is coming.’’’ \| Fear of losing transport benefit makes exercise lower priority \| Environmental Context and Resources  l \| \| --- \| --- \| --- \| --- \| \| Exercise not a priority for nurses \| \| Time  Kontos et al. (2007) \| “You are literally going from one task to the  next. So exercise, I certainly believe in exercise, for myself and for the patients. But it’s a low priority when you have a patient crashing or someone is bleeding.”, “ That falls down very low on the priority list. I  don’t have one patient to keep alive, I have 4  and they are all very elderly and all very needy and sick so that tends to fall, if I have time that’s like icing on the cake. I would love to do it if I had time.” \| Nurses lack time for IDE due to other tasks \| Environmental context and Resources \| \| Exercise not a priority for nurses \| \| Equipment  Kontos et al. (2007) \| ‘‘The ones with low blood pressure, they  cannot do the bicycle because they have to be sitting up to do the bicycle, and with low blood pressure you have to lie them down with the legs higher.’’ In addition, family care providers and patients expressed fears that using the bicycle would damage the fistula or chest line.”, “ Problem was that her line, she has a chest line, doesn’t always work well with movement. But she is very motivated now...and she does want to begin exercising...but because of the line, I mean it’s still kind of a fearful thing because  you have this thing protruding out of your chest. So she is a little worried about what exercises would be safe, what wouldn’t be safe.”, “You cannot even get to the machine in case of an emergency. With the bike in the way it is very hard to get to the machines without jumping over things, it’s really hard. I mean, God help you if a patient runs into some trouble during dialysis.”, ‘‘They’re big, heavy and it’s hard to move them. It hurts your back.’’ Because of these concerns some  nurses would rather not encourage the use of the bike by patients.”, “Most of us are women, we’re not big strong men, and it’s hard for us to move these big things, and should I risk killing my back so the patient can exercise for 10 min? So I think if we  had user friendly equipment that was easier for us to move around that would make a  difference.”, ‘‘I will discourage the patient because I don’t want to hurt my back.’’ \| General fears about safety \| Beliefs about consequences \| \| IDE bikes not suitable for everyone \| \| Lack of enough information support for patients \| Knowledge \| \| Reduced access to patients in emergency if IDE equipment in the way \| Environmental context and Resources, Beliefs about consequences \| \| Manual handling challenges for staff \| Environmental context and Resources \| \| Nurses fear injury to self from equipment \| Beliefs about consequences \| \| Fear of fistula damage \| \| User friendly IDE equipment \| Environmental context and Resources \| \| IDE equipment that is easy to move and maintain \| \| 4. Thompson *et al*. (2016) \| \| \| \| \| The role of the dialysis nurse  Thompson et al. (2016) \| “So that’s the hard part, I find, like, with patients who don’t know as well as others know, what they have to do. I think we have to do some minor adjustments on the bikes; seems to be a little bit more tension, just a little bit less tension, that’s something it’s quickly, we can do that and walk away; they’ll carry on with whatever they are doing. But some patients, like I said, who are not—I can’t say with it, but not as comfortable may be doing the exercises as others, it’s a little harder to—for us to monitor whatever they do is proper. I don’t know, it’s maybe they need a bit more education or its maybe they are not good people for the study.” \| Exercise monitoring/assessment/provision not included in care provision/ not the nurses’ role \| Social/Professional Role and Identity \| \| No Time \| Many staff members commented that there was “no time” to assist patients with IDE. The expectation that staff had the time to participate may have negatively influenced some staff’s attitudes toward IDE (Q35). For some staff, “no time” also meant that IDE was a low priority in their workflow and that IDE was seen as “extra work.” One staff member questioned the appropriateness of exercise for the dialysis unit (Q36). Another attributed their lack of time to the unpredictability of staffing and patient acuity. Staff often expressed that, because of the demands of the unit, the situation was irremediable (Q37). IDE as a low priority “I do know before the actual program started, I believe there was talk of the nurses taking on the role, and I don’t know if that was true…a lot of the nurses were not impressed, and they discussed that, that there’s just not time for that.” Q35  High demands on the unit “[Do the exercise] before they start dialysis, because it really is, like, here sometimes we have people come late or whatever, we’re busy, because something is seriously wrong with one of the patients, you just don’t have time; actually, you just don’t have time to do it. There’s already stuff that we’re supposed to do that we don’t have time to do.” Q36  “No, because even though if we are so-called satellite unit, people sometimes they feel sick and then they couldn’t do it and then we are busy, then we couldn’t help out with having the exercise done, and then we just have to leave it for [the kinesiologist] to come. If they don’t come that day, they just have to skip the exercise. Yeah, ‘cause, still, that is not the priority, is to help our patient’s safety, right? If they don’t feel good and some other emergency—that we have to deal with an emergency instead of helping them out with the exercise.” Q37 \| Nurses lack time for IDE due to other tasks \| Environmental Context and Resources \| \| Exercise not a priority for nurses \| | | | |
| 8. Jhamb *et al*. (2016) | | | |
| Theme 2: Reported barriers to exercise: dialysis makes exercise challenging | “I know the machine makes them tired, dialysis in general is just you know it’s a tiring disease” | Fatigue | Skills |
|  | "It’s also about finding what kind of exercises they might like or could fit into their day, just because dialysis already takes up so much time.” | Patient lack of time due to dialysis | Environmental Context and Resources |
|  | “Because of their age and maybe their health too. Some are sicker than others.” | Poor physical condition | Skills |
|  | “The tough part of putting in exercise regularly you know is more of a mental challenge than it is physical” | Lack of motivation | Intentions |
|  | “A lot of our patients are there because they really didn’t take care of their diabetes, hypertension, not very many are interested but some of them are interested in changing their ways and being healthier, but some are just like “Oh well”. |  |  |
|  | Providers felt most patients lacked the motivation to take care of their health and were thus not likely to care about exercising. |  |  |
|  | “The nature of a chronic illness I think has a set of mental challenges and obstacles all by itself simply because it’s a chronic disease” | Poor mental health | Skills |
|  | “I think I would [be more comfortable with counseling] if I had a guideline to follow or something, I think a little training, in-service training, would be good” | Doubt in their capacity to assist with IDE | Beliefs about capabilities |
| Theme 3: Reported barriers to intra-dialytic exercise: intra-dialytic exercise should be safe without disrupting usual care | “They could dislodge a needle possibly …. it would also cause them pain, and it could really cause some large damage to their access…the biggest barrier that I could see…. Just moving around too much and cause an infiltrate” | Concern injury and accident | Beliefs about consequences |
|  | “If they do too much movement they won’t get a good treatment because they will constantly alarm” | IDE could disrupt dialysis |  |
|  | “When people put their feet down you know blood pressure drops so it would definitely make it a little more challenging as far as making sure that the vitals are staying stabilized.” |  |  |
|  | fear of needle dislodgement and infiltration and cramps due to exercise were commonly reported barriers |  |  |
|  | “Sometimes just moving around causes cramping in a patient” | Fear of pain | Beliefs about consequences |
|  | “Buying equipment so we would need to get permission with [the administrator] with the budget. I don’t know if we’re going to want to spend all this money on some sort of equipment and have half the patients not use it” | Lack of resources | Environmental Context and Resources |
|  | Several participants talked about limitations related to the ease of use, cost, and storage of exercise equipment |  |  |
|  | “Some kind of equipment becomes cumbersome for patients” | IDE equipment not suitable for everyone |  |
|  | “Depending on the exercise, you have space limitations” | Facilities too crowded |  |
|  | Some of them used it [stationary bike] for a short time and then they lost interest in it | Previous negative exercise experience influences perception of IDE | Reinforcement |
|  | “I think for some people it could be a self-conscious thing and maybe they you know some people don’t like to go to the gym because they don’t want others to see them.” | Lack of privacy in IDE | Environmental Context and Resources |
|  | “As far as the staff goes I don’t think we would have the time to do any really one-on-one [exercise].” | IDE as an additional source of pressure | Environmental Context and Resources |
|  | “It would have to be very minimal. As our staffing doesn’t provide for helping them exercise. I mean just setting them up for it is you know is extra work. we don’t always have the staff and we don’t always have the time” | Exercise monitoring/assessment/provision not included in care provision/ not the nurses’ role | Social/Professional Role and Identity |
|  | “With the bicycle[stationary bike]…that did add to our work load and not that it was a bad thing but you know it was a good thing for the patients but….I really don’t have a minute to spare” | Staff doubt in their capacity to assist with IDE | Beliefs about capabilities |
|  | Some of them had prior experience using a stationary pedaling bike during HD and offered reasons for poor adherence...staff felt that it added to their workload. |  |  |
|  | “The exercising…..we try to encourage them as much as … some people can be very….strong-willed [in adopting changes to their HD routine].” | Lack of motivation | Intentions |
|  | “I just don’t see them being willing to, ….. they want to get on the machine, they want to either go to sleep or they want to sit there and do nothing and watch TV” |  |  |
|  | providers viewed resistance in terms of patients’ personal characteristics, describing some, especially long-term HD patients as “strong-willed”, and introducing any change to dialysis routine as being “an uphill battle” |  |  |
| 9. Painter *et al*. (2014) | | | |
| Theme 4: Barriers to Physical Activity Painter *et al*. (2014) | Nurses viewed the resulting physical limitation and low activity demonstrated in this case as a by-product of incomplete messages given by professional caregivers and internalized by the patient to view herself as unable to be active. Another dialysis staff member elaborated on how patients internalize a view of their own physical inability: “A very common reaction is, ‘I’m not supposed to use this arm because I’ve got a fistula in it now.’ Well, the next thing you know they’re not using that arm at all.” Patients’ perceived physical inability can manifest as a license to exert control over others, such as requests of staff, because “I can’t use this arm [with the fistula].” When asked why not, patients say, “I’m not supposed to.” To the dialysis staff, the “take it easy” message is given early in their treatment, without enough detail or follow-up to help patients understand when “take it easy” should give way to increasing physical activity. “They don’t fully understand what’s going on and then the next thing you know they’re pretty incapacitated because they just thought ‘well, I wasn’t supposed to do that.’” Physical limitations are compounded by social responses that reinforce their passivity as objects of care, rather than active participants in their lives, resulting in a cycle of increasing debility. | Used to being sedentary | Behavioural Regulation |
| Theme 4: Barriers to Physical Activity Painter *et al*. (2014) | “I think the limitations come more from the comorbidities than the dialysis.” | Poor physical condition | Skills |
|  | “I think a lot of it is weakness. They’re not being compliant with their fluids, and that fluid gain really puts a lot of pressure on their extremities. It makes it difficult to walk.” |  |  |
|  | As expected, the most frequently cited were clinical factors, including older age, comorbid conditions, diabetes, heart problems, and depression. However, the physical effects of dialysis were noted to diminish interest and energy, particularly if the patient was nonadherent to fluid restrictions. |  |  |
|  | Mental dimensions of kidney failure such as depression also were mentioned as barriers. | Poor mental health |  |
|  | “A lot of the hemo patients that I’ve discussed this with, they feel tired, really wiped after treatments, and so they don’t do anything. They go home and a lot of them sleep for a number of hours, and then they just don’t get that exercise.” | Fatigue | Skills |
|  | “When they first come in to get dialysis nobody ever feels good, they don’t really have any energy. That just kind of gets their mind set: this is what my life’s going to be. I’m never going to have energy, I’m never going to feel good. I might as well give up. And because I don’t feel good, I don’t want to do it.” | Patient belief that no improvement is possible | Optimism |
|  | “Our patients just diminish and give up. They think ‘This is it. This is basically where I’m going to be, and then I’m going to be gone.’ They don’t have to stay active if they’re diminishing.” |  |  |
|  | “They get the impression that they’re sick and that they can’t do it.” | Identity as a sick person | Social/Professional Role and Identity |
|  | “Depression. It’s a lifestyle. They don’t feel good.” |  |  |
|  | “I don’t know if it’s a mental thing or not. They think they’re sick and that’s an excuse not to do anything.” |  |  |
|  | “When they go into kidney failure, they become automatic disabled. Some of them adopt the label – ‘I’m disabled.’” |  |  |
|  | “I think there’s a lot of them that kind of get into a little bit of routine or they don’t fully understand what’s going on and then the next thing you know they’re pretty incapacitated because they just thought well, I wasn’t supposed to do that.” |  |  |
|  | Staff referred to patient nonparticipation in physical activity as a “lifestyle” or “mental thing” or an “impression” that they could not exercise. |  |  |
|  | “A lot of our patients are in the low-income category and live in low-income housing or apartments and can’t afford to pay for anything.” | Cost of exercise facilities/equipment | Environmental Context and Resources |
|  | Structural barriers were mentioned secondarily after physical and mental health barriers. These included income- and access-related barriers |  |  |
|  | “However, they could go out the door and walk around the block or something.” | Lack of motivation | Intentions |
|  | “Staff over-accommodate to the point that we make everybody’s doctors’ appointments. We enable them so much. We do everything for them. They just get used to not doing anything for themselves. Even little things.” | Excessive support from staff encourages dependency | Social Influences |
|  | Staff noted their own complicity in reinforcing patients’ view of themselves as too sick and unable to be physically active. Because they understood that patients did not feel well and they wanted to demonstrate compassionate care, they would help them to do things, such as take off their coats or get their blankets. It was easier to do it for them than encourage self-care. This enabling behavior was recognized as contributing to low activity |  |  |
|  | Families also protected patients and enabled low activity. “Family members are enablers,” stated one staff member, “instead of encouraging him they’re more like ‘oh, poor Peter.’” Some families were less inclined to “enable” the patient: “And some of them are like, ‘no, physical activity will make you feel better.’” | Family concern of ability |  |
|  |  | Importance of patient’s family | Social influences |
|  | “The environment almost encourages them not to be active. We give them so much information about all the ways they can get benefits so they don’t have to work and don’t have to be active.” | Expectation for patient to access benefits and not work | Environmental Context and Resources |
|  | The staff themselves stated that they encouraged the use of safety net resources that facilitate inactivity |  |  |
|  | “Doctors tell them, ‘you are restricted on this, you are restricted on that’ and they think, ‘well I just cannot do anything.’” | Misconceptions about the relationship between physical activity and condition(s) | Knowledge |
|  | “You have Medicare and you can get Social Security benefits if you decide not to go back to work. You know what I’m saying? I’m not saying that people are lazy but sometimes I think those benefits give just a bit of encouragement so people stop working. That makes them less active.” | Expectation for patient to access benefits and not work | Environmental Context and Resources |
|  | The medical care and social welfare systems also contributed to inactivity by medicalizing hemodialysis and acknowledging kidney failure as an “automatic qualification for disability.” |  |  |
| Theme 5: Practice Patterns of Assessment of Physical Function and Encouragement for Physical Activity Painter *et al*. (2014) | All patient care staff stated that there was no routine assessment of physical function undertaken in their practice setting. The closest the nurses get to asking about physical function or physical activity is a question about limitations in activities of daily living (ADLs) during their initial dialysis intake assessment. PCTs were similarly clear that their care was dialysis related and protocol driven. There was little clinical assessment and no formal plan for referral for those who may report physical function limitations.... No one could identify a specific role or individual who was responsible for providing information about physical activity. It was not part of any staff job description and they stated that it probably was the job of the physician. Staff would depend on patients to bring up the issue if they were having problems with mobility or ADLs. If a staff member thought a patient could benefit from a physical therapy referral, the information would be directed to the physician; however, there was no follow-up on results of that information transfer. Thus, the staff would have no information from physical therapy (if a referral was made) to encourage the patient....“Our care plans are all dialysis related: monitoring the aspects of dialysis, blood pressure and so forth.” “We chart the physical limitations but we do not do anything about it.” “[The doctors] could, I suppose, order physical therapy, but we haven’t dealt with that.” “I’d never heard of those guidelines [before you came here]. I don’t think anybody’s thought about getting these people exercising.” “Starting an exercise program is not what I’ve been trained to do, so unless I had some training.It’s a liability if you tell them something wrong. How do I know what they’re going to do? What if they have brittle bones or a heart problem, you know?”... Staff preferred “something standardized” so they would “feel comfortable” talking with patients on the topic. “It’s probably more if the institution wanted to initiate it then we would,” summed up one long-time staff member. The idea of inserting a nonstandardized element of care based on clinical judgment was considered unsupported, possibly unsafe, and professionally risky. | Exercise monitoring/assessment/provision not included in care provision/ not the nurses’ role | Social/Professional Role and Identity |
|  | Several staff mentioned that the physician sometimes recommends weight loss to patients interested in transplantation and suggested watching calories and increasing physical activity with no specific information provided. “The doctors just say, ‘Well, you need to lose weight, you need to exercise.’ But it’s not super helpful.” | Lack of guidance/structure for patients | Knowledge |
|  | Lacking a unit-based protocol for physical activity or any staff person responsible for the topic, a checkered pattern of individual staff effort based on good intentions was described (Box 6). In keeping with the value of self-care, some staff encouraged physical activity by “encouraging people to do all they can for themselves and try to walk out [of the dialysis center] if they can.” “As a routine part of care, no, I don’t try to convince them to be physically active. Occasionally I will. There are a few patients taking it a little too easy.” “I tease them, you know. I’m not mean and nasty. I don’t say you need to get off your butt and move. But I let them know what’s going to happen if they don’t get up and move.” “I’m sure the technicians could ask about activity, but it’s almost like you can’t suggest something to the patient because you’re not qualified to do that.” | Patient encouragement from staff | Social influences |
|  | Patient care staff expressed varying levels of confidence in making recommendations about physical activity. One PCT expressed willingness, stating “I’m sure the technicians could ask about activity, but it’s almost like you can’t suggest something to the patient because you’re not qualified to do that.” The same hesitancy was echoed by every other category of staff. | Staff not knowing what is right to instruct | Knowledge |
|  | The practical matter of accessing resources also was a barrier to staff activation: “I don’t have the resources” and “We don’t have any pamphlets on physical activity that I know of.” | Existing resources do not include physical activity/exercise guidance | Environmental Context and Resources |
| Theme 7: Observational findings Painter *et al*. (2014) | Review of the facility policy and procedures manual showed assessment of physical activity as part of the health history done by the registered nurse at the time of initiation of dialysis (within 30 days). The Activity/Exercise section of this health history listed 6 questions: walking and transfer independence, limitations (specifically symptoms of weakness, fatigue, dizziness, fainting, shortness of breath, and pain), need for oxygen, and level of exercise (sedentary, light, moderate, high, and how many times per week). Need for assistance with 11 ADLs was determined. The annual review of this history usually does not include the Activity/Exercise section. The medical staff is responsible for “a medical assessment to reflect the oversight and review of all areas in the assessment and care of the renal patient.” The final aspect in the list of 8 areas of care is “physical activity levels including possible physical or vocational rehabilitation.” The written plan of care must include goals and plans for each of the assessment areas, which includes “rehabilitation status.” The team member responsible for rehabilitation (physical and vocational rehabilitation) is the social worker. The ongoing plan of care after the first 90 days does not include activity or exercise... Six patients (2 women, 4 men; average age, 58 years; dialysis vintage range, 6-62 months) were observed to document the routine process that ensues upon the patient’s arrival to the clinic. The process is found in Box 10. There was no discussion of physical function or physical activity participation outside the clinic. There was only one observation of a PCT interaction during the weighing process that included physical function: The patient was in a wheelchair and had difficulty standing to be weighed. The staff member suggested that the patient practice standing up and sitting down to build up the strength in her legs so getting out of the chair would be easier. As suggested by this observation and confirmed by the interviews, physical function is assessed only when it affects the interactions/operations within the dialysis clinic. | Exercise monitoring/assessment/provision not included in care provision/ not the nurses’ role | Social/Professional Role and Identity |
|  | The website for the dialysis program includes a patient education section that has links to some information on physical activity, shown in Box 9. There was no indication as to whether and/or how patients are informed of this site. Staff were unaware of this resource. | Lack of information sources for patients and staff | Knowledge |
| 11. Zelko et al. (2023) | | | |
| The risks of intradialytic exercise: Exercise-related damage of vascular access and injury to the adjacent anatomical area | Three-quarters of participants believed that IDE could cause vascular access damage or malfunction and could injure the adjacent anatomical areas. The most frequently reported concerns regarded piercing and rupture of fistula, and pulling out the catheter and needles during haemodialysis therapy.   “For example, those patients with permcaths, I mean, catheters located in their chests. It is just enough that when he inhaled harder, and (his) permcath doesn’t work. I see only this risk (of exercise).” [nurse, f, 52 y, 28 ype] | Fear of fistula damage | Beliefs about consequences |
| The risks of intradialytic exercise: Insufficient individualization of IDE progressivity regarding patients’ physical condition | Half of the participants believed that insufficient individualization of training progress could increase the health risks of IDE and discourage patients from further exercising. They are worried that improper control and management of IDE could worsen their nephrology, cardiovascular, endocrine, neurology or neuromuscular diseases. Participants stated that the rate of progressivity should be individual and highly depended on the patients’ physical functioning, chronological and biological age, gender and other specific health conditions.  “Clinical profiles need to be reviewed first, but also psychological and physical conditions need to be taken into account. For example, younger patients are more willing to start exercise, while older are more susceptible to injury and overtraining.” [nurse, f, 54 y, 36 ype] | Concern injury and accident | Beliefs about consequences |
|  |  | General fears about safety |  |
|  |  | Tailored physical activity (by healthcare professional) suitable for dialysis patients | Social Influences |
| The risks of intradialytic exercise: Exercise-related injuries of musculoskeletal apparatus | One-third of participants expressed concerns that IDE could contribute to musculoskeletal injuries in haemodialysis patients. They believed that exercise during dialysis could contribute to tendon and/or muscle injuries. Furthermore, participants expressed the opinion that a combination of training-related injury with dialysis- and exercise-related fatigue could increase risks of falls and injuries in patients.  “They are heparinised; it’s important to avoid hitting, to minimise the creation of bruises. Also, avoid tendon injuries caused by sharp (extremities) movements. So basically to avoid injury because of exercise.” [nurse, f, 54 y, 36 ype]  “Maximally, I think that if the patient overloaded exercising, he could suffer injuries to muscles. When the patient is a little overwhelmed, he feels pain and muscle strain, but that’s nothing serious (for his health).” [nurse, f, 46 y, 23 ype] | Concern injury and accident | Beliefs about consequences |
|  |  | Fear of falling |  |
| The risks of intradialytic exercise: Insufficient understanding of patients’ clinical conditions and health-related risks | Some participants believed that insufficient consideration of patients’ actual clinical conditions by training assistants could increase health-related risks during IDE. Participants reported that patients are generally affected by musculoskeletal, cardiovascular and infectious disease risks and frequently experience acute clinical deterioration. They expressed that insufficient review and consideration of patients’ actual conditions prior to exercise session could negatively affects manifested diseases or hidden health risks in patients.  “High blood flow through dialysers is important for them (patients). Exercise could affect stability of flow and sometimes we need to reduce the operating speed (of the dialyser). This should affect dialysis efficiency.” [nurse, f, 43 y, 20 ype]  “So, I think that the positions of nephrologists are very important, because they monitor the patient’s health. So actually, if they see that it really makes sense, and if they agree, then I don't think there should be a problem.” [training assistant, f, 22 y, 1 ype] | Poor physical condition | Skills |
|  |  | Concern injury and accident | Beliefs about consequences |
|  |  | IDE could disrupt dialysis |  |
|  |  | Recommendations of healthcare professionals | Social influences |
| The risks of intradialytic exercise: Application of IDE early after initiation of haemodialysis therapy | A minority of participants believed that IDE could be risky for patients with acute kidney injury and patients early after initiation of maintenance dialysis. This could imply that these patients would be emotionally and physically overloaded by additional activity during the haemodialysis therapy. Participants preferred a cautionary approach to these groups of patients and postponement of IDE until the patient had established a dialysis regimen.  “It depends on whether it is a patient who suddenly fell into the (dialysis) programme or a patient who is from the nephrology outpatient clinic. For whoever suddenly falls, he cannot identify with the fact that something like this (dialysis therapy) has happened (to him).” [nurse, f, 38 y, 16 ype] | New patients overwhelmed by physical therapy requirement | Beliefs about capabilities |
| The barriers to intradialytic exercise: Limitations related to psychosocial dispositions of patients | All participants believed that patients had a minimal history of regular physical activity and remained inactive during their lives with nephrology disease. Their sedentary lifestyle and beliefs that physical activity is not important for their lives were identified as major barriers on the patients’ side for initiating IDE. Participants expressed their opinions that these attitudes resulted from patients’ frustration, passive negativism, shame, introversion, social isolation, closure personality, self-pity, “self-abandonment” and resignation on nearly everything. The minority reported that patients were deeply depressed with minimal “self-initiative” efforts for any exercise behaviour change.  “It really depends on the patient’s personality. One patient is willing to walk to dialysis, and another in nearly the same condition is requesting transportation. It’s about patient’s nature.” [nurse, f, 52 y, 28 ype] | Used to being sedentary | Behavioural Regulation |
|  |  | Depersonalisation due to dialysis | Social/Professional Role and Identity |
|  |  | Poor mental health | Skills AND Belief about Capabilities |
|  |  | Physical activity is optional | Goals |
| The barriers to intradialytic exercise: Lack of financial resources for personnel capacities | One of the most dominant subthemes was related to financial resources for the implementation of IDE. Most of the participants expressed their uncertainties about covering direct costs, especially those connected with financing personal capacities. Even in a minimalistic setting, IDE needs to be supervised, controlled and reported by one additional person in the care-providing team in every dialysis session. Participants believed that these costs could be covered by funding from health and research government authorities or health insurance companies. However, most of the participants were sceptical about the actual will for systematic change in reimbursement of health care. The only hope reported was an initiation for adding exercise intervention into the categorization system of nephrology medical services in the Slovak Republic. The majority of the participants believed that material resources for IDE were negligible and could be covered directly by internal financial recourses of dialysis centres. | Cost of exercise facilities/equipment | Environmental Context and Resources |
|  |  | Cost of dedicated staff |  |
|  |  | Exercise monitoring/assessment/provision not included in care provision/ not the nurses’ role | Social/Professional Role and Identity |
|  |  | Low cost material resources | Environmental Context and Resources |
| The barriers to intradialytic exercise: Limitations on the work capacity of care-providing teams at dialysis centres | Half of the participants reported that the implementation of IDE by training assistants produced minimal or no distraction of care-providing personnel and patients. Three-quarters of participants believed that nurses were not able to implement IDE due to their already high workload in monitoring and surveillance of patients during dialysis therapy. In their view, any additional task for nurses could negatively affect the quality of provided medical care. These participants also believed that IDE could be provided by other personnel of dialysis centres or as an external service for dialysis centres.  “During a dialysis session, one nurse is monitoring and supporting four patients. She cannot stay with one patient during half an hour for exercise.” [nurse, f, 54 y, 36 ype]  “To maintain exercise among, patients, additional personal attention is simply desirable, someone specialised and educated in this area, someone from outside the dialysis centre.” [nurse, f, 46 y, 23 ype] | Nurses lack time for IDE due to other tasks | Environmental Context and Resources |
|  |  | IDE provided by non-nurse staff | Environmental Context and Resources |
| The barriers to intradialytic exercise: Lack of support, information, motivation and guidance | One-third of participants reported that insufficient support from family is a barrier in IDE. About a quarter reported missing support from caregivers. In both groups, low accessibility to IDE-related information for caregivers, families and patients, and lack of motivation and guidance for patients was frequently mentioned as the barrier.  “We (nurses) would appreciate more information about possibilities for patients exercising and more communication (about exercise) with patients, nephrologists, physiotherapist and your academic colleagues.” [nurse, f, 49 y, 30 ype]  “Promotion or advertisements could help. To make patients informed and aware that something like this (exercise) exists and maybe they’ll start to ask for such activity on their own.” [training assistant, m, 22 y, 1 ype] | Lack of support from social network | Social Influences |
|  |  | Lack of information for caregivers and families | Knowledge |
|  |  | Lack of guidance/structure for patients |  |
|  |  | Lack of motivation | Intentions |
| The barriers to intradialytic exercise: Limitations related to the organization of patients’ therapy at dialysis centres | Some participants reported different barriers related to the “work culture” at dialysis centres. These subjects believed that IDE in a dialysis centre setting could be time- and space-limiting for patients and care providers. If IDE is performed shortly after dialysis starts, it could collide with nurses and nephrologists communication with patients. If performed late, it could postpone patients’ disconnection from the dialysis device. Space limitations were linked to patients’ privacy and quiet environment needs. Participants reported that a specific group of patients preferred to be more isolated during exercise, while other non-exercising patients might perceive IDE during dialysis as “noisy” and “sleep-disturbing”.  “We have an open-spaced dialysis room and some patients are ashamed to exercise. They (patients) preferred a more isolated space for the time of exercise.” [nephrologist, m, 43 y, 19 ype]  “Head nephrologists and nurses might initiate an exercise programme for patients. They’re responsible for the creation of conditions for such an activity.” [nurse, f, 54 y, 36 ype]  “Dialysis centres with higher and lower capacities are organised differently. Smaller centres usually have non-complicated patients and, let’s say, an easier mode. But I’m sure that in both cases, space and time capacities are suitable for patient’s exercise.” [manager, m, 57 y, 23 ype] | IDE could disrupt the routine on the dialysis unit | Environmental Context and Resources |
|  |  | Lack of privacy in IDE |  |
|  |  | A lack of support from  management | Social Influences |
| 17a. Young *et al*. (2015) (pre implementation of IDE) | | | |
| Staff workload Young *et al*. (2015) | Staff and patients both expressed concern about a lack of staff resources and busy workloads within the HD environment. All patients were wary of creating extra work for staff and believed that a lack of time would reduce supervision and encouragement during exercise. Staff agreed, and junior staff in particular strongly believed that IDE would increase in their workload. “It might not work if we have a very busy period. We have a lot of patients who need a lot of care, that would become our priority and it wouldn’t be the bike.” (Junior staff focus group) “There will always be an initial staff step back because its more work for them to do” (Senior staff focus group) | Staff doubt in their capacity to assist with IDE | Beliefs about capabilities |
|  |  | Nurses lack time for IDE due to other tasks | Environmental Context and Resources |
|  |  | Lack of time reducing effective delivery of IDE |  |
|  |  | IDE as an additional source of pressure |  |
|  |  | Exercise not a priority for nurses |  |
| Staff beliefs about  IDE and patients Young *et al*. (2015) | “I have only ever heard negatives [about IDE]. I was working in [another region] and they did it there and I heard a lot of moans...and then you see all the bikes at the side not being used” (Junior staff focus group) | Belief that IDE would be unsuccessful | Beliefs about consequences |
|  |  | Doubts about benefits |  |
|  | “[Patients] don’t really exercise, some do, but the majority don’t.” (Senior staff focus group) “At the moment I would say [exercise is] very low on [patients] agenda.” (Junior staff focus group)  Staff were initially extremely negative regarding  IDE, viewing it as a burden to both themselves and patients. They perceived patients to be  uninterested in IDE, incapable or unsuitable for exercise particularly if they were older or from  a minority ethnic background. | Staff don’t see patients as interested in exercise | Social/Professional Role and Identity |
| 17b. Young *et al*. (2015) (post implementation of IDE) | | | |
| Staff workload Young *et al*. (2015) | “Unless the staffing numbers start to get better I  don’t think we are going to have the chance [to  assist with IDE], it depends on how busy the shifts  are.” (Nurse) “I think the vast majority of staff are open to the  idea of cycling, it’s just what would happen on the  particular day. So if the [exercise professional] said  on X day we are going to have some training, is the  shift going to be a full compliment, are the staff  actually going to be in? I don’t think anyone has got  a resistance to actually learning.” (Healthcare  Assistant)  Staff workload and lack of time continued to be the most frequently cited barrier to sustaining IDE participation. Four staff members felt that they were unable to adequately supervise exercise sessions, creating concern about patient safety.  Large workloads, lack of time and unpredictable shift patterns also led staff to report difficulty attending IDE training sessions or using the knowledge and skills gained from them. | Nurses lack time for IDE due to other tasks | Environmental Context and Resources |
| Lack of staff  responsibility Young *et al*. (2015) | It would be nice to have [an exercise professional]  across two units or [a dedicated staff member] so it  doesn’t just burn out.” (Senior nurse)  “If you have somebody named on a shift that will  [provide the exercise] they can set it up and monitor  [the patients].” (Healthcare Assistant)  Staff and patients believed that there was too much variation  in IDE provision due to a lack of responsibility for the programme amongst staff. Seven patients identified the importance of an exercise professional to the initial implementation of the  programme but also felt that a dedicated staff member (not necessarily an exercise professional) was important to ongoing success. This individual could act as a ‘coach’, providing feedback, encouragement and support. | Additional, exercise specific staff | Environmental Context and Resources |

## Originally mixed themes Staff Barriers coded grey; facilitators coded green

| Original barrier  /Study | Data | New codes (after splitting) | | | | TDF domain mapped to | | | | |
| --- | --- | --- | --- | --- | --- | --- | --- | --- | --- | --- |
| 4. Thompson *et al*. (2016) | | | | | | | | | | |
| Theme1: Support (staff) 4. Thompson *et al*. (2016) | After hearing of the benefits of IDE from their patients, staff agreed that the exercise program was valuable for patients.  “No, I think it’s a really great program and I’ve had a lot of really positive feedback from the patients saying they have better energy levels, that they’re feeling healthier. So I’m very much about implementing the program on a more regular basis for dialysis patients.” | Staff recognition  of the benefits (general) of IDE (facilitator) | | | | Knowledge | | | | |
|  | However, systemic factors may have influenced staff perspectives of IDE. Changes to staffing ratios on the unit were to take effect in several months (unrelated to IDE but coinciding with initiation of the clinical exercise program). The knowledge that staffing was going to be “cut back” conveyed a lack of support from management (Q2). Several staff expressed uncertainty about the need for these changes and concern over how workflow in the unit might be affected (Q3). One staff suggested that these changes could be detrimental to patient care overall and expressed doubt in their capacity to consistently participate in IDE delivery (Q4).  “Right now is okay, but the only thing is I think also there is going to be some transitional—we’re going to have some changes on the staffing ratio on our unit, and they’re going to cut back on us, so it’s going to be some time during the day that they’re going to cut back; like, now we have nine staff, and they’re going to cut it back down to six staff…” Q2  “…So I don’t know how well, how much it will be affecting the [exercise] program, is going to be permanent for our patients. Because they don’t want that many—well, management has a reason to cut the staff, but we still have to wait and see what’s going to happen…”  “Well, things are changing for our unit and how the unit is run, so we’re going to be doing, like, different times and team nursing and everything, so we’re not going to have a lot of extra time to be helping patients with this [IDE], and it’s going to—we’re going to be short staffed—they’re going to take some staff ratios away. So it’s really going to affect us as well as the patients…” | Staff doubt in their capacity to assist with IDE (barrier) | | | | Beliefs about capabilities | | | | |
|  |  | A lack of support from  management | | | | Social influence | | | | |
| Theme 2: The role of the dialysis nurse - IDE is not the nurse’s role, awareness of their role in IDE and AND knowledge about IDE  4. Thompson *et al*. (2016) | Although staff recognized the benefits of IDE, they commonly expressed that assisting with IDE was not a nursing responsibility. One staff member indicated that it was the exercise (rather than assisting with a study) that was inconsistent with their role (Q16). Another staff member explained that tasks, such as IDE, were left to them by default (Q17). Although staff did not express safety concerns with IDE, one person expressed concern whether patients were “doing [the exercises] right” and commented that staff could not monitor it (Q18).    In the interview when encouragement was discussed, the staff members commented that patients would find encouragement to exercise more effective if it came from physicians, suggesting that staff may not appreciate their role in patients’ decision to exercise (Q19). Understanding of IDE could also influence staff interaction with patients. Several staff were surprised that the elderly patients had the physical capacity for IDE, whereas other patients, perceived as more suitable, were not interested (Q20). | Exercise monitoring/assessment/provision not included in care provision/ not the nurses’ role  (barrier) | | | | Social/Professional Role and Identity | | | | |
|  |  | IDE as an additional source of pressure (barrier) | | | |  |  |  |  |  |
|  | One staff member expressed that many patients in the unit were too immobile and sick to participate in IDE (Q21). | Patient belief that condition(s) preclude physical activity (barrier) | | | | Beliefs about capabilities | | | | |
|  | “Well, it’s some extra work, to be honest. Yeah. At first, it was kind of—well, we have a couple of studies ongoing, besides the ones that we have to do as a nurse for our patients and then answering alarms.” | Exercise not a priority (barrier) | | | | Environmental Context and Resources | | | | |
|  | “So that’s the hard part, I find, like, with patients who don’t know as well as others know, what they have to do. I think we have to do some minor adjustments on the bikes; seems to be a little bit more tension, just a little bit less tension, that’s something it’s quickly, we can do that and walk away; they’ll carry on with whatever they are doing. But some patients, like I said, who are not—I can’t say with it, but not as comfortable may be doing the exercises as others, it’s a little harder to—for us to monitor whatever they do is proper. I don’t know, it’s maybe they need a bit more education or its maybe they are not good people for the study.” | When patients can retrieve their own equipment for IDE and monitor themselves/low reliance on staff (facilitator) | | | | Environmental Context and Resources | | | | |
|  |  | Staff not knowing what is right to instruct | | | |  |  |  |  |  |
|  | “No. I think it’s just who it comes from is definitely the importance. They tend to put a lot of trust in the doctors, so I believe if it [encouragement] comes from a doctor, then it would affect their thinking a little bit more than if it was to come from a nurse or somebody that does exercise and is promoting the exercise. I think if it came from a doctor, the importance of it, then it probably would be more important to them.” | Recommendations of healthcare professionals (facilitator) | | | | Social Influences | | | | |
|  | “Yeah. It’s actually pretty surprising. Some patients that you wouldn’t think would have the stamina really enjoyed it and really did the bike for, like, 45 minutes…and some patients you would think that would appreciate doing it didn’t want to become involved…some of the patients, like, in their 70s, 80s, really enjoyed it.” Q20  “I think they’re [study staff] limited to the number of patients that they have on there, just because of our patients—the patients that we have there…their mobility is decreased already, they’re sick.” | Knowledge that a wide range of patients can/do take part in IDE (facilitator) | | | | Knowledge | | | | |
| Theme 3: Norms within the Dialysis Unit (staff) 4. Thompson *et al*. (2016) | Many of the staff expected that, before asking for help with IDE, dialysis-related tasks at the start of the shift were completed (Q28). Initiating IDE at the start of the shift was challenging, and some staff expressed frustration about how to effectively communicate with patients about the timing of exercise during dialysis (Q29 and Q30). One staff member indicated that negotiating aspects of HD delivery with patients was a preexisting issue, suggesting that IDE may have been an additional pressure (Q31). | Exercise not a priority for nurses (barrier) | | | | Environmental Context and Resources | | | | |
|  | “…But for us, sometimes we still have other patients to take care of, put patients on, and so sometimes we don’t get there until an hour or even 2 hours later.” Q29,   “They’re quite—they have quite negative comments if we can’t get to them in the time that they want. So unfortunately then the discussion of ‘Well, there is only two hands,’ blah blah blah blah. So that’s a bit of the unfortunate thing.” “Well, we have 18 patients and sometimes our patients are late or we’re short staffed, and we have patients that are quite demanding; they’re, like, ‘We have to do it now.’ And we know they don’t, but sometimes it’s just hard, you just don’t want to argue with your patients.” Q31 | IDE as an additional source of pressure (barrier) | | | |  |  |  |  |  |
|  | “Oh, [IDE] hasn’t been bad at all. As long as the patients are understanding that I can’t do it, like, right now, ‘cause I still have somebody else to put on, and most of them were pretty good about that.” Q28, | Shared understanding of how IDE is prioritised on dialysis unit (facilitator) | | | | Knowledge | | | | |
| 9. Painter *et al*. (2014) | | | | | | | | | | |
| Theme 1: Awareness of KDOQI Physical Activity Guideline | Most of the nurses were aware of the KDOQI guidelines, but none was aware of the specific guideline related to physical function and physical activity. None of the PCTs was aware of the guidelines | Staff lacking of knowledge of guidelines | | | | Knowledge | | | | |
|  | After learning of the KDOQI physical activity guideline, all interviewees stated that their patients were sedentary as a rule. | Used to being sedentary | | | | Behavioural Regulation | | | | |
|  | However, they thought regular physical activity was a good idea, particularly given their experience with an exceptional patient in their clinic: “We have a young gal (in her 30s) that works during the day and comes in for dialysis in the evening. Then she goes and plays tennis. She’s ideal. She’s always upbeat and doesn’t seem to have the problems others do. We have to change her dialysis schedule so she can go to tournaments.” | Observing/feeling the benefits | | | | Reinforcement | | | | |
| Theme 6: How Change Might Occur | Several staff had ideas about ways to incorporate physical activity into existing routines. The PCTs thought that they had the time to provide education and encouragement for physical activity, although they needed education and resources. “We are busy but we have downtime too. So if I had to once a month, once a week or whatever sit with each patient in my pod for 5 minutes that’s manageable,” said one PCT. “There’s plenty of opportunity to sit down and visit with them, but I wouldn’t do it unless I knew what I was talking about,” stated another. Ideally, “it would be a group job,” explained another staff member, with everyone who interacted with the patient “assessing their physical function, walking in the building, transferring.” “I don’t think encouraging somebody to do something takes up their time necessarily. It would probably have a better impact on the patients. I don’t see how it would negatively affect the staff but I can see that maybe some of the techs would have a problem with just...feeling overburdened.” “We’re staffed pretty thin and we’re pretty busy so I almost think it would need to be maybe an offsite or—yeah, offsite physical therapist who comes in and assess[es] the patients and says realistically this is what you could start doing and we could see how you do with that and go from there because honestly, right now the way we are, we don’t really have time which is sad but it’s reality.... Then we would try to follow up, say ‘hey, have you been doing some exercise? What have you been doing? How’s it going? Are you having problems?’” | Staff having time to support IDE (facilitator) | | | | Environmental Context and Resources | | | | |
|  |  | Existing resources do not include physical activity/exercise guidance (barrier) | | | | Environmental Context and Resources | | | | |
|  |  | Additional, exercise specific staff (facilitator) | | | | Environmental Context and Resources | | | | |
|  | One suggestion was to start with patients new to dialysis: “Newer patients would react to it better because they don’t know what to expect, unlike our long-term patients who like very few changes.” | Used to being sedentary (barrier) | | | | Behavioural Regulation | | | | |
|  | Building a new section into the dialysis orientation would not be difficult “as long as we’re sitting down talking to them and explaining things, incorporating physical activity” would be a natural addition. Another suggestion was to design a new place on the dialysis flow sheets for education. “In our assessment part of our flow sheet, just add little check boxes. Because on certain days we have certain things that we are supposed to teach them, and that could be like a oncea-week question.” One nurse asked for a new section to be added to monthly notes or care plans. The important point was making it a documented and routinized part of care. | Exercise monitoring/assessment/provision not included in care provision/ not the nurses’ role  (barrier) | | | | Social/Professional Role and Identity | | | | |
|  | Nurses thought that there was no time or opportunity within their tasks to take the time for encouraging physical activity or assessing physical function beyond what they do at the initiation of dialysis. However, each stated that they would support the PCTs in promoting and encouraging activity participation and thought it would be a good thing to consider as a team effort. | Nurses lack time for IDE due to other tasks | | | | Environmental Context and Resources | | | | |
|  |  | Staff view exercise as team effort (facilitator) | | | | Social/Professional Role and Identity | | | | |
| 12. Wodskou *et al*. (2021) | | | | | | | | | | |
| Patient Related Factors | The nurses pointed out that patients’ health and motivation, and the physical conditions of hemodialysis treatment, would affect IE. The nurses described patients in hemodialysis as a heterogenous group in terms of age, level of function, and morbidity. However, most patients were elderly with symptoms of chronic illness, such as chronic fatigue and poor general condition. Nurses felt that the patients’ general condition influenced their motivation for exercise. In addition, nurses considered the many restrictions patients had in daily life, particularly related to dietary and fluid intake, important to the type of physical activity each individual patient could manage and be motivated to undertake. Nurses reported that hemodialysis is “exhausting” and drains patients’ energy. They interpreted patients’ habitual choices to relax and sleep during the process as saving their energy for the things in life that really mattered to them. However, nurses mentioned several factors they thought could motivate patients to be more physically active during hemodialysis: expected benefits such as feeling more energetic and less fatigued, fewer side effects such as restless legs, fewer dietary restrictions, and improved quality of life. To maintain patients’ motivation, nurses felt that the exercise program should be individualized to the patients’ physical condition, manageable, and simple. They suggested that regular conversations between patients and the physiotherapist could help patients focus on the positive effects of exercise. They also mentioned that patients could motivate each other by exercising together or competing with or against each other. Finally, nurses felt that their professional role and long-standing relationships with patients were essential to their motivation to begin and continue IE. As one nurse put it, ”They do what we ask them to do . . . to a large extent”. Most hemodialysis patients have limited mobility due to the needles and tubes connecting them to hemodialysis machines. If they move a cannulated arm, the alarm is often triggered, requiring nurses to be in constant proximity. The nurses were very aware of limitations these physical conditions placed on IE. They reported that IE required “good access”, meaning a well-functioning fistula with blunt needles or a central or peripheral venous catheter, because sharp needles would increase the risk of vascular perforation. The space between beds is limited due to the presence of the hemodialysis machines, tubes, and power cords, requiring that care be taken when moving around. The nurses were concerned that the lack of available floor space, combined with physical exercise, could hinder their access to patients in case of an emergency. Nurses preferred IE to take place in bed with equipment such as elastic exercise bands, small ankle and hand weights, or bed bikes, with which several of the nurses had experience. Nurses also mentioned that restricted space and lack of privacy could decrease the motivation of more modest patients. | Poor physical condition | | | | Skills | | | | |
|  |  | Other health conditions | | | |  |  |  |  |  |
|  |  | Fatigue | | | |  |  |  |  |  |
|  |  | Differing capacities for exercise between patients | | | |  |  |  |  |  |
|  |  | Preference to rest during dialysis (rather than IDE) | | | | Intentions | | | | |
|  |  | Observing/feeling the benefits | | | | Reinforcement | | | | |
|  |  | Improvement in energy levels | | | |  |  |  |  |  |
|  |  | Encouragement from patient peers | | | | Social Influences | | | | |
|  |  | Positive sense of competition between patients | | | |  |  |  |  |  |
|  |  | Tailored physical activity (by healthcare professional) suitable for dialysis patients | | | |  |  |  |  |  |
|  |  | Recommendations of healthcare professionals | | | |  |  |  |  |  |
|  |  | Patient encouragement from staff | | | |  |  |  |  |  |
|  |  | Local environment risks | | | | Environmental Context and Resources | | | | |
|  |  | Reduced access to patients in emergency if IDE equipment in the way | | | | Environmental Context and Resources  AND  Beliefs about Consequences | | | | |
|  |  | Lack of privacy in IDE | | | | Environmental Context and Resources | | | | |
|  |  | Exercise that can be done in bed | | | | Environmental Context and Resources | | | | |
|  |  | Staff feel able to motivate patients | | | | Beliefs about capabilities | | | | |
|  |  |  | | | |  | | | | |
| Nurse Routines | Nurses described their work in the clinic as characterized by routines, such as starting and ending hemodialysis, checking blood test results, and administrating medications. IE would have to fit into their accustomed routines, but the degree of required fit would depend on how involved they needed to be. If the nurses were to be involved, they wanted IE to begin after they had started dialysis on all their patients, checked blood tests, and administered medications. To save time, all exercise equipment needed to be user friendly and easy to obtain and clean. The nurses felt that less involvement would be better, for example, if patients could begin their exercise independently, and its timing in relation to hemodialysis routines would matter less. Similarly, if patients had personal exercise equipment that they could keep in their lockers, nurses would not have to make time to obtain and clean equipment. | IDE could disrupt the routine on the dialysis unit | | | | Environmental Context and Resources | | | | |
|  |  | Exercise monitoring/assessment/provision not included in care provision/ not the nurses’ role | | | | Social/Professional Role and Identity | | | | |
|  |  | IDE equipment that is easy to move and maintain | | | | Environmental Context and Resources | | | | |
|  |  | User friendly IDE equipment for patients to use | | | |  |  |  |  |  |
|  |  | When patients can retrieve their own equipment for IDE and monitor themselves/low reliance on staff | | | | Environmental Context and Resources | | | | |
|  |  | Ongoing access to rehab equipment | | | |  |  |  |  |  |
| Nurses’ Motivation for Intradialytic Exercise | In general, all nurses viewed IE positively. They agreed that exercise would contribute to patients’ physical and mental well-being and, particularly, their quality of life. These benefits were their primary motivation for supporting the intervention. However, nurses felt it was unrealistic for all patients to do IE, and some thought that patients doing it while standing posed too great a risk. As one nurse argued, “I wouldn’t dare.” Another nurse agreed: “The standing and jumping and getting out of bed . . . I just can’t see it”. Some nurses had experience with hemodialysis patients exercising by using bed bikes or participating in between-treatment “walking teams”, but their opinions of IE were primarily based on their knowledge of pathophysiology and the physical conditions of hemodialysis and their views on how an extra task would affect their already busy workdays. Nurses agreed that they lacked knowledge about the effects and possible negative consequences of IE and asked for information on the subject. They needed to feel certain that it would not endanger patients by, for example, overlooking contraindicating comorbidities. Therefore, they wanted the physician to approve individual patients’ participation in an exercise program. Nurses viewed involving a physiotherapist in the development of individual IE programs as crucial to successful implementation. Specifically, they suggested that the physiotherapist, in collaboration with the patient and nurse, develop the exercise program, help the patient get well under way, and regularly follow up to adjust the program as needed. Nurses would have no role in the initial introduction, which they viewed as a barrier because of the time required. They perceived their role as primarily motivating, following up on patients’ progress, and helping to address any difficulties. One nurse noted that management support, enthusiasm among nursing colleagues, and a pilot test on a small group of patients, were needed to maintain their motivated support for IE in a busy clinic. Additionally, nurses reported that they could only give low priority to IE if unexpected events occurred in the clinic. | Percieved benefit to physical well-being | | | | Beliefs about consequences | | | | |
|  |  | Perceived benefit to mental well-being | | | |  |  |  |  |  |
|  |  | Positive beliefs about physical activity (long-term benefits) | | | |  |  |  |  |  |
|  |  | General fears about safety | | | | Beliefs about consequences | | | | |
|  |  | Exercise not a priority for nurses | | | | Environmental Context and Resources | | | | |
|  |  | Staff knowing which patients are safe to take part in IDE and where IDE is contraindicated | | | | Knowledge | | | | |
|  |  | Offering a pilot trial of IDE | | | |  |  |  |  |  |
|  |  | IDE provided by non-nurse staff | | | |  |  |  |  |  |
| 13. Castillo *et al*. (2021) | | | | | | | | | | |
| Knowledge, skills and expectations:  Risks and benefits for staff and patients  Most believed IDE was associated with a variety of health benefits  A few expressed doubts over benefits  Many raised concerns over risk of injury | *No quotes from staff, subcategories coded where information allows* | | Perceived benefit to physical well-being | | | | | Beliefs about Consequences | | |
|  |  |  | Concern injury and accident | | | | | Beliefs about Consequences | | |
|  |  |  | Doubts about benefits | | | | | Beliefs about Consequences | | |
| Knowledge, skills and expectations:  Insufficient skill and knowledge to create and oversee exercise plans  Staff questioned what factors precluded exercise  Staff believed they lacked the necessary skill and training to oversee exercise plans | ‘I think just the plan…what the expectations would be in terms of the exercise? So how they’re gonna progress the first few weeks or what the intention is?…what’s an absolute contraindication to the study? What’s something that we can modify? We would certainly need that information’. – Trish, RN | | Lack of guidance/structure for patients | | | | | Knowledge | | |
|  |  |  | Staff knowing which patients are safe to take part in IDE and where IDE is contraindicated | | | | |  |  |  |
|  |  |  | Exercise monitoring/assessment/provision not included in care provision/ not the nurses’ role | | | | | Social/Professional Role and Identity | | |
|  |  |  | Staff not knowing what is right to instruct | | | | |  | | |
| Knowledge, skills and expectations:  Assumptions about patient eligibility  Staff raised questions regarding eligibility criteria and contraindications  Both staff and patients believed people who were older, frail and with limited mobility were unlikely to be eligible for IDE  One staff participant believed assumptions about eligibility may lead to missed opportunities | ‘It depends on the patient but I think if I had a patient that I knew was having a lot of angina or I needed to intervene with and do ECGs on a regular basis I’d probably say that’s somebody I don’t think should exercise. But is that really true?’ – Molly, RN  ‘Usually the mobile patient is offered first, right, the people, the patients that walk in. The people that come in in wheelchairs and so forth are, you know, assessed by physio and, and that sort of thing. And then the ones that we know that tend to drop their blood pressure during dialysis are not offered. But usually the ones that walk in, you know, independently the younger patients’. – Susy, RN | | Staff knowing which patients are safe to take part in IDE and where IDE is contraindicated | | | | | Knowledge | | |
| Knowledge, skills and expectations:  Assumptions about patient interest  Staff and patients believed few eligible patients would be interested in IDE | ‘People just want to come in either turn on the TV or come in and have a nap. So, it’s a smaller percentage of patients that really want to participate while they’re on dialysis’. – Jane, manager  ‘Oh I think the exercise thing is awesome but I think it will be a bit of a challenge to get some patients to agree. There’ll be patients there’ll be a handful of patients that will be quite willing, but there’ll be some patients that we’ll need to persuade a little bit … But it’ll be challenging for sure looking at our group’. – Shelby, RN | | | | Lack of motivation | | Intentions | | | |
|  |  |  |  |  | Lack of ‘buy in’ from staff | | Social influences | | | |
| Knowledge, skills and expectations:  Identifying potential candidates  Some staff relied on patients to self-refer | ‘I guess if I saw value in it I would suggest it to a patient like patients who feel that they have restless legs or patients complain of other things, like being bored. I might say why don’t you try the cycling program? Typically, though, the patients ask us. I have to admit I’m probably not as good at suggesting it as I am at supporting it if someone asks’. – Molly, RN  ‘So for now it’s still on a voluntary basis so if any patients see the other patients exercising they can approach me or the nurses. Otherwise sometimes the doctors will refer them to me’. – Tina, exercise professional | | | | Observing/feeling the benefits | | Reinforcement | | | |
|  |  |  |  |  | Staff rely on patients to self-refer to take part in exercise | | Environmental Context and Resources | | | |
| Category 2: human, material and logistical resources  Concerns about workload  Participants believed an IDE intervention would significantly increase staff workload and would be difficult to prioritize  Perceptions of workload may be influenced by past experiences  Many believed nursing staff would be resistant to more work  A few believed workload may lessen | ‘I think that staff are already very, very busy and to throw something else at them I think will get an emotional response. There’ll be resistance based on workload’. – Penelope, manager  ‘…all you need is one of those patients to have a line that doesn’t work, a fistula that’s acting up or somebody that’s unwell and your whole day goes to hell in a handbag. So having to drag somebody’s physio equipment on top of it, you know, it’s just kind of one more thing to a never-ending list. But in that particular instance it’s not gonna get done because the priority is the sick patient, a line that doesn’t work’. – Amy, RN  ‘…while the patient’s actually dialysing there is downtime so that’s there’s opportunity for the staff to step in there and set up the equipment have the conversations with the patient. So I can’t really see it being an extra workload for anybody’. – Elizabeth, manager | | | Nurses lack time for IDE due to other tasks | | | | | Environmental Context and Resources | |
|  |  |  |  | Exercise not a priority for nurses | | | | |  | |
|  |  |  |  | Lack of ‘buy in’ from staff | | | | | Social Influences | |
|  |  |  |  | Staff having time to support IDE | | | | | Environmental Context and Resources | |
| Category 2: human, material and logistical resources  Need for exercise professionals  Many participants believed exercise professionals should take responsibility for IDE to address workload concerns  One participant shared how nursing staff were resistant to IDE even when an exercise professional was present | ‘Yeah that model [with physiotherapists] would be I think the better than just leave it for the nurses if there’s just one person comes and set it up and it would be some, it would be some resource accessible to the nurses too. They’re just not leaving the nurses with some kind of extra work to be done yeah’. – Sarah, RN  ‘So I think there was a lack of knowledge from the beginning on the nursing staff and a lot of resistance. So, you know, the fact that I was touching their patients I think that was a huge issue at the beginning’. – Tina, exercise professional | | | | IDE provided by non-nurse staff | | | | | Environmental Context and Resources |
|  |  |  |  |  | Lack of ‘buy in’ to IDE from staff | | | | | Social Influences |
| Category 2: human, material and logistical resources:  Space and equipment  Units differ in space requirements for bike storage and movement  Biking equipment must fit dialysis chair/beds and be easy to move, use, maintain and clean | ‘…if we have several bikes that are being used at a time it creates more equipment that could be tripped over or if we have someone who’s unstable and we had to call a code it’s just more equipment that could be in the way’. – Maddie, RN  ‘We’d have to wait until all the patients are on because the nurses are moving around and patients are coming and going and we don’t want anything on the floor to be in their way. So we’d have to wait until they were all on’. – Stephanie, unit aide  ‘[And] the cleaning of those things too. Like okay, if this thing comes off you can just put this thing back in or if this thing is loose you can tighten it from here or if this thing is making noise we can put oil here. So, those are some small things we need to know and that way we can run this program smoothly’. – Afim, unit aide  ‘I think the biggest trouble that I’ve had is logistics… is the right patient on the right bed for their session?…if you’re on the wrong bed I can’t bike with you’. – Edward, exercise professional | | | | Reduced access to patients in emergency if IDE equipment in the way | | | | | Environmental Context and Resources  AND Beliefs about Consequences |
|  |  |  |  |  | IDE could disrupt the routine on the dialysis unit | | | | | Environmental Context and Resources |
|  |  |  |  |  | Staff having practical knowledge of how to facilitate and support IDE | | | | | Knowledge |
|  |  |  |  |  | Where IDE has specific logistical needs | | | | | Environmental Context and Resources |
| Category 3: social dynamics of the unit  Champions    Champions are important enablers of IDE    Nephrologists were believed to be instrumental in supporting practice changes    Nurse champions were seen as necessary for supporting practice changes at the bedside and encouraging patients to cycle, though many suggested it would be difficult to recruit nurse champions    Patient champions were believed to encourage other patients, though not all patients would welcome a peer champion | ‘…it would need to be the physiotherapist and somebody from the administration side …then one of the physicians as well. So having at least one physician champion would be very helpful as well’. – Nurse, manager    ‘And having the physicians onboard too and maybe them coming to talk to the staff about it is another one’. – Veronica, RN      ‘I think it’s a good idea. But I don’t know… Just like from my time as charge nurse and then the coordinator saying, you know, get somebody on this, get somebody on this and never would you have anybody volunteering for things’. – Lou, RN | | | | Physician involvement | | | | | Social influences |
|  |  |  |  |  | Exercise champions | | | | |  |
|  |  |  |  |  | Nurses lack time for IDE due to other tasks | | | | | Environmental Context and Resources |
| Category 3: social dynamics of the unit  Patient stories to ignite motivation    Patient stories and experiences are highly influential and can motivate staff and patients to engage in IDE | ‘And then the other side would be patient stories. Any patients that have had the intradialytic exercise and what it means to them and the benefits of it…. If you’ve got some of that I think that would help’. – Polly Anna, manager    ‘I guess the feedback from the patients that they’re enjoying it. If it becomes important to the patient, then it becomes important to me’. – Lou, RN    ‘…if the patient’s excited about it it’s gonna be a lot harder to say no we’re not doing that for you’. – Trish, RN | | | | Inspiration from peers | | | | | Social Influences |
|  |  |  |  |  | Staff observing patient enjoyment | | | | | Social Influences/Reinforcement |

## Splitting of existing themes - Physician Data

**Data analysed from written text not just quotations*
Papers including physician perspectives:*

## Facilitators Physician

| Original barrier  /Study | Data | New barriers (after splitting) | TDF domain mapped to |
| --- | --- | --- | --- |
| 8. Jhamb et al. (2016) | | | |
| Theme 1: Knowledge and perceived benefits of exercise Jhamb et al. (2016) | “Improved sense of well-being, improved mood, and I think that would be a big quality of life improvement for dialysis patients” (PHYSICIAN) | Perceived benefit to mental well-being | Beliefs about consequences |
|  | “One of the biggest morbidities of dialysis is cardio-vascular so improving cardiovascular health……when patients have very low blood pressure [during HD treatment] sometimes it [exercise during HD] helps keep the pressure up” (PHYSICIAN) | Knowledge about the benefits of physical activity | Knowledge |
|  | “Help strength and balance and help them recover if they were ever in a situation where they would fall” (PHYSICIAN) | Knowledge about the benefits of physical activity | Knowledge |
|  | “Helps release endorphins and makes people feel better overall” (PHYSICIAN) |  |  |
|  | “Exercise is very good for helping people to deal with energy level” (PHYSICIAN) |  |  |
|  | “Sometimes it can help relieve muscle cramps because a lot of these patients have cramps” (PHYSICIAN) |  |  |
|  | “ It helps keep the weight down because a lot of the dialysis patients you know they’re diabetic they could be obese so that helps a lot” (PHYSICIAN) |  |  |
|  | “May allow them to remain independent longer” (PHYSICIAN) |  |  |
|  | “ In some ways I think you would be better off [exercising during HD], because the thing I worry about with exercise is are you releasing potassium into the blood because of the exercises.” (PHYSICIAN) | Safety benefits of IDE | Environmental Context and Resources |
|  | “A lot of these patients they have very sedentary lifestyles and won’t leave home and a large portion of them I would think for them just coming to dialysis is their only you know trip of the outside world, know give them a sense of participating in something [exercise during HD]” (PHYSICIAN) | Camaraderie and normalcy in the Unit | Social Influences |
|  | “If the whole team sort of supported them trying it then I think some of them would do that” PHYSICIAN | Patient encouragement from staff | Social Influences |
|  | “There is such a time thing for dialysis itself, it would be nice to somehow turn that time into productivity. I think accessibility would be good… you might have more compliance because you’re stuck there for four hours” PHYSICIAN | Exercise at dialysis centre/unit | Environmental Context and Resources |
|  | “Maybe in the beginning offering some sort of encouragement or incentive chance whether it’s you know they can have TV or something …. maybe a raffle or … a prize or something. That’d be key to get them motivated to want to do it.” PHYSICIAN | Patients having extrinsic goals | Goals |
| 12. Zelko et al. (2023) | | | |
| The benefits of intradialytic exercise: Improvements in patients’ physical functioning and musculoskeletal structure | All participants expressed one or more benefits related to improvements in muscle strength or muscle mass. Three-quarters believed that IDE increased patients’ muscle strength, and one-quarter expressed benefits related to gains and maintenance of muscle mass volume. A minority reported improvements in muscle endurance, flexibility and fine motor skills.  “I think it (exercise) maintained a certain conditioning, a certain quasi-muscle status of the patient. Because physical activity affects not only muscular, but also bone structures.” [nephrologist, m, 45y, 16 ype] | Perceived benefit to physical well-being | Beliefs about consequences |
| The benefits of intradialytic exercise: Improvements in patients’ psychosocial functioning | Nearly all participants believed that IDE positively affected patients’ psychosocial functioning. In their view, patients in standard dialysis regimens are highly vulnerable, resigned, self-regretful, depressive, grieving, wrathful, beaten inside and socially isolated. Participants expressed that IDE could change patients’ self-perception, make patients happier, less stressed, depressed and anxious, and contribute to positive attitudes and co-activation. | Staff recognition of the benefits (general) of IDE | Beliefs about consequences |
|  |  | Perceived benefit to mental well-being |  |
| The benefits of intradialytic exercise: Improvements in patients’ clinical profile and quality of therapy | The majority of participants believed that IDE could improve (to a certain and individual extent) the clinical profile of haemodialysis patients. The most frequently reported benefits were improvements in glucose metabolism, better cardiac and vessel health, a decrease in overhydration and lower morbidity and mortality of patients due to improvements in their nutritional status.  “It (exercise) could improve nutrition status and prospectively, maybe also the morbidity and mortality of patients.” [nephrologist, m, 44 y, 20 ype] | Positive beliefs about physical activity | Beliefs about consequences |
|  |  |  |  |
| The benefits of intradialytic exercise: Improvements in patients’ independence and self-efficacy | Most participants believed that IDE could improve patients’ self-efficacy in activities of daily life, decrease their dependence on help with basic needs from caregivers and family members and improve the level and safety of patients’ mobility.  “So, maybe they started to believe in themselves, that simply ‘I can do it, I can walk downstairs, I can go shopping, my son doesn’t have to go shopping for me’, right?” [nephrologist, m, 43 y, 16 ype]  “Relatives didn’t need to take as much care of patients; they didn’t need to take care of transporting patients. They (patients) are more independent regarding daily living.” [nephrologist, m, 43 y, 19 ype] | Maintain independence | Beliefs about consequences |
| *The benefits of intradialytic exercise: Alterations in patients’ perception of time spent in dialysis treatment* | *According to most participants, haemodialysis patients qualify time spent on dialysis as “boring”, “useless” and “tiresome and endless”. Half of the participants stated that IDE could alter patients’ perceptions of dialysis time and thus relieve their stereotypic feelings about the dialysis routine.* | *Not counted for physician as colourmap for paper indicated that it was not mentioned by that staff group* |  |
| The benefits of intradialytic exercise: Distractions of patients’ minds from feelings of physical and psychological discomfort | One-third of participants believed that IDE led to the distraction of patients’ negative thoughts about nephrology diseases, causes and causality of their health problems and further consequences during disease progression.  “(During exercise) They started to speak about totally different things than usual, a little bit changed their minds.” [nephrologist, m, 43 y, 19 ype]  “It’s also a change in their (patient’s) mind, because he started already thinking of other things, such as whether he had good blood flow, or whether he had good blood pressure, etc., so he’s disengaged. Physical activity distracted them partially from all of that stress.” [nephrologist, m, 68 y, 40 ype] | Perceived benefit to mental well-being | Beliefs about consequences |
| 18b. Young *et al*. (2015) (post implementation of IDE) | | | |
| Positive outcomes of participating in IDE Young et al. (2015) | “As a doctor working on a dialysis unit it can sometimes be fairly bleak in that dialysis is a very good treatment for keeping people alive but doesn’t always enable people to live. I think if there is a treatment that makes [patients] feel better that makes you feel a whole lot better about what you do to people.” (Consultant Nephrologist). | IDE could improve experience of dialysis | Beliefs about consequences |

## Barriers Physician

| Original barrier  /Study | Data | New barriers (after splitting) | TDF domain mapped to |
| --- | --- | --- | --- |
| 8. Jhamb *et al*. (2016) | | | |
| Theme 2: Reported barriers to exercise: dialysis makes exercise challenging | “People get really fatigue while on Dialysis and some of them complain of feeling terribly tired” | Fatigue | Skills |
|  | “A lot of our patients they have Diabetes and they’re old and they can’t see very well and can’t hear very well. They can’t feel their feet very well because they have Neuropathy” | Poor physical condition | Skills |
|  | “So yeah there is a fear component ...” | *Not coded as insufficient detail in quote* |  |
|  | “and there is a lazy component too.” | Lack of motivation | Intentions |
|  | “I have a patient who has Osteoporosis and she’s fallen and broken a hip and now she’s not in to any exercises. …so yeah there is a fear component” | Fear of falling | Beliefs about consequences |
| Theme 3: Reported barriers to intra-dialytic exercise: intra-dialytic exercise should be safe without disrupting usual care | “How do you interpret high blood pressures when you’re exercising? I don’t think you can. So it does create some issues that would have to be [addressed]” | General fears about safety | Beliefs about consequences |
|  | “People passed out on Dialysis. If they are doing something else that’s directing blood flow elsewhere again it’s just sort of not an ideal combination. If someone’s inclined because they tend to drop their blood pressure, if you’re going to make them exercise you’re going to exacerbate [hypotension]” |  |  |
|  | They’re almost like sort of tied up to the machine. one hand is immobile so that pretty much you know they just have the use of one hand and we don’t like the patients to be really moving their arm too much because then the needles would move and there is chances of them infiltrating | IDE could disrupt dialysis | Environmental Context and Resources |
|  | “It would be a little bit expensive because I guess it would involve some sort of equipment.” | Lack of resources |  |
|  | “Nurses and techs, they’re on their toes all the time, they are busy and there’s the machines are alarming, someone you know needs attention all the time, and they are not like over-staffed, they are under-staffed so this would be something extra” | IDE as an additional source of pressure |  |
|  |  | Nurses lack of time due to other tasks |  |
|  | “There are so many patients per nurse, how attentive can they be when there's another variable in play” | Nurses lack of time due to other tasks |  |
|  | “It not really part of the culture of during Dialysis….. some of them have been on Dialysis for a long time and they’re used to their routine and they come in and they do whatever they do and ….. I’m not sure that would be completely easy to change…its going to be an uphill battle to get them to exercise right now … they have their routine” | Lack of motivation | Intentions |
|  | “A lot of the patients are not receptive to new ideas …..it’s going to be a big change” |  |  |
| 12. Zelko et al. (2023) | | | |
| The risks of intradialytic exercise: Exercise-related damage of vascular access and injury to the adjacent anatomical area | Three-quarters of participants believed that IDE could cause vascular access damage or malfunction and could injure the adjacent anatomical areas. The most frequently reported concerns regarded piercing and rupture of fistula, and pulling out the catheter and needles during haemodialysis therapy.  “Either that arm was fixed, or in some way the patient was warned not to do anything with the hand, right, even if they were lifting the ball or (exercise) with rubber bands, or so. So, there’s a risk of vascular access (damage). And also same for catheters.” [nephrologist, m, 43 y, 19 ype]  “Risks could be related to vascular access. Patients, who have fistulas, sometimes must maintain a ‘forced’ position (of the upper extremity) and sometimes such a concentration on exercise could distract them from this position, and this certainly wouldn’t be favourable, because rupture of the vessel could occur.” [nephrologist, m, 45y, 16 ype] | Fear of fistula damage | Beliefs about consequences |
| The risks of intradialytic exercise: Insufficient individualization of IDE progressivity regarding patients’ physical condition | Half of the participants believed that insufficient individualization of training progress could increase the health risks of IDE and discourage patients from further exercising. They are worried that improper control and management of IDE could worsen their nephrology, cardiovascular, endocrine, neurology or neuromuscular diseases. Participants stated that the rate of progressivity should be individual and highly depended on the patients’ physical functioning, chronological and biological age, gender and other specific health conditions.   “Like I said, it (exercise) is limited by the type of vascular access used; you need to individualise (training).” [nephrologist, m, 43 y, 19 ype]  “They (patients) frequently had fractures; an ordinary fall could cause huge haematoma in these patients. So there is a need to approach the patient individually, what diagnoses he has, what the patient’s age is and what the patient can safely do (during exercising).” [nephrologist, m, 45 y, 16 ype] | Concern injury and accident | Beliefs about consequences |
|  |  | General fears about safety |  |
|  |  | Tailored physical activity (by healthcare professional) suitable for dialysis patients | Social Influences |
| The risks of intradialytic exercise: Exercise-related injuries of musculoskeletal apparatus | One-third of participants expressed concerns that IDE could contribute to musculoskeletal injuries in haemodialysis patients. They believed that exercise during dialysis could contribute to tendon and/or muscle injuries. Furthermore, participants expressed the opinion that a combination of training-related injury with dialysis- and exercise-related fatigue could increase risks of falls and injuries in patients.  “They are heparinised; it’s important to avoid hitting, to minimise the creation of bruises. Also, avoid tendon injuries caused by sharp (extremities) movements. So basically to avoid injury because of exercise.” [nurse, f, 54 y, 36 ype]  “Maximally, I think that if the patient overloaded exercising, he could suffer injuries to muscles. When the patient is a little overwhelmed, he feels pain and muscle strain, but that’s nothing serious (for his health).” [nurse, f, 46 y, 23 ype] | Concern injury and accident | Beliefs about consequences |
|  |  | Fear of falling |  |
| The risks of intradialytic exercise: Insufficient understanding of patients’ clinical conditions and health-related risks | Some participants believed that insufficient consideration of patients’ actual clinical conditions by training assistants could increase health-related risks during IDE. Participants reported that patients are generally affected by musculoskeletal, cardiovascular and infectious disease risks and frequently experience acute clinical deterioration. They expressed that insufficient review and consideration of patients’ actual conditions prior to exercise session could negatively affects manifested diseases or hidden health risks in patients.  It (exercise) could cause deterioration of patient’s general condition – his basic disease; we had cardiac, hypoglycaemic and patients with other serious diseases. These need to be somehow monitored. If a patient’s condition deteriorated, exercise must be stopped.” [nephrologist, m, 43 y, 19 ype] | Poor physical condition | Skills |
|  |  | Concern injury and accident | Beliefs about consequences |
| The risks of intradialytic exercise: Application of IDE early after initiation of haemodialysis therapy | A minority of participants believed that IDE could be risky for patients with acute kidney injury and patients early after initiation of maintenance dialysis. This could imply that these patients would be emotionally and physically overloaded by additional activity during the haemodialysis therapy. Participants preferred a cautionary approach to these groups of patients and postponement of IDE until the patient had established a dialysis regimen.  “We have patients who we refer to as ‘acute’. These are problematic. These unstable patients just started their dialysis programme and among these patients exercise is problematic and risky.” [nephrologist, m, 45 y, 16 ype] | New patients overwhelmed by physical therapy requirement | Beliefs about capabilities |
|  |  | Exercise could be risky for unstable patients | Skills |
| The barriers to intradialytic exercise: Limitations related to psychosocial dispositions of patients | All participants believed that patients had a minimal history of regular physical activity and remained inactive during their lives with nephrology disease. Their sedentary lifestyle and beliefs that physical activity is not important for their lives were identified as major barriers on the patients’ side for initiating IDE. Participants expressed their opinions that these attitudes resulted from patients’ frustration, passive negativism, shame, introversion, social isolation, closure personality, self-pity, “self-abandonment” and resignation on nearly everything. The minority reported that patients were deeply depressed with minimal “self-initiative” efforts for any exercise behaviour change.  “Physical activity and psychological conditions are connected. And so, I would say they surrender and are very depressed.” [nephrologist, m, 43 y, 19 ype]  “I think they’re ashamed to exercise in front of other patients.” [nephrologist, m, 43 y, 19 ype] | Used to being sedentary | Behavioural Regulation |
|  |  | Detachment due to dialysis | Belief about Capabilities |
|  |  | Poor mental health | Skills AND Belief about Capabilities |
|  |  | Patients believe physical activity is optional | Goals |
|  |  | Lack of privacy in IDE | Environmental Context and Resources |
| The barriers to intradialytic exercise: Lack of financial resources for personnel capacities | One of the most dominant subthemes was related to financial resources for the implementation of IDE. Most of the participants expressed their uncertainties about covering direct costs, especially those connected with financing personal capacities. Even in a minimalistic setting, IDE needs to be supervised, controlled and reported by one additional person in the care-providing team in every dialysis session. Participants believed that these costs could be covered by funding from health and research government authorities or health insurance companies. However, most of the participants were sceptical about the actual will for systematic change in reimbursement of health care. The only hope reported was an initiation for adding exercise intervention into the categorization system of nephrology medical services in the Slovak Republic. The majority of the participants believed that material resources for IDE were negligible and could be covered directly by internal financial recourses of dialysis centres.  “To buy the necessary equipment, a few sets of bands and balls, that isn’t a problem. These cost a few (hundreds) of euros’ it’s minor, a one-time expense.” [nephrologist, m, 44 y, 20 ype] | Cost of exercise facilities/equipment | Environmental Context and Resources |
|  |  | Low cost material resources | Environmental Context and Resources |
|  |  |  |  |
| The barriers to intradialytic exercise: Limitations on the work capacity of care-providing teams at dialysis centres | Half of the participants reported that the implementation of IDE by training assistants produced minimal or no distraction of care-providing personnel and patients. Three-quarters of participants believed that nurses were not able to implement IDE due to their already high workload in monitoring and surveillance of patients during dialysis therapy. In their view, any additional task for nurses could negatively affect the quality of provided medical care. These participants also believed that IDE could be provided by other personnel of dialysis centres or as an external service for dialysis centres.  “Well, there should be some staff available to deal with it and to do it. Basically, our nurses are quite busy.” [nephrologist, m, 43 y, 19 ype] | Nurses lack time for IDE due to other tasks | Environmental Context and Resources |
|  |  | IDE provided by non-nurse staff | Environmental Context and Resources |
| The barriers to intradialytic exercise: Lack of support, information, motivation and guidance | One-third of participants reported that insufficient support from family is a barrier in IDE. About a quarter reported missing support from caregivers. In both groups, low accessibility to IDE-related information for caregivers, families and patients, and lack of motivation and guidance for patients was frequently mentioned as the barrier.  “We see this in our patients; if they have a good family background and support, they don’t surrender; they just try; they’re more physically active.” [nephrologist, m, 43 y, 19 ype]  “Once I noticed that exercising patients started to talk with other patients about exercise in the locker room, about how they feel good (after exercise), about how they like doing something with their muscles. Maybe this support could encourage them to exercise.” [nephrologist, m, 43 y, 19 ype] | Lack of support from social network | Social Influences |
|  |  | Low accessibility to IDE-related information for caregivers, families and patient | Knowledge |
|  |  | Lack of guidance/structure for patients |  |
|  |  | Lack of motivation | Intentions |
|  |  | The importance of patient’s family | Social Influences |
|  |  | Encouragement from patient peers |  |
| The barriers to intradialytic exercise: Limitations related to the organization of patients’ therapy at dialysis centres | Some participants reported different barriers related to the “work culture” at dialysis centres. These subjects believed that IDE in a dialysis centre setting could be time- and space-limiting for patients and care providers. If IDE is performed shortly after dialysis starts, it could collide with nurses and nephrologists communication with patients. If performed late, it could postpone patients’ disconnection from the dialysis device. Space limitations were linked to patients’ privacy and quiet environment needs. Participants reported that a specific group of patients preferred to be more isolated during exercise, while other non-exercising patients might perceive IDE during dialysis as “noisy” and “sleep-disturbing”.   “We have an open-spaced dialysis room and some patients are ashamed to exercise. They (patients) preferred a more isolated space for the time of exercise.” [nephrologist, m, 43 y, 19 ype] | IDE could disrupt the routine on the dialysis unit | Environmental Context and Resources |

## Originally mixed themes Physicians

| Original barrier  /Study | | | | Data | | New codes (after splitting) | | | TDF domain mapped to |
| --- | --- | --- | --- | --- | --- | --- | --- | --- | --- |
| 14. Castillo *et al*. (2021) | | | | | | | | | |
| Knowledge, skills and expectations:  Risks and benefits for staff and patients  Most believed IDE was associated with a variety of health benefits  A few expressed doubts over benefits  Many raised concerns over risk of injury | | | | ‘And so I think if they could get in an exercise program and hopefully get stronger in order to better prepare them for transplant I would be excited for that patient’. – Anne, MD  ‘So intradialytic exercise has been shown to improve certain patient symptoms like restless legs, for example. That’s the one that I believe may be true. There’s a bunch of other stuff that intradialytic exercise has been named to improve, like in terms of outcomes like depression, blood pressure, ultrafiltration, but I’m not sure I believe any of those other stuff, so maybe restless legs’. – Sam, MD | | Positive beliefs about physical activity (long-term benefits) | | | Beliefs about Consequences |
|  |  |  |  |  |  | Aiming to be healthy for transplant | | | Goals |
|  |  |  |  |  |  | Concern injury and accident | | | Beliefs about Consequences |
|  |  |  |  |  |  | Doubts about benefits | | | Beliefs about Consequences |
| Knowledge, skills and expectations:  Insufficient skill and knowledge to create and oversee exercise plans  Staff questioned what factors precluded exercise  Staff believed they lacked the necessary skill and training to oversee exercise plans | | | | ‘And it’s not just randomly pedalling without a clear goal or expectation. I think that’s the part where nurses, technicians and doctors even nephrologists don’t really have a lot of expertise in. And so, you know, if you just bring pedals to a unit and you say here you go that’s where you’re likely gonna fail because we lack that ability to really assess and tailor the programs to patients individually’. – Justin, MD | | Tailored physical activity (by healthcare professional) suitable for dialysis patients | | | Social Influences |
|  |  |  |  |  |  | Lack of guidance/structure for patients | | | Knowledge |
| Knowledge, skills and expectations:  Assumptions about patient eligibility  Staff raised questions regarding eligibility criteria and contraindications  Both staff and patients believed people who were older, frail and with limited mobility were unlikely to be eligible for IDE  One staff participant believed assumptions about eligibility may lead to missed opportunities | | | | ‘Yeah I think your biggest challenge, on the medical side, your challenge is making sure that we don’t, that doctors or nephrologists don’t make patients ineligible when in fact they might stand to benefit. I think that’s the biggest risk from a medical standpoint’. – Justin, MD | | Not knowing which patients are safe to exercise | | | Knowledge |
| Knowledge, skills and expectations:  Assumptions about patient interest  Staff and patients believed few eligible patients would be interested in IDE | | | ‘I think for certain patients who are keen to do it and are going to do it on an ongoing basis… unfortunately I don’t think there’s a lot of them…’. – George, MD | | | | Lack of motivation | Intentions | |
| Category 2: human, material and logistical resources  Need for exercise professionals  Many participants believed exercise professionals should take responsibility for IDE to address workload concerns | ‘And I think that somebody with exercise expertise who could transmit that and then you get into the champions idea so that there are people who know who have a better understanding of what’s going on. I think that would work best’. – Sue, MD | | | | | | IDE provided by non-nurse staff | Environmental Context and Resources | |
| Category 2: human, material and logistical resources:  Biking equipment must fit dialysis chair/beds and be easy to move, use, maintain and clean | | ‘The nurses don’t like having to move that bike around so they don’t encourage it.…the nurses don’t because frankly they don’t like having to lug it out and move it around. That’s my honest impression. [laugh]’ – Brian, MD | | | Manual handling challenges for staff | | | Environmental Context and Resources | |

## Splitting of existing themes -  Carer data

**Data analysed from written text not just quotations**

Barriers

| Original facilitator  /Study | Data | New Barriers(after splitting) | TDF domain mapped to |
| --- | --- | --- | --- |
| 2. Kontos et al. (2007) | | | |
| ESRD, its sequelae, and other comorbidities    Kontos et al. (2007) | “You need to have some time to recuperate from dialysis. As you know it’s exhausting, and it takes a while until you get over it, a few hours. I mean, overnight sleep is fine. But you find yourself in the morning exhausted, and still you want to sleep again. I don’t know about other people but that is how I feel.”, In reference to his wife who receives dialysis treatment, a family care provider similarly commented: “Dialysis saps your energy. The fatigue, oh the fatigue.” | Fatigue | Skills |
| 11. Sheshadri et al. 2024 | | | |
| Barriers to Promoting Pretransplant and Exercise  Activity for Their Patient (Care Partner)  Capability | Similarly to patients, care partners also reported that 1 major obstacle to promoting exercise and activity for patients was “a shortage of (exercise-related) information…” leading to a lack of understanding of what would be an appropriate regimen (Table 3).This lack of guidance may in part have been related to gaps in communication with the dialysis or transplantation care teams, resulting in missed opportunities for care partners to intervene, with some care partners saying “it didn’t make me feel like they care about what part I had to take in (the transplantation process)” or that they felt “lost.” | Lack of information sources for patients and staff | Knowledge |
|  |  | Lack of enough information support for patients | Knowledge |
|  |  | Lack of guidance/structure for patients | Knowledge |
|  |  | Carers not included in care plan | Social Influences |
|  | Another common barrier was care partner concerns with their own health, including not just physical impairment but also anxiety and stress. One care partner suggested that the anxiety was especially “taxing, and anything that is emotional will eventually manifest itself physically (in me).” A minority of care partners reported that they already had difficulty with patient adherence even outside of exercise or had tried to promote exercise but had no success due in part to difficulty with getting their patient to focus or be organized (Table S2). No individuals (either patient or care partner) reported weight as a barrier to exercise. | *Carer poor health* | *Not added to codebook as very specific to carer experience rather than patient experience* |
|  |  | *Carer poor mental health* |  |
|  |  | *Poor adherence by patient to current regime makes carer disinclined to promote exercise* |  |
|  |  | Patient lack of focus/disorganised | Memory, Attention and Decision Processes |
| Capability: Lack of guidance from patient’s care team | “I didn’t remember them saying, ‘Well, it’s really important you keep up your exercises and eat well,’ or whatever. They weren’t real specific.” (ID C2) “[On lack of support] I don’t feel personal assistance [from] a doctor telling me ‘…We’re going to try this and you’ll feel better.’” (ID C13) “They could [explain to] us the caregiving aspect… what do we look out for?” | Lack of enough information support for patients | Knowledge |
|  |  | Lack of guidance/structure for patients | Knowledge |
|  |  | Lack of guidance from healthcare professionals | Social Influences |
|  |  | Patients lack of knowledge of the benefits | Knowledge |
|  |  | Caregivers taught how to support | Knowledge |
| Capability: Concerns with care partner’s own health | “[On burden of care]: It’s continuous. Blood pressure goes up. Heart rate goes up…it’s taking its toll on me.” (ID C13) “… we’re a little bit more cautious. We know that if we fall, our bones will be a little bit more brittle.” (ID C15) | *Carer poor health* | *Not added to codebook as very specific to carer experience rather than patient experience* |
| Barriers to Promoting Pretransplant and Exercise  Activity for Their Patient (Care Partner)  Opportunity | A major theme among care partner responses was lacking enough time to promote patient exercise because of overall burden of time spent, such that with caregiving, bringing in income, and housekeeping they had 3 jobs and that because their patient started dialysis “everything’s gone downhill.” Caregiving was felt to be “rewarding, but…very taxing.” Similarly to patients, the dialysis schedule presented a major opportunity barrier toward exercise, with dialysis described as “all-consuming” and “deflat[ing]…joy in doing things.” Furthermore, for the minority of care partners living away from patients, COVID meant sharply reduced opportunities for interaction (Table S2). | *Carer lack of time to promote exercise* | *Not added to codebook as very specific to carer experience rather than patient experience* |
| Opportunity: Competing priorities or lack of time | “I have a lot of responsibilities to take care of… I’m kind of the go-to person in my family, extended family, and so I get dragged into a lot of stuff for mostly medical issues.”(ID C8) | *Carer lack of time to promote exercise* | *Not added to codebook as very specific to carer experience rather than patient experience* |
| Opportunity: Dialysis schedule limiting ability to exercise | “…he [sequesters] himself for… 12, 16 hours…? And maybe he wakes up in the morning… and the cycle hasn’t completed, so he’s stuck in bed for another however many hours.” (ID C21) “…on dialysis days he comes home and he’s…wiped out – functionally just exhausted.” (ID C23) | Fatigue | Skills |
|  |  | Patient lack of time due to dialysis | Environmental Context and Resources |
| Barriers to Promoting Pretransplant and Exercise  Activity for Their Patient (Care Partner)  Motivation | Motivational-level barriers to care partners promoting exercise or activity for patients included the perception that “if he doesn’t want to do it, he’s not going to.” Some care partners felt the patient was outright depressed and would tell them they were “tired of dialysis” or that dialysis “would certainly bring my mood down” and therefore they did not feel up to promoting anything other than the necessities of life. Similarly to patients, care partners also expressed concern about patient safety with activity or exercise, or that they “(worried) about (the patient) going on their own for walks” or felt that they had to serve as “the gatekeeper…(to activity)” to prevent the patient harming themselves inadvertently. | Lack of motivation | Intentions |
|  |  | Poor mental health | Emotion  AND Skills |
|  |  | Physical activity is optional | Goals |
|  |  | General fears about safety | Beliefs about consequences |
|  |  | Concern injury and accident |  |
| Motivation: Patient does not appear motivated or appears depressed | “He’s telling me that he can’t walk that far. His back starts hurting. So I just stopped asking” (ID C4) “The years grind on, and all the plans that he had…they have to basically die, right?… I can only imagine how it grinds on you.” (ID C21) | Pain during exercise | Reinforcement |
|  |  | Poor physical condition | Skills |
|  |  | Poor mental health |  |
| Motivation: Concerns about patient safety or ability | “He wanted to be able to walk [with his friend]… and I said, ‘Well, I would be concerned that he would trip.’” (ID C2) “He likes to work… but – sometimes I got to tell him, ‘You can’t be doing that.’” | Family concern of ability | Social Influences |
|  |  | Direct guidance not to exercise from family |  |

Facilitators

| 11. Sheshadri et al. 2024 | | | |
| --- | --- | --- | --- |
| Facilitators to Promoting Pretransplant and Exercise Activity for Their Patient (Care Partner): Capability | About a third of the care partners interviewed suggested that they would feel more capable of promoting exercise for patients if they had support in other ways, including psychosocial support. One care partner told us that “the bottom line is (to hear from their care team), ‘You’re not in this alone.…We’ll figure it out together’” | Exercise as part of routine care | Skills  AND Social/Professional AND Intention |
|  |  | Recommendations of healthcare professionals | Social Influences |
|  |  | Support from care partner |  |
| Capability: Direct support for care partner | “I had a doctor taught me how to take time for myself, so I kind of get up like a half an hour early in the morning and that’s my own just-me time.” (ID C10 | *Carer taking time for own health* | *Not added to codebook as very specific to carer experience rather than patient experience* |
|  |  | *Health professionals considering needs of carers* |  |
| Facilitators to Promoting Pretransplant and Exercise Activity for Their Patient (Care Partner): Opportunity | Most care partners were very favorable of joint participation in an exercise program, saying “it would be beneficial for both of us, not just physically, but emotionally.” Care partners also emphasized the importance of individualizing such a program: “Not one size fits all. I don’t believe in that.” Care partners were also in favor of other social support for exercise beyond a health coach or therapist, suggesting that working out with other patients could be beneficial but cautioning that “(patients)’ lives are already expensive.” Finally, most care partners were in favor of trying to incorporate exercise into the daily routine gradually, to avoid over-fatiguing the patient and allow for continued engagement. | Tailored physical activity (by healthcare professional) | Social Influences |
|  |  | Encouragement from peers | Social Influences |
|  |  | Integrating with daily tasks | Behavioural regulation, Environmental Context and Resources |
|  |  | Fear of further fatigue | Beliefs about consequences |
| Opportunity: Joint participation in individualized exercise program | “I like the idea of somebody monitoring…[being] accountable to someone other than myself.” (ID C6) “Instead of just throwing him into water, I should be in the water with him and show him ‘this is how you float.’” (ID C15) “If someone told her what her what needs to be met then…I would do it too… we’ll work out or go walking more or whatever the case is.” (ID C17) | Accountability partner | Social Influences |
|  |  | Exercising with care partner | Social Influences |
|  |  | Demonstration of the exercises | Knowledge |
| Opportunity: Incorporating into daily routine | “If I can’t make it to the banks, I make him walk so he can get the walking exercise.” (ID C14) “[His dietitian] recommended lifting weights. So…I said ‘I need detergent, please’… and he’ll go and get [the heavy bottle]. You can’t just [say] ‘Go lift some weights.’ They’re not going to do it.” (ID C15) | Active travel | Environmental Context and Resources |
|  |  | Integrating with daily tasks | Environmental Context and Resources |
| Facilitators to Promoting Pretransplant and Exercise Activity for Their Patient (Care Partner): Motivation | Most care partners expressed improving or maintaining functionality for both the patient and themselves as a primary motivator for promoting exercise and activity and  “critical for anyone who’s preop, pretransplantation, anything like that.” Furthermore, several care partners expressed that they could not “(help) others if I don’t take care of myself.” Similar to patients, care partners were also motivated to promote exercise in hopes of a successful transplantation. One care partner directly stated that “If you told him, ‘Hey, we need you to increase your activity, we’re about to give you a kidney,’ I’m sure he’ll be running around the blocks because that would be his motivation.” About half of the care partners emphasized that promoting exercise was in part making it enjoyable or “almost…like a game,” with most care partners also expressing that exercise was a route by which they could spend more meaningful time with the patient outside of caregiving, and that “when we feel self-enriched…we can do better jobs.” | Perceived benefit to physical well-being | Beliefs about consequences |
|  |  | Aiming to be healthy for transplant | Goals |
|  |  | Enjoyment | Emotion AND Reinforcement |
|  |  |  |  |
| Motivation: Improving or maintaining functionality for patient and care partner | “Why don’t we start going to the gym…so that we’re not both so…stagnant.” (ID C23) “I take care of myself to be able to take care of him.” (ID C4) “I wanted to make sure that I was…able to help take care of him as well as myself.” (ID C9) | *Carer taking care of own health to better support patient* | *Not added to codebook as very specific to carer experience rather than patient experience* |
| Motivation: Successful transplantation | “(The transplant) really motivated him to exercise on a daily basis…We just want to have a better hope and we pray to survive.” (ID C16) “I kind of want to see him on the other side of this…I don’t see him ending his life this way, being on dialysis…it’s not who he is.” (ID C10) | Aiming to be healthy for transplant | Goals |
|  |  | *Carer taking care of own health to better support patient* | *Not added to codebook as very specific to carer experience rather than patient experience* |
| Motivation: Enjoyability of exercise | “Every time that [our granddaughter] would come over, we’d go walk around the lake.” (ID C10) “…he works alongside [friends or family] so he can feel normal, like there’s nothing wrong.” (ID C19) “For someone who didn’t want to walk the dog at all…now that’s a guaranteed 3 times a day that he’s out walking for at least 30 minutes each time.” (ID C10) | Enjoyment | Emotion AND Reinforcement |
