# Supplementary material for: Using behavioural theory to explore barriers and facilitators to physical activity in haemodialysis patients: an updated systematic review of qualitative evidence
Source: Health Psychol Behav Med. 2026 Jul 27;14(1):2707668. doi: 10.1080/21642850.2026.2707668 (PMC13410551; doi:10.1080/21642850.2026.2707668)
Supplement: Supplemental Material — Supplementary_Material_4.docx [file RHPB_A_2707668_SM9112.docx]

## Supplementary Material 4

## Examples of ambiguous quotes and final domain allocations

| Quote | Code | Why it could be perceived differently | Final coding decision |
| --- | --- | --- | --- |
| My neighbour didn’t ask me out to exercise sometimes due to my slow walking pace” | Lack of physical ability | Slow walking pace is stated and presented as physical limitation and so coded to skills. However it could be considered ‘belief about capabilities’ as their belief is that they are slow, not necessarily fact. | Skills |
| “You need to have some time to recuperate from dialysis. As you know it’s exhausting, and it takes a while until you get over it, a few hours” | Fatigue | Fatigue is a well-known symptom of dialysis and presented as a physical barrier, therefore coded to skills. However it could be considered ‘belief about capabilities’ as the patient is expressing their own opinion. | Skills |
| “being the age I am, I’m 76 years old now, that doesn’t really put you in the frame of mind to start any marathon.”, | Belief that age limits capacity | Although the patient is 76 years old and this is likely to have some impact on their ability to exercise, the limitation is expressed as evaluation of their own ability and coded as ‘belief about capabilities’. However it could be considered ‘skills’ if age was interpreted as a physical limitation. | Belief about capabilities |
| “I was very active at one time and now I’m very tired. You can’t make your body do what you want it to do either all the time because you’re tired” | Fatigue | Patient presents fatigue as a physical limitation and so it is coded to ‘skills’. However it could be considered ‘belief about capability’ if the interpretation was instead that the patient did not belief they could make their body do what they want. | Skills |
| “‘I had to go to the ER, with chest pain again. So that week was a bust’.” | Poor physical condition | Having chest pain which precludes exercise is considered here as a physical limitation rather than one of belief and coded to skills. However it is possible that minor chest pain would not stop a patient from gentle walking for example, so this could have been coded to ‘belief about capability’. | Skills |
| “When it was first mentioned one of the nurses came to me and said they won’t let you do that. I said yes they will, why wouldn’t they? They won’t. And he ... didn’t seem too keen” | Staff belief that patients are not capable of IDE | As the staff comment appears to be a belief rather than a medical fact, based in opinion, this was coded to ‘beliefs about capabilities’. However this could be interpreted that the staff had knowledge that the patient wasn’t safe to exercise, which could be coded to ‘Skills’. | Beliefs about capabilities |
