# Supplementary material for: Using behavioural theory to explore barriers and facilitators to physical activity in haemodialysis patients: an updated systematic review of qualitative evidence
Source: Health Psychol Behav Med. 2026 Jul 27;14(1):2707668. doi: 10.1080/21642850.2026.2707668 (PMC13410551; doi:10.1080/21642850.2026.2707668)
Supplement: Supplemental Material — Supplementary_Material_5.docx [file RHPB_A_2707668_SM9105.docx]

Critically Appraisal Skills Program (CASP) checklist (Munabi-Babigumira et al., 2015).

| Citation | Q1 | Q2 | | Q3 | Q4 | Q5 | Q6 | Q7 | Q8 | Q9 | Q10 | Q11 | Q12 | Q13 | Q14 | Score |
| --- | --- | --- | --- | --- | --- | --- | --- | --- | --- | --- | --- | --- | --- | --- | --- | --- |
| Hu, 2024 | Y | | Y | Y | Y | Y | UN | Y | Y | Y | Y | Y | Y | Y |  | 13 |
| Zelko, 2024 | Y | | Y | Y | Y | Y | UN | Y | Y | Y | Y | Y | Y | Y |  | 13 |
| Wodskou, 2021 | Y | | Y | Y | Y | Y | UN | UN | Y | Y | Y | Y | Y | Y |  | 12 |
| Castillo, 2022 | Y | | Y | Y | Y | Y | Y | Y | Y | Y | Y | Y | Y | Y |  | 14 |
| Rothpletz-Puglia, 2022 | Y | | UN | Y | Y | Y | UN | Y | Y | Y | Y | Y | Y | Y |  | 12 |
| Huang, 2023 | Y | | Y | Y | Y | Y | Y | Y | Y | Y | Y | Y | Y | Y |  | 14 |
| Sheshadri, 2020 | Y | | Y | UN | Y | Y | Y | N | UN | Y | Y | Y | Y | Y | Y | 11 |
| Young, 2015 | Y | | Y | Y | Y | Y | Y | UN | Y | Y | Y | Y | Y | Y | Y | 13 |

Y, yes; N, no; UN, unclear

1. Is this study qualitative research?
2. Are the research questions clearly stated?
3. Have ethical issues been taken into consideration?
4. Is the qualitative approach clearly justified?
5. Is the approach appropriate for the research question?
6. Is the study context clearly described?
7. Is the role of the researcher clearly described?
8. Sampling method clearly described?
9. Is the sampling strategy appropriate for the research question?
10. Is the method of data collection clearly described?
11. Is the data collection method appropriate to the research question?
12. Is the method of analysis clearly described?
13. Is the chosen analytical approach suitable for addressing the research question?
14. Are the claims made supported by sufficient evidence?

From Li et al. Yongxin et al not included in current study.

Supplement 2 Quality assessment of included studies

| Citation | Q1 | Q2 | Q3 | Q4 | Q5 | Q6 | Q7 | Q8 | Q9 | Q10 | Q11 | Q12 | Q13 | Q14 | Sores |
| --- | --- | --- | --- | --- | --- | --- | --- | --- | --- | --- | --- | --- | --- | --- | --- |
| Yongxin^[25]^ | Y | Y | Y | Y | UN | Y | UN | Y | Y | Y | Y | Y | Y | Y | 12 |
| Sutherland ^[20]^ | N | Y | Y | Y | UN | Y | UN | N | N | Y | Y | Y | Y | Y | 9 |
| Heiwe ^[26]^ | Y | Y | Y | Y | UN | Y | UN | Y | Y | Y | Y | Y | Y | Y | 12 |
| Thompson ^[27]^ | N | Y | Y | Y | Y | Y | Y | N | UN | Y | Y | Y | Y | Y | 11 |
| Song Y^[21]^ | Y | Y | Y | Y | Y | Y | UN | Y | Y | Y | Y | Y | Y | Y | 13 |
| Sieverdes ^[28]^ | Y | Y | UN | Y | Y | Y | UN | N | UN | Y | Y | Y | Y | Y | 11 |
| Kontos ^[29]^ | Y | Y | Y | Y | Y | Y | UN | Y | UN | Y | Y | Y | Y | Y | 12 |
| Jhamb ^[19]^ | Y | Y | Y | Y | Y | Y | UN | Y | Y | Y | Y | Y | Y | Y | 13 |
| Painter ^[30]^ | Y | Y | Y | Y | Y | Y | Y | N | UN | Y | Y | Y | Y | Y | 12 |
| Liu ^[31]^ | Y | Y | Y | Y | Y | Y | UN | N | UN | Y | Y | Y | Y | Y | 11 |

Y, yes; N, no; UN, unclear.
