# Supplementary material for: Using behavioural theory to explore barriers and facilitators to physical activity in haemodialysis patients: an updated systematic review of qualitative evidence
Source: Health Psychol Behav Med. 2026 Jul 27;14(1):2707668. doi: 10.1080/21642850.2026.2707668 (PMC13410551; doi:10.1080/21642850.2026.2707668)
Supplement: Supplemental Material — Supplementary_Material_6.docx [file RHPB_A_2707668_SM9050.docx]

Barrier and Facilitator Thematic Analysis – Patient Data

**Facilitators**

| **TDF Domain** *(Corresponding to COM-B construct)* | **Facilitators in domain** *(Number of times each facilitator present****)*** | **Themes** *Description of theme* | **Facilitators in each theme** | **Supportive data** *(First author, year)* |
| --- | --- | --- | --- | --- |
| Knowledge  *Capability – psychological*  “An awareness of the existence of something” | Desire to acquire specific knowledge about exercise    Knowledge about the benefits of PA    Knowledge about the consequences of lack of PA    Education program/package for patients    Needing staff to have practical knowledge of how to facilitate and support IDE  Knowing what to expect of IDE to address apprehension  Patient education on benefits of exercise is key    Offering a pilot trial of IDE  Demonstration of the exercises  Curiosity and interest    Awareness of different exercise options including those that suited for limited mobility patients  Staff knowledge to reassure and encourage patients | **Having sufficient knowledge about how to perform PA**  *Where sufficient information and guidance around what to expect, how to perform and how to support PA is provided to staff and patients. Information which supports patients to take part in PA on a practical level* | Desire to acquire specific knowledge about exercise  Education program/package for patients  Needing staff to have practical knowledge of how to facilitate and support IDE  Knowing what to expect of IDE to address apprehension  Offering a pilot trial of IDE  Demonstration of the exercises  Curiosity and interest  Awareness of different exercise options including those that suited for limited mobility patients   Needing staff to have sufficient knowledge to reassure and encourage patients | “I quite enjoy [exercise]...and I tend to push things a bit...but I don’t know now whether that’s a good or a bad thing. I’d like to ask someone” (Young, 2015) |
|  |  | **Understanding the importance of engaging with PA**  *Where the benefits of participating and consequences of not participating in PA are understood by patients and staff. This drives patient motivation to take part and staff motivation to support* | Knowledge about the benefits of PA    Knowledge about the consequences of lack of PA  Education program/package for patients Patient education on benefits of exercise is key | “I mean the only way I’m going to get out of this chair is to get up and start moving around. If I sit here it’s just going to get harder and harder for me to do, so I have to try, I have to try” (Liu, 2020) |
| Skills  *Capability – psychological and physical*  “An ability or proficiency acquired through practice” | *No facilitators coded* |  |  |  |
| Social/Professional Role and Identity  *Motivation – reflective and automatic* | Sense of community not limited to dialysis    Guided by spiritual beliefs    Connecting with previous identity as an active person  Desire to be productive member of society    Ability to personalise activity to match personal preferences  Desire to not be seen as visibly sick | **Fostering a sense of community with other dialysis patients**  *Where patients view themselves as part of a community not limited to dialysis treatment, but which has a strong focus on PA* | Sense of community not limited to dialysis | “Like, you can ask us dialysis patients when we’re sitting waiting around for each other or when we’re dialyzing beside one another, it’s just something—another exciting thing that, yes, we have dialysis in common, but now this is a positive thing we have in common that we can talk to each other about and encourage each other with.” (Thompson, 2016) |
|  |  | **Being more than just a dialysis patient**  *Where patient sense of identity outside of dialysis provides motivation and strength* | Guided by spiritual beliefs  Connecting with previous identity as an active person  Desire to be productive member of society  Ability to personalise activity to match personal preferences  Desire to not be seen as visibly sick | “God makes it easy for me to move” (Liu, 2020) |
| Beliefs about capabilities  *Motivation – reflective*  “Acceptance of the truth, reality, or validity about an ability, talent or facility that a person can put to constrictive use” | Increased body confidence and sense of capability through technical instruction and support    Confidence in ability to maintain activity    Confidence in ability to perform activity    Seeing examples of dialysis patients doing exercise | **Being confident in capability for PA**  *Where patients have confidence that they are able to perform and maintain the activity* | Increased body confidence and sense of capability through technical instruction    Confidence in ability to maintain activity    Confidence in ability to perform activity    Seeing examples of dialysis patients doing exercise | “She [the kinesiologist] helped me with what level I should go to and what I could handle, and that way, I felt very good about that.” (Thompson, 2016) |
| Optimism  *Motivation – reflective and automatic*  “The confidence that things will happen for the best or that desired goals will be attained” | Positive attitude | **A positive attitude towards PA**  *Having a positive attitude and interest towards PA* | Positive attitude | ‘‘I enjoy exercising. I was always active. So the desire is still there and that’s what pulls me through the days that are a little tougher because I’m not so well.’’ (Kontos, 2007) |
| Beliefs about consequences  *Motivation – reflective*  “Acceptance of the truth, reality, or validity about outcomes of a behaviour in a given situation” | Perceived benefit to physicalwell-being    Perceived benefit to mental well-being    Positive beliefs about PA (long-term benefits)  Weight-loss    Improved self-image    Coping with stress    Maintain independence  IDE could improve experience of dialysis  Physical activity allows more water intake | **PA could improve patient quality of life**  *The patient believing that some aspect of their life could be improved through taking part in PA. This could be physical, mental or to do with lifestyle* | Perceived benefit to physical well-being  Perceived benefit to mental well-being  Positive beliefs about PA (long-term benefits)  Weight-loss    Improved self-image  Maintain independence  IDE could improve experience of dialysis  Coping with stress  Physical activity allows more water intake | ‘‘Keeps the blood pressure down, that’s the big thing. That’s why I exercise. And that’s what motivates me, otherwise I have to take the blood pressure pill everyday, but now my blood pressure seems to be holding up alright.’’ (Kontos, 2007)  “I don't have the energy if I don't walk. If I walk more often now, I will feel better.” (Huang, 2023) |
|  |  |  |  |  |
| Reinforcement  *Motivation – automatic*  “Increasing the probability of a response by arranging a dependent relationship, or contingency, between the response and a given stimulus” | Helping someone else is incentive to exercise    Observing/feeling the benefits    Improvement in energy levels    Enjoyment    Patient encouragement from staff    Instructor providing increased confidence and capability    Previous good experience of rehab support    IDE pleasant to pass the time on dialysis    Sense of pride and satisfaction from exercising  Recognition of loss of physical fitness    Feeling of accomplishment    Feeling guilty if exercise is missed    PA gives sense of purpose | **Perceived positive outcomes from doing PA**  *Where patients associate PA with positive feelings and this encourages them to repeat the PA* | Observing/feeling the benefits  Improvement in energy levels  Enjoyment  Previous good experience of rehab support  IDE pleasant to pass the time on dialysis  Sense of pride and satisfaction from exercising  Recognition of loss of physical fitness    Feeling of accomplishment    Feeling guilty if exercise is missed    PA gives sense of purpose  Helping someone else is incentive to exercise  Patient encouragement from staff  Instructor providing increased confidence and capability | “I’m going to continue on my own, because you [the exercise program] already gave me the tools to work with and I already could see what it does to my life and to my personal life, my personal self, my health life—I see what it does for me.” (Thompson, 2016)  “Last year on National Day, even though it was cold and rainy, I ran. I was running hot, sweating, and feeling very comfortable. Now I enjoy running more and more.” (Huang, 2023) |
| Intentions  *Motivation – reflective*  “A conscious decision to perform a behaviour or a resolve to act in a certain way” | Willingness to engage    Already attempting exercise    Patients taking responsibility for their own care    IDE introduced as something active and pleasant while on dialysis    Feeling of taking an active role in their care | **Interest and willingness to take part**  *Where PA is considered appealing* | Willingness to engage  IDE introduced as something active and pleasant while on dialysis | “I definitely want to know about exercise. Doctors and nurses should give us some suggestions in terms of exercise, for example, how to exercise” (Song, 2019) |
|  |  | **Desire to take a more active role in own care**  *Where taking part in PA is seen as taking an active role in patient’s own care* | Already attempting exercise  Patients taking responsibility for their own care  Feeling of taking an active role in their care | “But my life is different now being on dialysis because I’m taking more control. It’s not like I have to go to work. I have to come to the hospital because it’s my health, but I have a choice, too. When I was in the workforce, it was just set. So, I wasn’t really planning.” (Rothpletz-Puglia, 2022) |
| Goals  *Motivation – reflective*  “Mental representations of outcomes or end states that an individual wants to achieve” | Desire to live  Desire to gain/maintain independence    Desire not to decline    Desire to improve strength    Desire to regain previous ability  Patients having intrinsic goals  Patients having extrinsic goals    Aiming to be healthy for transplant | **Determination to maintain current function**  *Where taking part in PA demonstrates patients’ desire not to give up. This can entail desired improvement, or desire to avoid decline* | Desire to live  Desire to gain/maintain independence    Desire not to decline    Desire to improve strength    Desire to regain previous ability | “If I sit here it’s just going to get harder and harder for me to do, so I have to try, I have to try” (Liu, 2020) |
|  |  | **Having specific goals to aim for gives patients a sense of purpose**  *Where patients have a goal which motivates them to take part in PA* | Patients having intrinsic goals  Patients having extrinsic goals  Aiming to be healthy for transplant | “If I don’t walk, I don’t get a kidney.” (Sheshadri, 2024) |
| Memory, Attention and Decision Processes  *Capability – psychological*  “The ability to retain information, focus selectively on aspects of the environment and choose between two or more alternatives” | *No facilitators coded* |  |  |  |
| Environmental Context and Resources  *Opportunity – physical*  “Any circumstance of a person’s situation or environment that discourages or encourages the development of skills and abilities, independence, social competence, and adaptive behaviour” | Group exercise ensures safety    Access to gyms    Ongoing access to rehab equipment    Exercising alone    User friendly IDE equipment for patients to use    When patients can retrieve their own equipment and monitor themselves/low reliance on staff    Supervision by healthcare professional    IDE bikes available    Exercise at dialysis centre/unit    PA as part of job    Integrating with daily tasks    Being able to regulate own workload    Additional, exercise specific staff    Active travel    Not taking up free time    Being able to exercise at home    Able to perform complementary activity as they wish in own time    Access to parks | **Equipment that’s easy to access and use**  *Where there are minimal barriers to accessing and using equipment for PA. Patients feel in control and can be less reliant on staff and staff are less burdened* | User friendly IDE equipment for patients to use  When patients can retrieve their own equipment and monitor themselves/low reliance on staff  IDE bikes available  Being able to regulate own workload | Most patients experienced the cycle as easy to handle without assistance and, thus, regarded it as a useful tool for exercising while undergoing haemodialysis. “I was surprised how easy it was to cycle right there in bed.” (Heiwe & Tollin, 2012) |
|  |  | **Access to a suitable environment for PA**  *Where patients have access to an environment for them to perform PA. This should be within their own control and to their own preferences* | Access to gyms  Exercise at dialysis centre/unit  Access to parks  Being able to exercise at home  Ongoing access to rehab equipment  Exercising alone  Able to perform complementary activity as they wish in own time | “I wouldn’t mind a home-based [exercise program] because you can keep to that no matter what …whereas the gym, you’re relying on a lot of different other things and I don’t feel safe…” (Sheshadri, 2024) |
|  |  | **An environment where support from others provides safety**  *Where patients have access to an environment which provides support with PA and offers safety in case of accident* | Group exercise ensures safety  Supervision by healthcare professional  Additional, exercise specific staff | “Patients also described group exercise as an essential way of guaranteeing safety in case an emergency arose related to their poor physical conditions” (Song, 2019) |
|  |  | **Integrating PA with daily routine**  *Where PA can be integrated with patient’s normal routine such as work or travel and doesn’t require additional planning* | Integrating with daily tasks  Active travel    Not taking up free time  PA as part of job | “I live on Fangwei Road, very close to the hospital. When I came for hemodialysis and when I left, I didn't take a car, I just walked, and I walked fast.” (Huang, 2023) |
| Social Influences  *Opportunity – social*  “Those interpersonal processes that can cause individuals to change their thoughts, feelings, or behaviours” | Social support from personal network    Support from care partner    Group exercise ensures safety    Recommendations of healthcare professionals    Importance of patient’s family    Patient encouragement from staff    The influence of staff personal values about exercise  Camaraderie and normalcy in the Unit    Fostering a positive common identity    Tailored PA (by healthcare professional) suitable for dialysis patients    Positive sense of competition between patients    Encouragement from patient peers    Inspiration from peers    Physician involvement  Exercise champions    Seeing inactive peers decline | **Personal social support system**  *Where social connections outside of dialysis such as family, friends and community also support PA in any capacity* | Social support from personal network  Support from care partner  Importance of patient’s family | “My mom and I used to go to the gym together but now we don’t because her work schedule changed. We used to ride bikes together in her subdivision but once again we don’t anymore because of the schedule change. They are always encouraging me to get up and be active. My mom always says that working out helps with your self-esteem. . . .” (Sieverdes, 2015) |
|  |  | **Support from dialysis staff and community**  *Where social connections within the dialysis centre such as peers and healthcare professionals support PA in any capacity* | Group exercise ensures safety  Recommendations of healthcare professionals  Patient encouragement from staff  The influence of staff personal values about exercise  Camaraderie and normalcy in the Unit  Fostering a positive common identity  Tailored PA (by healthcare professional) suitable for dialysis patients  Positive sense of competition between patients  Encouragement from patient peers    Inspiration from peers  Physician involvement  Exercise champions  Seeing inactive peers decline | “My doctor, the one that I deal with at clinic, I’d do anything he tells me to do” (Jhamb, 2016) |
| Emotion  *Motivation – automatic*  “A complex reaction pattern, involving experiential, behavioural and physiological elements by which the individual attempts to deal with a personally significant matter or event” | Enjoyment    Sense of pride and satisfaction from exercising  Avoiding boredom | **Perceived psychological benefits**  *Where patients perceive that PA makes them feel better. It could be a positive emotion or it could be the absence of a negative one such as boredom* | Enjoyment    Sense of pride and satisfaction from exercising  Avoiding boredom | Enjoyment of PA was an important determinant of maintaining PA. The patient spoke with pride when it came to running when it rained and going out for a walk with his or her neighbours when it snowed. (Huang, 2023) |
| Behavioural Regulation  *Capability – psychological*  “Anything aimed at managing or changing objectively observed or measured actions” | Self-monitoring (specifically with technology)    Forming a routine    Integrating with daily tasks    Feeling of taking an active role in their care    Choice within activity    Being able to regulate own workload    Choice of when to exercise during dialysis | **A perceived sense of control**  *Where patients are able to exert control over some aspects of PA. This could be having choice in activity, regulating the intensity, being able to monitor themselves or something else* | Self-monitoring (specifically with technology)    Forming a routine    Integrating with daily tasks    Feeling of taking an active role in their care    Choice within activity    Being able to regulate own workload    Choice of when to exercise during dialysis | “At first it was still difficult to play all these games, so I did it more slowly, if I could adjust it to suit me, it would make my training more effective.” (Hu, 2024) |

**Barriers**

| **TDF Domain** *(Corresponding to COM-B construct)* | **Barriers in domain** (Number of times each barrier present**)** | **Themes** *Description of theme* | **Barriers in each theme** | **Supportive data** *(First author, year)* |
| --- | --- | --- | --- | --- |
| Knowledge  *Capability – psychological*  “An awareness of the existence of something” | Lack of information sources for patients and staff    Misconceptions about the relationship between PA and condition(s)    Patients lack of knowledge of the benefits    Lack of enough information support for patients    Lack of guidance/structure for patients  Not knowing what to expect from exercise    Not knowing which patients are eligible    Lack of practical knowledge to support IDE | **Low prioritisation of PA due to lack of understanding of value**  *Where patients don’t perceive any benefit from PA or negative consequences of not engaging, therefore do not see it as necessary or important or of value* | Misconceptions about the relationship between PA and condition(s)  Patients lack of knowledge of the benefits | “No one said to me to be active. I don't know it (PA) and don't value that.” (Huang, 2023)  “There's nothing wrong with being inactive anyway, right?” (Huang, 2023) |
|  |  | **Lack of practical education and knowledge**  *Where patients lack practical instructions or guidance on how to perform PA which prevents them from confidently engaging* | Lack of information sources for patients and staff  Lack of enough information support for patients    Lack of guidance/structure for patients  Not knowing what to expect from exercise  Lack of practical knowledge to support IDE | “Doctors told me to do exercise moderately, and it is beneficial. But they didn’t say anything about specific exercise intensity, so I understand exercise as walking” (Song, 2019) |
|  |  | **Perceived inability to participate**  *Lack of knowledge as to whether the patient is safe and capable of taking part in PA* | Not knowing which patients are eligible | “It depends on their health state and depends on their age too. Age matters if you are very old, some people are very old there so I don’t believe those people can do the exercise” (Castillo, 2021) |
| Skills  *Capability – psychological and physical* | Lack of physical ability  Poor physical condition    Fatigue    Other health conditions    Pain (general)    Poor mental health | **Patients believe that they are too unwell for PA**  *Belief that their physical and/or mental condition is not healthy enough to take part in PA* | Lack of physical ability  Poor physical condition    Fatigue    Other health conditions    Pain (general)    Poor mental health | “I feel somewhat exhausted after a bit of exertion, you know. Of course I have a bit of a breathing problem, bronchitis, that goes with the lung disease, taking puffers and so on. I also had a double bypass. I had a lot of things happening, that may have something to do with why I don’t exercise. Having kidney disease, heart disease, lung disease and the rest of it and  being the age I am, I’m 76 years old now, that doesn’t really put you in the frame of mind to start any marathon.” (Kontos, 2007) |
| Social/Professional Role and Identity  *Motivation – reflective and automatic* | Cultural beliefs about PA    Exercise monitoring/assessment/provision not included in care provision/ not the nurses’ role    Identity as a ‘good’ patient | **Patient’s view of identity not aligned with PA**  *Where a patient’s view of their identity doesn’t align with performing PA due to cultural beliefs* | Cultural beliefs about PA | “Doing exercise in the gym is too vigorous. You know, strenuous exercise is not suitable for patients with HD, which is not harmful to Shen Qi” (Song, 2019) |
|  |  | **Nurses should be nursing**  *Where PA provision is not considered part of the role of dialysis nurse. This creates an unwillingness of patients to request support* | Exercise monitoring/assessment/provision not included in care provision/ not the nurses’ role    Identity as a ‘good’ patient | “My concern is the nurses need to be nursing not lifting bikes on and off beds” Young et al. (2015) |
| Beliefs about capabilities  *Motivation – reflective*  “Acceptance of the truth, reality, or validity about an ability, talent or facility that a person can put to constrictive use” | Patient belief that condition(s) preclude PA  Staff belief that patients are not capable of IDE    Belief that age limits capacity  Lack of confidence in ability to perform activity    Poor mental health  Additional source of pressure for patients  Detachment due to dialysis | **Patients’ perceived lack of confidence and competence**  *When patients don’t feel able to engage in PA confidently and competently. This could be due to perceived physical or mental limitations.* | Patient belief that condition(s) preclude PA  Belief that age limits capacity  Lack of confidence in ability to perform activity    Poor mental health  Additional source of pressure for patients  Detachment due to dialysis | “I found I’ve got osteoporosis this year. I assume people with long-term HD have the same condition with me, suggesting our bones are frail. So bone fracture may occur if exercise is performed” (Song, 2019) |
|  |  | **Discouraged from doing PA by staff**  *Where staff believe the patient isn’t capable of participating in PA and openly or discreetly express this belief it is discouraging* | Staff belief that patients are not capable of IDE | “When it was first mentioned one of the nurses came to me and said they won’t let you do that. I said yes they will, why wouldn’t they? They won’t. And he ... didn’t seem too keen” (Young, 2015) |
| Optimism  *Motivation – reflective and automatic*  “The confidence that things will happen for the best or that desired goals will be attained” | Patient belief that no improvement is possible | **A pessimistic attitude towards PA**  *Where patients do not believe that any improvement is possible, they do not see PA as capable of changing them* | Patient belief that no improvement is possible | “I feel this way myself, because I have been through it for a long time, um, it will definitely get worse as I go on.” (Huang, 2023) |
| Beliefs about consequences  *Motivation – reflective*  “Acceptance of the truth, reality, or validity about outcomes of a behaviour in a given situation” | Concern injury and accident    Concern exercise may lead to sickness    Lack of perceived benefit    Reduced access to patients in emergency if IDE equipment in the way    Belief exercise is not necessary for long-term health goals  Asking for help with IDE could result in less help for more important things  Fear of fistula damage  Fear of pain    Fear of falling    General fears about safety    Fear of further fatigue    Patients fear being a burden to staff    IDE could disrupt dialysis    Movement from exercise could set off dialysis alarm    Doubts about benefits | **Concerns and fear of adverse events**  *Belief that PA could make the situation worse. This could be through increasing chance of injury, or by worsening their dialysis experience, or something else* | Concern injury and accident    Concern exercise may lead to sickness  Reduced access to patients in emergency if IDE equipment in the way  Asking for help with IDE could result in less help for more important things  Fear of fistula damage  Fear of pain    Fear of falling    General fears about safety    Fear of further fatigue  Patients fear being a burden to staff    IDE could disrupt dialysis    Movement from exercise could set off dialysis alarm | “I am more concerned if I have low blood pressure, and pass out I would be stuck on a chair with a cumbersome bike at the end” (Young, 2015) |
|  |  | **Perceived lack of meaningful impact**  *Belief that PA will not result in any meaningful improvement in any aspect of patient lives, or doubt that it would make a difference* | Lack of perceived benefit  Belief exercise is not necessary for long-term health goals  Doubts about benefits | “And we all have osteoporosis at some level, and arthritis. And the pain and the discomfort...  ruins your will to continue. You say to yourself, ‘it’s always some other thing’’ and ‘‘what good is it doing to me?’’ and you can get very depressed about it. And I think that takes away the will to do any exercise.” (Kontos, 2007) |
| Reinforcement  *Motivation – automatic*  “Increasing the probability of a response by arranging a dependent relationship, or contingency, between the response and a given stimulus” | Pain during exercise    Fatigue after exercise    Lack of perceived benefit    Previous negative exercise test experience influences perception of IDE  Movement from IDE could set off dialysis alarm  Unpleasant physical reactions to exercise  Previous injury | **Previous experience of negative outcomes**  *Where PA is associated with negative feelings for patients, due to previous negative experiences which could be symptomatic, emotional or environmental.* | Pain during exercise    Fatigue after exercise  Previous negative exercise test experience influences perception of IDE  Unpleasant physical reactions to exercise  Previous injury  Movement from IDE can set off dialysis alarm | “I have chest pain when I walk sometimes, and I have to stop. I don’t want to do more than my body can do – I know my body.” (Sheshadri, 2020)  The main barrier to IE was concern about triggering the hemodialysis machine alarm.  Most patients had experienced triggering the machine alarm if they moved their cannulated  arm at all. The concern with the machine alarm seemed to be the alarm itself and not  potential reasons for the alarm, such as changes in blood pressure or dialysis flow. (Wodskou, 2021) |
|  |  | **Lack of perceived benefit**  *Where patient has not perceived any benefit from PA and therefore is not motivated to take part* | Lack of perceived benefit | “You say to yourself, ‘it’s always some other thing’’ and ‘‘what good is it doing to me?’’ (Song, 2019) |
| Intentions  *Motivation – reflective*  “A conscious decision to perform a behaviour or a resolve to act in a certain way” | Lack of motivation    Preference is not PA    Lack of interest | **Lack of motivation and desire**  *Where patients are uninterested in taking part in PA, lacking sufficient motivation to do so* | Lack of motivation    Preference is not PA    Lack of interest | “I rarely do exercise. I have never thought about it at all. I am lazy . . . I have no  motivation to exercise . . . no reason, no motivation, and I am used to it” (Song, 2019) |
| Goals  *Motivation – reflective*  “Mental representations of outcomes or end states that an individual wants to achieve” | Patients believe PA is optional  Not necessary for long-term health goals  Goals unattainable so no point trying | **Misunderstanding relationship between PA and goals**  *Patients believe that PA isn’t necessary to reach goals, or that even if they take part in PA they won’t be able to reach goals anyway* | Patients believe PA is optional  Not necessary for long-term health goals  Goals unattainable so no point trying | “I saw that goal, and I didn’t think I could reach it, so I didn’t think it was worth trying” (Sheshadri, 2020) |
| Memory, Attention and Decision Processes  *Capability – psychological*  “The ability to retain information, focus selectively on aspects of the environment and choose between two or more alternatives” | *No barriers coded* |  |  |  |
| Environmental Context and Resources  *Opportunity – physical*  “Any circumstance of a person’s situation or environment that discourages or encourages the development of skills and abilities, independence, social competence, and adaptive behaviour” | Existing resources do not include PA/exercise guidance    Bad weather    Cost of exercise facilities/equipment    Some facilities pose high risk of injury    Facilities too crowded    Exercise not a priority for nurses    Patient lack of time due to dialysis    Nurses lack of time due to other tasks    IDE equipment not suitable for everyone  Exercising in the dialysis centre    IDE as an additional source of pressure    IDE could disrupt the routine on the dialysis unit    Local environment risks    IDE bikes not stable  Fistula/catheter prevents activity    Physical limitation during dialysis prevents IDE    Lack of suitable exercise options (general)  Lack of privacy in IDE    No appropriate route to request assistance with IDE    Dialysis environment restricts conversation/sense of community    Instructor lack of knowledge of condition    Lack of in-centre options  Hard to use equipment  Lack of time reducing effective delivery of IDE  Staff rely on patients to self-refer  Exercise options in local gyms are unsuitable for dialysis patients | **Perceived environmental barriers, hazards and risks**  *Where there are issues linked to the environment they would like to engage in PA in such as parks, gyms or even the IDE equipment in the dialysis centre. These additional barriers must be overcome if they are to take part this creates an additional barrier to PA* | Bad weather  Some facilities pose high risk of injury  Facilities too crowded  Local environment risks    IDE bikes not stable  Instructor lack of knowledge of condition  Cost of exercise facilities/equipment | “I don’t do too much outside walking when there is ice on the ground” (Liu, 2020) |
|  |  | **Lack of priority for PA in dialysis environment**  *Where dialysis centres are not designed to offer PA and there is no culture as such or consideration for offering support with it* | Existing resources do not include PA/exercise guidance  Exercise not a priority for nurses    Patient lack of time due to dialysis    Nurses lack of time due to other tasks  Exercising in the dialysis centre  IDE as an additional source of pressure    IDE could disrupt the routine on the dialysis unit  Lack of privacy in IDE  No appropriate route for patients to request assistance with IDE    Environment restricts conversation/sense of community  Lack of in-centre options  Lack of time reducing effective  delivery of IDE  Staff rely on patients to self-refer | Patients described aspects of the unit’s social structure that were barriers to receiving assistance with IDE. The existing processes for obtaining help from staff (ringing the bell) were viewed as inappropriate for IDE (Q33). One patient expressed concern that using the bell for help with exercise could have negative consequences when help was urgently needed. (Thompson, 2016) |
|  |  | **Lack of dialysis specific guidelines and adaptations restrict PA**  *Where the specific needs of dialysis patients in regard to their skills or physical limitations is not addressed and this creates a barrier to PA* | Hard to use equipment  Lack of suitable exercise options (general)  Fistula/catheter prevents activity  Physical limitation during dialysis prevents IDE  IDE equipment not suitable for everyone  Exercise options in local gyms are unsuitable for dialysis patients | “Based on the fact that I now have a catheter and the fistula, it’s prohibited of me from going back to that [water pool exercise class]” (Jhamb, 2016) |
| Social Influences  *Opportunity – social*    “Those interpersonal processes that can cause individuals to change their thoughts, feelings, or behaviours” | Friends/acquaintances not as understanding as family/spouse  Lack of support from social network   Lack of support from health professionals  Feeling excluded from social exercise opportunities with those who do not require dialysis  Peers’ information/advice untrustworthy and confusing  Lack of guidance from healthcare professionals  Family/friends lack of knowledge of condition(s)  plan  Family concern of ability  Direct guidance not to exercise from family  Friends/Family lack of PA  Inconsistent help from dialysis staff for IDE  Staff appear too busy to help with IDE  Lack of ‘buy in to IDE’ from staff  Dependence on others to exercise  Negative comments about IDE from patients | **Perceived lack of support from staff**  *Where dialysis staff are not perceived to be supportive of PA, or don’t provide necessary support* | Lack of support from health professionals  Lack of guidance from healthcare professionals  Inconsistent help from dialysis staff for IDE  Staff appear too busy to help with IDE  Lack of ‘buy in’ to IDE from staff | “I know the nurses don’t like doing it. They don’t ask you and they don’t remind you, ‘Are you going to do your exercises?’ Some do, some don’t.” (Thompson, 2016) |
|  |  | **Perceived lack of support from personal social network**  *Where patients’ social network do not support PA. This could include friends, family, partners or others they engage with, and could be lack of support or clear discouragement* | Friends/acquaintances not as understanding as family/spouse  Lack of support from social network  Feeling excluded from social exercise opportunities with those who do not require dialysis  Peers’ information/advice untrustworthy and confusing  Family/friends lack of knowledge of condition(s)  plan  Family concern of ability  Direct guidance not to exercise from family  Friends/Family lack of PA  Dependence on others to exercise  Negative comments about IDE from patients | “My family thinks exercise makes me feel tired, so all they tell me is to have more rest” (Song, 2019) |
| Emotion  *Motivation – automatic*    “A complex reaction pattern, involving experiential, behavioural and physiological elements by which the individual attempts to deal with a personally significant matter or event” | Pain (general)  Apprehension towards exercise  Lack of enjoyment/monotonous nature of PA/disliking exercising/finding it boring  Frustration of failure | **Negative symptoms associated with PA**  *Where patient experiences negative symptoms in relation to PA* | Pain (general) | Some patients had problems with lower or upper back pain and were concerned that cycling in bed or the haemodialysis chair would increase their pain.” (Heiwe & Tollin, 2012) |
|  |  | **Negative emotion and feeling overwhelmed**  *Where patients find PA or the thought of it mentally overwhelming or unpleasant* | Apprehension towards exercise  Frustration of failure  Lack of enjoyment/monotonous nature of PA/disliking exercising/finding it boring | “. The participants also worried that they would experience cycling as boring in the long term, and this would cause them to drop out” (Heiwe & Tollin, 2012)  “And we all have osteoporosis at some level, and arthritis. And the pain and the discomfort...  ruins your will to continue. You say to yourself, ‘it’s always some other thing’’ and ‘‘what good is it doing to me?’’ and you can get very depressed about it. And I think that takes away the will to do any exercise.  (Kontos, 2007) |
| Behavioural Regulation  *Capability – psychological*  “Anything aimed at managing or changing objectively observed or measured actions” | Used to being sedentary | **Accustomed to sedentary lifestyle**  *Where patients are used to being sedentary, and have no habit of taking part in PA* | Used to being sedentary | “I rarely do exercise. I have never thought about it at all. I am lazy . . . I have no  motivation to exercise . . . no reason, no motivation, and I am used to it” (Song, 2019) |

# Barrier and Facilitator Thematic Analysis – Merged Staff and Physician Data

**Facilitators**

| **TDF Domain** *(Corresponding to COM-B construct)* | **Facilitators in domain**  underlined codes denote staff and physician physician only codes are labelled (physician) | **Themes** *Description of theme*  underlined themes denote staff and physician physician only themes are labelled (physician) | **Facilitators in each theme** | **Supportive data** *Participant group, (First author, year)* |
| --- | --- | --- | --- | --- |
| Knowledge  *Capability – psychological*  “An awareness of the existence of something” | Knowledge about the benefits of physical activity  Knowledge about the consequences of lack of physical activity  Education programme/package for patients  Staff recognition of the benefits (general) of IDE  Staff having practical knowledge of how to facilitate and support IDE  Patient education on benefits of exercise is key  Offering a pilot trial of IDE  Staff knowing which patients are safe to take part in IDE and where IDE is contraindicated  Shared understanding of how IDE is prioritised on dialysis unit  Staff knowledge to reassure and encourage patients  Knowledge that a wide range of patients can/do take part in IDE | **Staff having practical knowledge to facilitate and support PA**   *Where sufficient information and guidance around what is expected, what is safe, and how physical activity will be supported is provided to staff and patients. Information which supports patients to take part in physical activity on a practical level* | Staff having practical knowledge of how to facilitate and support IDE  Offering a pilot trial of IDE  Staff knowing which patients are safe to take part in IDE and where IDE is contraindicated  Shared understanding of how IDE is prioritised on dialysis unit  Staff knowledge to reassure and encourage patients  Knowledge that a wide range of patients can/do take part in IDE | “[Training] gives the staff chance to learn about [IDE] and understand the information and be able to learn it. You can’t expect them to do it straight away.” Staff (Young, 2015) |
|  |  | **Patients and staff understanding why physical activity is needed**   *Where the benefits of participating and consequences of not participating in physical activity are understood by patients and staff. This drives patient motivation to take part and staff motivation to support* | Knowledge about the benefits of physical activity  Knowledge about the consequences of lack of physical activity  Education programme/package for patients  Staff recognition of the benefits (general) of IDE  Patient education on benefits of exercise is key | “The patient couldn’t make it 200 metres uphill in his walk home from dialysis. He travelled everywhere in his car. At the very end of our exercises, he was able to walk that way comfortably. He could walk to his home on the second floor.” Staff (Zelko, 2023)  “One of the biggest morbidities of dialysis is cardio-vascular so improving cardiovascular health……when patients have very low blood pressure [during HD treatment] sometimes it [exercise during HD] helps keep the pressure up” Physician (Jhamb, 2018) |
| Skills  *Capability – psychological and physical*  “An ability or proficiency acquired through practice” | Exercise as part of routine care | **Including physical activity as part of routine care**  *Where physical activity is included as part of routine care, it can be incorporated more easily into the existing care provision* | Exercise as part of routine care | “Exercise can be introduced. They already comply with the diet and fluid restrictions because they have to. So exercise should also be something they have to do. This has to be part of their education and once they get involved in their own treatment, in the same way that they are already with diet and fluid, I believe they will get the motivation and they will continue. And if it were part of their treatment, it would be part of what we do too.” Staff (Kontos, 2007) |
| Social/Professional Role and Identity  *Motivation – reflective and automatic* | Exercise as part of routine care  Fostering a positive common identity as patients who exercise  Staff view exercise as team effort | **Staff accepting that supporting physical activity is part of care**  *Where dialysis staff see supporting physical activity as part of their role rather than an additional request. Where it is seen as something the whole team contributes to* | Exercise as part of routine care  Staff view exercise as team effort | However, each [nurse] stated that they would support the PCTs in promoting and encouraging activity participation and thought it would be a good thing to consider as a team effort.  Staff (Painter, 2014) |
| Beliefs about capabilities  *Motivation – reflective*  “Acceptance of the truth, reality, or validity about an ability, talent or facility that a person can put to constrictive use” | Staff feel able to motivate patients | **Staff have skills to motivate patients to engage in PA** *Where staff feel that their skills and relationship with patients is well-suited to motivating and supporting engagement in physical activity* | Staff feel able to motivate patients | ”They do what we ask them to do . . . to a large extent” Staff (Wodskou, 2021) |
| Optimism  *Motivation – reflective and automatic*  “The confidence that things will happen for the best or that desired goals will be attained” | *No facilitators coded* |  |  |  |
| Beliefs about consequences  *Motivation – reflective*  “Acceptance of the truth, reality, or validity about outcomes of a behaviour in a given situation” | Perceived benefit to physical well-being  Perceived benefit to mental well-being  Positive beliefs about physical activity (long-term benefits)  Staff recognition of the benefits (general) of IDE  Maintain independence  Belief that IDE could enhance dialysis treatment  Healthier patients are easier for staff to manage  IDE could improve experience of dialysis | **Physical activity could improve patient quality of life**  *Where staff are aware of the benefits taking part in physical activity has for patients and how it can impact different aspects of their life such as functionality and dialysing* | Perceived benefit to physical well-being  Perceived benefit to mental well-being  Positive beliefs about physical activity (long-term benefits)  Staff recognition of the benefits (general) of IDE  Maintain independence  IDE could improve experience of dialysis | “I mean you may not have working kidneys but you should be able to be active. Otherwise you’re just kind of stagnant and don’t do anything. I think some patients just kind of you know, ‘I’m on dialysis, I don’t ever really feel good.’ But I think if they got up and moving and actually had a little bit more physical activity they might feel a lot better than if they just kind of sit there and let the sickness take over their life.”  Staff (Painter, 2014)  “I think it (exercise) maintained a certain conditioning, a certain quasi-muscle status of the patient. Because physical activity affects not only muscular, but also bone structures.” Physician (Zelko, 2023) |
|  |  | **Patients engaging in PA could dialyse better and have higher functionality**  *Where staff perceive that patients taking part in physical activity could make work on the dialysis unit easier and have less risk of complications, accidents and injury* | Belief that IDE could enhance dialysis treatment  Functionally able patients are easier for staff to manage | “Well, I think that exercise improved their blood flow, (in) the circulatory system and the blood circulation in brain and also filling of veins and thus for us also better blood flow in vascular access.” Staff (Zelko, 2023)  “Taking care of a dialysis patient when they’re more able to do things on their own means they’re a much easier patient to take care of. Here and at home. They feel better, they’re happier, they’re not needy. You know, [imitates whining tone] ‘wipe my chin’.” Staff (Painter, 2024) |
| Reinforcement  *Motivation – automatic*  “Increasing the probability of a response by arranging a dependent relationship, or contingency, between the response and a given stimulus” | Observing/feeling the benefits  Improvement in energy levels  Previous good experience of rehab support  Staff observing patient enjoyment | **PA is associated with positive outcomes for patients**  *Where staff see that patients enjoy physical activity and gain benefits from it* | Observing/feeling the benefits  Improvement in energy levels  Previous good experience of rehab support  Staff observing patient enjoyment | “Last year [before the cycling] the dialysis  patients tended to be unmotivated, depressed.  I’ve seen them cycling and they are more  cheerful, happy, its helping them” Staff(Young, 2015) |
| Intentions  *Motivation – reflective*  “A conscious decision to perform a behaviour or a resolve to act in a certain way” | Exercise as part of routine care  Patients taking responsibility for their own care | **Staff and patients committing to physical activity**  *Where taking part in physical activity is seen as a shared intention between staff and patients, both are motivated more* | Exercise as part of routine care  Patients taking responsibility for their own care | “Exercise can be introduced. They already comply with the diet and fluid restrictions because they have to. So exercise should also be something they have to do. This has to be part of their education and once they get involved in their own treatment, in the same way that they are already with diet and fluid, I believe they will get the motivation and they will continue. And if it were part of their treatment, it would be part of what we do too.” Staff (Kontos, 2007) |
| Goals  *Motivation – reflective*  “Mental representations of outcomes or end states that an individual wants to achieve” | Patients having intrinsic goals  Patients having extrinsic goals | **Patients having goals which align with PA**  *Where patients have a goal which motivates them to take part in physical activity* | Patients having intrinsic goals  Patients having extrinsic goals | “Some of our patients are motivated by health goals, it doesn’t just have to be transplant, if they wrestle with something else health wise and they’re on a path for improvement their own health for some can be motivating as well.” Staff (Jhamb, 2016)  “And so I think if they could get in an exercise program and hopefully get stronger in order to better prepare them for transplant I would be excited for that patient” Physician (Castillo, 2021) |
| Memory, Attention and Decision Processes  *Capability – psychological*  “The ability to retain information, focus selectively on aspects of the environment and choose between two or more alternatives” | *No facilitators coded* |  |  |  |
| Environmental Context and Resources  *Opportunity – physical*  “Any circumstance of a person’s situation or environment that discourages or encourages the development of skills and abilities, independence, social competence, and adaptive behaviour” | Ongoing access to rehab equipment  User friendly IDE equipment for patients to use  IDE equipment that is easy to move and maintain  When patients can retrieve their own equipment and monitor themselves/low reliance on staff  Exercise at dialysis centre/unit  Staff having time to support IDE  Additional, exercise specific staff  Not taking up free time  Low cost material resources  IDE provided by non-nurse staff  Exercise that can be done in bed  Safety benefits of IDE (physician) | **Minimising disruption to current dialysis processes**  *Where taking part in PA is easy for patients and relies minimally on existing dialysis staff. This could also include where it uses additional staff and therefore doesn’t take from existing staff, and choosing PA which patients can manage themselves* | User friendly IDE equipment for patients to use  IDE equipment that is easy to move and maintain  When patients can retrieve their own equipment and monitor themselves/low reliance on staff  Additional, exercise specific staff  IDE provided by non-nurse staff  Exercise that can be done in bed | “So that’s the hard part, I find, like, with patients who don’t know as well as others know, what they have to do. I think we have to do some minor adjustments on the bikes; seems to be a little bit more tension, just a little bit less tension, that’s something it’s quickly, we can do that and walk away; they’ll carry on with whatever they are doing. But some patients, like I said, who are not—I can’t say with it, but not as comfortable may be doing the exercises as others, it’s a little harder to—for us to monitor whatever they do is proper. I don’t know, it’s maybe they need a bit more education or its maybe they are not good people for the study.” Staff (Thompson, 2016) |
|  |  | **Benefits of IDE**  *Where taking part in PA in IDE offers benefits such as convenience, safety and good use of time for patients, in comparison to non-medical settings.* | Exercise at dialysis centre/unit  Not taking up free time  Safety benefits of IDE (physician) | “The convenience too, if they were here for 3–4 h, you know” Staff (Jhamb, 2016)  “There is such a time thing for dialysis itself, it would be nice to somehow turn that time into productivity. I think accessibility would be good… you might have more compliance because you’re stuck there for four hours” Physician (Jhamb, 2016) |
|  |  | **Financial viability**  *Where implementing physical exercise is considered to be financially viable* | Low cost material resources | The majority of the participants believed that material resources for IDE were negligible and could be covered directly by internal financial recourses of dialysis centres.  Staff (Zelko, 2023)  “To buy the necessary equipment, a few sets of bands and balls, that isn’t a problem. These cost a few (hundreds) of euros’ it’s minor, a one-time expense.” Physician (Zelko, 2023) |
| Social Influences  *Opportunity – social*  “Those interpersonal processes that can cause individuals to change their thoughts, feelings, or behaviours” | Recommendations of healthcare professionals  Importance of patient’s family  Patient encouragement from staff  Camaraderie and normalcy in the Unit  Fostering a positive new common identity with other dialysis patients  Tailored physical activity (by healthcare professional) suitable for dialysis patients  Positive sense of competition between patients  Encouragement from peers  Inspiration from peers  Physician involvement  Exercise champions  Staff observing patient enjoyment  Collaboration between staff team to support IDE  Active involvement and leadership of senior staff | **Patients having support from family, dialysis staff and community**  *Where social connections both within and outside the dialysis centre such as family, peers and healthcare professionals support physical activity in any capacity* | Recommendations of healthcare professionals  Patient encouragement from staff  Camaraderie and normalcy in the Unit  Fostering a positive common identity  Tailored physical activity (by healthcare professional) suitable for dialysis patients  Positive sense of competition between patients  Encouragement from peers  Inspiration from peers  Physician involvement  Exercise champions  Patient enjoyment  Collaboration between staff team to support IDE  Active involvement and leadership of senior staff  Importance of patient’s family | “They would see how much fun other people were having doing it, and if the staff gets into it, just walking around and doing their work, they’ll be playing around with it, and it just makes for a very light and fun afternoon, time will go fast.” Staff (Jhamb, 2016)  “It’s that mentality if you see someone else trying to improve themselves and then you kind of like feel bad about yourself …….. And then maybe if they are seeing their friends across the aisle doing it, then that might motivate them to do it as well.” Staff (Jhamb, 2016)  “So, I think that the positions of nephrologists are very important, because they monitor the patient’s health. So actually, if they see that it really makes sense, and if they agree, then I don't think there should be a problem.” Staff (Zelko, 2023)  “Once I noticed that exercising patients started to talk with other patients about exercise in the locker room, about how they feel good (after exercise), about how they like doing something with their muscles. Maybe this support could encourage them to exercise.” Physician (Zelko, 2023) |
| Emotion  *Motivation – automatic*  “A complex reaction pattern, involving experiential, behavioural and physiological elements by which the individual attempts to deal with a personally significant matter or event” | Distraction from treatment | **Physical activity makes patients feel better**  *Where patients perceive that physical activity makes them feel better. It could be a positive emotion or it could be the absence of a negative one such as boredom* | Distraction from treatment | The anecdotal patients’ feedback was overwhelming, not just one or two, essentially all patients seemed to very much appreciate the attention, the diversion…. the patient response one after another after another totally was “This is fun, I like it, I love the person, I look forward to it” Staff (Jhamb, 2016) |
| Behavioural Regulation  *Capability – psychological*  “Anything aimed at managing or changing objectively observed or measured actions” | Change in medical culture | **Changing focus of care**  Where medical culture is changed to afford priority to physical activity for dialysis patients, as part of routine care that should be offered | Change in medical culture | “Exercise can be introduced. They already comply with the diet and fluid restrictions because they have to. So exercise should also be something they have to do. This has to be part of their education and once they get involved in their own treatment, in the same way that they are already with diet and fluid, I believe they will get the motivation and they will continue. And if it were part of their treatment, it would be part of what we do too.” Staff (Kontos, 2007) |

**Barriers**

| **TDF Domain** *(Corresponding to COM-B construct)* | **Barriers in domain**  underlined codes denote staff and physician physician only codes are labelled (physician) | **Themes** *Description of theme*  underlined themes denote staff and physician physician only themes are labelled (physician) | **Barriers in each theme** | **Supportive data** *(First author, year)* |
| --- | --- | --- | --- | --- |
| Knowledge  *Capability – psychological*  “An awareness of the existence of something” | Lack of information sources for patients and staff  Misconceptions about the relationship between physical activity and condition(s)  Staff lack of knowledge of the benefits of physical activity  Staff not knowing what is right to instruct  Lack of enough information support for patients  Staff lacking of knowledge of guidelines  Lack of guidance/structure for patients  Lack of information for caregivers and families  Lack of practical knowledge to support IDE  Not knowing which patients are safe to exercise (physician) | **Low prioritisation of PA by staff due to lack of understanding of value**  *Where staff don’t perceive there could be any meaningful benefit to patients from physical activity, nor do they perceive that there could be negative consequences of not engaging, therefore do not see physical activity as necessary or important* | Lack of information sources for patients and staff  Misconceptions about the relationship between physical activity and condition(s)  Staff lack of knowledge of the benefits of physical activity  Staff lacking of knowledge of guidelines  Lack of information for caregivers and families | “Doctors tell them, ‘you are restricted on this, you are restricted on that’ and they think, ‘well I just cannot do anything.’” Staff (Painter, 2014) |
|  |  | **Staff lack of practical education and knowledge of PA**  *Where staff lack practical instructions or guidance on how to facilitate physical activity for patients which prevents them from confidently engaging* | Staff not knowing what is right to instruct  Not knowing which patients are safe to exercise (physician)  Lack of practical knowledge to support IDE | Patient care staff expressed varying levels of confidence in making recommendations about physical activity. One PCT expressed willingness, stating “I’m sure the technicians could ask about activity, but it’s almost like you can’t suggest something to the patient because you’re not qualified to do that.” The same hesitancy was echoed by every other category of staff. Staff (Painter, 2014)  “Yeah I think your biggest challenge, on the medical side, your challenge is making sure that we don’t, that doctors or nephrologists don’t make patients ineligible when in fact they might stand to benefit. I think that’s the biggest risk from a medical standpoint” Physician (Castillo, 2021) |
|  |  | **Staff perceive lack of education and knowledge for patients**  *Where staff feel patients lack practical instructions or guidance on how to perform PA* | Lack of enough information support for patients  Lack of guidance/structure for patients | “I think you need to have at the outset, when you’re first told that you have kidney disease, I think there needs to be a whole education program including nutrition, exercise, what’s going to happen to you, and all the options that are open to you. Exercise is never even discussed but if it were discussed that would certainly motivate me.” Staff (Kontos, 2007)  “And it’s not just randomly pedalling without a clear goal or expectation. I think that’s the part where nurses, technicians and doctors even nephrologists don’t really have a lot of expertise in. And so, you know, if you just bring pedals to a unit and you say here you go that’s where you’re likely gonna fail because we lack that ability to really assess and tailor the programs to patients individually” Physician (Castillo, 2021) |
| Skills  *Capability – psychological and physical* | Poor physical condition  Fatigue  Other health conditions  Poor mental health  Exercise could be risky for unstable patients (physician) | **Staff believe many patients are too unwell for PA**  *Belief that patients’ physical and/or mental condition is not healthy enough to take part in physical activity* | Poor physical condition  Fatigue  Other health conditions  Poor mental health | The nurses described patients in hemodialysis as a heterogenous group in terms of age, level of function, and morbidity. However, most patients were elderly with symptoms of chronic illness, such as chronic fatigue and poor general condition Staff(Wodskou, 2021)  “The nature of a chronic illness I think has a set of mental challenges and obstacles all by itself simply because it’s a chronic disease” Physician (Jhamb, 2016) |
|  |  | **Acutely unwell patients are perceived to be too unstable for PA (physician)** | Exercise could be risky for unstable patients | “We have patients who we refer to as ‘acute’. These are problematic. These unstable patients just started their dialysis programme and among these patients exercise is problematic and risky.” Physician (Zelko, 2023) |
| Social/Professional Role and Identity  *Motivation – reflective and automatic* | Exercise monitoring/assessment/provision not included in care provision/ not the nurses’ role  Identity as a sick person  Staff don’t see patients as interested in exercise | **Nurses should be nursing**  *Where physical activity provision is not considered part of the role of dialysis nurse. This creates an unwillingness to support physical activity within care* | Exercise monitoring/assessment/provision not included in care provision/ not the nurses’ role | “It would have to be very minimal. As our staffing doesn’t provide for helping them exercise. I mean just setting them up for it is you know is extra work. we don’t always have the staff and we don’t always have the time” Staff (Jhamb, 2016)  “All you need is one of those patients to have a line that doesn’t work, a fistula that’s acting up or somebody that’s unwell and your whole day goes to hell in a handbag. So having to drag somebody’s physio equipment on top of it, you know, it’s just kind of one more thing to a never-ending list. But in that particular instance it’s not gonna get done because the priority is the sick patient, a line that doesn’t work” Staff (Castillo, 2021) |
|  |  | **Staff believe patients won’t engage in PA**  *Where the staff see the patient identity as ‘sick’ incompatible with interest in physical activity* | Identity as a sick person  Staff don’t see patients as interested in exercise | “At the moment I would say [exercise is] very low on [patients] agenda.” Staff (Young, 2015) |
| Beliefs about capabilities  *Motivation – reflective*  “Acceptance of the truth, reality, or validity about an ability, talent or facility that a person can put to constrictive use” | Patient belief that condition(s) preclude physical activity  Staff doubt in their capacity to assist with IDE  Poor mental health  New patients overwhelmed by physical therapy requirement  Detatchment due to dialysis (physician) | **Staff see PA as beyond patient ability**  *Where physical activity is seen as beyond patient ability. This includes both physical and mental* | Patient belief that condition(s) preclude physical activity  Poor mental health  New patients overwhelmed by physical therapy requirement  Detatchment due to dialysis | “The nature of a chronic illness I think has a set of mental challenges and obstacles all by itself simply because it’s a chronic disease” Staff (Jhamb, 2016)  “Physical activity and psychological conditions are connected. And so, I would say they surrender and are very depressed.” Physician (Zelko, 2023) |
|  |  | **Staff do not perceive themselves as capable of facilitating and supporting PA**  *Where staff don’t believe they are capable of facilitating and supporting physical activity for the patients* | Staff doubt in their capacity to assist with IDE | “With the bicycle[stationary bike]…that did add to our work load and not that it was a bad thing but you know it was a good thing for the patients but….I really don’t have a minute to spare” Staff (Jhamb, 2016) |
| Optimism  *Motivation – reflective and automatic*  “The confidence that things will happen for the best or that desired goals will be attained” | Patient belief that no improvement is possible | **Staff perceive patients have a pessimistic attitude towards PA**   *Where patients do not believe that any improvement is possible, they do not see physical activity as capable of changing them* | Patient belief that no improvement is possible | “When they first come in to get dialysis nobody ever feels good, they don’t really have any energy. That just kind of gets their mind set: this is what my life’s going to be. I’m never going to have energy, I’m never going to feel good. I might as well give up. And because I don’t feel good, I don’t want to do it.” Staff (Painter, 2014) |
| Beliefs about consequences  *Motivation – reflective*  “Acceptance of the truth, reality, or validity about outcomes of a behaviour in a given situation” | Concern injury and accident  Fear of losing transport benefit makes exercise lower priority  Reduced access to patients in emergency if IDE equipment in the way  Nurses fear injury to self from IDE equipment  Fear of fistula damage  Fear of pain  Fear of falling  General fears about safety  IDE could disrupt dialysis  Doubts about benefits  Belief that IDE would be unsuccessful | **Concerns and fear of adverse events**  *Belief that physical activity could make the situation worse. This could be through dangers to patients and staff, or inconvenience such as disruption or loss of transport* | Concern injury and accident  Fear of losing transport benefit makes exercise lower priority  Reduced access to patients in emergency if IDE equipment in the way  Nurses fear injury to self from IDE equipment  Fear of fistula damage  Fear of pain  Fear of falling  General fears about safety  IDE could disrupt dialysis | One-third of participants expressed concerns that IDE could contribute to musculoskeletal injuries in haemodialysis patients. They believed that exercise during dialysis could contribute to tendon and/or muscle injuries. Furthermore, participants expressed the opinion that a combination of training-related injury with dialysis- and exercise-related fatigue could increase risks of falls and injuries in patients.  Staff (Zelko, 2023)  They’re almost like sort of tied up to the machine. one hand is immobile so that pretty much you know they just have the use of one hand and we don’t like the patients to be really moving their arm too much because then the needles would move and there is chances of them infiltrating Physician (Jhamb, 2016) |
|  |  | **Perceived lack of meaningful impact for patients**  *Belief that physical activity will not result in any meaningful improvement in any aspect of patient lives, or doubt that it would make a difference* | Doubts about benefits  Belief that IDE would be unsuccessful | “I have only ever heard negatives [about IDE]. I was working in [another region] and they did it there and I heard a lot of moans...and then you see all the bikes at the side not being used” Staff(Young, 2015)  “So intradialytic exercise has been shown to improve certain patient symptoms like restless legs, for example. That’s the one that I believe may be true. There’s a bunch of other stuff that intradialytic exercise has been named to improve, like in terms of outcomes like depression, blood pressure, ultrafiltration, but I’m not sure I believe any of those other stuff, so maybe restless legs” Physician (Castillo, 2021) |
| Reinforcement  *Motivation – automatic*  “Increasing the probability of a response by arranging a dependent relationship, or contingency, between the response and a given stimulus” | Previous negative exercise experience influences perception of IDE | **Lack of perceived benefit for patients**  *Where a negative experience of IDE or physical activity reinforces the idea that it would not be successful* | Previous negative exercise experience influences perception of IDE | “Some of them used it [stationary bike] for a short time and then they lost interest in it”  Staff(Jhamb, 2016) |
| Intentions  *Motivation – reflective*  “A conscious decision to perform a behaviour or a resolve to act in a certain way” | Lack of motivation  Preference to rest during dialysis (rather than IDE)  Staff rely on patients to self-refer to take part in exercise | **Staff do not believe patients are motivated enough to engage in PA**  *Where staff believe patients are uninterested in taking part in physical activity and lack sufficient motivation to do so* | Lack of motivation  Preference to rest during dialysis (rather than IDE)  Staff rely on patients to self-refer to take part in exercise | “Oh I think the exercise thing is awesome but I think it will be a bit of a challenge to get some patients to agree. There’ll be patients there’ll be a handful of patients that will be quite willing, but there’ll be some patients that we’ll need to persuade a little bit … But it’ll be challenging for sure looking at our group” Staff (Castillo, 2021)  “Its not really part of the culture of during Dialysis….. some of them have been on Dialysis for a long time and they’re used to their routine and they come in and they do whatever they do and ….. I’m not sure that would be completely easy to change…its going to be an uphill battle to get them to exercise right now … they have their routine” Physician (Jhamb, 2016) |
| Goals  *Motivation – reflective*  “Mental representations of outcomes or end states that an individual wants to achieve” | Patients believe physical activity is optional | **Staff perceive that patients believe PA is optional**  *Where staff believe patients don’t place importance on physical activity or see it as necessary for them* | Patients believe physical activity is optional | All participants believed that patients had a minimal history of regular physical activity and remained inactive during their lives with nephrology disease. Their sedentary lifestyle and beliefs that physical activity is not important for their lives were identified as major barriers on the patients’ side for initiating IDE Staff group including physician (Zelko, 2023) |
| Memory, Attention and Decision Processes  *Capability – psychological*  “The ability to retain information, focus selectively on aspects of the environment and choose between two or more alternatives” | *No facilitators coded* |  |  |  |
| Environmental Context and Resources  *Opportunity – physical*  “Any circumstance of a person’s situation or environment that discourages or encourages the development of skills and abilities, independence, social competence, and adaptive behaviour” | Existing resources do not include physical activity/exercise guidance  Cost of exercise facilities/equipment  Facilities too crowded  Exercise not a priority for nurses  Fear of losing transport benefit makes exercise lower priority  Patient lack of time due to dialysis  Nurses lack of time due to other tasks  IDE equipment not suitable for everyone  Reduced access to patients in emergency if IDE equipment in the way  IDE as an additional source of pressure  IDE could disrupt the routine on the dialysis unit  Local environment risks  Lack of resources  Lack of privacy in IDE  Expectation for patient to access benefits and not work  Cost of dedicated staff  Where IDE has specific logistical needs  Lack of time reducing effective delivery of IDE  Manual handling challenges for staff | **Lack of priority for physical activity in dialysis setting**  *Where dialysis centres are not designed to offer physical activity and there is no culture as such or consideration for offering support with it* | Existing resources do not include physical activity/exercise guidance  Exercise not a priority for nurses  Fear of losing transport benefit makes exercise lower priority  Patient lack of time due to dialysis  Nurses lack of time due to other tasks  IDE equipment not suitable for everyone  IDE as an additional source of pressure  IDE could disrupt the routine on the dialysis unit  Lack of privacy in IDE  Expectation for patient to access benefits and not work  Lack of time reducing effective delivery of IDE  Where IDE has specific logistical needs | “It might not work if we have a very busy period. We have a lot of patients who need a lot of care, that would become our priority and it wouldn’t be the bike.” Staff (Young, 2015)  “Nurses and techs, they’re on their toes all the time, they are busy and there’s the machines are alarming, someone you know needs attention all the time, and they are not like over-staffed, they are under-staffed so this would be something extra” Physician (Jhamb, 2016) |
|  |  | **Financial limitations**  *Where cost, particularly the cost of physical exercise equipment or staffing, is a barrier* | Cost of exercise facilities/equipment  Lack of resources  Cost of dedicated staff | “A lot of our patients are in the low-income category and live in low-income housing or apartments and can’t afford to pay for anything.” Staff (Painter, 2014)  “It would be a little bit expensive because I guess it would involve some sort of equipment.”  Physician (Zelko, 2023) |
|  |  | **Perceived environmental hazards for patients and nurses**  *Where there are hazards linked to the environments for physical activity in such as parks, gyms or even the IDE equipment in the dialysis centre for patients and nurses* | Reduced access to patients in emergency if IDE equipment in the way  Local environment risks  Facilities too crowded  Manual handling challenges for staff | The nurses were concerned that the lack of available floor space, combined with physical exercise, could hinder their access to patients in case of an emergency. Nurses preferred IE to take place in bed with equipment such as elastic exercise bands, small ankle and hand weights, or bed bikes, with which several of the nurses had experience. Staff (Wodskou, 2021)  ‘The nurses don’t like having to move that bike around so they don’t encourage it.…the nurses don’t because frankly they don’t like having to lug it out and move it around. That’s my honest impression. [laugh]’  Physician (Castillo, 2021) |
| Social Influences  *Opportunity – social*    “Those interpersonal processes that can cause individuals to change their thoughts, feelings, or behaviours” | Lack of support from social network  Lack of guidance from healthcare professionals  Family concern of ability  A lack of support from  management  Lack of ‘buy in’ from staff  Excessive support from staff encourages dependency | **Staff do not perceive any priority for PA in interpersonal relationships within the dialysis setting**  *Where dialysis staff are not perceived to be supportive of physical activity, or don’t provide necessary support. They also do not see other staff providing support, particularly at higher levels, nor do they receive social support for PA from their seniors.* | Lack of guidance for patients from healthcare professionals  A lack of support from  management  Lack of ‘buy in’ from staff  Excessive support from staff encourages dependency | “I think that staff are already very, very busy and to throw something else at them I think will get an emotional response. There’ll be resistance based on workload’ Physician (Castillo, 2021) |
|  |  | **Staff perceive that patients lack support from their personal social network**  *Where patients’ social network do not support physical activity. This could include friends, family, partners or others they engage with, and could be lack of support or clear discouragement* | Lack of support from social network  Family concern of ability | One-third of participants reported that insufficient support from family is a barrier in IDE. About a quarter reported missing support from caregivers.  Staff (Zelko, 2023) |
| Emotion  *Motivation – automatic*    “A complex reaction pattern, involving experiential, behavioural and physiological elements by which the individual attempts to deal with a personally significant matter or event” | *No barriers coded* |  |  |  |
| Behavioural Regulation  *Capability – psychological*  “Anything aimed at managing or changing objectively observed or measured actions” | Used to being sedentary | **Staff perceive patients as accustomed to sedentary lifestyle**  *Where patients are used to being sedentary, and have no habit of taking part in physical activity* | Used to being sedentary | All participants believed that patients had a minimal history of regular physical activity and remained inactive during their lives with nephrology disease. Their sedentary lifestyle and beliefs that physical activity is not important for their lives were identified as major barriers on the patients’ side for initiating IDE  Staff group including physicians(Zelko, 2023) |
